# Photoredox radical/polar crossover enables carbo-heterofunctionalization of alkenes: facile access to 1,3-difunctionalized nitro compounds†

**DOI:** 10.1039/d4cc06005a

**Published:** 2024-12-20

**Authors:** Subrata Patra, Vasiliki Valsamidou, Bhargav N. Nandasana, Dmitry Katayev

**Affiliations:** a Department of Chemistry, Biochemistry and Pharmaceutical Sciences, University of Bern Freiestrasse 3 3012 Bern Switzerland dmitry.katayev@unibe.ch

## Abstract

Herein, we present an efficient and practical method for multicomponent carbo-heterofunctionalization of alkenes *via* radical-polar crossover photoredox catalysis. Employing geminal bromonitroalkanes as redox-active reagents with a wide range of O-centered nucleophiles allows rapid access to various 1,3-difunctionalized nitro compounds, including β-nitro ketones, 1,3-nitro alcohols, 1,3-nitro ethers as well as cyclic molecules.

## Abstract 2

We report a method for carbo-heterofunctionalization of alkenes *via* radical-polar crossover photoredox catalysis, enabling the synthesis of 1,3-difunctionalized nitro compounds from redox-active organic reagents as nitroalkyl radical precursors.

## Graphical abstract

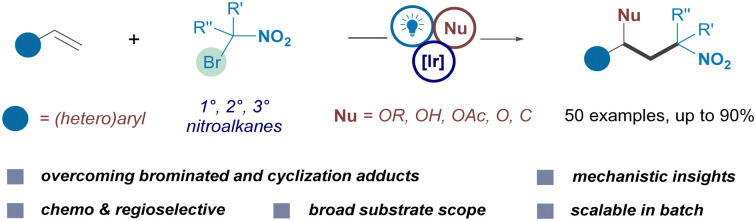

Alkenes, readily accessible from renewable feedstocks, serve as versatile intermediates in organic synthesis and are essential for constructing complex molecules.^1^ Selective difunctionalization of alkenes, especially radical-mediated approaches, efficiently increases molecular complexity.^2^ Nitrative difunctionalization of alkenes enables the preparation of substituted nitroalkanes, which have broad applications in synthesis,^3^ biology,^4^ materials science,^5^ and agrochemistry.^6^ This highlights the demand for efficient and streamlined methods to access nitro-functionalized compounds.^7^ The nitro group also serves as a versatile precursor for amines, aldehydes, and carboxylic acids, further emphasizing their importance.^3,8^ As a result, the development of synthetic methods for nitro-derived molecules has garnered considerable attention. While the 1,2-nitrative difunctionalization of alkenes is well-established, 1,3-difunctionalization remains relatively underexplored.^9^ This approach, particularly with the concurrent installation of O-centered substituents, offers efficient pathways to β-nitro ketones, 1,3-nitro alcohols, and ethers, valuable building blocks for organic synthesis.^10^ In addition, these nitro derivatives can be reduced to important intermediates, including 1,3-amino ketones and 1,3-amino alcohols.^11^

*gem*-Halonitroalkanes are known for their ability to introduce nitro-derived motifs, with their α-acidic protons making them well-suited for various nucleophilic and cycloaddition reactions.^12^ Recent advancements in photoredox activation of redox-active reagents have allowed the utilization of *gem*-halonitroalkanes in radical functionalization of alkenes.^13^ The Ooi group developed a photocatalytic system for reaction of α-bromonitroalkanes with styrenes, yielding either γ-bromo nitroalkanes or isoxazoline-*N*-oxides, though in moderate yields (Scheme 1A).^14^ The Reiser^15^ and Jiao^16^ groups also explored *gem*-halonitroalkanes in photocatalytic nitroalkylation of alkenes and silyl enol ethers, respectively.

**Scheme 1:**
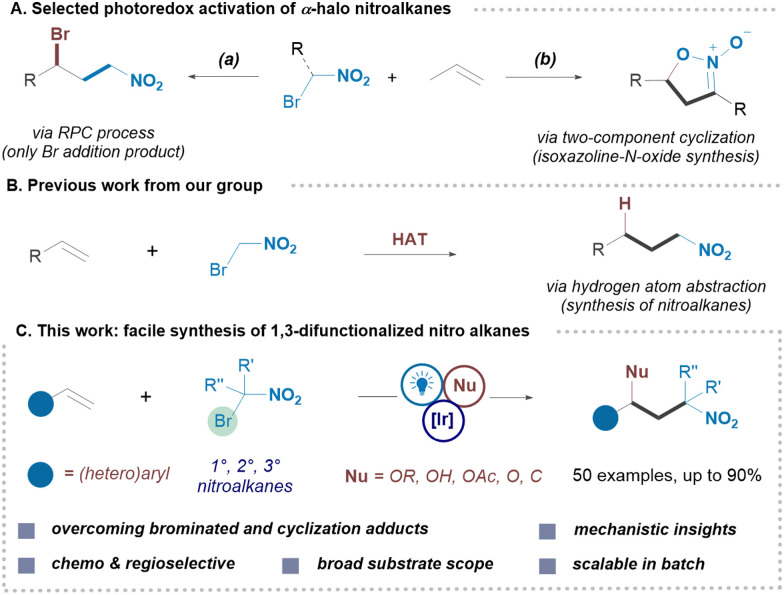
(A) Previous photoredox activation of α-halo nitroalkanes. (B) Our work on synthesis of nitroalkanes. (C) This work: direct access to the 1,3-difunctionalized nitro compound from olefins.

Our group has long focused on developing sustainable methods for the synthesis of nitro compounds, including an approach based on radical nitrative difunctionalization of olefins.^17^ Very recently we revealed anti-Markovnikov hydronitration and hydronitroalkylation of alkenes to access terminal nitroalkanes.^18^ Using thiol-based hydrogen atom donors, we successfully inhibited the formation of brominated and isoxazoline-*N*-oxide adducts (Scheme 1B). Building on this process, we proposed that the transient alkyl radical (Giese-type intermediate) formed by addition of nitroalkyl radicals could be oxidized to a carbocation using a photocatalyst. Sequestering bromide ions reduces bromide's role as a nucleophile, paving the way for diverse nucleophilic reactions and facilitating the synthesis of novel 1,3-difunctionalized nitro compounds.

We began the reaction development using 4-*tert*-butylstyrene 1 as a model substrate, bromonitromethane as a redox-active reagent (*E*^red^_1/2_ = −0.87 V *vs.* SCE), and ethanol as a nucleophile. After screening various parameters (see the ESI† for details), we observed the desired reactivity in the presence of only 0.5% Ir-based photocatalyst, Ag_2_CO_3_ (0.7 equiv.), EtOH (5.0 equiv.) in MeCN under 440 nm visible light irradiation for 8 hours (Table 1, entry 1). Attempts to enhance the reactivity using other classes of photocatalysts have proven unsuccessful (entries 2 and 3), while MeCN as a solvent provided the best conversion (entries 4 and 5). Interestingly, silver salts (entries 6–8) act as effective halogen scavengers to prevent side reactions, with Ag_2_CO_3_ standing out for its ability to efficiently suppress C–Br bond formation and enable a seamless RPC reaction.^16*b*^ After identifying optimal conditions, we proceeded to evaluate the substrate scope by testing several styrene derivatives, alcohols, and *gem*-bromonitroalkanes as reagents.

**Table 1:** Investigation of the reaction conditions

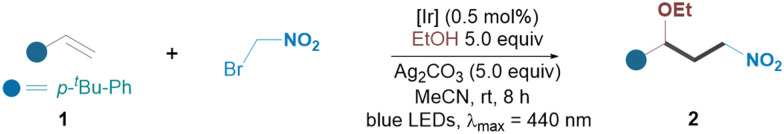		
Entry	Variation of the optimal conditions^a^	2^b^ (%)
1	None	91 (87)^c^
2	[Ru]/[Mes-Acr]	88/3
3	[PTH]/[4CzIPN]	31/81
4	DMF/THF	0/6
5	DCE/DMC	31/25
6	AgNO_2_/AgNO_3_	16/27
7	CF_3_CO_2_Ag/PhCO_2_Ag	57/11
8	Na_2_CO_3_/Cs_2_CO_3_/K_2_CO_3_	Up to 42

a Reaction conditions: 1 (1.0 equiv.), [Ir] (0.5 mol%), BrCH_2_NO_2_ (1.4 equiv.), Ag_2_CO_3_ (0.7 equiv.), EtOH (5.0 equiv.), MeCN (0.04 M), blue LEDs, rt, 8 h, N_2_.

b Yields were determined by GC-MS against *n*-decane as an internal standard.

c Isolated yield. [Ir] = *fac*-Ir(ppy)_3_; [Ru] = Ru(bpy)_3_(PF_6_)_2_.

Common functionalities at *o*- and *p*-positions of styrenes were examined, revealing a great level of site-selectivity, with product yields ranging from 81 to 90% (Scheme 2A). Notably, the benzylic chlorine in 5 remained intact under established conditions. Naphthalene and thiazole derivatives also exhibited great reactivity (7, 10). Likewise, α,α-disubstituted and α,β-disubstituted olefins provided the corresponding products 8 and 9 in good yields. Varying alcohols as shown in Scheme 2B did not significantly affect the outcome of the reaction suggesting that both linear and branched alcohols can be employed. We then utilized substituted *gem*-bromo-nitroalkanes as reagents, obtaining secondary nitroalkanes from 1-bromo-1-nitroethane and tertiary nitroalkanes from 2-bromo-2-nitropropane or 2-bromo-2-nitro-1,3-dioxane. These highly substituted adducts 14–16 were isolated in excellent yields with great selectivity. To demonstrate the scalability of our protocol, we extended the reaction time for substrate 1 to 24 hours in a batch process (10.0 mmol), achieving an isolated yield of 84%. We next applied our strategy to the intramolecular processes, including radical-triggered lactonization and cycloetherification of olefins as well as semipinacol-type rearrangements (Scheme 3A). For example, carboxylic acid and alcohol derivatives underwent smooth cycloaddition reactions, yielding the corresponding lactones or substituted furans with nitro groups in their structures. Allylic alcohol derivative underwent a semipinacol-type rearrangement, forming an all-carbon quaternary γ-nitro ketone 22. Bronopol (2-bromo-2-nitropropane-1,3-diol), a widely used antimicrobial preservative, pharmaceutical, and industrial product, was also explored as a reagent.^19^ The intriguing property of this molecule is its potential to be used as a bifunctional reagent, and we were pleased to see that a number of highly substituted furan derivatives can be easily generated in excellent isolated yields (23–29) (Scheme 3B). We envision that this method will find application in the synthesis of polycyclic molecules. Since the protocol generates a carbocation intermediate, we hypothesized that conducting the reaction in the presence of DMSO would facilitate rapid access to biologically important β-nitro ketones (Scheme 4A). Notably, various styrene derivatives featuring electron-donating and electron-withdrawing aryl substituents were efficiently difunctionalized (30–39). Varying different *gem*-bromonitroalkanes in the presence of DMSO has also resulted in the formation of β-nitro ketones with nitro groups located at secondary or tertiary carbon centres (40–42). The direct synthesis of 1,3-nitro alcohols can also be achieved by switching the nucleophile to water (43–46) (Scheme 4B). Notably, when no external nucleophiles were used and Ag_2_CO_3_ was replaced with AgOAc, the latter served as both a trap for halogen ions and a source of nucleophile, resulting in the formation of 1,3-nitroesters 47–50 (Scheme 4C).

**Scheme 2:**
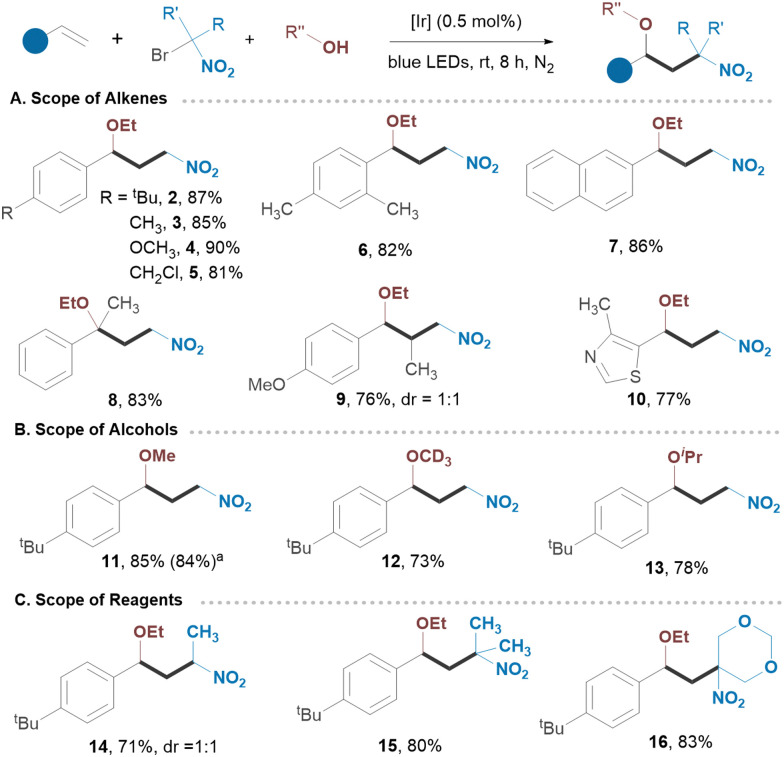
(A)–(C) Synthesis 1,3-nitroethers. Conditions: alkene (0.2 mmol, 1.0 equiv.), *fac*-[Ir(ppy)_3_] (0.5 mol%), reagent (1.4 equiv.), Ag_2_CO_3_ (0.7 equiv.), ROH (5.0 equiv.), MeCN, blue LEDs, rt, 8 h; yields refer to isolated products.^ *a*^ Scale-up in batch: 10 mmol of alkene, 24 h.

**Scheme 3:**
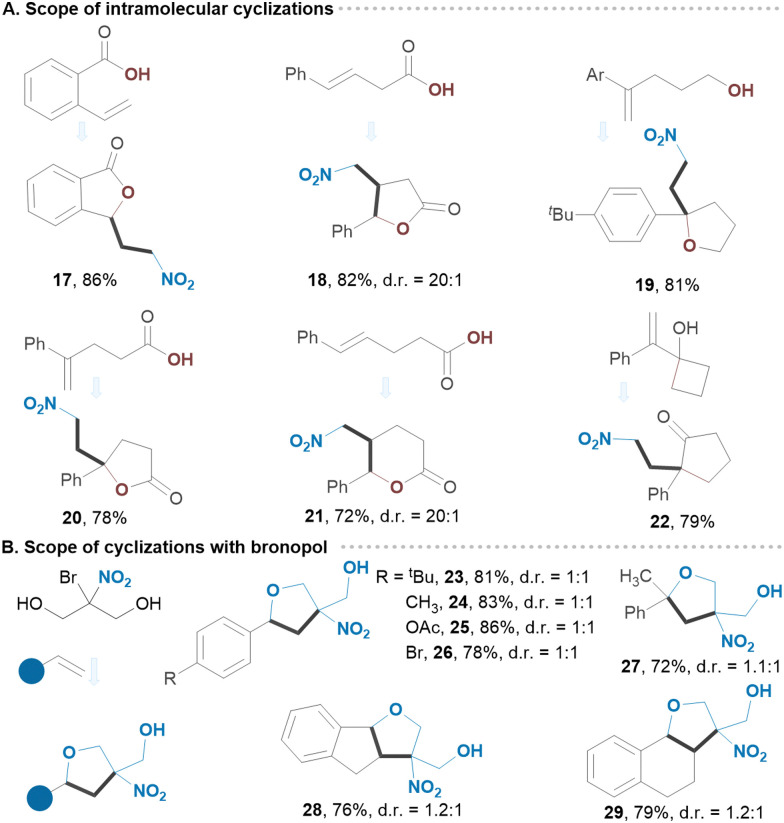
(A) and (B) Scope of intramolecular cyclizations using bromonitromethane and bronopol as redox active reagents.

**Scheme 4:**
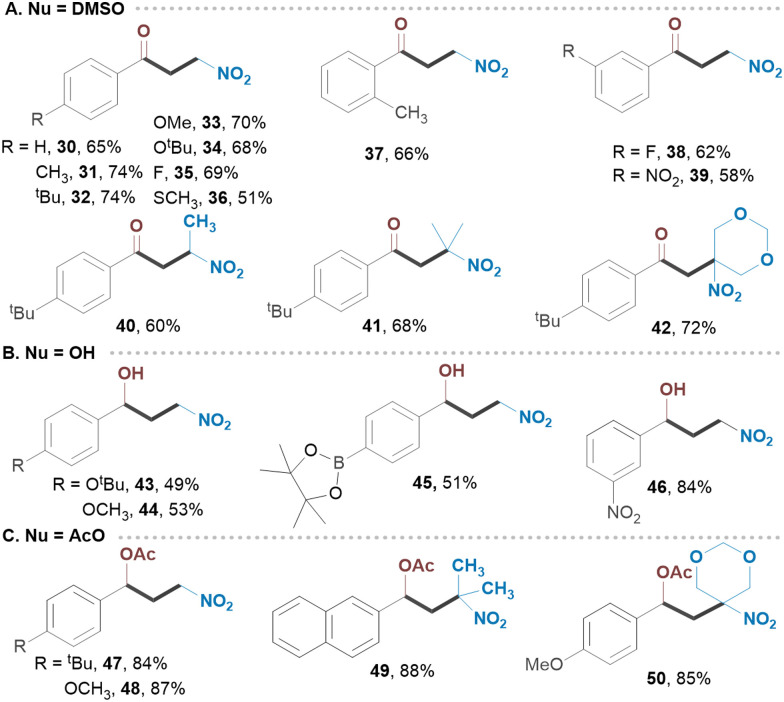
(A)–(C) Scope of O-centered nucleophiles.

Following the successful exploration of the substrate scope, we then examined the reaction mechanism. Control experiments (Scheme 5A) highlighted the crucial roles of both the photocatalyst and light. No reaction was observed, even under heating at 90 °C. Adding radical scavengers, such as TEMPO or BHT completely inhibited the reaction, indicating a likely radical pathway. The reaction occurred smoothly under visible light but halted immediately in its absence, as confirmed by on–off experiments, indicating a photoredox catalytic pathway rather than a radical chain (Scheme 5B). The reaction of the unactivated alkene 1-decene led to no product formation, suggesting the RPC mechanism, as the Ir^IV^ species is not expected to effectively oxidize the alkyl radical intermediate. Also, no product 53 was found when subjecting 54 to our conditions (Scheme 5C). Interestingly, a radical clock experiment did not yield the expected product 51a (Scheme 5D). However, in the presence of the HAT reagent, the ring-opening product 51b was obtained, supporting the involvement of an alkyl radical intermediate. Furthermore, a competition reaction experiment with increasing amounts of the HAT reagent showed that as the HAT reagent increases, hydride addition product formation also increases, with 2 equivalents yielding product 52 exclusively (Scheme 5E). This suggests that the HAT process with a Giese-type intermediate outpaces its oxidation to the corresponding carbocation by Ir^IV^. Building on this experimental evidence and our previous findings, we propose that the reagent undergoes a reductive single electron transfer (SET) process, yielding an electrophilic α-nitroalkyl radical^20^ (i) and a bromide ion, with the latter being trapped by the silver salt (Scheme 5F). The high global electrophilicity of nitroalkyl radicals (Scheme 5F) can be attributed to the stabilization of radicals *via* its π-delocalization with electron-withdrawing nitro group. In a subsequent reaction with alkene, the intermediate ii is formed and then oxidized to carbocation iii by a photocatalyst. The presence of nucleophiles enables to tuning the reactivity of carbocation, providing access to 1,3-disubstituted nitro compounds.

**Scheme 5:**
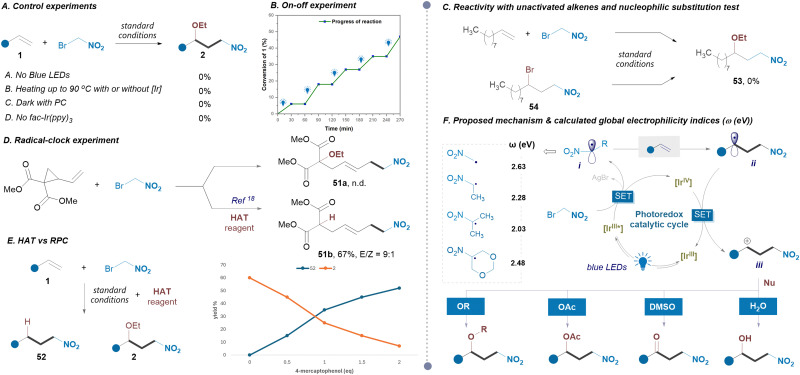
Mechanistic studies (A)–(E) and plausible reaction pathway (F). TEMPO = (2,2,6,6-tetramethylpiperidin-1-yl)oxyl, BHT = butylated hydroxytoluene.

In summary, we have demonstrated that α-bromo nitroalkanes can act as redox-active reagents and, in the presence of silver carbonate, be employed in a net-neutral radical-polar crossover (RPC) strategy. This approach enables diverse nitrative difunctionalization reactions with O-centered nucleophiles as coupling partners, achieving excellent regioselectivity. The ongoing exploration of *gem*-halonitroalkanes as reagents in molecular design using RPC and radical ligand transfer (RLT) mechanisms, as well as the role of silver salts in suppressing the ATRA process, is currently being investigated by our group.

D. K. acknowledges the Swiss National Science Foundation (SNSF, PCEFP2_186964) and the University of Bern for financial support of this research. We thank Dr A. J. Fernandes for calculating the philicity parameters of nitroalkyl radicals.

## Data availability

The data supporting this article (original ^1^H and ^13^C NMR spectra, HRMS) have been included as part of the ESI.† For the known compounds, we provide our spectra with the corresponding literature references. The manuscript does not contain X-ray data. The ESI† file includes additional optimization tables that are not part of the manuscript.

## Conflicts of interest

There are no conflicts to declare.

## Supplementary Material

CC-061-D4CC06005A-s001
