# Correction: Photoredox radical/polar crossover enables carbo-heterofunctionalization of alkenes: facile access to 1,3-difunctionalized nitro compounds

**DOI:** 10.1039/d5cc90151k

**Published:** 2025-05-01

**Authors:** Subrata Patra, Vasiliki Valsamidou, Bhargav N. Nandasana, Dmitry Katayev

**Affiliations:** a Department of Chemistry, Biochemistry and Pharmaceutical Sciences, University of Bern Freiestrasse 3 3012 Bern Switzerland dmitry.katayev@unibe.ch

## Abstract

Correction for ‘Photoredox radical/polar crossover enables carbo-heterofunctionalization of alkenes: facile access to 1,3-difunctionalized nitro compounds’ by Subrata Patra *et al.*, *Chem. Commun.*, 2025, **61**, 1689–1692, https://doi.org/10.1039/D4CC06005A.

The authors regret that the following text was not included in the acknowledgements in the original article:

“The photoreactor used in the present work is a modified version of the initial design conceived by B. Jelier in collaboration with the mechanical workshop of the Department of Chemistry and Applied Biosciences at ETH Zurich, as reported in ref. 1 listed here.”

The Royal Society of Chemistry apologises for these errors and any consequent inconvenience to authors and readers.
