# Supplementary material for: Photoredox radical/polar crossover enables carbo-heterofunctionalization of alkenes: facile access to 1,3-difunctionalized nitro compounds
Source: Chem Commun (Camb). 2024 Dec 20;61(8):1689–92. doi: 10.1039/d4cc06005a (PMC11696862; doi:10.1039/d4cc06005a)
Supplement: CC-061-D4CC06005A-s001 [file CC-061-D4CC06005A-s001.pdf]

Supplementary Information (SI) for ChemComm.
This journal is © The Royal Society of Chemistry 2024

Supporting Information

Photoredox Radical/Polar Crossover Enables Carbo-Heterofunctionalization of Alkenes: Facile Access to 1,3-Difunctionalized Nitro Compounds

Subrata Patra, Vasiliki Valsamidou, Bhargav Nandasana, Dmitry Katayev*

Correspondence to: dmitry.katayev@unibe.ch

Contribution from:

[1] *Department of Chemistry, Biochemistry, and Pharmaceutical Sciences, University of Bern (UNIBE), Freiestrasse 3, 3012 Bern (Switzerland)*

Table of Contents

1. General Information S3

   1.1. *Material and methods* *S3*

   1.2. *High intensity photoreactors* *S4*

2. Development of the Reaction Conditions S5

   2.1. *Survey of Photocatalysts* *S5*

   2.2. *Survey of solvents* *S6*

   2.3. *Survey of silver salts and bases* *S6*

   2.4. *Control experiments* *S7*

3. Availability of Starting Materials S8

   3.1. *Commercially available starting materials* *S8*

   3.2. *Prepared starting materials* *S9*

4. General Procedures S11

5. On-Off Experiment S13

6. Radical Trapping Experiment with TEMPO and BHT S14

7. HAT vs RPC Experiment S15

8. Ractivity with Unactivated Alkene S16

9. Radical-clock Experiment S16

10. Scale-up Synthesis in Batch S17

11. Determination of the Philicity Parameters S17

12. NMR Data S18

13. NMR Spectra of Isolated Compounds S34

14. References S137

1. General Information

1.1. Material and methods

All reactions were conducted using flame-dried glassware under an inert argon atmosphere, equipped with Teflon-coated magnetic stirring bars and secured with dry septa. Glassware was pre-dried at 120 °C overnight to ensure dryness before use. Starting materials were obtained commercially from Thermo Fisher Scientific (Acros), Sigma-Aldrich, Apollo Scientific, Fluorochem, and TCI unless otherwise specified. To ensure quality, all commercially available olefins were characterized via ^{1}H-NMR spectroscopy prior to use. Anhydrous acetonitrile was stored over preconditioned 3 Å molecular sieves.

Analytical thin-layer chromatography (TLC) was performed on Merck silica gel 60 F254 glass plates, visualized under 254 nm UV light or stained with potassium permanganate solution and heated. Product purification was achieved using flash chromatography on Brunschwig silica gel (32-63 µm, 60 Å) with a pressure of 0.3-0.5 bar. Medium-pressure liquid chromatography (MPLC) was performed on a Teledyne ISCO CombiFlash Rf200 System equipped with UV detection and fraction collection, or manually with SilicaFlash P60, 40-63 µm. Preparative HPLC was done using a Teledyne Isco CombiFlash EZ Prep system fitted with Macherey-Nagel VP 250/21 Nucleosil 50-5 columns.

Nuclear magnetic resonance (NMR) spectra were recorded on Bruker Ultrashield 300 and Bruker Ascend 400 spectrometers, with ^{1}H and ^{13}C-NMR at 300.1 MHz and 75.5 MHz. Fluorine NMR ^{19}F spectra were acquired on Bruker DPX-300, Ultrashield 300. Chemical shifts (δ) are reported in ppm relative to solvent peaks (e.g., $CDCl_3$ at 7.26 ppm for ^{1}H, $CDCl_3$ at 77.16 ppm for ^{13}C). Signals are labeled as singlet (s), doublet (d), triplet (t), quartet (q), multiplet (m), with coupling constants (*J*) in Hz.

High-resolution mass spectrometry (HR-MS) with electrospray ionization (ESI+) was conducted on a Bruker FTMS 4.7T BioAPEX II or Thermo Scientific LTQ Orbitrap XL, including electron impact ionization (EI) on a VG-TRIBRID system. Gas chromatography-mass spectrometry (GC-MS) was performed on an Agilent 8890 series GC coupled with an Agilent 5977B mass selective detector.

1.2. High intensity photoreactors

The photoreactor was custom designed and built by Katayev and co-workers in coordination with the mechanical workshop in the Department of Chemistry and Applied Biosciences at ETH Zürich having blue LEDs, equally spaced in a circular design, powered by a 10.3 A power supply, emitting 350 W of light with the measured UV-Vis spectrum (Figure 1). The LEDs were water-cooled and further cooled by built-in fans to maintain an ambient temperature.

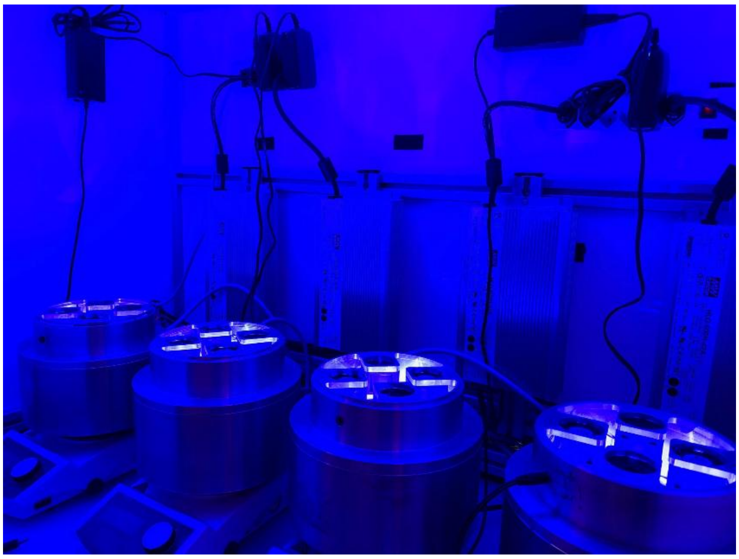

Figure 1.1. Custom high-intensity, blue LED photoreactors for photocatalytic reactions.

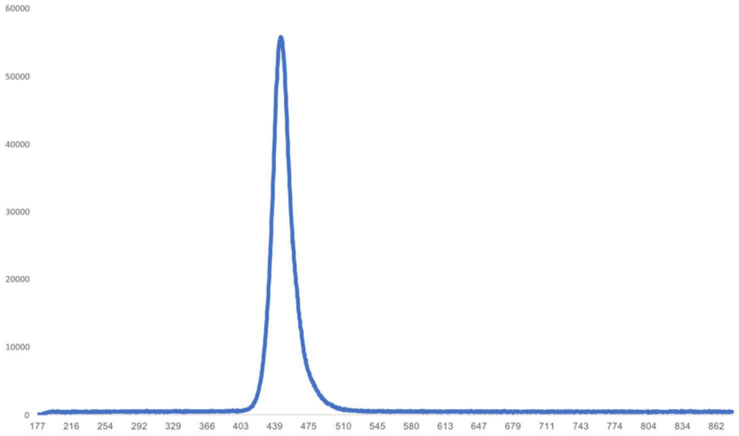

Figure 1.2. UV-Vis emission spectrum of high-intensity, blue LED photoreactor (λ_{max} = 440 nm, FWHM = 20 nm). The figure is taken from.[1]

2. Development of the Reaction Conditions

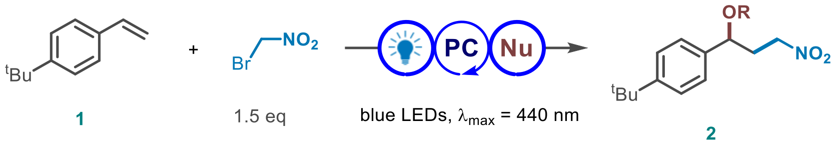

A flame-dried 5 mL crimp cap vial was charged with photocatalyst (PC) (*x* mol%), bromo(nitro)methane (*x* equiv), additive (*x* equiv) nucleophile (*x* equiv), and equipped with a magnetic bar. The vial contents were then subject to three vacuum/N_2 cycles. Anhydrous solvent (*x* mL) and 4-*tert*-butylstyrene **1** (0.5 mmol, 1.0 equiv) were introduced to the solution *via* syringes under an N_2 atmosphere. The reaction mixture was irradiated at room temperature under blue LEDs for 8 h. After that, *n*-decane as an internal standard was added with a microsyringe. An aliquot was taken and analyzed by GC-MS to obtain the calibrated yields for the desired product.

2.1. *Survey of Photocatalysts*

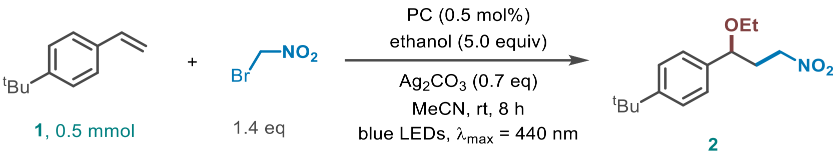

**Entry**[a]	**PC** [0.5 mmol]	**Yield of 2** [%][b]
1	PC1	91 (87)
2	PC2	88
3	PC3	3
4	PC4	31
5	PC5	81

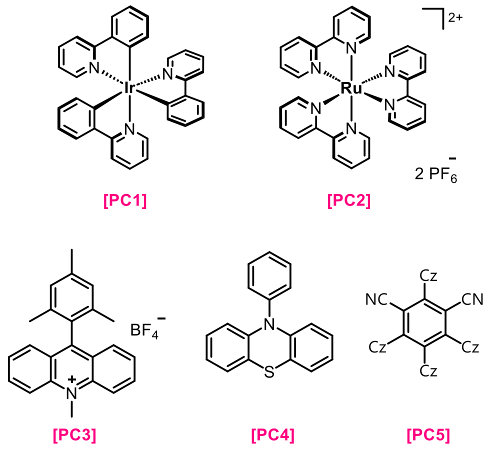

Table 2.1. [a] Reaction conditions: 4-*tert*-butylstyrene (0.5 mmol, 1.0 equiv), bromo(nitro)methane (1.4 equiv), *PC* (0.5 mol%), Ag_2CO_3 (0.7 equiv), ethanol (5.0 equiv) and MeCN (1.25 mL), 350 W blue LEDs, rt, 8 h. [b] Determined by GC-MS against an internal standard of *n*-decane. [c] Isolated yield. Cz = carbazole.

2.2. *Survey of solvents*

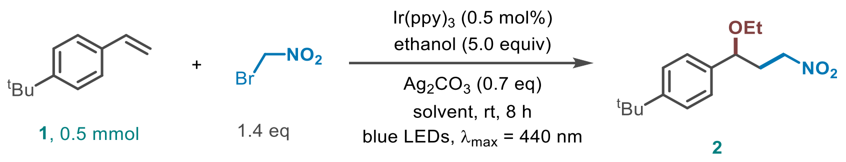

Entry[a]	solvent	yield of 2 [%][b]
1	MeCN	91 (87)[c]
2	THF	6
3	DCE	31
4	$OC(OCH_3)_2$	25
5	DMF	0

Table 2.2. [a] Reaction conditions: 4-*tert*-butylstyrene (0.5 mmol, 1.0 equiv), bromo(nitro)methane (1.4 equiv), *fac*-Ir(ppy)$_3$ (0.5 mol%), Ag_2CO_3 (0.7 equiv), ethanol (5.0 equiv) and solvent (1.25 mL), 350 W blue LEDs, rt, 12 h. [b] Determined by GC-MS against an internal standard of *n*-decane.

2.3. *Survey of silver salts and bases*

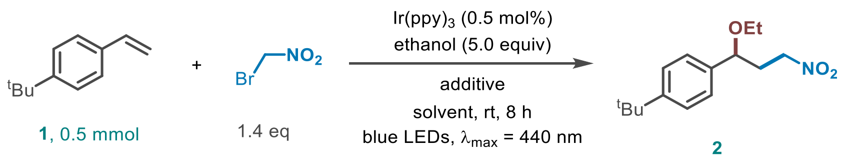

Entry[a]	Base (eq)	yield of 2 [%][b]
1	Ag_2CO_3 (0.7)	91 (87)[c]
2	$AgNO_2$	16
3	$AgNO_3$	27
4	CF_3CO_2Ag	57
5	$PhCO_2Ag$	11
6	Na_2CO_3	37
7	K_2CO_3	25
8	Cs_2CO_3	42
9	0.3 eq of Ag_2CO_3 instead of 0.7 equiv	53

Table 2.3. [a] Reaction conditions: 4-*tert*-butylstyrene (0.5 mmol, 1.0 equiv), bromo(nitro)methane (1.4 equiv), *fac*-Ir(ppy)$_3$ (0.5 mol%), Ag_2CO_3 (0.7 equiv), ethanol (5.0 equiv) and MeCN (1.25 mL), 350 W blue LEDs, rt, 12 h. [b] Determined by GC-MS against an internal standard of *n*-decane.

2.4. Control experiments

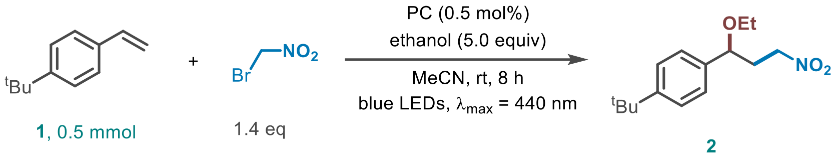

Entry	Variables	Yield of 2 [%][b]
1	Standard conditions[a]	91 (87)[c]
2	Without LEDs	0
3	Without **PC1**	0
4	Heating at 90 ˚C without light	0

Table 2.4. [a] Standard reaction conditions: 4-*tert*-butylstyrene (0.5 mmol, 1.0 equiv), bromo(nitro)methane (1.4 equiv), *fac*-Ir(ppy)$_3$ (0.5 mol%), Ag_2CO_3 (0.7 equiv), ethanol (5.0 equiv) and MeCN (1.25 mL), 350 W blue LEDs, rt, 12 h. [b] Determined by GC-MS against an internal standard of *n*-decane.

3. Availability of Starting Materials

3.1. Commercially available starting materials

Starting materials which are commercially available are mostly purchased from Thermoscientific – Acros, Sigma Aldrich, Apollo Scintific, Fluorochem and TCI.

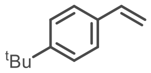

CAS
1746-23-2

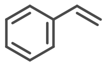

CAS
100-42-5

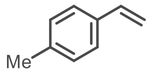

CAS
622-97-9

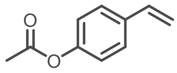

CAS
2628-16-2

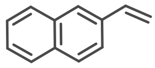

CAS
827-54-3

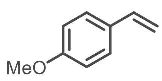

CAS
637-69-4

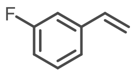

CAS
350-51-6

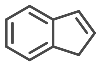

CAS
95-13-6

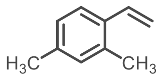

CAS
2234-20-0

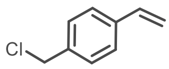

CAS
1592-20-7

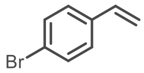

CAS
2039-82-9

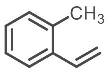

CAS
611-15-4

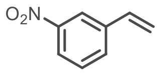

CAS
100-13-0

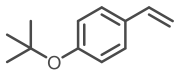

CAS
95418-58-9

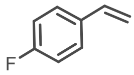

CAS
405-99-2

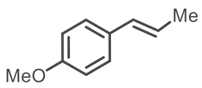

CAS
4180-23-8

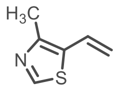

CAS
1759-28-0

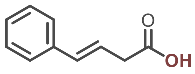

CAS
1914-58-5

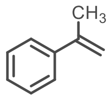

CAS
98-83-9

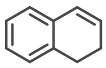

CAS
447-53-0

3.2. Prepared starting materials

General procedure A

To synthesize the desired alkene, methyltriphenylphosphonium bromide (3 equivalents) was first suspended in dry tetrahydrofuran (THF) at a concentration of 0.2 M, with the solution then cooled to 0°C. To this, tert-butoxide potassium (t-BuOK, 3 equivalents) was added in a single step, and the mixture was stirred for 30 minutes at 0°C. Following this, the ketone or aldehyde (1 equivalent) was introduced, and the reaction was gradually warmed to room temperature (RT) while stirring. Progress was monitored by thin-layer chromatography (TLC) and allowed to continue from 2 to 16 hours until completion. Upon completion, the reaction was quenched by adding water, and the resulting solution was extracted with three portions of ethyl acetate (EtOAc, 50 mL each). The organic layers were combined, washed with water, dried over sodium sulfate (Na_2SO_4), filtered, and concentrated under reduced pressure. The crude product was then purified via flash column chromatography on silica gel, yielding the pure alkene.

Methyl(4-vinylphenyl)sulfane

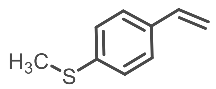

According to a general procedure A, the titled compound was obtained as yellow oil (77% yield, 5% ethyl acetate in hexane as eluent). The characterization data match the literature.[2]

^{1}H-NMR (300 MHz, $CDCl_3$): δ 7.34 (d, *J* = 8.3 Hz, 2H), 7.22 (d, *J* = 8.4 Hz, 2H), 6.67 (dd, *J* = 17.6, 10.9 Hz, 1H), 5.71 (dd, *J* = 17.6, 0.9 Hz, 1H), 5.21 (dd, *J* = 10.9, 0.9 Hz, 1H), 2.49 (s, 3H).

^{13}C-NMR (75 MHz, $CDCl_3$): δ 138.1, 136.3, 134.7, 126.8, 126.7, 113.4, 16.0.

4,4,5,5-Tetramethyl-2-(4-vinylphenyl)-1,3,2-dioxaborolane

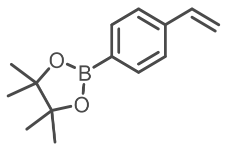

Pinacol (5.5 mmol, 1.1 equiv) was added in one portion to a solution of 4-vinylphenylboronic acid (5.0 mmol, 1.0 equiv) and MgSO4 (10 mol%) in THF (15.0 mL). After stirring the resulting mixture for 2 hours at room temperature, it was filtered and concentrated under a vacuum. The crude product was then purified by column chromatography on silica gel using Hexanes: EtOAc 95:5 (v/v), and the titled compound was obtained as a colorless oil. The characterization data match the literature.[3]

^{1}H-NMR (300 MHz, $CDCl_3$): δ 7.79 (d, J = 8.1 Hz, 2H), 7.42 (d, J = 8.0 Hz, 2H), 6.74 (dd, J = 17.6, 10.9 Hz, 1H), 5.82 (dd, J = 17.6, 1.0 Hz, 1H), 5.30 (dd, J = 10.8, 0.9 Hz, 1H), 1.36 (s, 12H).

^{13}C-NMR (75 MHz, $CDCl_3$): δ 140.3, 137.0, 135.2, 125.6, 115.0, 83.9, 25.0.

2-Vinylbenzoic acid

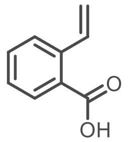

According to a general procedure A, the titled compound was obtained as white solid (88% yield, 10% ethyl acetate in hexane as eluent). The characterization data match the literature.[4]

^{1}H-NMR (300 MHz, $CDCl_3$): δ 11.78 (br, 1H), 8.05 (dt, *J* = 11.0, 1.3, 1H), 7.66 – 7.52 (m, 3H), 7.42 – 7.33 (m, 1H), 5.68 (dd, *J* = 17.4, 1.3, 1H), 5.39 (dd, *J* = 11.0, 1.3, 1H).

4. General Procedures

4.1. GP1 for the synthesis of 1,3-nitro ethers

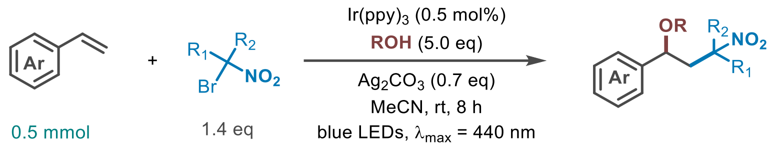

A flame-dried 5 mL crimp cap vial was charged with $Ir(ppy)_3$ (0.5 mol%), Ag_2CO_3 (0.7 equiv), and equipped with a magnetic bar. The vial contents were then subject to three vacuum/N_2 cycles. Anhydrous MeCN (1.25 mL), bromo(nitro)alkane (1.4 equiv), alcohol (5.0 equiv), and alkene (0.5 mmol, 1.0 equiv) were introduced to the solution via syringes under an N_2 atmosphere. The reaction mixture was irradiated at room temperature under blue LEDs for 8 h. The solvent was evaporated under reduced pressure, and the crude product was purified by flash column chromatography over silica gel as indicated to get the desired product.

4.2. GP2 for the cyclization reactions with bronopol

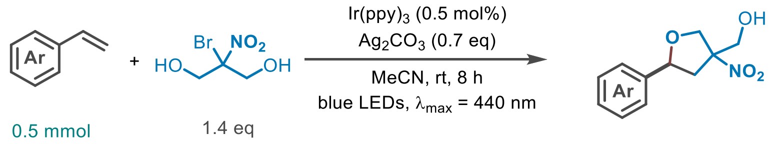

A flame-dried 5 mL crimp cap vial was charged with $Ir(ppy)_3$ (0.5 mol%), Ag_2CO_3 (0.7 equiv), bronopol (1.4 equiv), and equipped with a magnetic bar. The vial contents were then subject to three vacuum/N_2 cycles. Anhydrous MeCN (1.25 mL), and alkene (0.5 mmol, 1.0 equiv) were introduced to the solution via syringes under an N_2 atmosphere. The reaction mixture was irradiated at room temperature under blue LEDs for 8 h. The solvent was evaporated under reduced pressure, and the crude product was purified by flash column chromatography over silica gel as indicated to get the desired product.

4.3. GP3 for the synthesis of β-nitro ketones

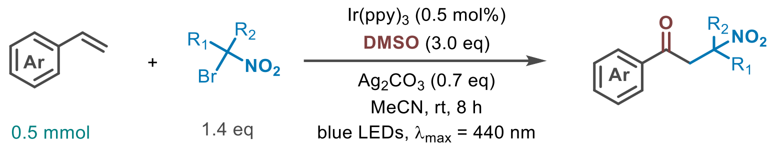

A flame-dried 5 mL crimp cap vial was charged with $Ir(ppy)_3$ (0.5 mol%), Ag_2CO_3 (0.7 equiv), and equipped with a magnetic bar. The vial contents were then subject to three vacuum/N_2 cycles. Anhydrous MeCN (1.25 mL), bromo(nitro)alkane (1.4 equiv), DMSO (3.0 equiv), and alkene (0.5 mmol, 1.0 equiv) were introduced to the solution via syringes under an N_2 atmosphere. The reaction mixture was irradiated at room temperature under blue LEDs for 8 h. The solvent was evaporated under reduced pressure, and the crude product was purified by flash column chromatography over silica gel as indicated to get the desired product.

4.4. GP4 for the synthesis of 1,3-nitro alcohols

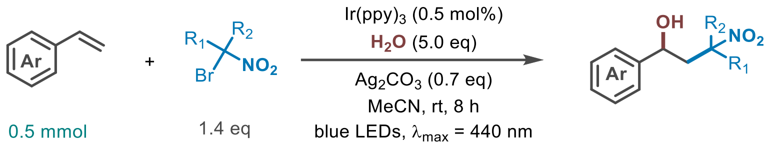

A flame-dried 5 mL crimp cap vial was charged with $Ir(ppy)_3$ (0.5 mol%), Ag_2CO_3 (0.7 equiv), and equipped with a magnetic bar. The vial contents were then subject to three vacuum/N_2 cycles. Anhydrous MeCN (1.25 mL), bromo(nitro)alkane (1.4 equiv), water (5.0 eq) and alkene (0.5 mmol, 1.0 equiv) were introduced to the solution via syringes under an N_2 atmosphere. The reaction mixture was irradiated at room temperature under blue LEDs for 8 h. The solvent was evaporated under reduced pressure, and the crude product was purified by flash column chromatography over silica gel as indicated to get the desired product.

4.5. GP5 for the synthesis of 1,3-nitro acetates

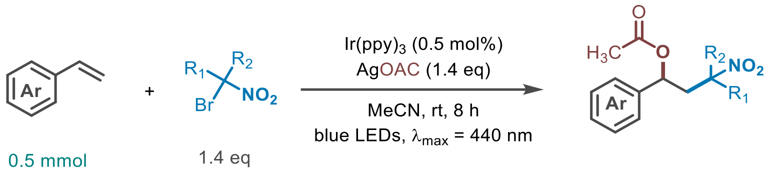

A flame-dried 5 mL crimp cap vial was charged with $Ir(ppy)_3$ (0.5 mol%), AgOAc (1.4 equiv), and equipped with a magnetic bar. The vial contents were then subject to three vacuum/N_2 cycles. Anhydrous solvent (1.3 mL), bromo(nitro)alkane (1.4 equiv), and alkene (0.5 mmol, 1.0 equiv) were introduced to the solution via syringes under an N_2 atmosphere. The reaction mixture was irradiated at room temperature under blue LEDs for 8 h. The solvent was evaporated under reduced pressure, and the crude product was purified by flash column chromatography over silica gel as indicated to get the desired product.

5. On-Off Experiment

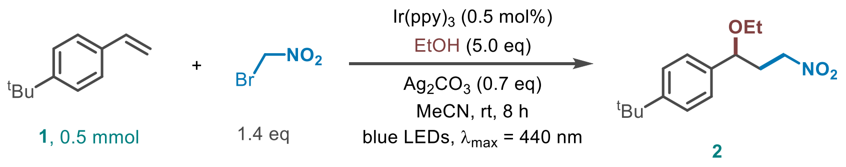

A flame-dried 5 mL crimp cap vial was charged with $Ir(ppy)_3$ (0.5 mol%), Ag_2CO_3 (0.7 equiv), and equipped with a magnetic bar. The vial contents were then subject to three vacuum/N_2 cycles. Anhydrous MeCN (1.25 mL), bromo(nitro)methane (1.4 equiv), ethanol (5.0 equiv), and 4-tert-butylstyrene **1** (0.5 mmol, 1.0 equiv) were introduced to the solution via syringes under an N_2 atmosphere. The reaction mixture was irradiated at room temperature under blue LEDs as following sequence: reaction mixture was stirred in the presence of blue LEDs for 30 min (interval with bulb) and then stirred at room temperature without LEDs for 30 min (interval without bulb). At the end of each time interval, aliquot of the reaction mixture was taken, filtrated through a short pot of silica gel and subjected to GC-MS analysis. Results of the experiments are presented in Figure 5.1.

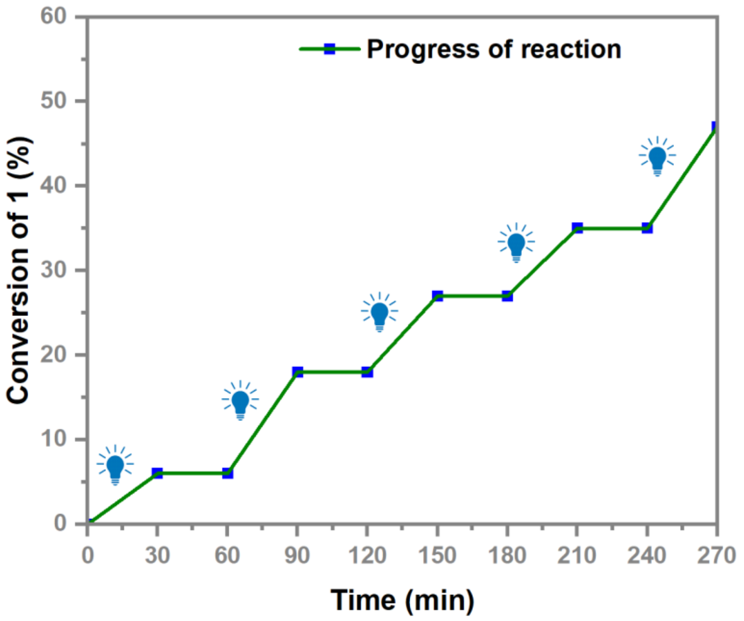

Figure 5.1. Conversion vs reaction time for the light on-off experiment.

6. Radical Trapping Experiment with TEMPO and BHT

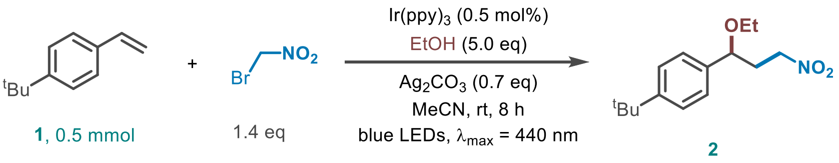

A flame-dried 5 mL crimp cap vial was charged with $Ir(ppy)_3$ (0.5 mol%), Ag_2CO_3 (0.75 equiv), additive TEMPO (2.0 equiv) or BHT (2.0 equiv) and equipped with a magnetic bar. The vial contents were then subject to three vacuum/N_2 cycles. Anhydrous MeCN (1.25 mL), bromo(nitro)methane (1.5 equiv), ethanol (5.0 equiv), and 4-tert-butylstyrene **1** (0.5 mmol, 1.0 equiv) were introduced to the solution via syringes under an N_2 atmosphere. The reaction mixture was irradiated at room temperature under blue LEDs for 8 h. An aliquot of the reaction mixture was taken, filtrated through a short pot of silica gel and subjected to GC-MS analysis. Results of the experiments are presented in Figure 6.1.

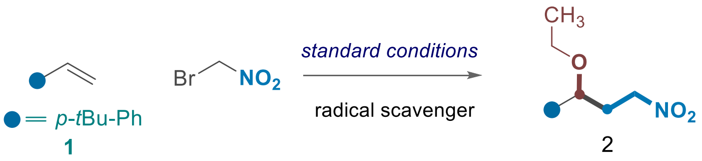

radical scavenger:	none	TEMPO	BHT
yield:	91%	0%	10%

Figure 6.1. Results of trapping experiments with TEMPO and BHT.

7. HAT vs RPC Experiment

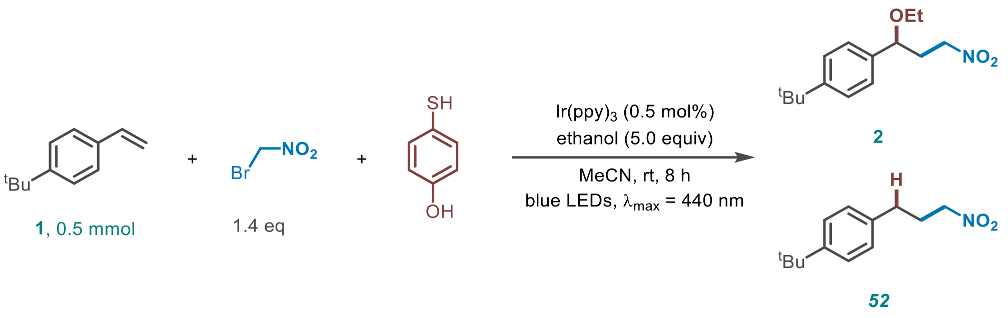

A flame-dried 5 mL crimp cap vial was charged with Ir(ppy)$_3$ (0.5 mol%), Ag_2CO_3 (0.7 equiv), additive 4-hydroxythiophenol (0.0 equiv, 0.5 equiv, 1.0 equiv, 1.5 equiv, 2.0 equiv in separate vials) and equipped with a magnetic bar. The vial contents were then subject to three vacuum/N_2 cycles. Anhydrous MeCN (1.25 mL), bromo(nitro)methane (1.4 equiv), ethanol (5.0 equiv), and 4-tert-butylstyrene **1** (0.5 mmol, 1.0 equiv) were introduced to the solution via syringes under an N_2 atmosphere. The reaction mixture was irradiated at room temperature under blue LEDs for 8 h. An aliquot of the reaction mixture was taken, filtrated through a short pot of silica gel and subjected to GC-MS analysis. Results of the experiments are presented in Figure 7.1.

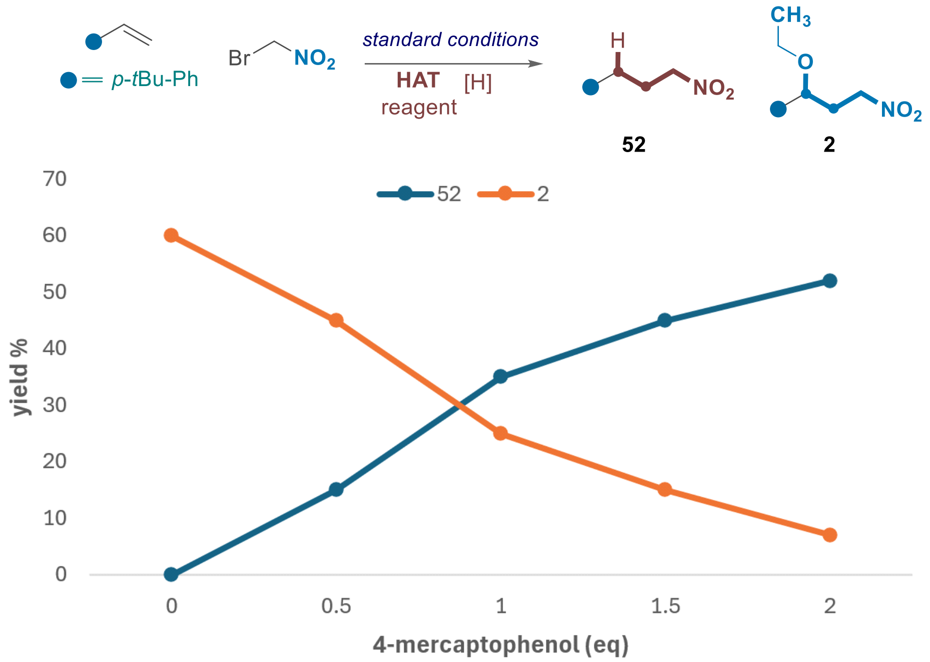

Figure 7.1. HAT vs RPC experiments.

8. Reactivity with Unactivated Alkene

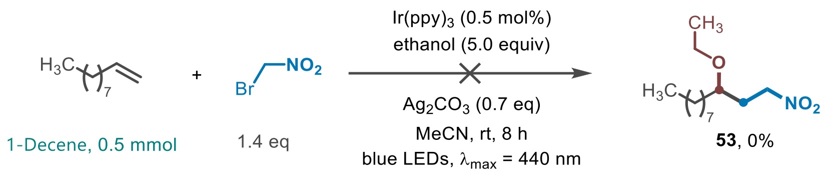

A flame-dried 5 mL crimp cap vial was charged with $Ir(ppy)_3$ (0.5 mol%), Ag_2CO_3 (0.7 equiv), and equipped with a magnetic bar. The vial contents were then subject to three vacuum/N_2 cycles. Anhydrous MeCN (1.25 mL), bromo(nitro)methane (1.4 equiv), ethanol (5.0 equiv), and 1-decene (0.5 mmol, 1.0 equiv) were introduced to the solution via syringes under an N_2 atmosphere. The reaction mixture was irradiated at room temperature under blue LEDs for 8 h. An aliquot of the reaction mixture was taken, filtrated through a short pot of silica gel and subjected to GC-MS analysis.

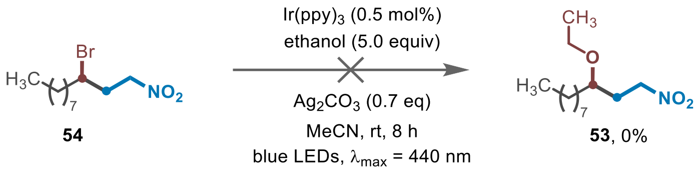

A flame-dried 5 mL crimp cap vial was charged with $Ir(ppy)_3$ (0.5 mol%), Ag_2CO_3 (0.7 equiv), and equipped with a magnetic bar. The vial contents were then subject to three vacuum/N_2 cycles. Anhydrous MeCN (1.25 mL), ethanol (5.0 equiv), and **54** (0.5 mmol, 1.0 equiv) were introduced to the solution via syringes under an N_2 atmosphere. The reaction mixture was irradiated at room temperature under blue LEDs for 8 h. An aliquot of the reaction mixture was taken, filtrated through a short pot of silica gel and subjected to GC-MS analysis.

9. Radical-clock Experiment

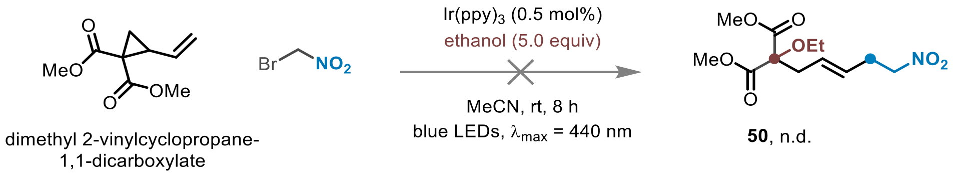

A flame-dried 5 mL crimp cap vial was charged with $Ir(ppy)_3$ (0.5 mol%), Ag_2CO_3 (0.7 equiv), and equipped with a magnetic bar. The vial contents were then subject to three vacuum/N_2 cycles. Anhydrous MeCN (1.25 mL), bromo(nitro)methane (1.4 equiv), ethanol (5.0 equiv), and dimethyl 2-vinylcyclopropane-1,1-dicarboxylate (0.5 mmol, 1.0 equiv) were introduced to the solution via syringes under an N_2 atmosphere. The reaction mixture was irradiated at room temperature under blue LEDs for 8 h. An aliquot of the reaction mixture was taken, filtrated through a short pot of silica gel and subjected to GC-MS analysis.

10. Scale-up Synthesis in Batch

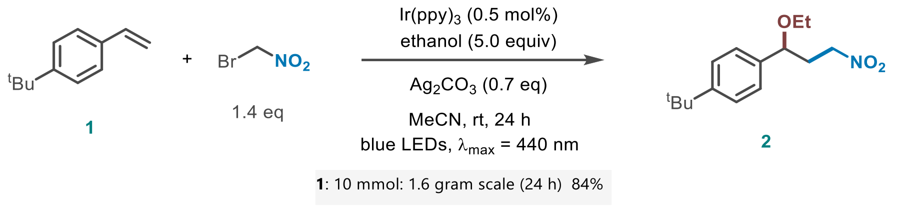

A flame-dried 5 mL crimp cap vial was charged with $Ir(ppy)_3$ (0.5 mol%), Ag_2CO_3 (0.7 equiv), and equipped with a magnetic bar. The vial contents were then subject to three vacuum/N_2 cycles. Anhydrous MeCN (25 mL), bromo(nitro)methane (1.4 equiv), ethanol (5.0 equiv), and 4-tert-butylstyrene **1** (0.5 mmol, 1.0 equiv) were introduced to the solution via syringes under an N_2 atmosphere. The reaction mixture was irradiated at room temperature under blue LEDs for 8 h.

11. Determination of the Philicity Parameters

The detailed computational procedure was described in a recent review published by us and the Houk group.

	Parameters in gas phase (U)B3LYP-D3(BJ)/6-311+G(d,p)					
**Nitroalkyl Radical**	**IP (eV)**	**EA (eV)**	***η* (eV)**	***μ* (eV)**	***ω* (eV)**	**ω^- (eV)**
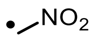	11.41	2.38	9.03	-6.89	2.631	0.116
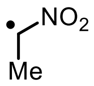	10.11	2.03	8.08	-6.07	2.280	0.168
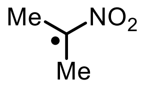	9.21	1.78	7.43	-5.49	2.032	0.208
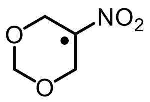	9.48	2.39	7.09	-5.94	2.485	0.171

Table 11. Computed values at the (U)B3LYP-D3(BJ)/6-311+G(d,p).

12. NMR Data

1-(tert-butyl)-4-(1-ethoxy-3-nitropropyl)benzene (2)

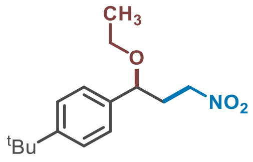

Compound **2** was obtained according to general procedure **GP1** as a colorless oil (87% yield) after purification by column chromatography (SiO_2, hexane/EA=4:1).

^{1}H-NMR (300 MHz, $CDCl_3$): δ 7.38 (d, *J* = 8.4 Hz, 2H), 7.21 (d, *J* = 8.2 Hz, 2H), 4.60 (dt, *J* = 13.3, 7.2 Hz, 1H), 4.43 (dt, *J* = 13.2, 6.4 Hz, 1H), 4.32 (t, *J* = 6.6 Hz, 1H), 3.41 (dq, *J* = 9.2, 7.0 Hz, 1H), 3.29 (dq, J = 9.2, 7.0 Hz, 1H), 2.34 (q, *J* = 6.7 Hz, 2H), 1.32 (s, 9H), 1.16 (t, *J* = 7.0 Hz, 3H).

^{13}C-NMR (75 MHz, $CDCl_3$): δ 151.2, 138.1, 126.1, 125.7, 78.2, 72.7, 64.5, 35.8, 34.7, 31.5, 15.3.

HRMS (ESI) *m/z*, $[M]^+$ calcd for $C_{15}H_{23}NO_3^+$: 265.1678; found 265.1676.

1-(1-ethoxy-3-nitropropyl)-4-methylbenzene (3)

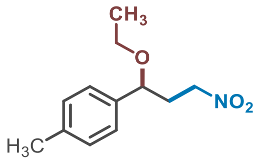

Compound **3** was obtained according to general procedure **GP1** as a colorless oil (85% yield) after purification by column chromatography (SiO_2, hexane/EA=4:1).

^{1}H-NMR (300 MHz, $CDCl_3$): δ 7.19 (d, *J* = 0.8 Hz, 4H), 4.59 (dt, *J* = 13.3, 7.1 Hz, 1H), 4.43 (dt, *J* = 13.2, 6.4 Hz, 1H), 4.32 (dd, *J* = 7.3, 6.0 Hz, 1H), 3.46 – 3.22 (m, 2H), 2.39 – 2.32 (m, 5H), 1.16 (td, *J* = 7.0, 0.6 Hz, 3H).

^{13}C-NMR (75 MHz, $CDCl_3$): δ 138.1, 137.9, 129.5, 126.4, 78.2, 72.6, 64.4, 35.8, 21.2, 15.2.

HRMS (ESI) *m/z*, $[M]^+$ calcd for $C_{12}H_{17}NO_3^+$: 223.1208; found 223.1205.

1-(1-ethoxy-3-nitropropyl)-4-methoxybenzene (4)

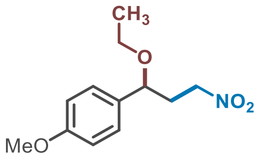

Compound **4** was obtained according to general procedure **GP1** as a light-yellow oil (90% yield) after purification by column chromatography (SiO_2, hexane/EA=4:1).

^{1}H-NMR (300 MHz, $CDCl_3$): δ 7.22 (d, *J* = 8.7 Hz, 2H), 6.90 (d, *J* = 8.8 Hz, 2H), 4.58 (ddd, *J* = 13.2, 7.5, 6.6 Hz, 1H), 4.42 (dt, *J* = 13.3, 6.5 Hz, 1H), 4.29 (dd, *J* = 8.1, 5.3 Hz, 1H), 3.81 (s, 3H), 3.43 – 3.20 (m, 2H), 2.39 – 2.27 (m, 2H), 1.15 (t, *J* = 7.0 Hz, 3H).

^{13}C-NMR (75 MHz, $CDCl_3$): δ 159.6, 133.1, 127.7, 114.2, 78.0, 72.7, 64.3, 55.4, 35.8, 15.3.

HRMS (ESI) *m/z*, $[M+Na]^+$ calcd for $C_{12}H_{17}NO_4+Na^+$: 262.1055; found 262.1053.

1-(chloromethyl)-4-(1-ethoxy-3-nitropropyl)benzene (5)

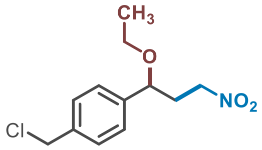

Compound **5** was obtained according to general procedure GP1 as a light-yellow oil (81% yield) after purification by column chromatography (SiO2, hexane/EA=4:1).

^{1}H-NMR (300 MHz, $CDCl_3$): δ 7.40 (d, *J* = 8.2 Hz, 2H), 7.30 (d, *J* = 8.0 Hz, 2H), 4.64 – 4.56 (m, 3H), 4.50 – 4.29 (m, 2H), 3.46 – 3.21 (m, 2H), 2.40 – 2.27 (m, 2H), 1.17 (t, *J* = 7.0 Hz, 3H).

^{13}C-NMR (75 MHz, $CDCl_3$): δ 141.6, 137.5, 129.1, 126.8, 78.1, 72.5, 64.8, 46.0, 35.8, 15.3.

HRMS (ESI) *m/z*, $[M]^+$ calcd for $C_{12}H_{16}NClO_3^+$: 257.0819; found 257.0817.

1-(1-ethoxy-3-nitropropyl)-2,4-dimethylbenzene (6)

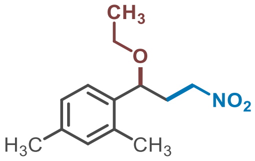

Compound **6** was obtained according to general procedure **GP1** as a colorless oil (82% yield) after purification by column chromatography (SiO_2, hexane/EA=4:1).

^{1}H-NMR (300 MHz, $CDCl_3$): δ 7.19 (dd, *J* = 12.2, 7.8 Hz, 1H), 6.96 (dd, *J* = 7.8, 1.8 Hz, 1H), 6.89 (d, *J* = 1.8 Hz, 1H), 4.64 – 4.52 (m, 1H), 4.48 (dd, *J* = 9.5, 3.6 Hz, 1H), 4.37 (dt, *J* = 13.5, 5.9 Hz, 1H), 3.37 – 3.09 (m, 3H), 2.22 (s, 3H), 2.20 (s, 3H), 1.08 (t, *J* = 7.0 Hz, 3H).

^{13}C-NMR (75 MHz, $CDCl_3$): δ 137.3, 136.2, 135.1, 131.6, 127.2, 125.7, 74.8, 72.6, 64.4, 34.5, 21.1, 18.8, 15.3.

HRMS (ESI) *m/z*, $[M]^+$ calcd for $C_{13}H_{19}NO_3^+$: 237.1365; found 237.1363.

2-(1-ethoxy-3-nitropropyl)naphthalene (7)

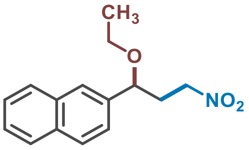

Compound **7** was obtained according to general procedure **GP1** as a light yellow oil (86% yield) after purification by column chromatography (SiO_2, hexane/EA=4:1).

^{1}H-NMR (300 MHz, $CDCl_3$): δ 7.94 – 7.72 (m, 4H), 7.55 – 7.40 (m, 3H), 4.65 (dt, *J* = 13.3, 7.1 Hz, 1H), 4.56 – 4.41 (m, 2H), 3.53 – 3.30 (m, 2H), 2.44 (ddd, *J* = 7.2, 6.3, 5.3 Hz, 2H), 1.20 (t, *J* = 7.0 Hz, 3H).

^{13}C-NMR (75 MHz, $CDCl_3$): δ 138.6, 133.4, 133.3, 128.9, 128.0, 127.9, 126.5, 126.3, 125.7, 123.9, 78.6, 72.6, 64.7, 35.6, 15.3.

HRMS (ESI) *m/z*, $[M+Na]^+$ calcd for $C_{15}H_{17}NO_3+Na^+$: 282.1106; found 282.1104.

(2-ethoxy-4-nitrobutan-2-yl)benzene (8)

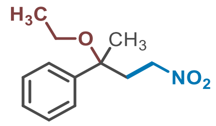

Compound **8** was obtained according to general procedure **GP1** as a light yellow oil (83% yield) after purification by column chromatography (SiO_2, hexane/EA=4:1).

^{1}H NMR (300 MHz, $CDCl_3$): δ 7.42 – 7.32 (m, 5H), 4.47 (ddd, *J* = 13.3, 9.5, 6.1 Hz, 1H), 4.28 (ddd, *J* = 13.2, 9.4, 5.9 Hz, 1H), 3.37 (dq, *J* = 8.8, 6.9 Hz, 1H), 3.21 (dq, *J* = 8.8, 7.0 Hz, 1H), 2.58 – 2.34 (m, 2H), 1.61 (s, 3H), 1.19 (t, *J* = 7.0 Hz, 3H).

^{13}C NMR (75 MHz, $CDCl_3$): δ 144.1, 128.7, 127.5, 125.7, 72.0, 58.1, 40.6, 31.2, 24.1, 15.6.

HRMS (ESI) *m/z*, $[M]^+$ calcd for $C_{12}H_{17}NO_3^+$: 223.1208; found 223.1206.

1-(1-ethoxy-2-methyl-3-nitropropyl)-4-methoxybenzene (9)

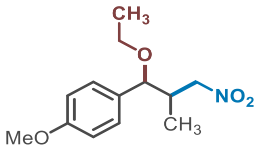

Compound **9** was obtained according to general procedure **GP1** as a light yellow oil (76% yield) after purification by column chromatography (SiO_2, hexane/EA=4:1).

^{1}H NMR (300 MHz, $CDCl_3$): δ 7.19 (d, *J* = 8.5 Hz, 2H), 6.89 (d, *J* = 8.8 Hz, 2H), 4.66 (dd, *J* = 12.1, 5.0 Hz, 1H), 4.38 (dd, *J* = 12.2, 8.2 Hz, 1H), 3.98 (d, *J* = 8.3 Hz, 1H), 3.82 (s, 3H), 3.39 – 3.16 (m, 2H), 2.66 – 2.51 (m, 1H), 1.12 (t, *J* = 7.0 Hz, 3H), 0.83 (d, *J* = 6.9 Hz, 3H).

^{13}C NMR (75 MHz, $CDCl_3$): δ 159.6, 132.0, 128.5, 114.1, 83.5, 79.1, 64.4, 55.4, 39.6, 15.2, 14.5.

HRMS (ESI) *m/z*, $[M+Na]^+$ calcd for $C_{13}H_{19}NO_4+Na^+$: 276.1212; found 276.1210.

5-(1-ethoxy-3-nitropropyl)-4-methylthiazole (10)

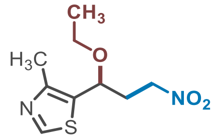

Compound **10** was obtained according to general procedure **GP1** as an orange oil (77% yield) after purification by column chromatography (SiO_2, hexane/EA=1:1).

^{1}H NMR (300 MHz, $CDCl_3$): δ 8.69 (s, 1H), 4.76 – 4.57 (m, 2H), 4.45 (dt, *J* = 13.8, 6.0 Hz, 1H), 3.50 – 3.23 (m, 2H), 2.43 (s, 3H), 2.42 – 2.34 (m, 2H), 1.15 (t, *J* = 7.0 Hz, 3H).

^{13}C NMR (75 MHz, $CDCl_3$): δ 151.7, 150.3, 132.8, 72.0, 71.5, 64.8, 35.6, 15.4, 15.1.

HRMS (ESI) *m/z*, $[M]^+$ calcd for $C_9H_{14}NSO_3^+$: 230.0725; found 230.0722.

1-(tert-butyl)-4-(1-methoxy-3-nitropropyl)benzene (11)

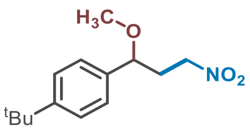

Compound **11** was obtained according to general procedure **GP1** as a light yellow oil (85% yield) after purification by column chromatography (SiO_2, hexane/EA=4:1).

^{1}H-NMR (300 MHz, $CDCl_3$): δ 7.40 (d, *J* = 8.4 Hz, 2H), 7.21 (d, *J* = 8.2 Hz, 2H), 4.58 (dt, *J* = 13.3, 7.2 Hz, 1H), 4.42 (dt, *J* = 13.2, 6.5 Hz, 1H), 4.22 (t, *J* = 6.6 Hz, 1H), 3.22 (s, 3H), 2.41 – 2.31 (m, 2H), 1.33 (s, 9H).

^{13}C-NMR (75 MHz, $CDCl_3$): δ 151.2, 137.2, 126.1, 125.6, 80.0, 72.4, 56.8, 35.5, 34.6, 31.4.

HRMS (ESI) *m/z*, $[M]^+$ calcd for $C_{14}H_{21}NO_3^+$: 251.1521; found 251.1519.

1-(tert-butyl)-4-(1-(methoxy-d3)-3-nitropropyl)benzene (12)

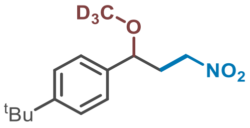

Compound **12** was obtained according to general procedure **GP1** as a light yellow oil (73% yield) after purification by column chromatography (SiO_2, hexane/EA=4:1).

^{1}H-NMR (300 MHz, $CDCl_3$): δ 7.39 (d, *J* = 8.4 Hz, 2H), 7.21 (d, *J* = 8.1 Hz, 2H), 4.64 – 4.36 (m, 1H), 4.21 (t, *J* = 6.6 Hz, 1H), 2.39 – 2.31 (m, 2H), 1.33 (s, 9H).

^{13}C-NMR (75 MHz, $CDCl_3$): δ 151.3, 137.3, 126.2, 125.8, 80.0, 72.6, 35.6, 35.6, 34.7, 31.5.

HRMS (ESI) *m/z*, $[M]^+$ calcd for $C_{14}H_{18}D_3NO_3^+$: 254.1710; found 254.1708.

1-(tert-butyl)-4-(1-isopropoxy-3-nitropropyl)benzene (13)

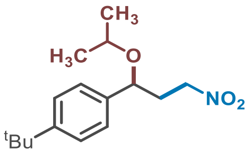

Compound **13** was obtained according to general procedure **GP1** as a light yellow oil (78% yield) after purification by column chromatography (SiO_2, hexane/EA=4:1).

^{1}H NMR (300 MHz, $CDCl_3$): δ 7.38 (d, *J* = 8.4 Hz, 3H), 7.23 (d, *J* = 8.3 Hz, 2H), 4.60 (ddd, *J* = 13.4, 7.7, 6.7 Hz, 1H), 4.49 – 4.35 (m, 2H), 3.50 (p, *J* = 6.1 Hz, 1H), 2.30 (dtd, *J* = 8.4, 6.7, 5.4 Hz, 2H), 1.33 (s, 9H), 1.10 (dd, *J* = 10.6, 6.1 Hz, 6H).

^{13}C NMR (75 MHz, $CDCl_3$): δ 151.0, 138.8, 126.1, 125.6, 75.1, 72.7, 69.1, 36.1, 34.7, 31.5, 23.5, 21.1.

HRMS (ESI) *m/z*, $[M]^+$ calcd for $C_{16}H_{25}NO_3^+$: 279.1834; found 279.1832.

1-(tert-butyl)-4-(1-ethoxy-3-nitrobutyl)benzene (14)

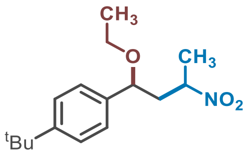

Compound **14** was obtained according to general procedure **GP1** as a light-yellow oil (71% yield) after purification by column chromatography (SiO_2, hexane/EA=4:1).

^{1}H NMR (300 MHz, $CDCl_3$): δ 7.36 (d, *J* = 8.3 Hz, 2H), 7.19 (d, *J* = 8.4 Hz, 2H), 5.00 (ddd, *J* = 10.1, 6.7, 3.2 Hz, 1H), 4.17 (dd, *J* = 10.5, 2.9 Hz, 1H), 3.41 (dq, *J* = 9.3, 7.0 Hz, 1H), 3.27 (dq, *J* = 9.2, 7.0

Hz, 1H), 2.28 (ddd, J = 14.9, 10.3, 2.9 Hz, 1H), 1.99 (ddd, J = 14.9, 10.5, 3.2 Hz, 1H), 1.54 (d, J = 1.7 Hz, 3H), 1.32 (s, 10H), 1.17 (t, J = 7.0 Hz, 3H).

^{13}C NMR (75 MHz, $CDCl_3$): δ 151.0, 138.5, 126.0, 125.6, 80.8, 77.8, 64.7, 44.0, 34.7, 31.5, 20.2, 15.3.

HRMS (ESI) *m/z*, $[M]^+$ calcd for $C_{16}H_{25}NO_3^+$: 279.1834; found 279.1832.

1-(tert-butyl)-4-(1-ethoxy-3-methyl-3-nitrobutyl)benzene (15)

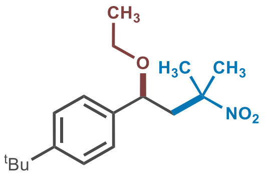

Compound **15** was obtained according to general procedure **GP1** as a light-yellow oil (80% yield) after purification by column chromatography (SiO_2, hexane/EA=4:1).

^{1}H NMR (300 MHz, $CDCl_3$): δ 7.36 (d, J = 8.3 Hz, 2H), 7.21 (d, J = 8.3 Hz, 2H), 4.30 (dd, J = 10.3, 2.5 Hz, 1H), 3.35 (dq, J = 16.2, 6.9 Hz, 1H), 3.20 (dq, J = 9.1, 7.0 Hz, 1H), 2.49 (dd, J = 15.1, 10.3 Hz, 1H), 2.11 – 2.02 (m, 1H), 1.67 (s, 6H), 1.32 (s, 9H), 1.12 (t, J = 7.0 Hz, 3H).

^{13}C NMR (75 MHz, $CDCl_3$): δ 150.8, 139.1, 126.0, 125.6, 87.2, 78.3, 64.4, 48.8, 34.7, 31.5, 27.0, 15.1.

HRMS (ESI) *m/z*, $[M]^+$ calcd for $C_{17}H_{27}NO_3^+$: 293.1991; found 293.1989.

5-(2-(4-(tert-butyl)phenyl)-2-ethoxyethyl)-5-nitro-1,3-dioxane (16)

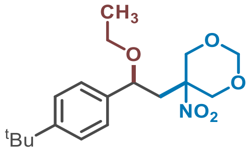

Compound **16** was obtained according to general procedure **GP1** as a yellow oil (83% yield) after purification by column chromatography (SiO_2, hexane/EA=3:1).

^{1}H NMR (300 MHz, $CDCl_3$): δ 7.35 (d, J = 8.4 Hz, 2H), 7.14 (d, J = 8.3 Hz, 2H), 5.07 – 4.96 (m, 2H), 4.72 (d, J = 6.3 Hz, 1H), 4.63 (ddd, J = 12.5, 2.7, 0.9 Hz, 1H), 4.19 (dd, J = 10.5, 2.3 Hz, 1H), 3.95 (d, J = 13.0 Hz, 1H), 3.82 (d, J = 12.5 Hz, 1H), 3.39 – 3.17 (m, 2H), 2.13 (dd, J = 15.1, 10.5 Hz, 1H), 1.93 (dd, J = 15.2, 2.3 Hz, 1H), 1.31 (s, 9H), 1.14 (d, J = 7.0 Hz, 3H).

^{13}C NMR (75 MHz, $CDCl_3$): δ 151.2, 137.9, 125.9, 125.7, 93.9, 86.0, 77.1, 71.2, 71.0, 64.4, 42.5, 34.6, 31.4, 15.2.

HRMS (ESI) *m/z*, $[M+Na]^+$ calcd for $C_{16}H_{21}NO_4$+Na^+: 314.1363; found 314.1360.

3-(2-nitroethyl)isobenzofuran-1(3H)-one (17)

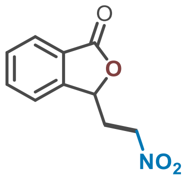

Compound **17** was obtained according to general procedure **GP1** (without adding ethanol) as a colourless oil (86% yield) after purification by column chromatography (SiO_2, hexane/EA=9:1).

^{1}H-NMR (300 MHz, $CDCl_3$): δ 7.93 (dt, J = 7.7, 1.0 Hz, 1H), 7.74 (td, J = 7.5, 1.2 Hz, 1H), 7.64 – 7.56 (m, 1H), 7.51 (dq, J = 7.7, 0.9 Hz, 1H), 5.59 (dd, J = 9.4, 3.1 Hz, 1H), 4.71 (ddd, J = 14.5, 8.2, 6.5 Hz,

1H), 4.54 (ddd, *J* = 14.2, 7.0, 5.4 Hz, 1H), 2.90 (dddd, *J* = 15.1, 8.1, 7.0, 3.1 Hz, 1H), 2.27 (dddd, *J* = 14.9, 9.4, 6.5, 5.4 Hz, 1H).

^{13}C-NMR (75 MHz, $CDCl_3$): δ 169.7, 148.1, 134.7, 130.1, 126.3, 125.9, 122.0, 77.5, 71.1, 32.4.

HRMS (ESI) *m/z*, $[C_{10}H_9O_4N+Na]^+$ calcd: 230.0424; found 230.0419.

3-(2-nitroethyl)isobenzofuran-1(3H)-one (18)

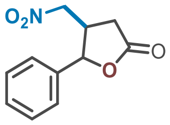

Compound **18** was obtained according to general procedure **GP1** (without adding ethanol) as a yellow oil (82% yield) after purification by column chromatography (SiO_2, hexane/EA=9:1).

^{1}H-NMR (300 MHz, $CDCl_3$): δ 7.42 (tdd, *J* = 3.8, 2.5, 1.0 Hz, 3H), 7.38 – 7.32 (m, 2H), 5.23 (d, *J* = 7.1 Hz, 1H), 4.65 – 4.45 (m, 2H), 3.29 – 3.12 (m, 1H), 2.98 (dd, *J* = 17.7, 8.4 Hz, 1H), 2.57 (dd, *J* = 17.7, 8.4 Hz, 1H).

^{13}C-NMR (75 MHz, $CDCl_3$): δ 173.9, 136.6, 129.7, 129.3, 125.9, 82.5, 75.5, 42.6, 32.8.

HRMS (ESI) *m/z*, $[C_{11}H_{11}O_4N]^+$ calcd: 221.0688; found 221.0686.

2-(4-(tert-butyl)phenyl)-2-(2-nitroethyl)tetrahydrofuran (19)

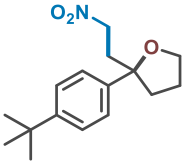

Compound **19** was obtained according to general procedure **GP1** (without adding ethanol) as a light-yellow oil (81% yield) after purification by column chromatography (SiO_2, hexane/EA=9:1).

^{1}H-NMR (300 MHz, $CDCl_3$): δ 7.35 (d, *J* = 8.6 Hz, 2H), 7.25 (d, *J* = 8.5 Hz, 2H), 4.43 (ddd, *J* = 13.4, 8.3, 7.6 Hz, 1H), 4.11 – 3.86 (m, 3H), 2.52 (t, *J* = 7.8 Hz, 2H), 2.26 – 2.05 (m, 2H), 2.04 – 1.75 (m, 2H), 1.32 (s, 9H).

^{13}C-NMR (75 MHz, $CDCl_3$): δ 150.1, 141.6, 125.5, 124.8, 84.6, 72.3, 68.1, 39.6, 39.4, 34.6, 31.5, 25.6.

HRMS (ESI) *m/z*, $[C_{16}H_{23}O_3N]^+$ calcd: 277.1678; found 277.1676.

5-(2-nitroethyl)-5-phenyldihydrofuran-2(3*H*)-one (20)

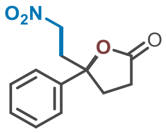

Compound **20** was obtained according to general procedure **GP1** (without adding ethanol) as a yellow oil (78% yield) after purification by column chromatography (SiO_2, hexane/EA=9:1).

^{1}H-NMR (300 MHz, $CDCl_3$): δ 7.45 – 7.28 (m, 5H), 4.45 (ddd, *J* = 13.9, 9.5, 6.2 Hz, 1H), 4.05 (ddd, *J* = 13.9, 9.5, 6.0 Hz, 1H), 2.83 – 2.41 (m, 6H).

^{13}C-NMR (75 MHz, $CDCl_3$): δ 175.5, 140.5, 129.3, 128.6, 124.4, 86.5, 71.0, 39.0, 36.1, 28.1.

HRMS (ESI) *m/z*, $[C_{12}H_{13}O_4N+Na]^+$ calcd: 258.0742; found 258.0739.

5-(nitromethyl)-6-phenyltetrahydro-2*H*-pyran-2-one (21)

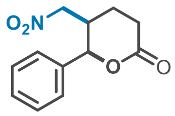

Compound **21** was obtained according to general procedure **GP1** (without adding ethanol) as a yellow oil (72% yield) after purification by column chromatography (SiO_2, hexane/EA=9:1).

^{1}H-NMR (300 MHz, $CDCl_3$): δ 7.47 – 7.28 (m, 5H), 5.20 (d, *J* = 10.0 Hz, 1H), 4.28 – 4.15 (m, 2H), 2.89 – 2.62 (m, 3H), 2.26 – 2.11 (m, 1H), 2.06 – 1.86 (m, 1H).

^{13}C-NMR (75 MHz, $CDCl_3$): δ 170.0, 136.3, 129.9, 129.4, 127.1, 82.4, 76.3, 38.7, 28.6, 22.6.

HRMS (ESI) *m/z*, $[C_{12}H_{14}O_4N]^+$ calcd: 236.0917; found 236.0915.

2-(2-nitroethyl)-2-phenylcyclopentan-1-one (22)

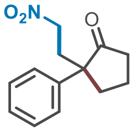

Compound **22** was obtained according to general procedure **GP1** (without adding ethanol) as a yellow oil (79% yield) after purification by column chromatography (SiO_2, hexane/EA=9:1).

^{1}H-NMR (300 MHz, $CDCl_3$): δ 7.42 – 7.25 (m, 5H), 4.35 (ddd, *J* = 13.4, 9.8, 5.6 Hz, 1H), 4.07 (ddd, *J* = 13.5, 9.8, 6.1 Hz, 1H), 2.61 (ddd, *J* = 14.0, 9.8, 5.5 Hz, 2H), 2.44 – 2.20 (m, 3H), 2.04 – 1.91 (m, 2H), 1.86 – 1.70 (m, 1H).

^{13}C-NMR (75 MHz, $CDCl_3$): δ 218.3, 137.1, 129.4, 127.9, 126.7, 72.2, 55.1, 37.08, 35.7, 35.3, 18.7.

HRMS (ESI) *m/z*, $[C_{13}H_{14}O_3N]^+$ calcd: 232.0968; found 232.0971.

5-(4-(tert-butyl)phenyl)-3-nitrotetrahydrofuran-3-yl)methanol (SP-15-184)

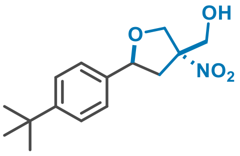

Compound **23** was obtained according to general procedure **GP2** (81% yield) after purification by column chromatography (SiO_2, hexane/EA=2:1).

23a: light-yellow oil.

^{1}H-NMR (300 MHz, $CDCl_3$): δ 7.40 (d, *J* = 8.5 Hz, 2H), 7.27 (d, *J* = 8.3 Hz, 2H), 5.18 (dd, *J* = 9.7, 6.2 Hz, 1H), 4.35 (q, *J* = 10.7 Hz, 2H), 4.06 (q, *J* = 12.3 Hz, 2H), 3.05 (dd, *J* = 14.3, 6.1 Hz, 1H), 2.65 (s, 1H), 2.12 (dd, *J* = 14.3, 9.7 Hz, 1H), 1.32 (s, 9H).

^{13}C-NMR (75 MHz, $CDCl_3$): δ 151.4, 136.6, 125.7, 125.7, 97.8, 81.2, 73.7, 66.1, 42.7, 34.7, 31.4.

HRMS (ESI) *m/z*, $[C_{15}H_{21}NO_4+Na]^+$ calcd for: 302.1363; found 302.1361.

23a: light-yellow solid.

^{1}H NMR (300 MHz, $CDCl_3$): δ 7.40 (d, *J* = 8.5 Hz, 2H), 7.31 (d, *J* = 8.4 Hz, 2H), 4.89 (dd, *J* = 9.1, 6.7 Hz, 1H), 4.71 (d, *J* = 11.0 Hz, 1H), 4.25 – 3.92 (m, 4H), 2.98 (s, 1H), 2.82 – 2.58 (m, 2H), 1.32 (s, 9H).

^{13}C NMR (75 MHz, $CDCl_3$): δ 151.6, 136.3, 126.0, 125.7, 98.3, 81.3, 73.8, 65.6, 42.0, 34.7, 31.4.

3-nitro-5-(p-tolyl)tetrahydrofuran-3-yl)methanol (24)

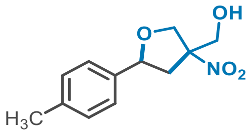

Compound **24** was obtained according to general procedure **GP2** as a light-yellow solid (83% yield) after purification by column chromatography (SiO_2, hexane/EA=2:1).

24a: light-yellow solid.

^{1}H NMR (300 MHz, $CDCl_3$): δ 7.21 (q, *J* = 8.2 Hz, 4H), 5.17 (dd, *J* = 9.7, 6.1 Hz, 1H), 4.42 – 4.29 (m, 2H), 4.14 – 3.97 (m, 2H), 3.04 (dd, *J* = 14.3, 6.2 Hz, 1H), 2.63 (s, 1H), 2.35 (s, 3H), 2.10 (dd, *J* = 14.3, 9.7 Hz, 1H).

^{13}C NMR (75 MHz, $CDCl_3$): δ 138.2, 136.6, 129.5, 125.8, 97.8, 81.3, 73.7, 66.1, 42.9, 21.3.

HRMS (ESI) *m/z*, $[M+Na]^+$ calcd for $C_{12}H_{15}NO_4$+Na^+: 260.0893; found 260.0894.

24b: light-yellow solid.

^{1}H NMR (300 MHz, $CDCl_3$): δ 7.27 (d, *J* = 8.0 Hz, 2H), 7.21 – 7.14 (d, *J* = 8.0 Hz, 2H), 4.88 (dd, *J* = 9.1, 6.8 Hz, 1H), 4.71 (d, *J* = 11.0 Hz, 1H), 4.25 – 3.93 (m, 3H), 2.80 – 2.57 (m, 3H), 2.35 (s, 3H).

^{13}C NMR (75 MHz, $CDCl_3$): δ 138.3, 136.4, 129.5, 126.2, 98.2, 81.4, 73.9, 65.6, 42.3, 21.3.

4-(4-(hydroxymethyl)-4-nitrotetrahydrofuran-2-yl)phenyl acetate (25)

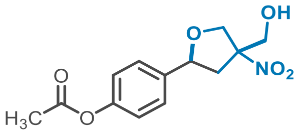

Compound **25** was obtained according to general procedure **GP2** as a light-yellow oil (86% yield) after purification by column chromatography (SiO_2, hexane/EA=2:1).

25a: light-yellow solid.

^{1}H-NMR (300 MHz, $CDCl_3$): δ 7.33 (d, *J* = 0.6 Hz, 2H), 7.08 (d, *J* = 8.7 Hz, 2H), 5.17 (dd, *J* = 9.8, 6.1 Hz, 1H), 4.41 – 4.26 (m, 2H), 4.11 – 3.92 (m, 2H), 3.06 (dd, *J* = 14.3, 6.1 Hz, 1H), 2.79 (s, 1H), 2.29 (s, 3H), 2.05 (dd, *J* = 10.0, 4.3 Hz, 1H).

^{13}C-NMR (75 MHz, $CDCl_3$): δ 169.9, 150.5, 137.5, 126.9, 121.9, 97.8, 80.7, 73.7, 66.1, 65.9, 42.8, 21.2.
HRMS (ESI) *m/z*, $[C_{13}H_{15}NO_6+Na]^+$ calcd for: 304.0797; found 304.0795.

25b: yellow solid.

^{1}H-NMR (300 MHz, $CDCl_3$): δ 7.39 (d, *J* = 8.5 Hz, 2H), 7.09 (d, *J* = 8.6 Hz, 2H), 4.90 (t, *J* = 8.0 Hz, 1H), 4.70 (d, *J* = 10.9 Hz, 1H), 4.25 – 3.91 (m, 3H), 2.79 – 2.50 (m, 3H), 2.30 (s, 3H).

^{13}C-NMR (75 MHz, $CDCl_3$): δ 169.8, 150.6, 137.2, 127.4, 121.9, 98.2, 80.8, 73.9, 65.4, 42.1, 21.2.

(5-(4-bromophenyl)-3-nitrotetrahydrofuran-3-yl)methanol (26)

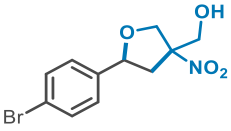

Compound **26** was obtained according to general procedure **GP2** (78% yield) after purification by column chromatography (SiO_2, hexane/EA=2:1).

26a: light-yellow oil.

^{1}H-NMR (300 MHz, $CDCl_3$): δ 7.49 (d, *J* = 8.5 Hz, 2H), 7.21 (d, *J* = 8.7 Hz, 2H), 5.15 (dd, *J* = 9.8, 6.2 Hz, 1H), 4.43 – 4.26 (m, 2H), 4.11 (d, *J* = 12.4 Hz, 1H), 4.01 (dd, *J* = 12.4, 0.9 Hz, 1H), 3.07 (dd, *J* = 14.3, 6.2 Hz, 1H), 2.63 (s, 1H), 2.04 (dd, *J* = 14.3, 9.7 Hz, 1H).

^{13}C-NMR (75 MHz, $CDCl_3$): δ 138.8, 131.9, 127.5, 122.2, 97.7, 80.6, 73.8, 66.0, 42.9.i

26b: light-yellow solid.

^{1}H NMR (300 MHz, $CDCl_3$): δ 7.42 (d, *J* = 8.5 Hz, 2H), 7.17 (d, *J* = 8.3 Hz, 2H), 4.81 (t, *J* = 7.9 Hz, 1H), 4.63 (d, *J* = 11.0 Hz, 1H), 4.34 (d, *J* = 12.7 Hz, 1H), 4.18 (d, *J* = 12.7 Hz, 1H), 3.91 (d, *J* = 11.0 Hz, 1H), 2.61 (d, *J* = 7.9 Hz, 2H).

^{13}C NMR (75 MHz, $CDCl_3$): δ 138.6, 131.9, 127.8, 122.3, 98.2, 80.7, 74.0, 66.3, 65.5, 42.1.

HRMS (ESI) *m/z*, $[C_{11}H_{12}NBrO_4+Na]^+$ calcd for: 323.9847; found 323.9844.

5-methyl-3-nitro-5-phenyltetrahydrofuran-3-yl)methanol (27)

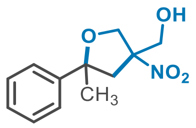

Compound **27** was obtained according to general procedure **GP2** (72% yield) after purification by column chromatography (SiO_2, hexane/EA=2:1).

27a: light-yellow oil.

^{1}H-NMR (300 MHz, $CDCl_3$): δ 7.38 (d, *J* = 4.0 Hz, 4H), 7.34 – 7.27 (m, 1H), 4.63 (d, *J* = 10.9 Hz, 1H), 3.97 (d, *J* = 11.0 Hz, 1H), 3.86 – 3.70 (m, 2H), 3.03 (d, *J* = 14.3 Hz, 1H), 2.72 (d, *J* = 14.4 Hz, 1H), 2.63 (s, 1H), 1.63 (s, 3H).

^{13}C-NMR (75 MHz, $CDCl_3$): δ 145.7, 128.8, 127.3, 124.4, 99.2, 85.8, 72.1, 65.8, 46.4, 30.6.

27b: light-yellow solid.

^{1}H NMR (300 MHz, $CDCl_3$): δ 7.44 – 7.33 (m, 4H), 7.31 – 7.22 (m, 1H), 4.47 (d, *J* = 10.7 Hz, 1H), 4.22 (d, *J* = 10.6 Hz, 1H), 4.08 (s, 2H), 3.29 (d, *J* = 14.5 Hz, 1H), 2.84 (s, 1H), 2.41 (d, *J* = 14.5 Hz, 1H), 1.58 (s, 3H).

^{13}C NMR (75 MHz, $CDCl_3$): δ 145.1, 128.6, 127.3, 124.6, 98.2, 85.6, 71.0, 67.0, 46.0, 30.6.

HRMS (ESI) *m/z*, $[C_{12}H_{15}NO_4+Na]^+$ calcd for: 260.0899; found 260.0897.

(3-nitro-3,3a,4,8b-tetrahydro-2H-indeno[1,2-b]furan-3-yl)methanol (28)

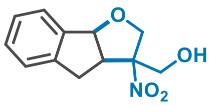

Compound **28** was obtained according to general procedure **GP2** (76% yield) after purification by column chromatography (SiO_2, hexane/EA=2:1).

28a: light-yellow oil.

^{1}H-NMR (300 MHz, $CDCl_3$): δ 7.49 – 7.40 (m, 1H), 7.37 – 7.18 (m, 4H), 5.78 (d, *J* = 6.9 Hz, 1H), 4.51 (dd, *J* = 10.9, 1.1 Hz, 1H), 4.10 (s, 2H), 3.99 – 3.87 (m, 1H), 3.64 (d, *J* = 10.9 Hz, 1H), 3.27 (dd, *J* = 17.4, 9.5 Hz, 1H), 3.11 (dd, *J* = 17.5, 4.3 Hz, 1H), 2.19 (s, 1H).

^{13}C-NMR (75 MHz, $CDCl_3$): δ 142.0, 139.9, 129.7, 127.8, 125.8, 124.6, 101.4, 88.4, 72.7, 63.3, 48.5, 32.4.

28b: light-yellow solid.

^{1}H NMR (300 MHz, $CDCl_3$): δ 7.47 – 7.39 (m, 1H), 7.37 – 7.27 (m, 2H), 7.24 – 7.16 (m, 1H), 5.58 (d, *J* = 6.2 Hz, 1H), 4.34 – 4.22 (m, 1H), 4.19 (d, *J* = 9.8 Hz, 1H), 4.10 – 3.93 (m, 2H), 3.27 – 3.11 (m, 2H), 2.92 (s, 1H), 2.79 – 2.65 (m, 1H).

^{13}C NMR (75 MHz, $CDCl_3$): δ 142.1, 139.5, 129.8, 127.8, 125.7, 124.6, 98.5, 87.3, 67.1, 65.6, 45.9, 34.0.

HRMS (ESI) *m/z*, $[C_{12}H_{13}NO_4+Na]^+$ calcd for: 258.0742; found 258.0740.

(3-nitro-2,3,3a,4,5,9b-hexahydronaphtho[1,2-b]furan-3-yl)methanol (29)

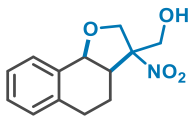

Compound **29** was obtained according to general procedure **GP2** (79% yield) after purification by column chromatography (SiO_2, hexane/EA=2:1).

29a: light-yellow oil.

^{1}H-NMR (300 MHz, $CDCl_3$): δ 7.44 – 7.34 (m, 1H), 7.30 – 7.20 (m, 2H), 7.15 (dd, *J* = 5.5, 3.7 Hz, 1H), 5.03 (d, *J* = 5.0 Hz, 1H), 4.52 (d, *J* = 11.0 Hz, 1H), 4.19 – 4.00 (m, 3H), 3.16 (dt, *J* = 13.2, 4.7 Hz, 1H), 2.92 – 2.58 (m, 3H), 2.14 – 1.98 (m, 1H), 1.53 (qd, *J* = 12.7, 4.4 Hz, 1H).

^{13}C-NMR (75 MHz, $CDCl_3$): δ 136.7, 132.7, 130.3, 128.5, 126.8, 102.3, 77.9, 72.0, 63.0, 45.1, 29.0, 21.1.

29b: light-yellow solid.

^{1}H NMR (300 MHz, $CDCl_3$): δ 7.42 (dd, *J* = 5.5, 3.7 Hz, 1H), 7.30 – 7.20 (m, 2H), 7.14 (dd, *J* = 5.5, 3.7 Hz, 1H), 4.87 – 4.74 (m, 2H), 4.56 – 4.44 (m, 1H), 4.08 (dd, *J* = 20.9, 11.6 Hz, 2H), 2.84 (dt, *J* = 16.4, 3.9 Hz, 1H), 2.74 – 2.55 (m, 2H), 2.46 (s, 1H), 1.78 – 1.45 (m, 3H).

^{13}C NMR (75 MHz, $CDCl_3$): δ 137.0, 132.6, 130.0, 128.5, 128.5, 126.8, 101.0, 77.4, 70.9, 66.4, 44.5, 28.7, 21.2.

HRMS (ESI) *m/z*, $[C_{13}H_{15}NO_4+Na]^+$ calcd for: 272.0899; found 272.0897.

3-nitro-1-phenylpropan-1-one (30)[5]

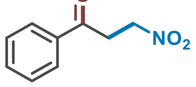

Compound **30** was obtained according to general procedure **GP3** as a colorless oil (65% yield) after purification by column chromatography (SiO_2, hexane/EA=9:1).

^{1}H-NMR (300 MHz, $CDCl_3$): δ 8.03 – 7.93 (m, 2H), 7.67 – 7.57 (m, 1H), 7.51 (dd, *J* = 8.3, 6.8 Hz, 2H), 4.83 (t, *J* = 6.1 Hz, 2H), 3.66 (t, *J* = 6.1 Hz, 2H).

^{13}**C-NMR** (75 MHz, $CDCl_3$): δ 195.1, 135.8, 134.1, 129.0, 128.3, 69.4, 35.0.

3-nitro-1-(p-tolyl)propan-1-one (31)[6]

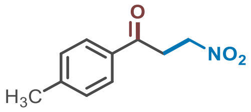

Compound **31** was obtained according to general procedure **GP3** as a colorless oil (74% yield) after purification by column chromatography (SiO_2, hexane/EA=9:1).

^{1}**H-NMR** (300 MHz, $CDCl_3$): δ 7.87 (d, *J* = 8.2 Hz, 2H), 7.29 (d, *J* = 7.9 Hz, 2H), 4.81 (t, *J* = 6.1 Hz, 2H), 3.63 (t, *J* = 6.2 Hz, 2H), 2.43 (s, 3H).

^{13}**C-NMR** (75 MHz, $CDCl_3$): δ 195.1, 135.8, 134.1, 129.0, 128.8, 69.4, 35.0.

1-(4-(tert-butyl)phenyl)-3-nitropropan-1-one (32)[6]

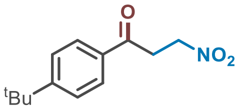

Compound **32** was obtained according to general procedure **GP3** as a colorless oil (74% yield) after purification by column chromatography (SiO_2, hexane/EA=9:1).

^{1}**H-NMR** (300 MHz, $CDCl_3$): δ 7.93 (d, *J* = 8.6 Hz, 2H), 7.52 (d, *J* = 8.6 Hz, 2H), 4.83 (t, *J* = 6.2 Hz, 2H), 3.65 (t, *J* = 6.2 Hz, 2H), 1.35 (s, 9H).

1-(4-methoxyphenyl)-3-nitropropan-1-one (33)[6]

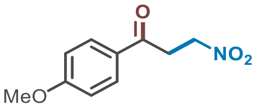

Compound **33** was obtained according to general procedure **GP3** as a colorless oil (70% yield) after purification by column chromatography (SiO_2, hexane/EA=9:1).

^{1}**H-NMR** (300 MHz, $CDCl_3$): δ 7.56 (d, *J* = 9.0 Hz, 2H), 6.93 (d, *J* = 8.9 Hz, 3H), 4.70 – 4.59 (m, 2H), 3.84 (s, 3H), 3.53 – 3.44 (m, 2H).

1-(4-(tert-butoxy)phenyl)-3-nitropropan-1-one (34)

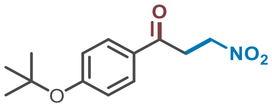

Compound **34** was obtained according to general procedure **GP3** as a colorless oil (68% yield) after purification by column chromatography (SiO_2, hexane/EA=9:1).

^{1}**H-NMR** (300 MHz, $CDCl_3$): δ 7.92 (d, *J* = 8.8 Hz, 2H), 7.06 (d, *J* = 8.8 Hz, 2H), 4.82 (t, *J* = 6.2 Hz, 2H), 3.62 (t, *J* = 6.2 Hz, 2H), 1.44 (s, 9H).

^{13}**C-NMR** (75 MHz, $CDCl_3$): δ 193.8, 161.4, 130.2, 129.9, 122.4, 80.1, 69.6, 34.8, 29.1.

HRMS (ESI) *m/z*, $[M+Na]^+$ calcd for $C_{13}H_{17}NO_4+Na^+$: 274.1050; found 274.1048.

1-(4-fluorophenyl)-3-nitropropan-1-one (35)[6]

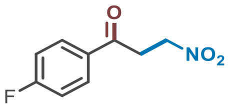

Compound **35** was obtained according to general procedure **GP3** as a colorless oil (69% yield) after purification by column chromatography (SiO_2, hexane/EA=9:1).

^{1}H-NMR (300 MHz, $CDCl_3$): δ 8.07 – 7.96 (m, 2H), 7.22 – 7.14 (m, 2H), 4.83 (t, *J* = 6.1 Hz, 2H), 3.63 (t, *J* = 6.1 Hz, 2H).

1-(4-(methylthio)phenyl)-3-nitropropan-1-one (36)

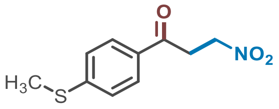

Compound **36** was obtained according to general procedure **GP3** as a colorless oil (51% yield) after purification by column chromatography (SiO_2, hexane/EA=9:1).

^{1}H-NMR (300 MHz, $CDCl_3$): δ 7.88 (d, *J* = 8.6 Hz, 2H), 7.29 (d, *J* = 8.6 Hz, 2H), 4.82 (t, *J* = 6.2 Hz, 2H), 3.62 (t, *J* = 6.2 Hz, 2H), 2.53 (s, 4H).

^{13}C-NMR (75 MHz, $CDCl_3$): δ 194.0, 147.4, 132.1, 128.6, 125.2, 69.5, 34.8, 14.8.

HRMS (ESI) *m/z*, $[M+Na]^+$ calcd for $C_{10}H_{11}SNO_3+Na^+$: 248.0352; found 248.0351.

3-nitro-1-(o-tolyl)propan-1-one (37)[6]

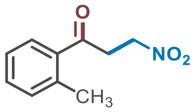

Compound **37** was obtained according to general procedure **GP3** as a colorless oil (66% yield) after purification by column chromatography (SiO_2, hexane/EA=9:1).

^{1}H-NMR (300 MHz, $CDCl_3$): δ 7.74 (dd, *J* = 7.7, 1.4 Hz, 1H), 7.44 (td, *J* = 7.5, 1.5 Hz, 1H), 7.35 – 7.27 (m, 2H), 4.81 (t, *J* = 6.0 Hz, 2H), 3.58 (t, *J* = 6.0 Hz, 2H), 2.52 (s, 3H).

^{13}C-NMR (75 MHz, $CDCl_3$): δ 198.2, 139.4, 136.1, 132.5, 132.4, 129.0, 126.1, 69.6, 37.3, 21.7.

1-(3-fluorophenyl)-3-nitropropan-1-one (38)[6]

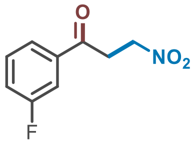

Compound **38** was obtained according to general procedure **GP3** as a colorless oil (62% yield) after purification by column chromatography (SiO_2, hexane/EA=9:1).

^{1}H-NMR (300 MHz, $CDCl_3$): δ 7.77 (dt, *J* = 7.7, 1.3 Hz, 1H), 7.67 (dt, *J* = 9.2, 2.1 Hz, 1H), 7.50 (td, *J* = 8.0, 5.5 Hz, 1H), 7.39 – 7.30 (m, 1H), 4.84 (t, *J* = 6.1 Hz, 2H), 3.64 (t, *J* = 6.1 Hz, 2H).

^{19}F-NMR (300 MHz, $CDCl_3$): δ -111.08.

^{13}C-NMR (75 MHz, $CDCl_3$): δ 193.8, 162.9 (d, *J* = 249.1 Hz), 137.8, 130.64 (d, *J* = 7.5 Hz), 123.93 (d, *J* = 3.0 Hz), 121.1 (d, *J* = 21.6 Hz), 115.0 (d, *J* = 22.4 Hz), 69.1, 35.0.

3-nitro-1-(3-nitrophenyl)propan-1-one (39)

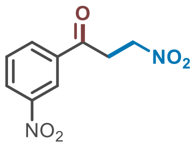

Compound **39** was obtained according to general procedure **GP3** as a colorless oil (58% yield) after purification by column chromatography (SiO_2, hexane/EA=9:1).

^{1}H-NMR (300 MHz, $CDCl_3$): δ 8.52 (t, *J* = 2.1 Hz, 1H), 8.27 (ddd, *J* = 8.3, 2.3, 1.1 Hz, 1H), 8.01 – 7.93 (m, 1H), 7.61 (t, *J* = 8.0 Hz, 1H), 4.74 (t, *J* = 7.3 Hz, 2H), 3.52 (t, *J* = 7.3 Hz, 2H).

^{13}C-NMR (75 MHz, $CDCl_3$): δ 152.8, 148.7, 136.4, 132.0, 130.0, 124.5, 121.4, 71.0, 24.6.

1-(4-(tert-butyl)phenyl)-3-nitrobutan-1-one (40)

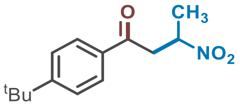

Compound **40** was obtained according to general procedure **GP3** as a colorless oil (60% yield) after purification by column chromatography (SiO_2, hexane/EA=9:1).

^{1}H-NMR (300 MHz, $CDCl_3$): δ 7.90 (d, *J* = 8.6 Hz, 2H), 7.50 (d, *J* = 8.6 Hz, 2H), 5.26 – 5.12 (m, 1H), 3.89 (dd, *J* = 18.0, 7.7 Hz, 1H), 3.26 (dd, *J* = 18.0, 5.3 Hz, 1H), 1.68 (d, *J* = 6.9 Hz, 3H), 1.35 (s, 9H).

^{13}C-NMR (75 MHz, $CDCl_3$): δ 194.8, 157.9, 133.5, 128.2, 125.9, 78.4, 42.5, 35.4, 31.2, 19.8.

HRMS (ESI) *m/z*, $[M]^+$ calcd for $C_{13}H_{16}NO_3^+$: 234.1125; found 234.1128.

1-(4-(tert-butyl)phenyl)-3-methyl-3-nitrobutan-1-one (41)

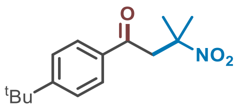

Compound **41** was obtained according to general procedure **GP3** as a colorless oil (68% yield) after purification by column chromatography (SiO_2, hexane/EA=9:1).

^{1}H-NMR (300 MHz, $CDCl_3$): δ 7.87 (d, *J* = 8.6 Hz, 2H), 7.48 (d, *J* = 8.6 Hz, 2H), 3.66 (s, 2H), 1.75 (s, 6H), 1.34 (s, 9H).

^{13}C-NMR (75 MHz, $CDCl_3$): δ 194.7, 157.6, 134.0, 128.0, 125.8, 85.1, 47.1, 35.3, 31.4, 31.1, 26.7.

HRMS (ESI) *m/z*, $[M]^+$ calcd for $C_{15}H_{21}NO_3^+$: 263.1521; found 263.1519.

1-(4-(tert-butyl)phenyl)-2-(5-nitro-1,3-dioxan-5-yl)ethan-1-one (42)

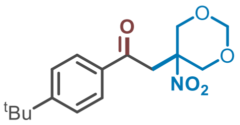

Compound **42** was obtained according to general procedure **GP3** as a colorless oil (72% yield) after purification by column chromatography (SiO_2, hexane/EA=9:1).

^{1}H-NMR (300 MHz, $CDCl_3$): δ 7.88 (d, *J* = 8.6 Hz, 2H), 7.50 (d, *J* = 8.7 Hz, 2H), 4.95 (d, *J* = 6.1 Hz, 1H), 4.82 (d, *J* = 6.1 Hz, 1H), 4.45 (d, *J* = 11.7 Hz, 2H), 4.30 (d, *J* = 11.7 Hz, 2H), 3.84 (s, 2H), 1.34 (s, 9H).

^{13}C-NMR (75 MHz, $CDCl_3$): δ 194.2, 158.2, 133.5, 128.2, 126.0, 94.4, 82.0, 71.1, 40.8, 35.4, 31.2.

HRMS (ESI) *m/z*, $[M+Na]^+$ calcd for $C_{16}H_{21}NO_5+Na^+$: 330.1312; found 330.1310.

1-(4-(tert-butoxy)phenyl)-3-nitropropan-1-ol (43)

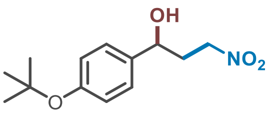

Compound **43** was obtained according to general procedure **GP4** as a colorless oil (49% yield) after purification by column chromatography (SiO_2, hexane/EA=3:1).

^{1}H-NMR (300 MHz, $CDCl_3$): δ 7.23 (d, *J* = 8.5 Hz, 2H), 6.97 (d, *J* = 8.5 Hz, 2H), 4.79 (dd, *J* = 7.5, 5.6 Hz, 1H), 4.60 (dt, *J* = 14.0, 7.1 Hz, 1H), 4.46 (dt, *J* = 13.3, 6.5 Hz, 1H), 2.44 – 2.33 (m, 2H), 1.34 (s, 9H).

^{13}C-NMR (75 MHz, $CDCl_3$): δ 155.5, 137.8, 126.4, 124.5, 78.9, 72.6, 71.0, 36.1, 29.0.

HRMS (ESI) *m/z*, $[C_{13}H_{19}O_4N+Na]^+$ calcd: 276.1206; found 276.1204.

1-(4-methoxyphenyl)-3-nitropropan-1-ol (44)[6]

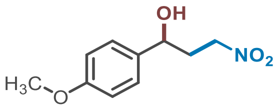

Compound **44** was obtained according to general procedure **GP4** as a colorless oil (53% yield) after purification by column chromatography (SiO_2, hexane/EA=3:1).

^{1}H-NMR (300 MHz, $CDCl_3$): δ 7.27 (d, *J* = 8.5 Hz, 2H), 6.90 (d, *J* = 8.5 Hz, 2H), 4.80 (t, *J* = 6.2 Hz, 1H), 4.58 (dt, *J* = 13.4, 7.2 Hz, 1H), 4.44 (dt, *J* = 13.4, 6.6 Hz, 1H), 3.80 (s, 3H), 3.61 (t, *J* = 6.2 Hz, 1H), 2.38 (q, *J* = 6.6 Hz, 2H), 2.08 (s, 1H).

3-nitro-1-(4-(4,4,5,5-tetramethyl-1,3,2-dioxaborolan-2-yl)phenyl)propan-1-ol (45)

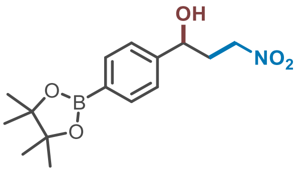

Compound **45** was obtained according to general procedure **GP4** as a colorless oil (51% yield) after purification by column chromatography (SiO_2, hexane/EA=3:1).

^{1}H-NMR (300 MHz, $CDCl_3$): δ 7.81 (d, *J* = 8.1 Hz, 2H), 7.35 (d, *J* = 7.9 Hz, 2H), 4.85 (dd, *J* = 8.4, 4.6 Hz, 1H), 4.61 (ddd, *J* = 13.6, 7.6, 6.7 Hz, 1H), 4.44 (dt, *J* = 13.3, 6.4 Hz, 1H), 2.47 – 2.29 (m, 2H), 1.34 (s, 12H).

^{13}C-NMR (75 MHz, $CDCl_3$): δ 146.1, 135.4, 125.0, 84.1, 72.4, 71.2, 36.1, 25.0.

HRMS (ESI) *m/z*, $[C_{15}H_{22}O_5NB+Na]^+$ calcd: 330.1483; found 330.1481.

1-(4-(tert-butyl)phenyl)-3-nitropropyl acetate (46)

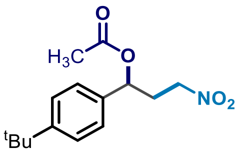

Compound **46** was obtained according to general procedure **GP5** as a yellow oil (84% yield) after purification by column chromatography (SiO_2, hexane/EA=8:1).

^{1}H-NMR (300 MHz, $CDCl_3$): δ 7.39 (d, *J* = 8.4 Hz, 2H), 7.25 (d, *J* = 8.3 Hz, 3H), 5.85 (dd, *J* = 7.8, 5.4 Hz, 1H), 4.50 – 4.29 (m, 2H), 2.66 – 2.46 (m, 2H), 2.08 (s, 3H), 1.31 (s, 9H).

^{13}C-NMR (75 MHz, $CDCl_3$): δ 170.1, 151.8, 135.7, 126.1, 125.9, 72.7, 72.1, 34.8, 33.7, 31.4, 21.2.

HRMS (ESI) *m/z*, $[M–CH_3]^+$ calcd for $C_{14}H_{18}NO_4$: 264.1230; found 264.1235.

1-(4-methoxyphenyl)-3-nitropropyl acetate (47)

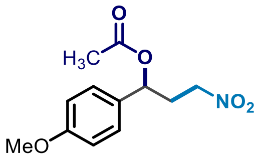

Compound **47** was obtained according to general procedure **GP5** as a yellow oil (87% yield) after purification by column chromatography (SiO_2, hexane/EA=8:1).

^{1}H-NMR (300 MHz, $CDCl_3$): δ 7.26 (d, *J* = 8.6 Hz, 2H), 6.89 (d, *J* = 8.7 Hz, 2H), 5.81 (dd, *J* = 8.0, 5.5 Hz, 1H), 4.48 – 4.28 (m, 2H), 3.80 (s, 3H), 2.61 (ddt, *J* = 14.8, 8.1, 6.9 Hz, 1H), 2.48 (ddd, *J* = 14.4, 7.1, 1.6 Hz, 1H), 2.06 (s, 3H).

^{13}C-NMR (75 MHz, $CDCl_3$): δ 170.1, 159.9, 130.8, 127.8, 114.3, 72.6, 72.1, 55.4, 33.6, 21.1.

HRMS (ESI) *m/z*, $[M+Na]^+$ calcd for $C_{12}H_{15}NO_5+Na^+$: 276.0842; found 276.0841.

3-methyl-1-(naphthalen-2-yl)-3-nitrobutyl acetate (48)

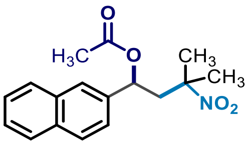

Compound **48** was obtained according to general procedure **GP5** as a yellow oil (88% yield) after purification by column chromatography (SiO_2, hexane/EA=8:1).

^{1}H-NMR (300 MHz, $CDCl_3$): δ 7.88 – 7.78 (m, 4H), 7.52 – 7.42 (m, 3H), 6.08 (dd, *J* = 10.7, 3.1 Hz, 1H), 2.96 (dd, *J* = 15.4, 10.7 Hz, 1H), 2.24 (dd, *J* = 15.4, 3.1 Hz, 1H), 2.04 (s, 3H), 1.70 (s, 3H), 1.64 (s, 3H).

^{13}C-NMR (75 MHz, $CDCl_3$): δ 170.0, 137.3, 133.2, 133.2, 128.8, 128.1, 127.8, 126.5, 126.4, 125.6, 123.8, 86.2, 72.1, 46.5, 27.9, 24.5, 20.9.

HRMS (ESI) *m/z*, $[M]^+$ calcd for $C_{17}H_{19}NO_4$: 301.1309; found 301.1312.

1-(4-methoxyphenyl)-2-(5-nitro-1,3-dioxan-5-yl)ethyl acetate (49)

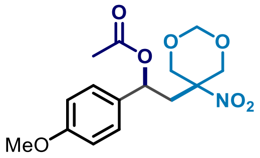

Compound **49** was obtained according to general procedure **GP5** as a yellow oil (85% yield) after purification by column chromatography (SiO_2, hexane/EA=8:1).

^{1}H-NMR (300 MHz, $CDCl_3$): δ 7.21 (d, *J* = 8.7 Hz, 2H), 6.85 (d, *J* = 8.7 Hz, 2H), 5.73 (dd, *J* = 10.3, 3.5 Hz, 1H), 4.88 (d, *J* = 6.3 Hz, 1H), 4.73 (d, *J* = 6.2 Hz, 1H), 4.54 (d, *J* = 12.4 Hz, 2H), 3.94 – 3.83 (m, 2H), 3.77 (s, 3H), 2.55 (dd, *J* = 15.5, 10.3 Hz, 1H), 2.20 (dd, *J* = 15.4, 3.5 Hz, 1H), 1.97 (s, 3H).

^{13}C-NMR (75 MHz, $CDCl_3$): δ 169.7, 159.9, 131.2, 127.7, 114.2, 93.9, 83.5, 71.6, 70.3, 69.5, 55.3, 39.6, 20.8.

HRMS (ESI) *m/z*, $[M]^+$ calcd for $C_{15}H_{19}NO_7^+$: 325.1156; found 325.1161.

13. NMR Spectra of Isolated Compound

^{1}H NMR (300 MHz, $CDCl_3$) of **2**

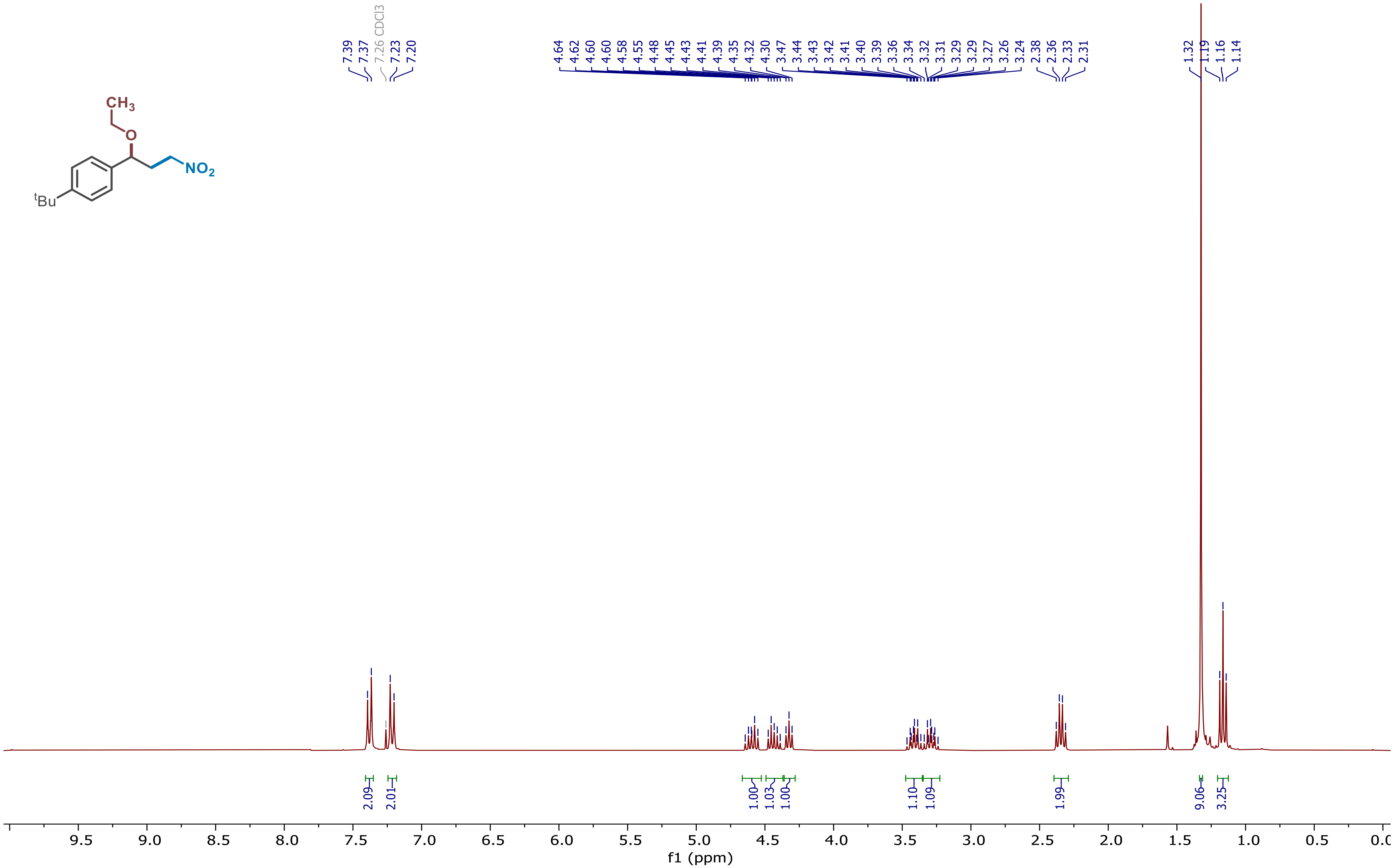

^{13}C NMR (75 MHz, $CDCl_3$) of **2**

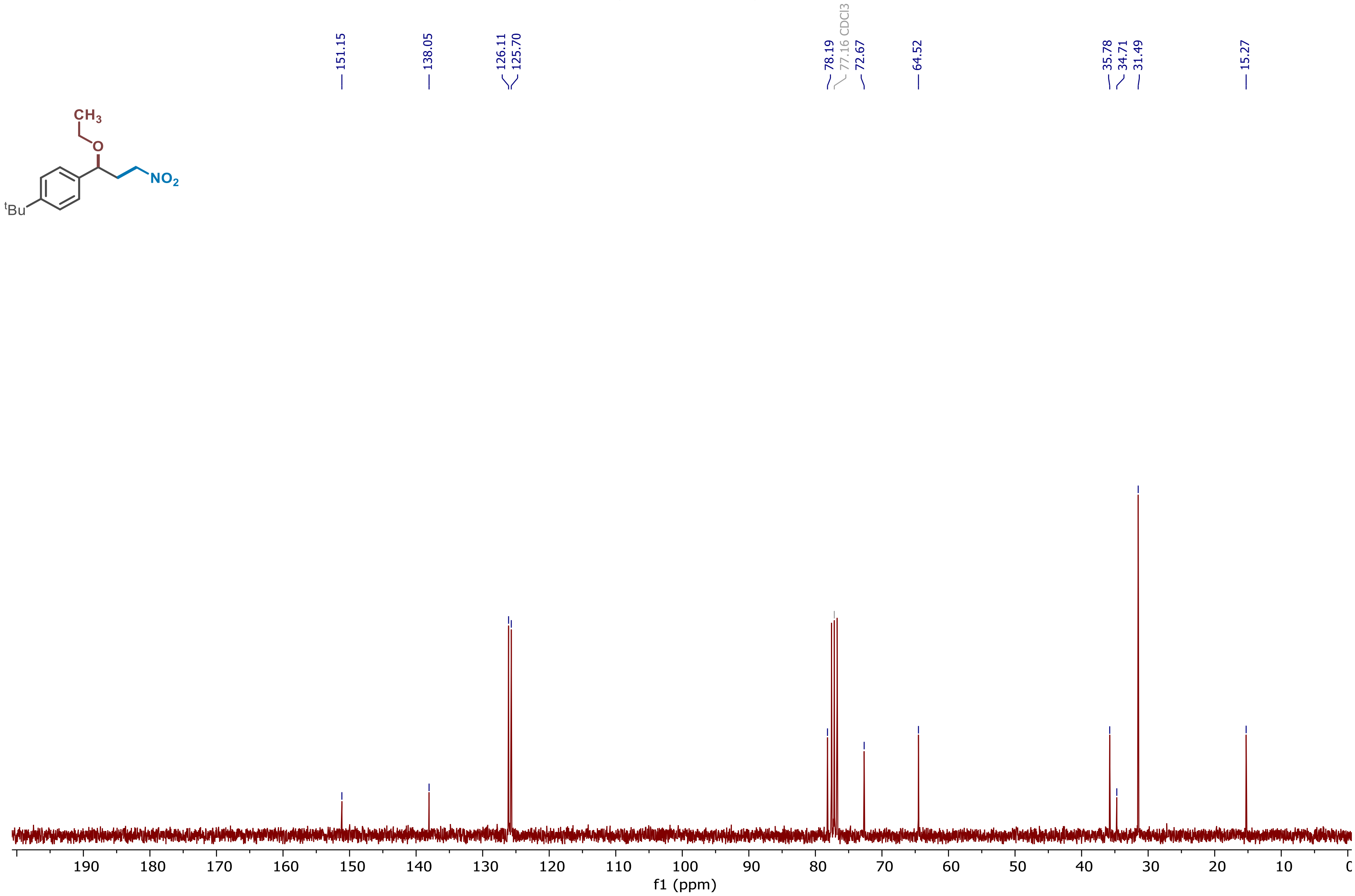

^{1}H NMR (300 MHz, $CDCl_3$) of **3**

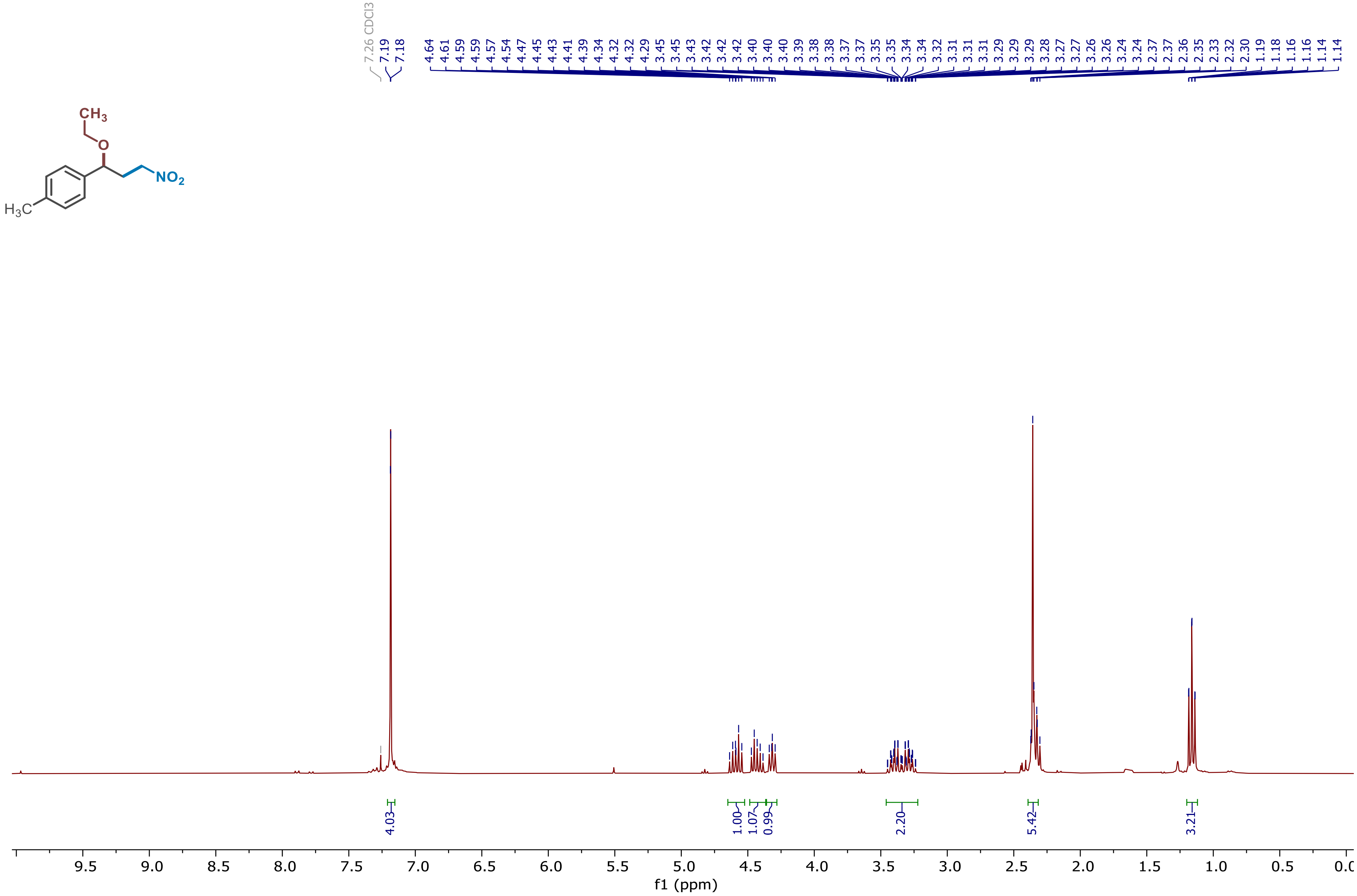

^{13}C NMR (75 MHz, CDCl$_3$) of **3**

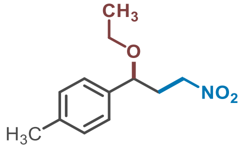

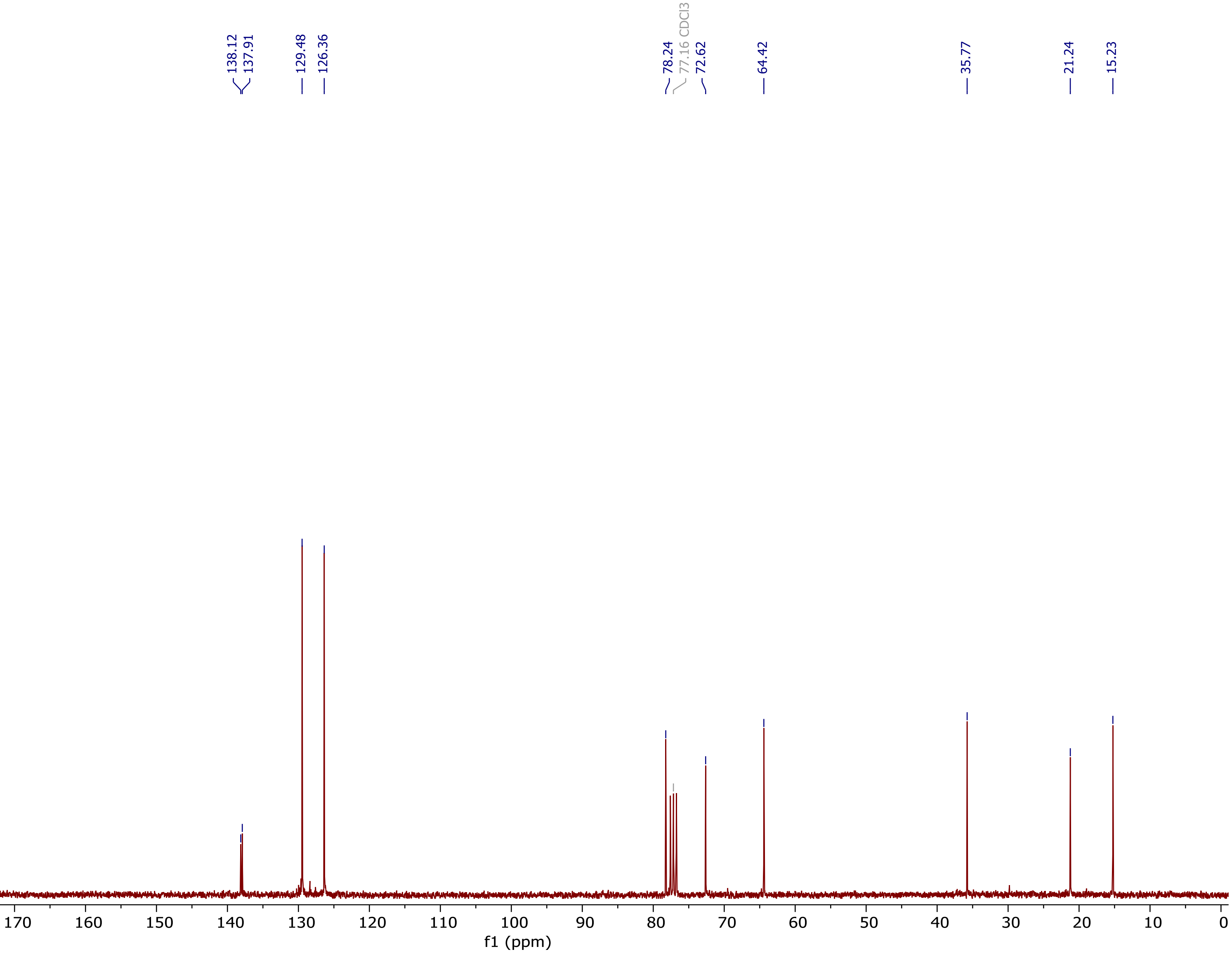

^{1}H NMR (300 MHz, $CDCl_3$) of **4**

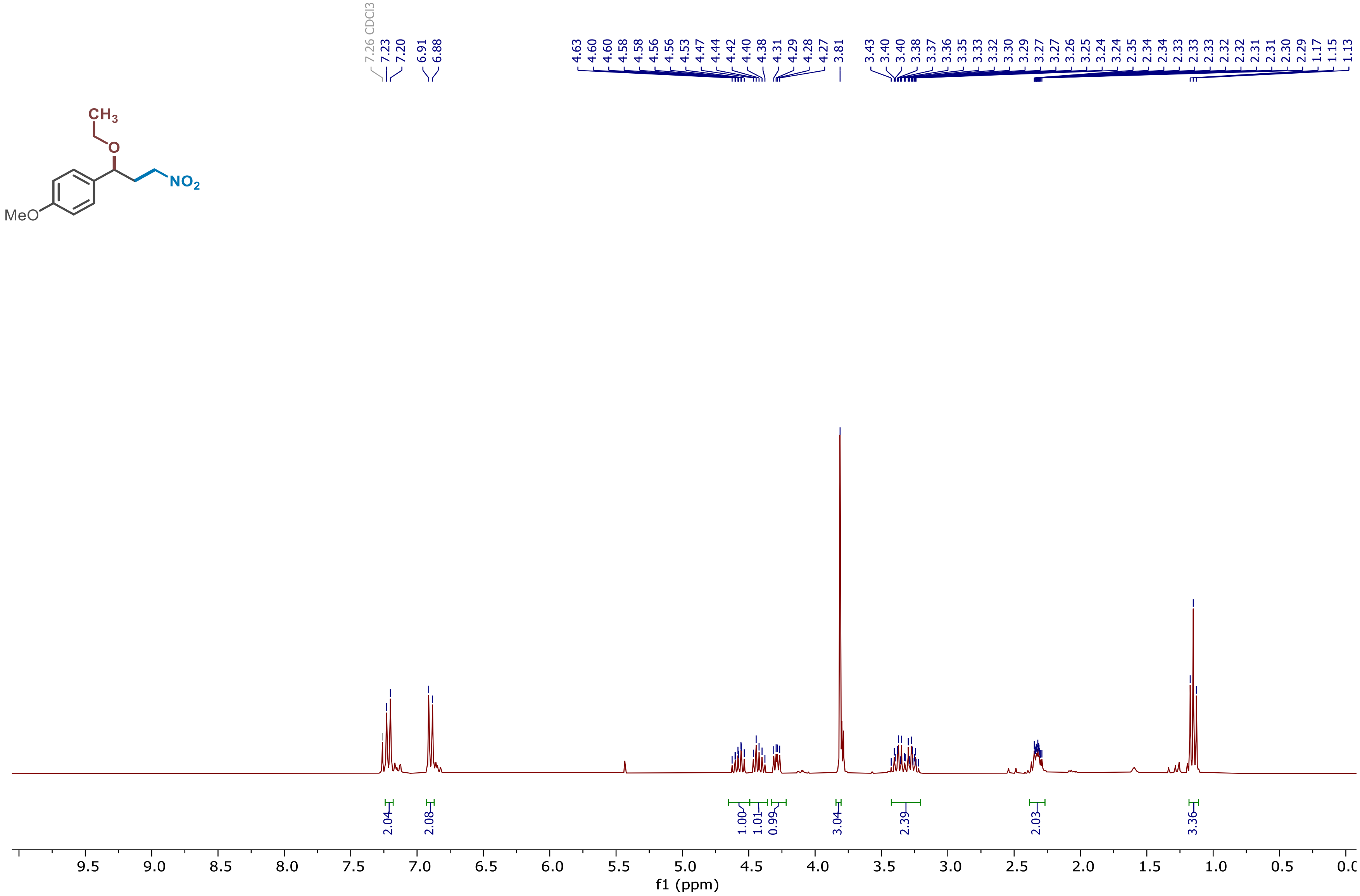

^{13}C NMR (75 MHz, $CDCl_3$) of **4**

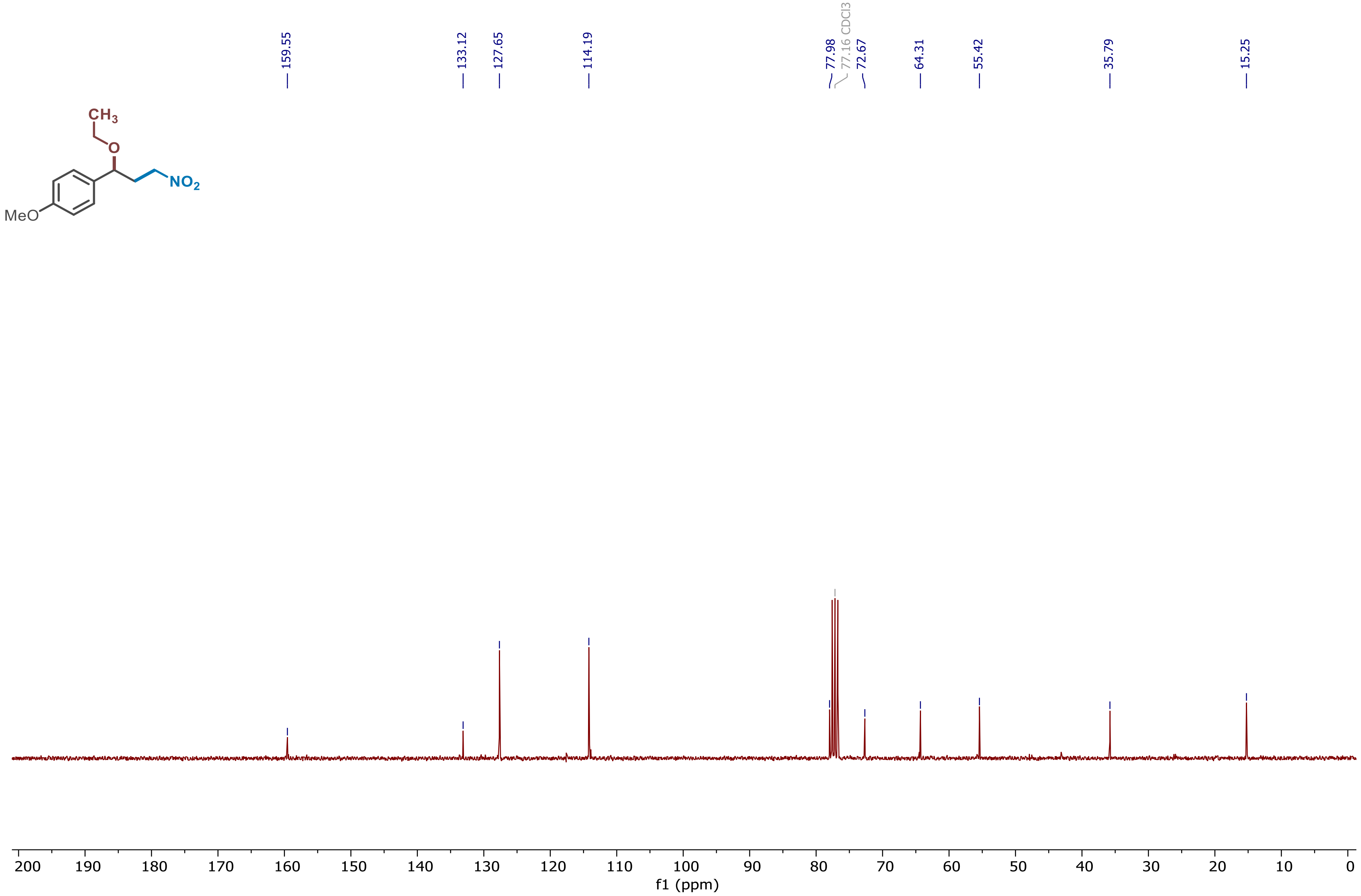

^{1}H NMR (300 MHz, $CDCl_3$) of **5**

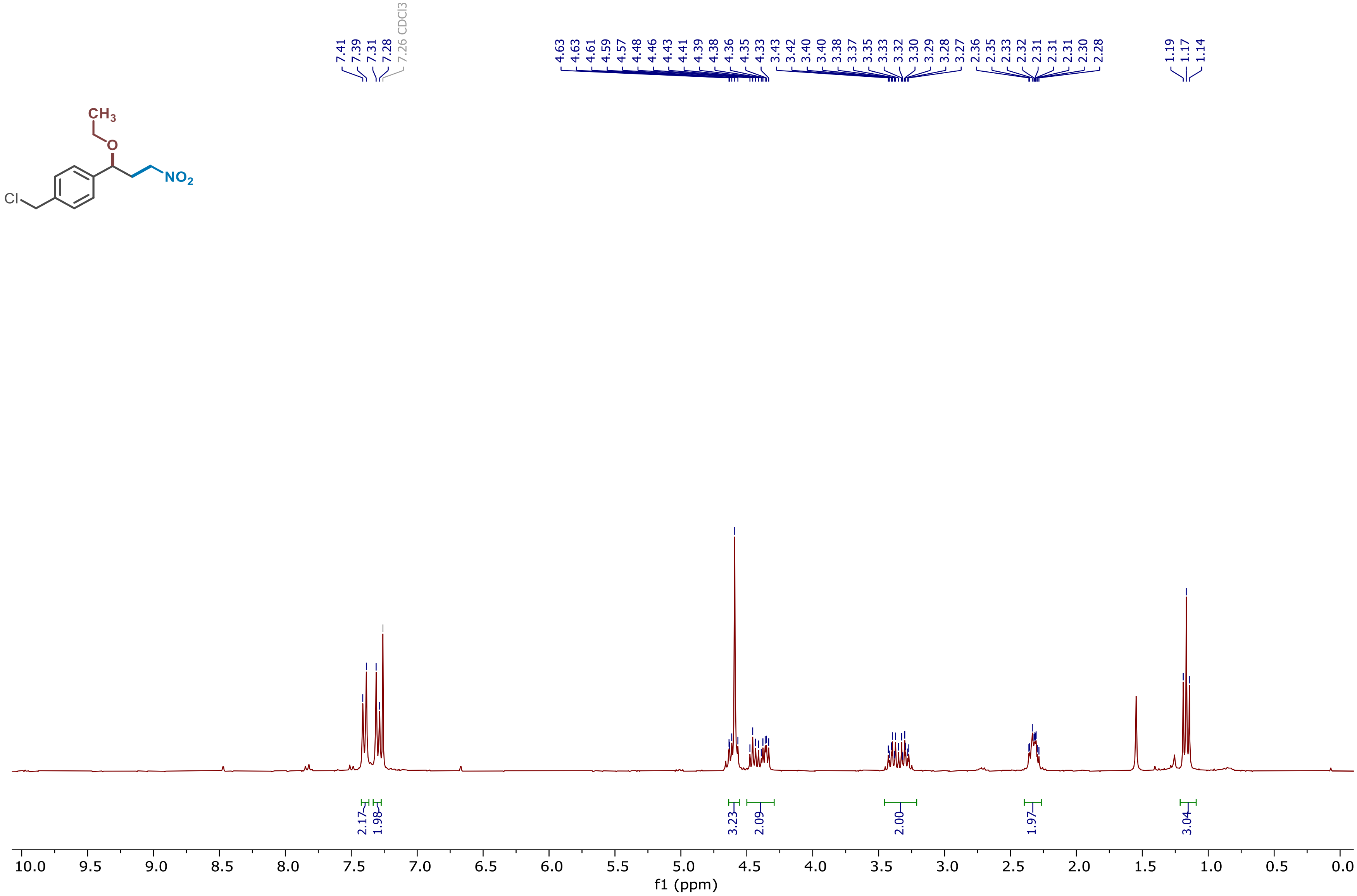

^{13}C NMR (75 MHz, $CDCl_3$) of **5**

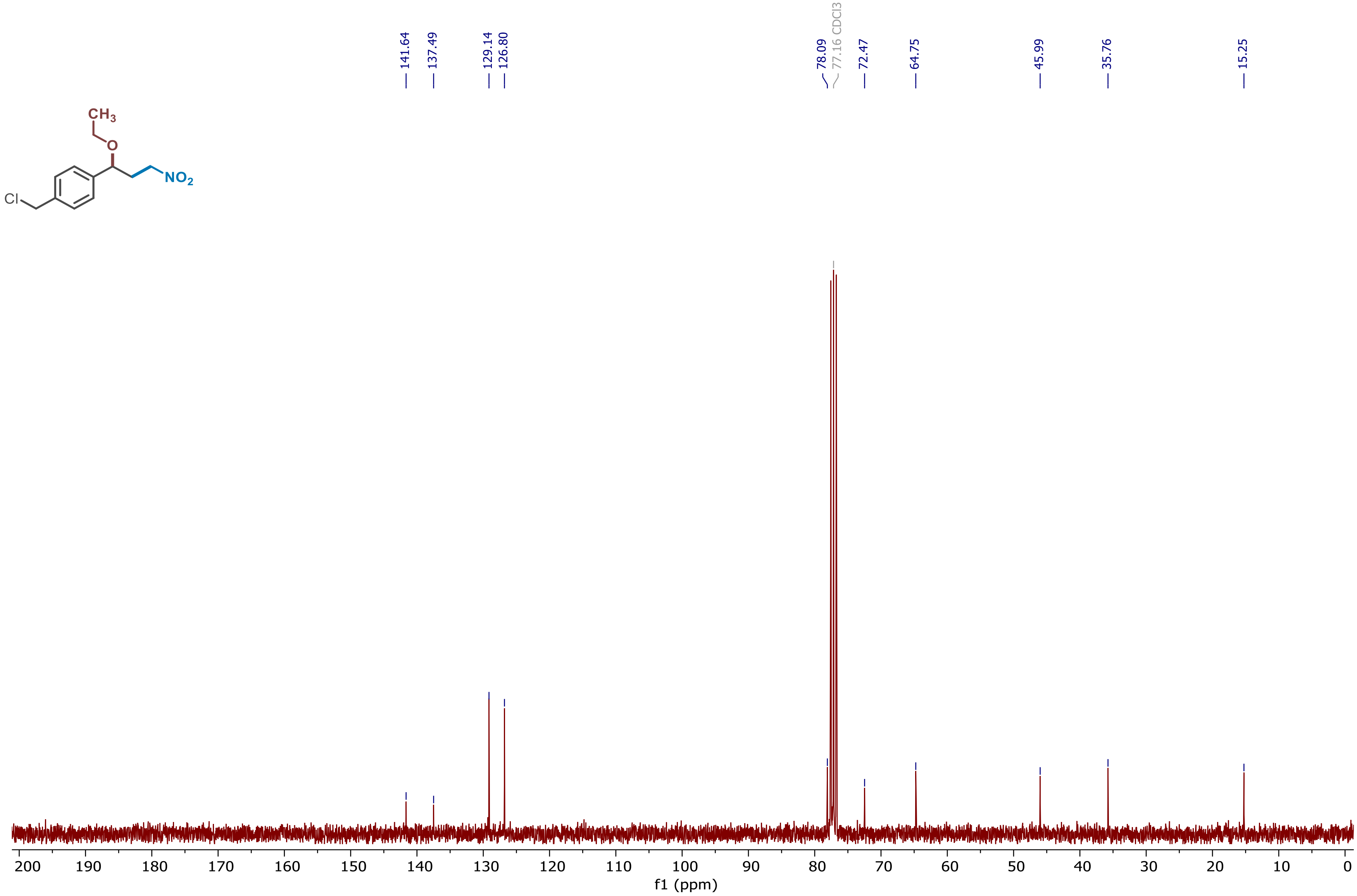

^{1}H NMR (300 MHz, $CDCl_3$) of **6**

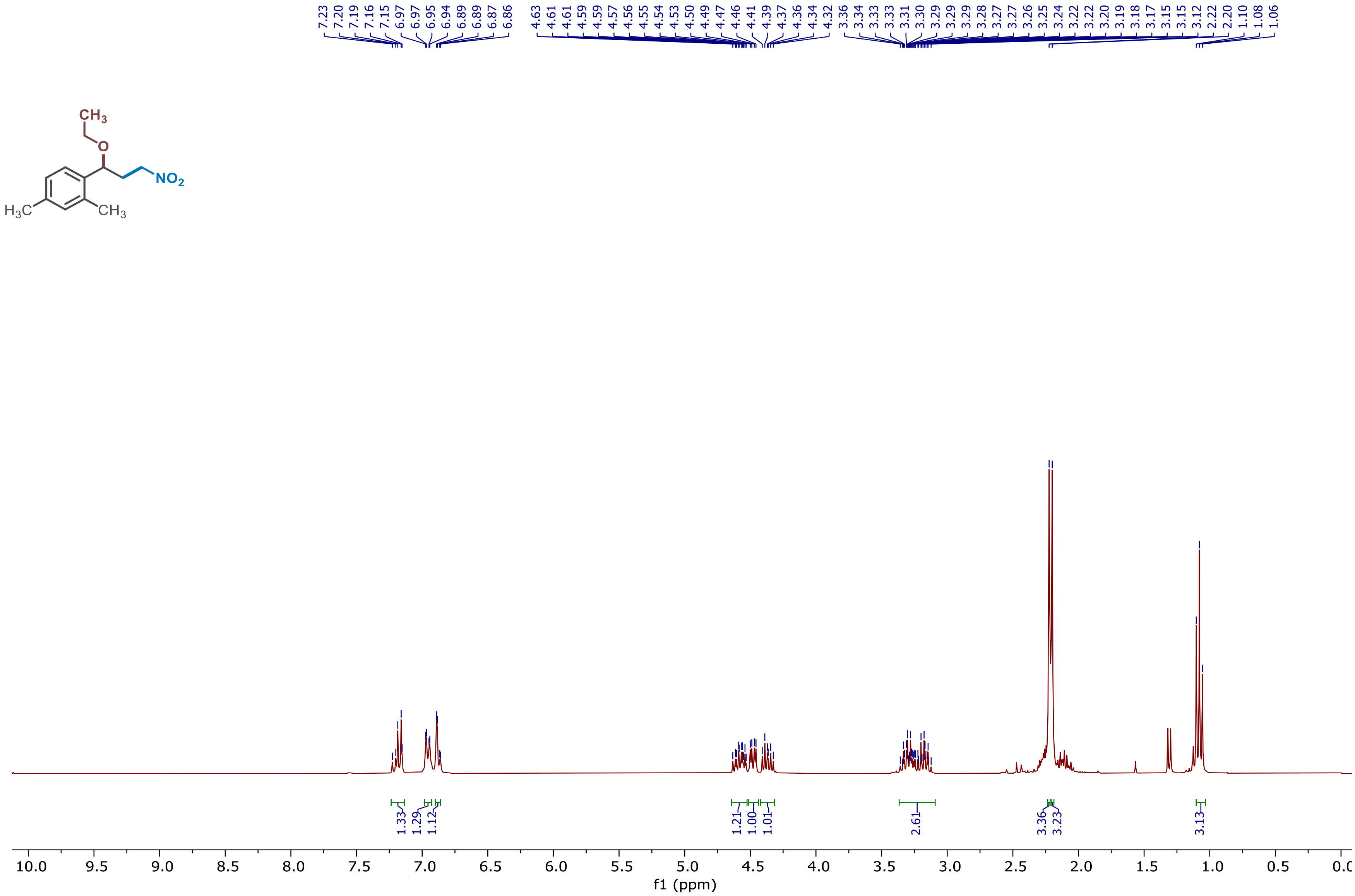

^{13}C NMR (75 MHz, $CDCl_3$) of **6**

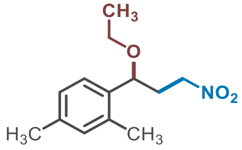

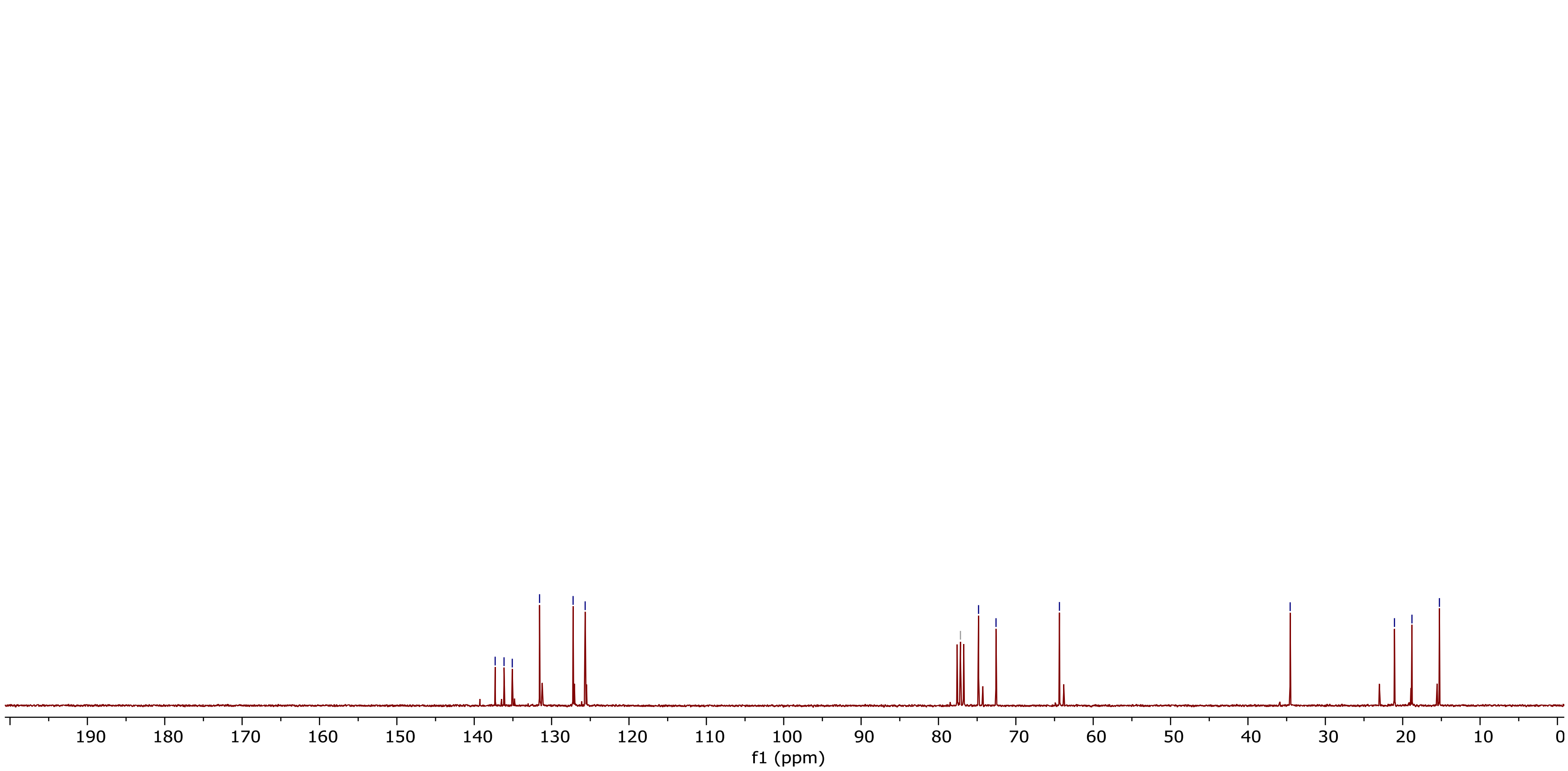

^{1}H NMR (300 MHz, $CDCl_3$) of **7**

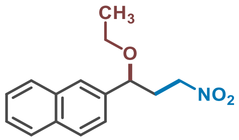

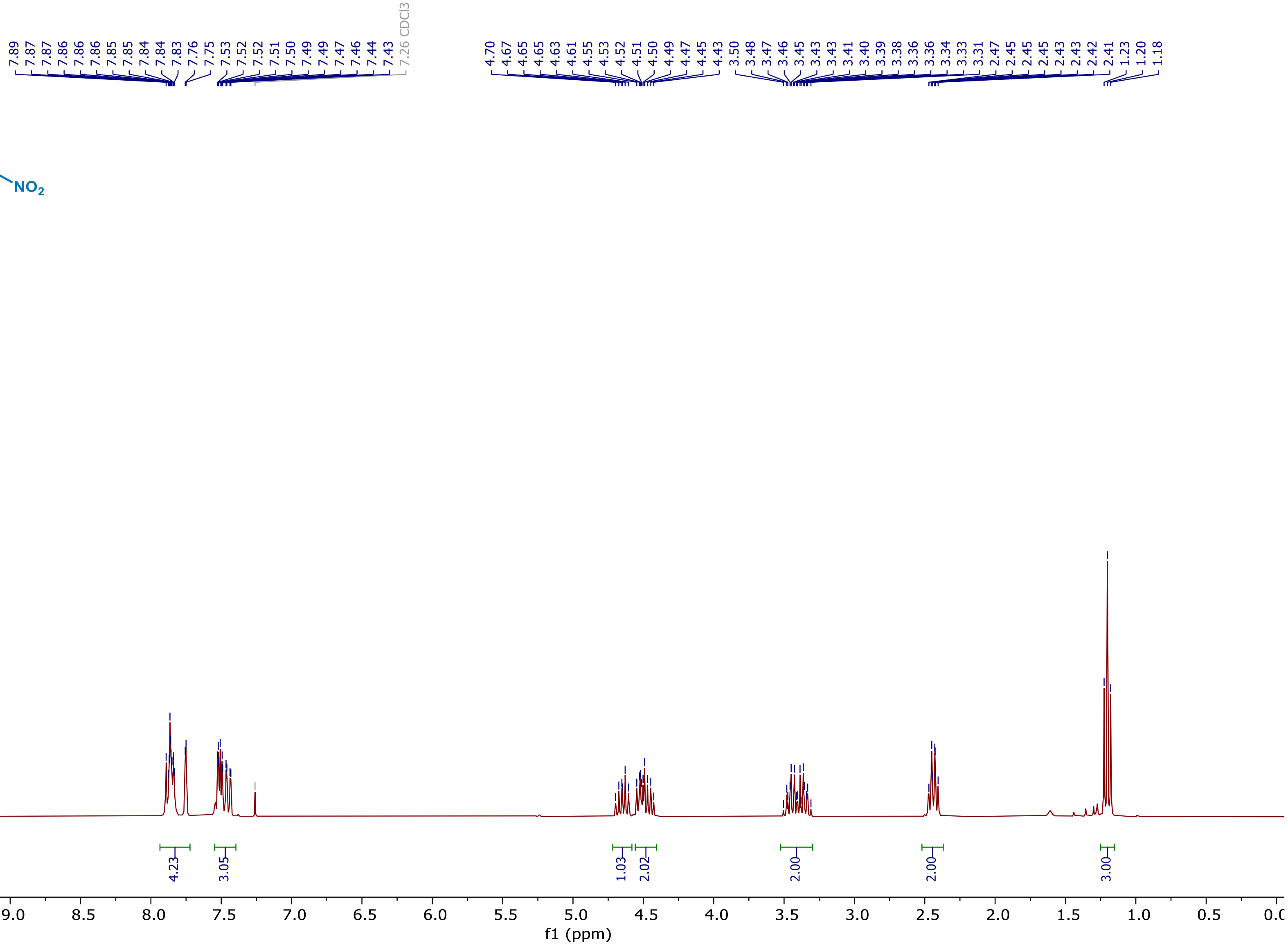

^{13}C NMR (75 MHz, CDCl$_3$) of **7**

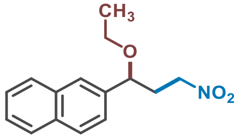

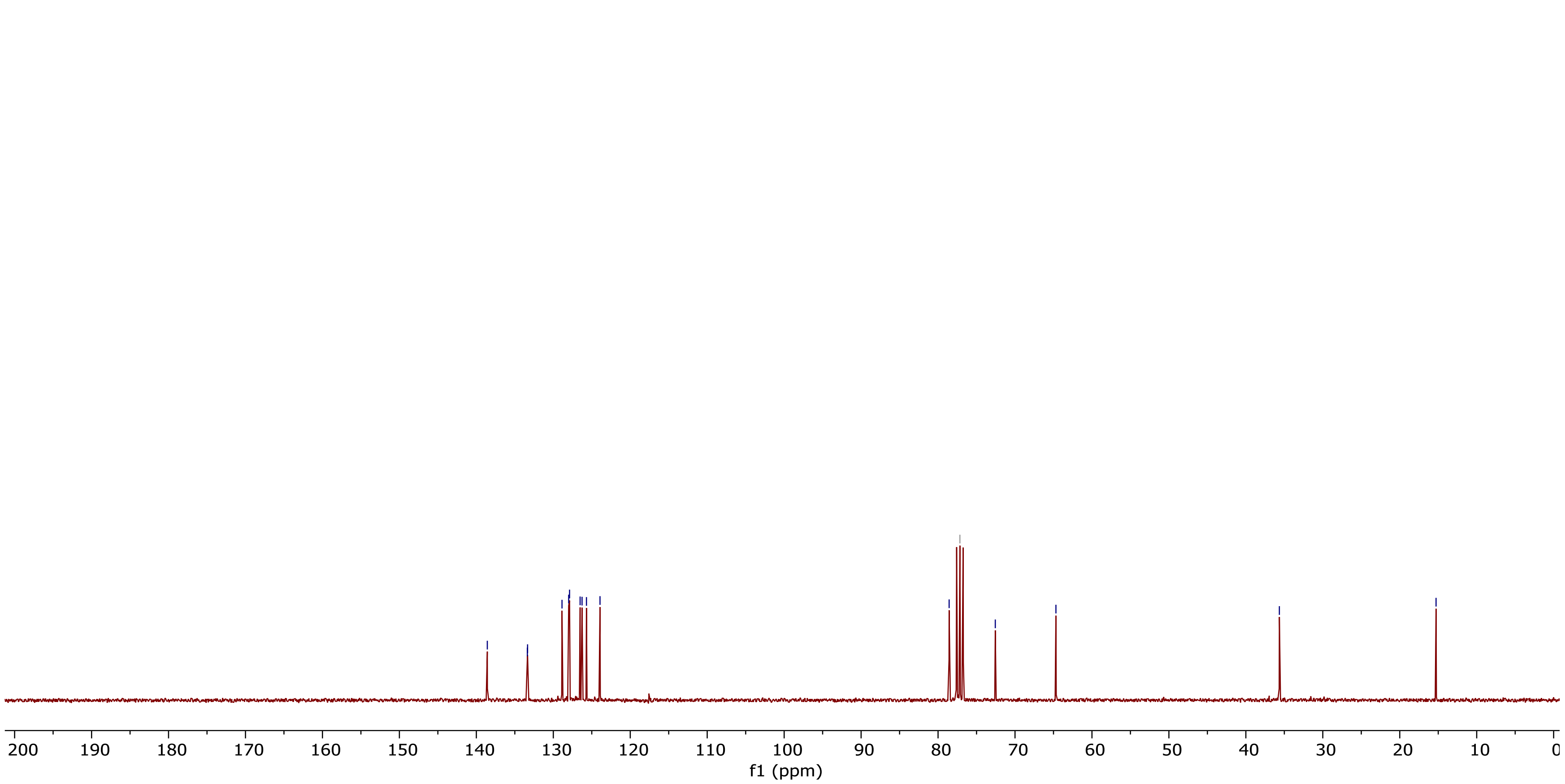

^{1}H NMR (300 MHz, $CDCl_3$) of **8**

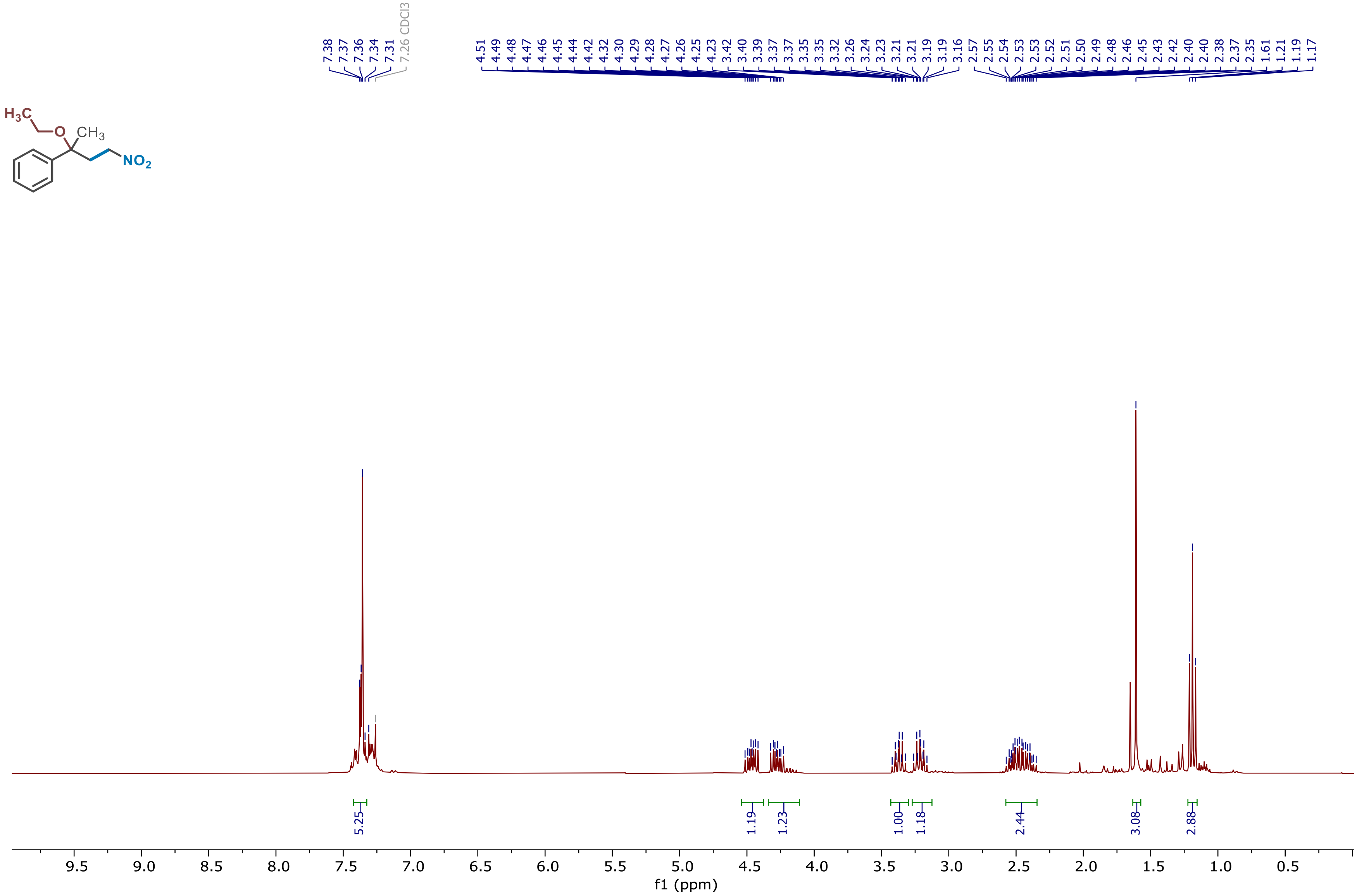

^{13}C NMR (75 MHz, $CDCl_3$) of **8**

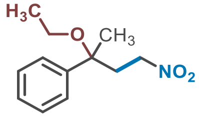

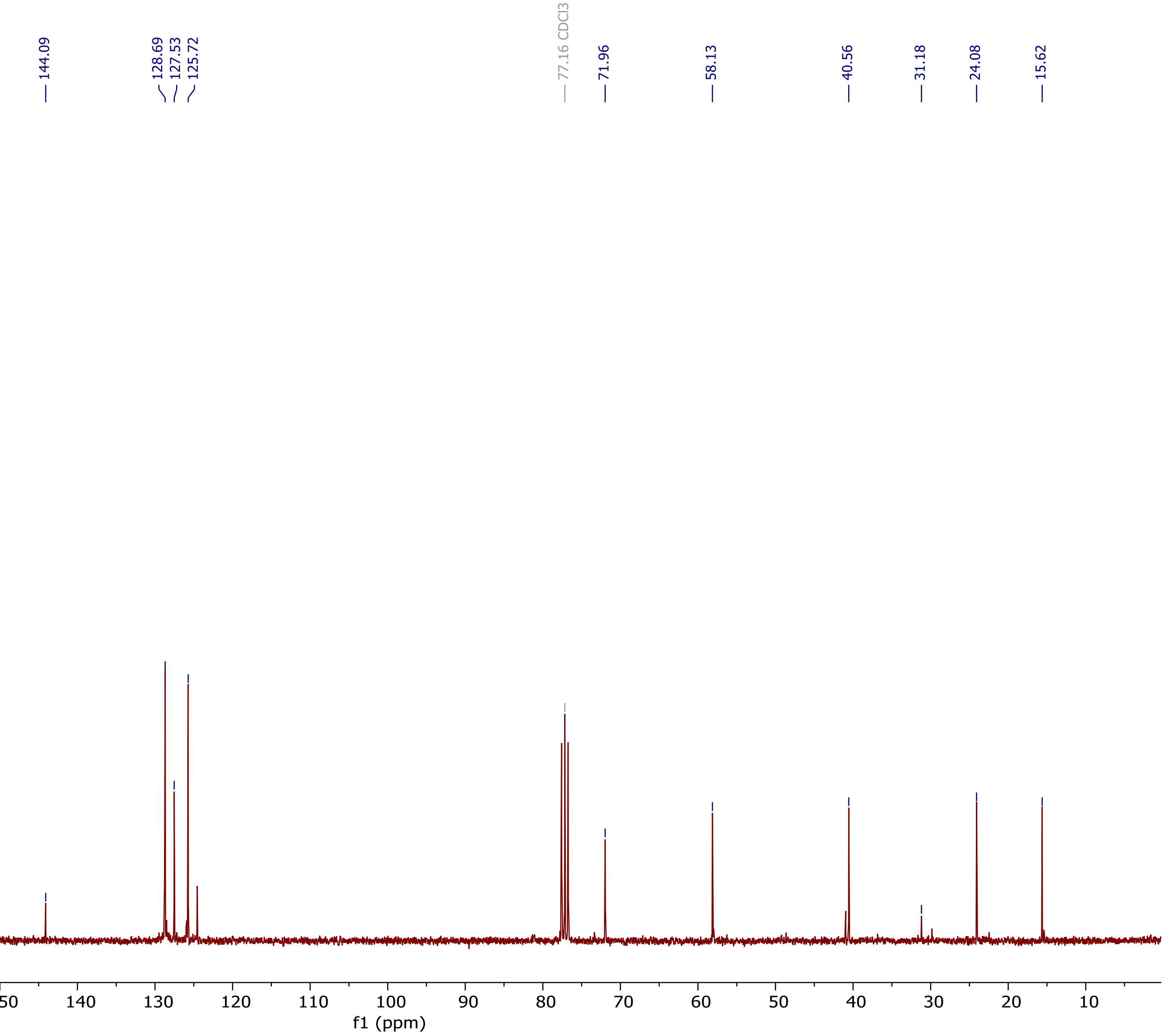

^{1}H NMR (300 MHz, $CDCl_3$) of **9**

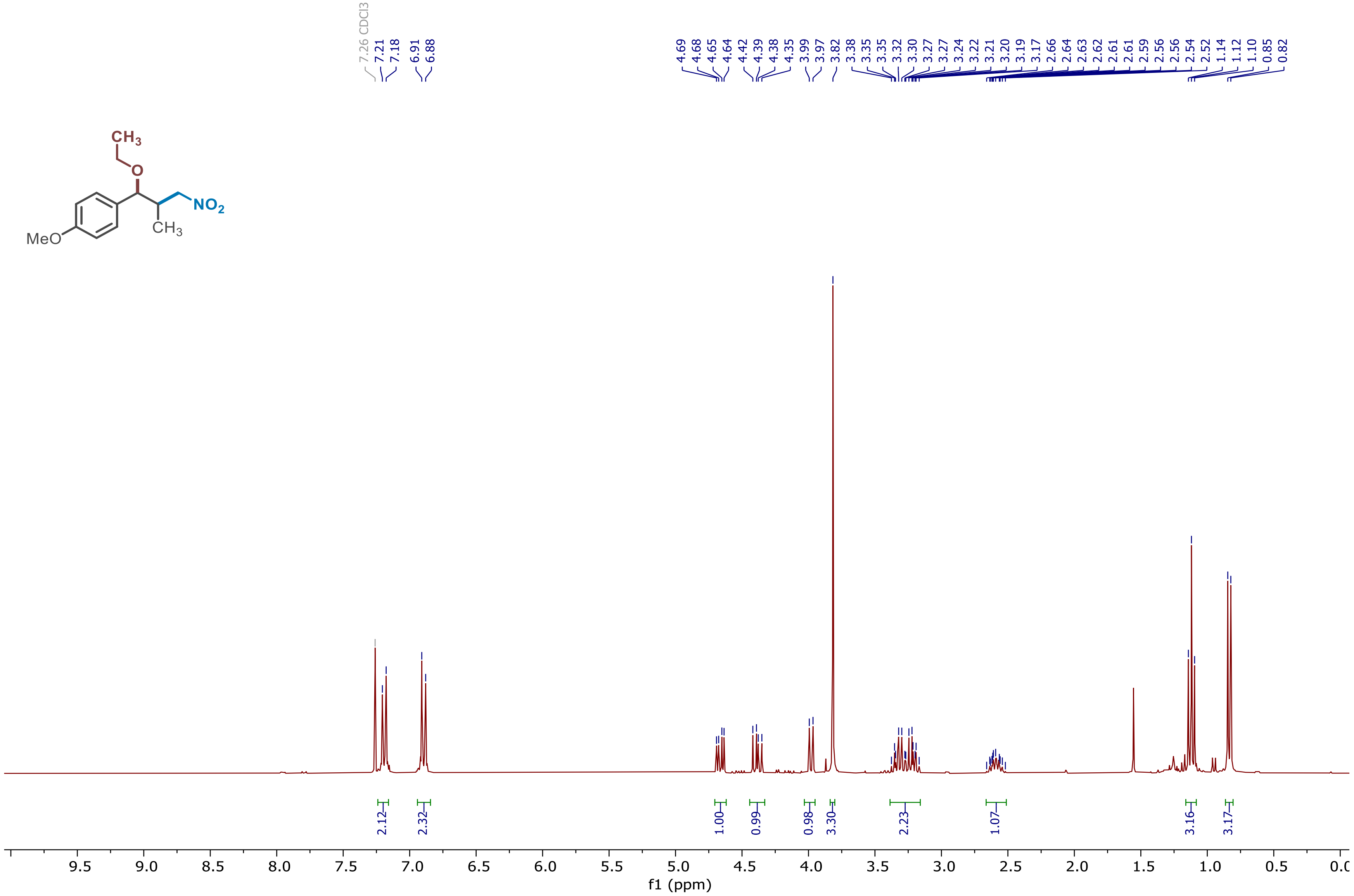

^{13}C NMR (75 MHz, $CDCl_3$) of **9**

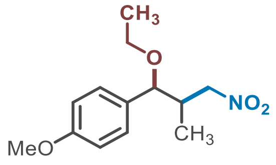

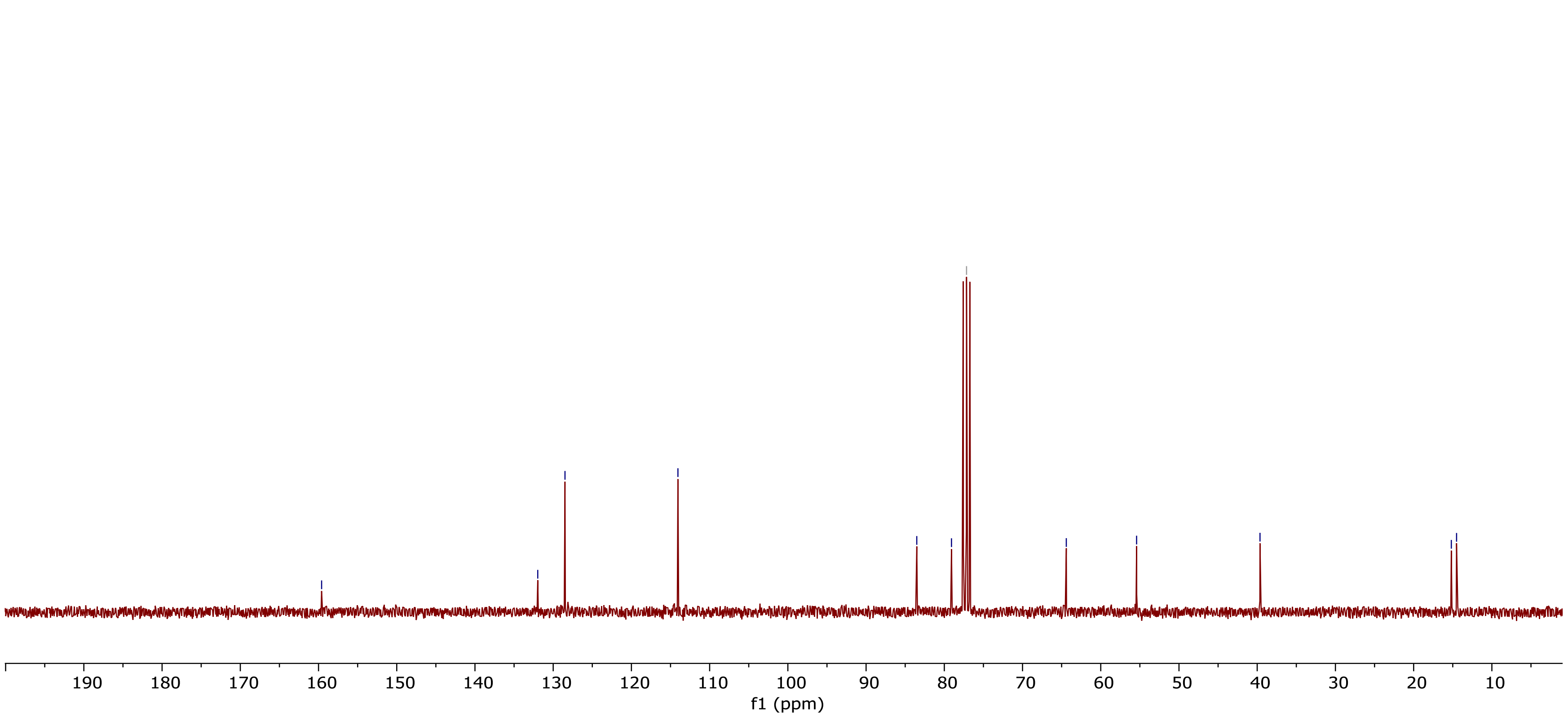

^1H NMR (300 MHz, $CDCl_3$) of **10**

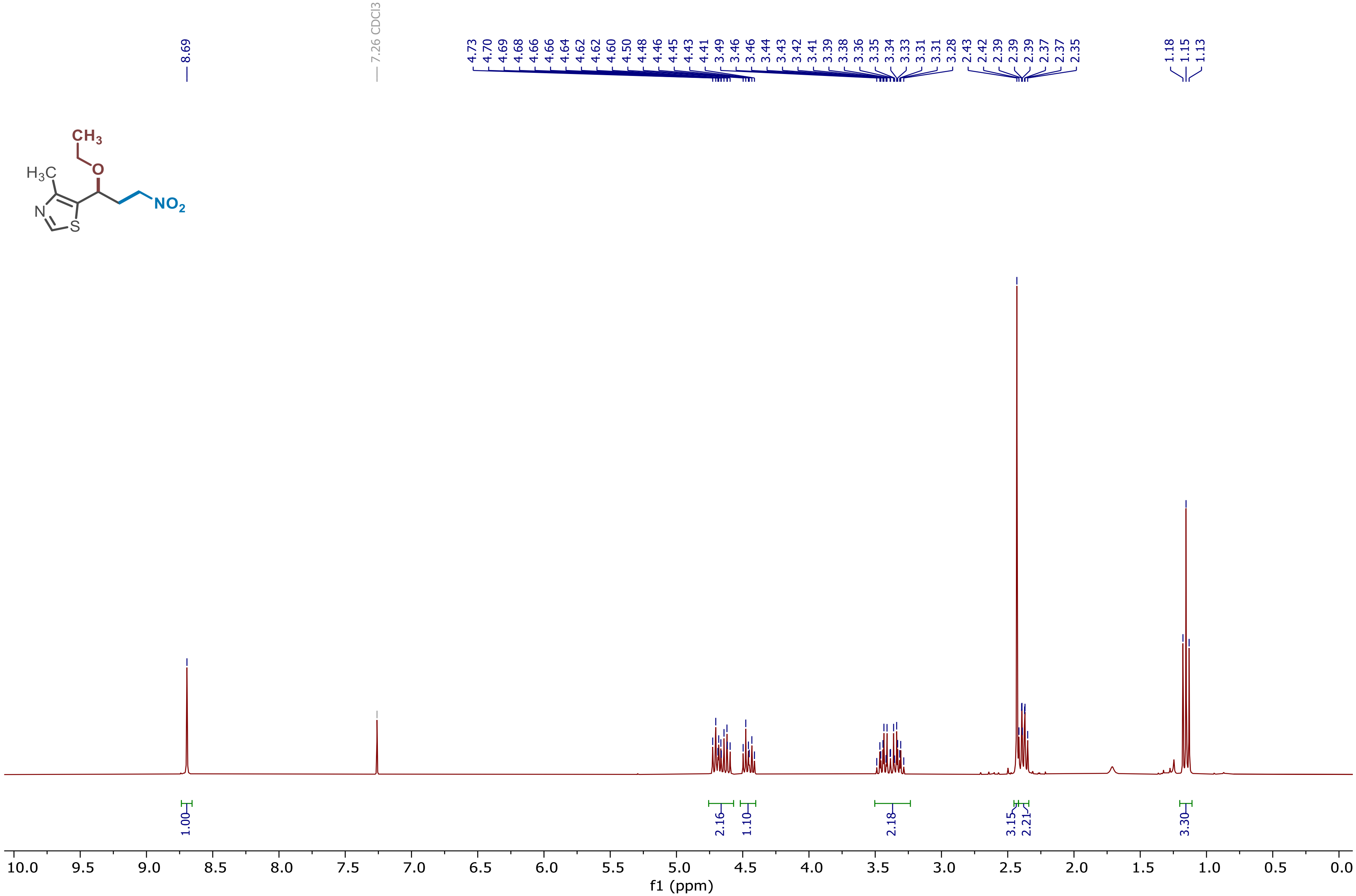

^{13}C NMR (75 MHz, $CDCl_3$) of **10**

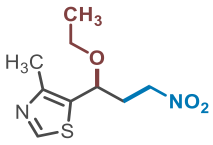

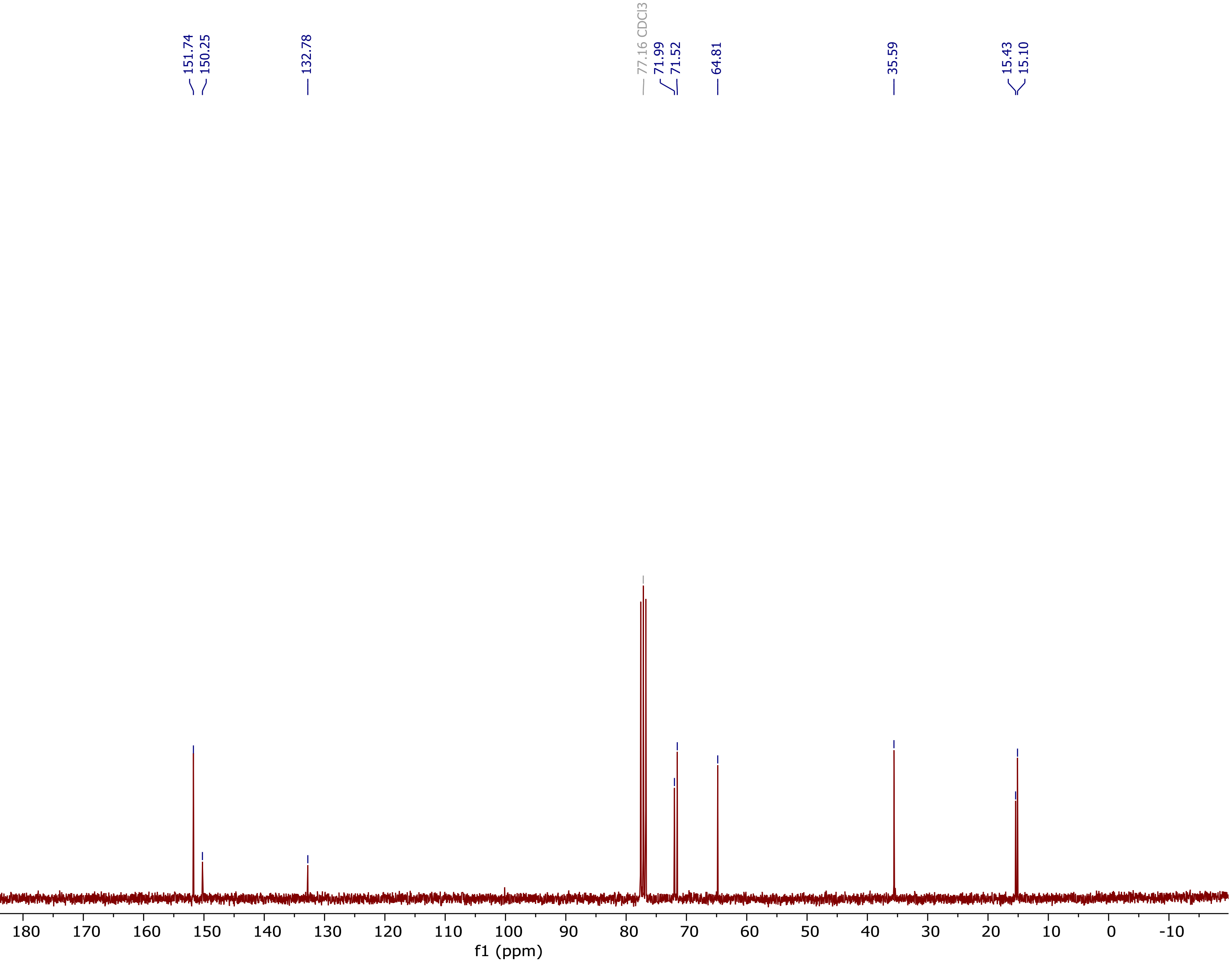

^{1}H NMR (300 MHz, $CDCl_3$) of **11**

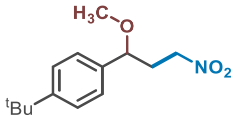

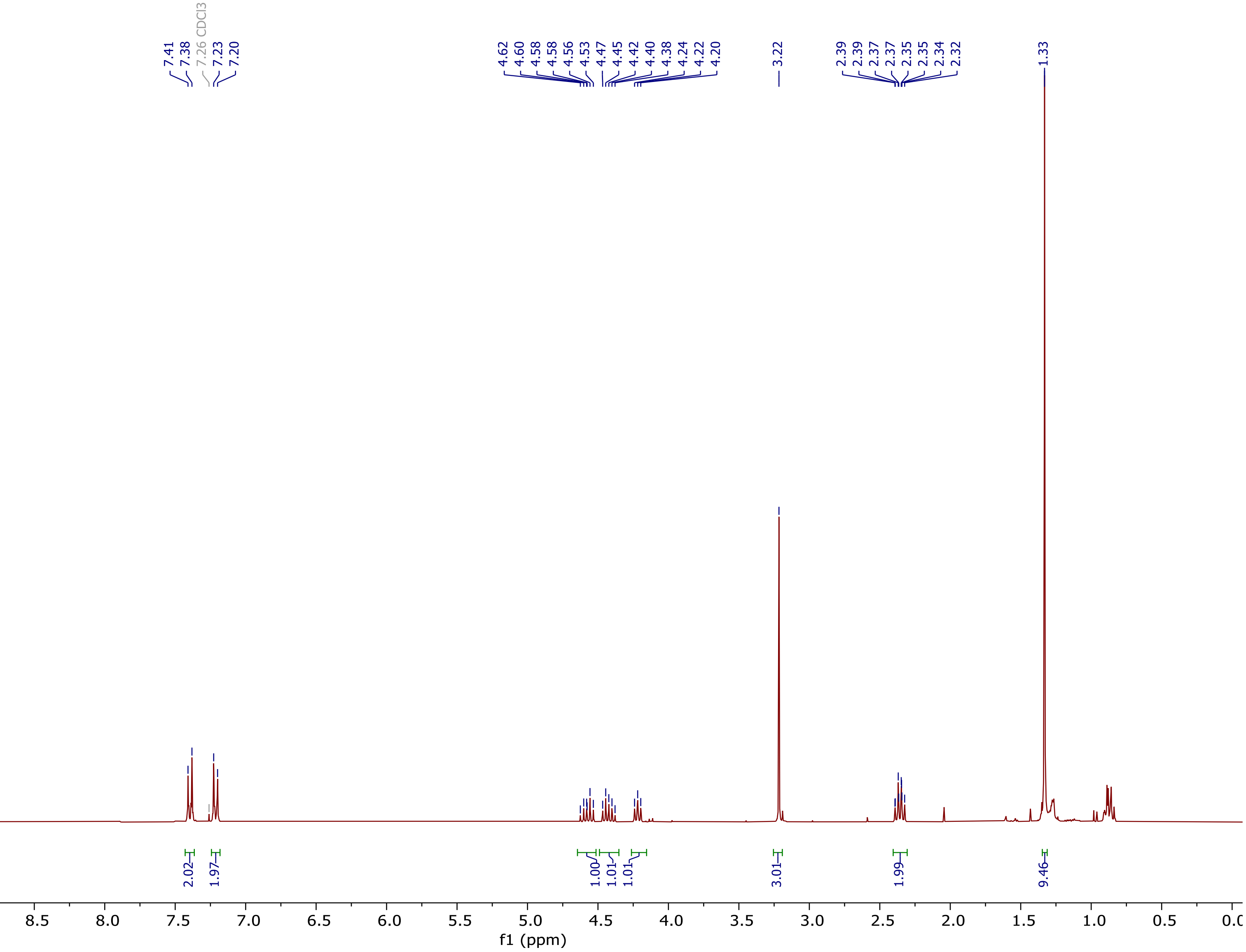

^{13}C NMR (75 MHz, $CDCl_3$) of **11**

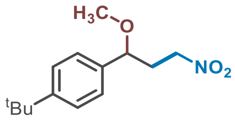

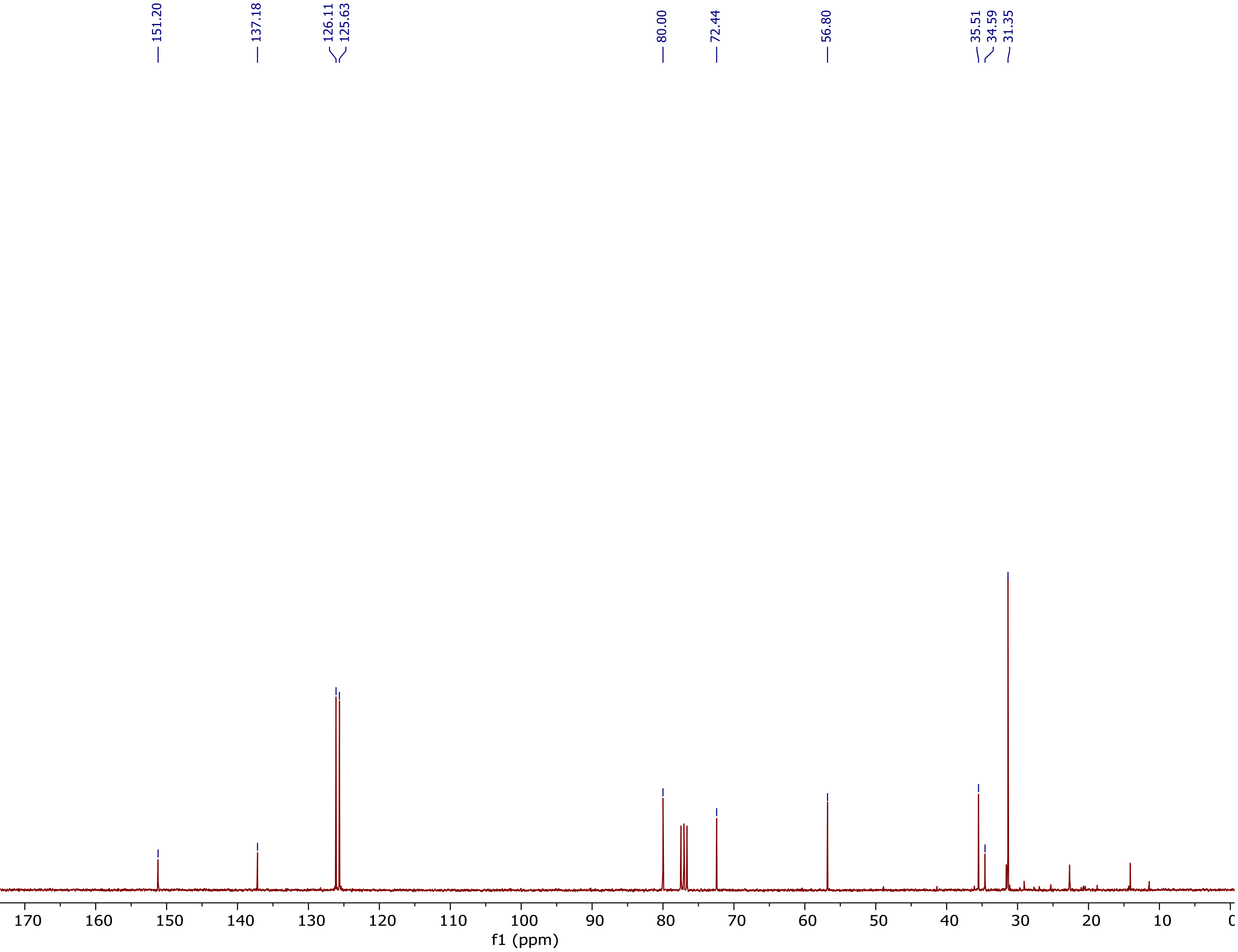

^{1}H NMR (300 MHz, $CDCl_3$) of **12**

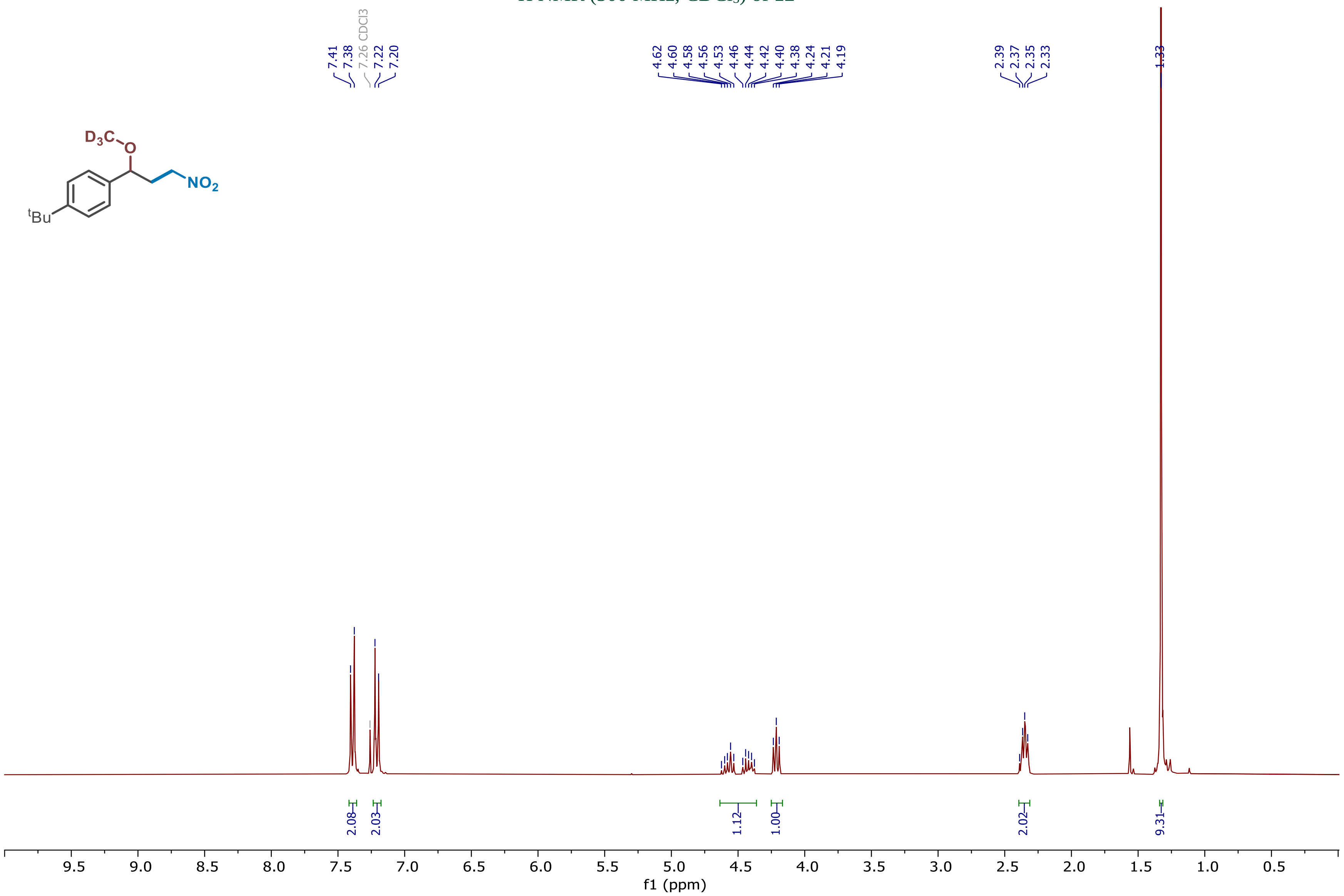

^{13}C NMR (75 MHz, $CDCl_3$) of **12**

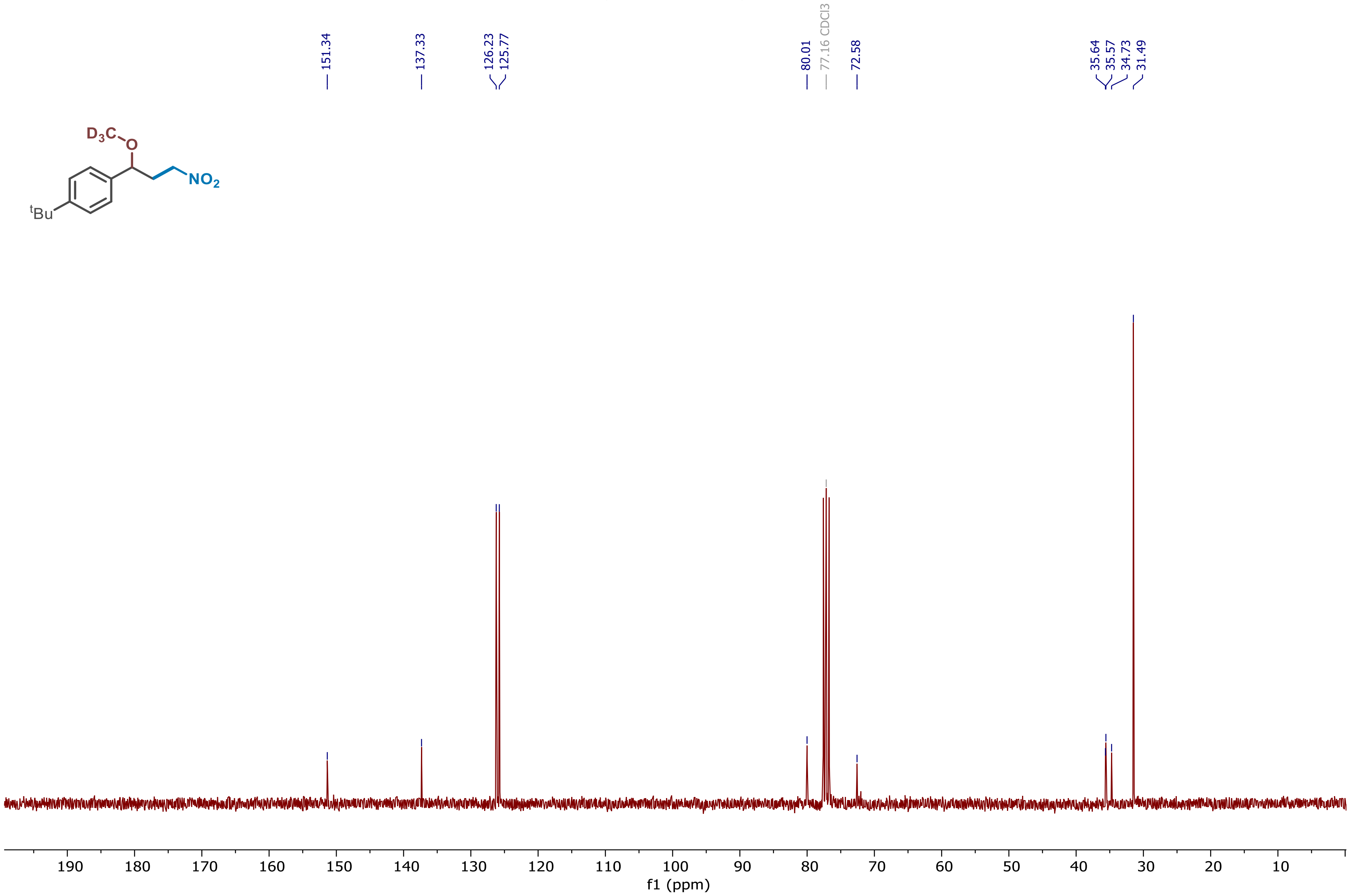

^{1}H NMR (300 MHz, $CDCl_3$) of **13**

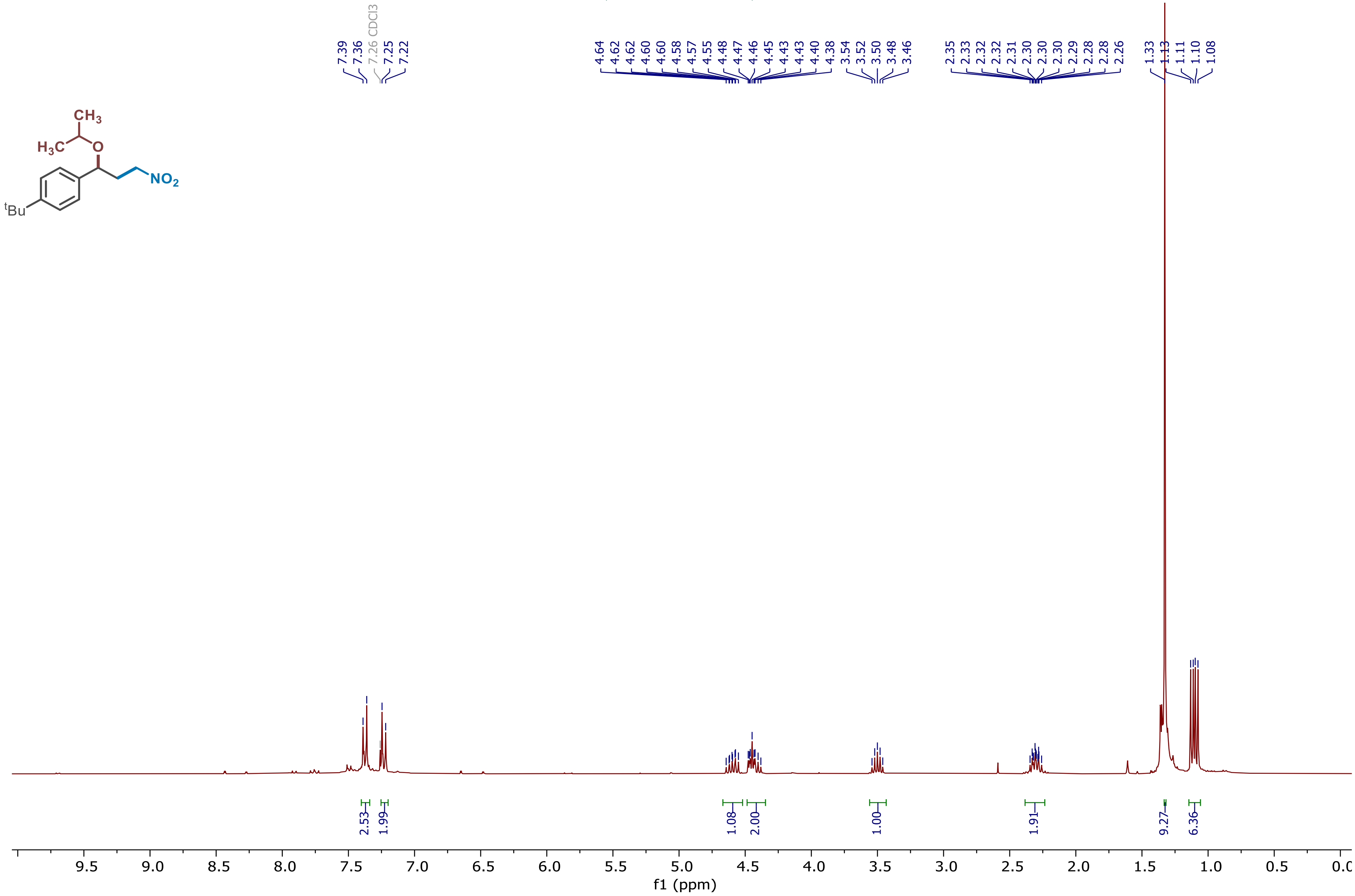

^{13}C NMR (75 MHz, $CDCl_3$) of **13**

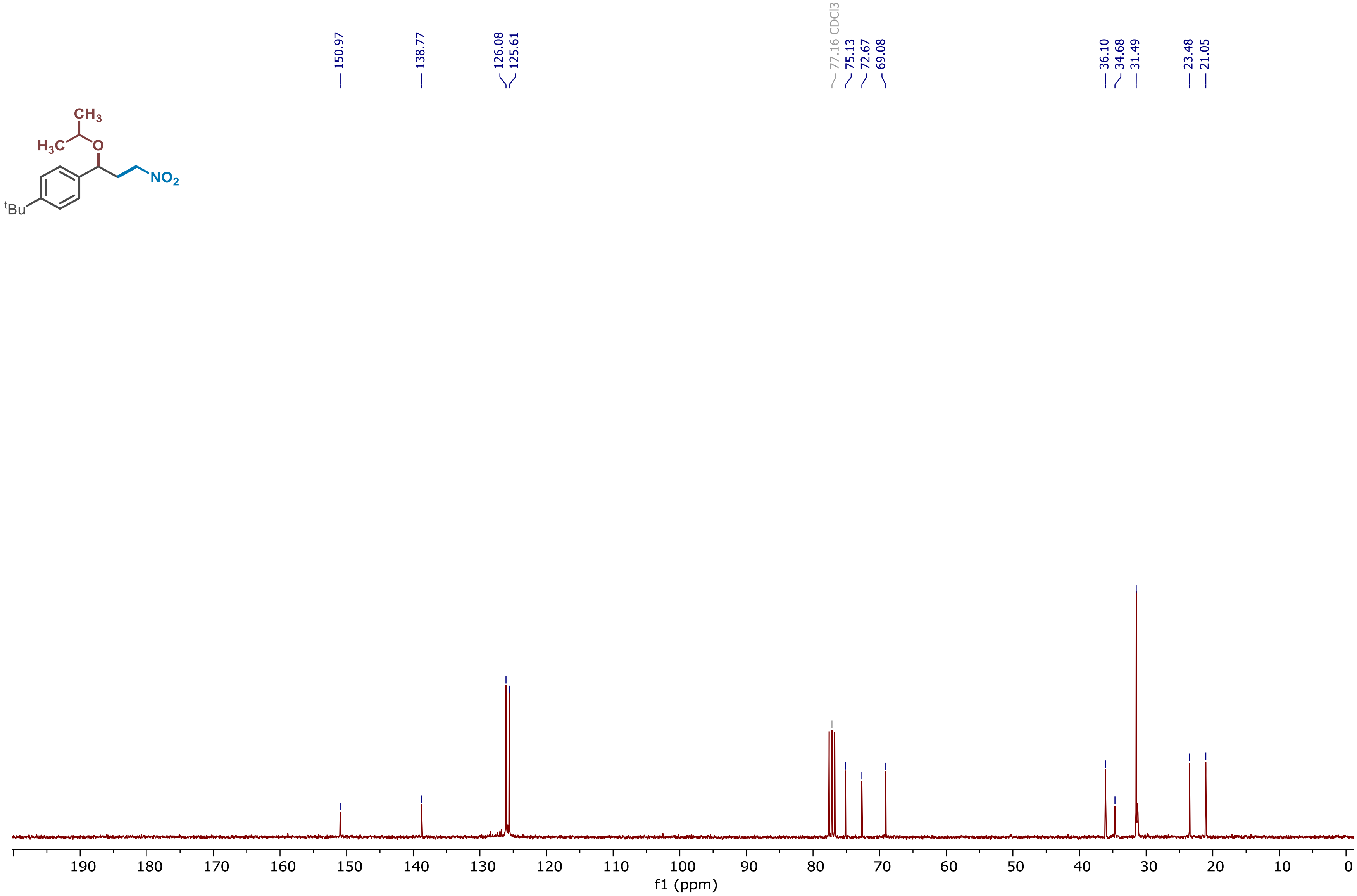

^{1}H NMR (300 MHz, $CDCl_3$) of **14**

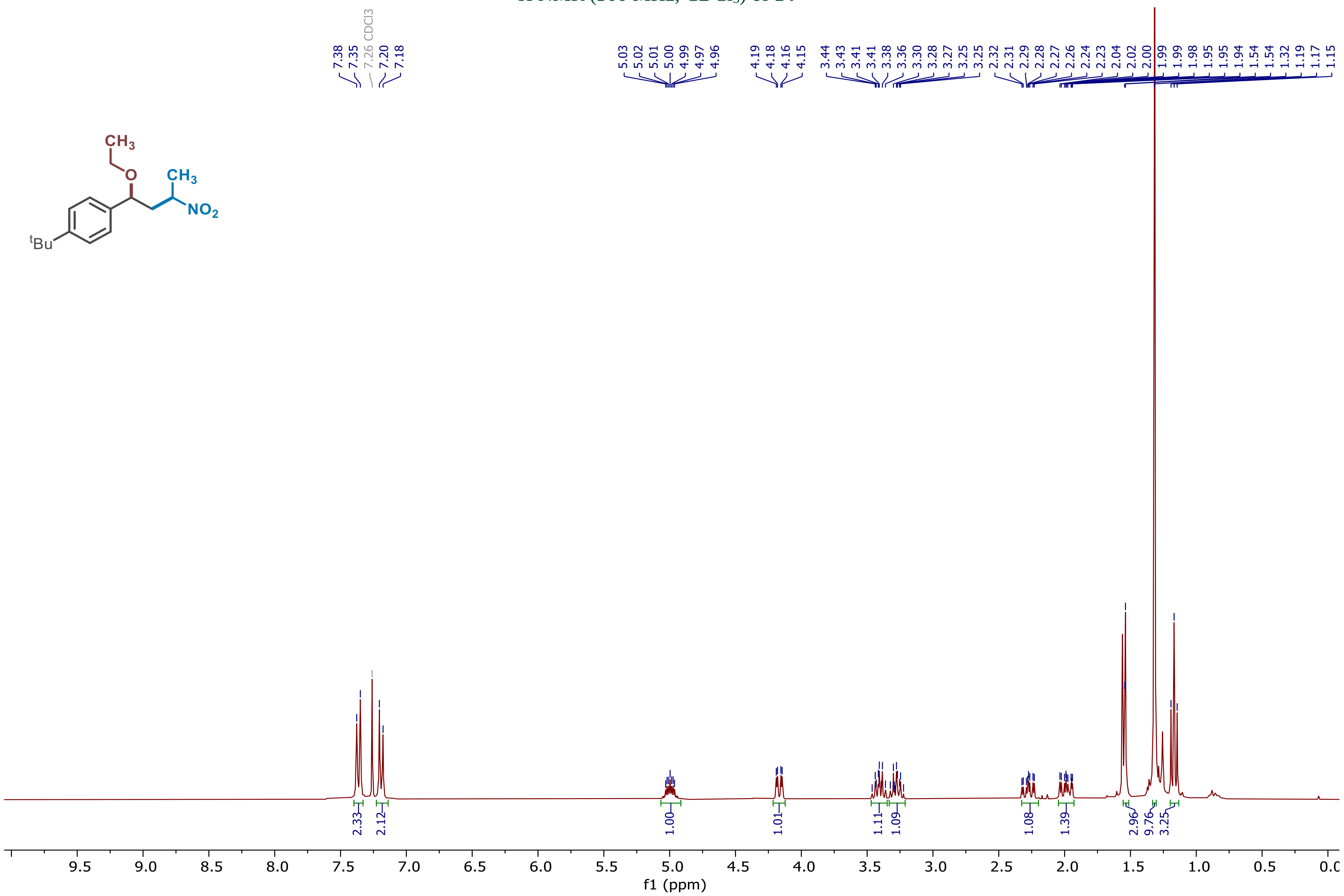

^{13}C NMR (75 MHz, $CDCl_3$) of **14**

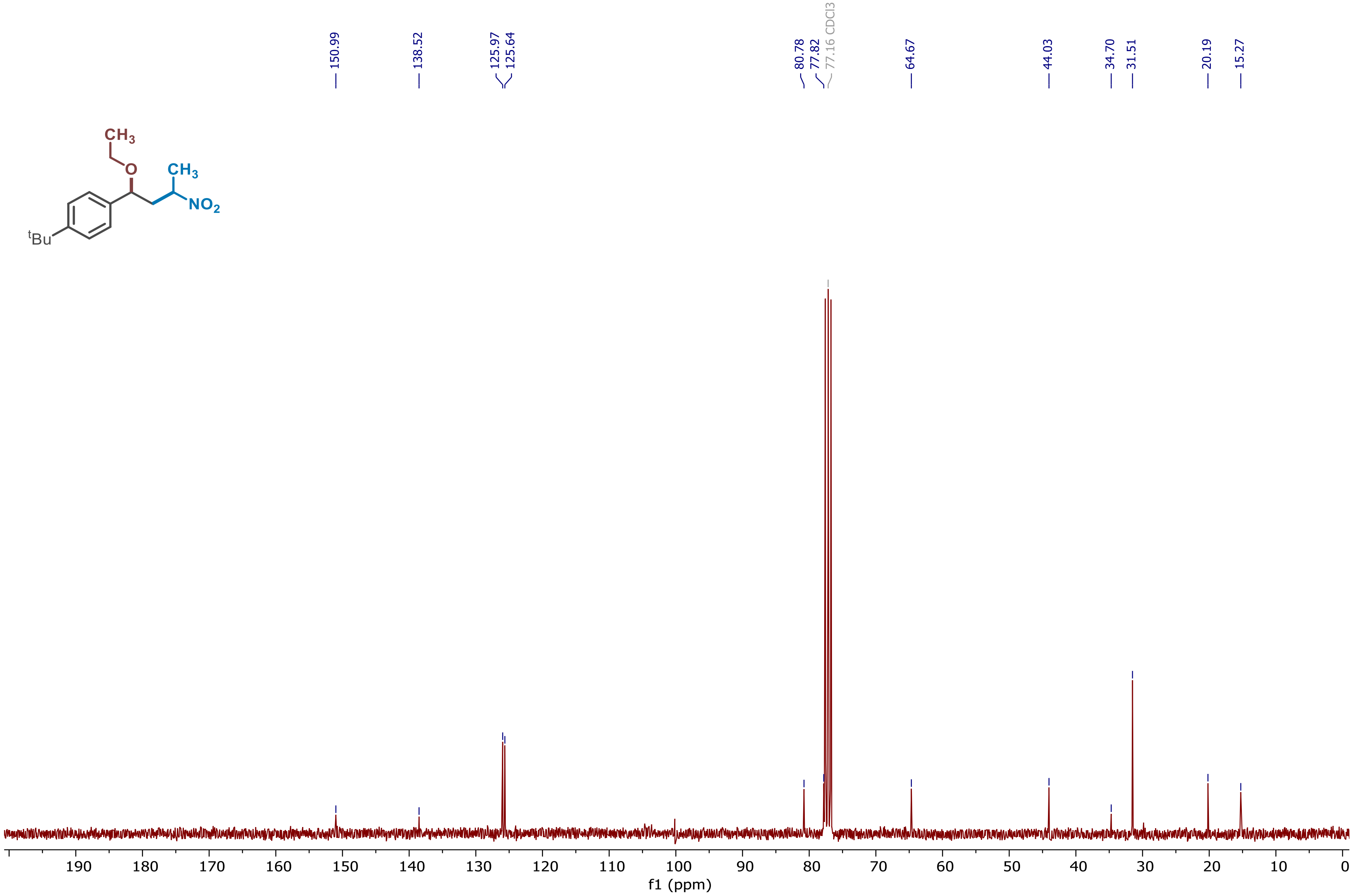

^{1}H NMR (300 MHz, $CDCl_3$) of **15**

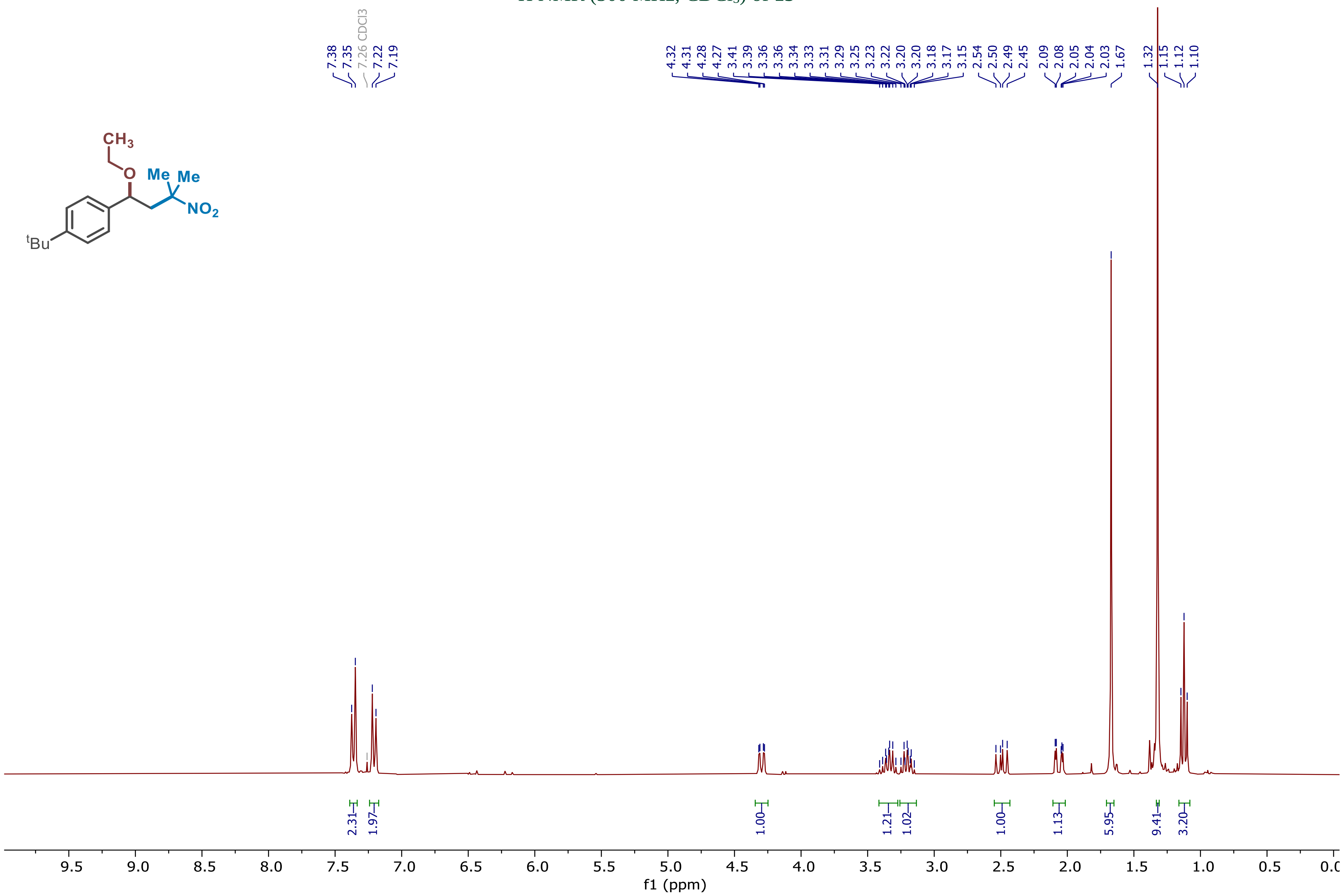

^{13}C NMR (75 MHz, $CDCl_3$) of **15**

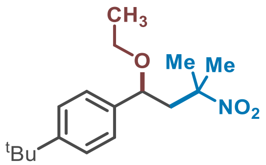

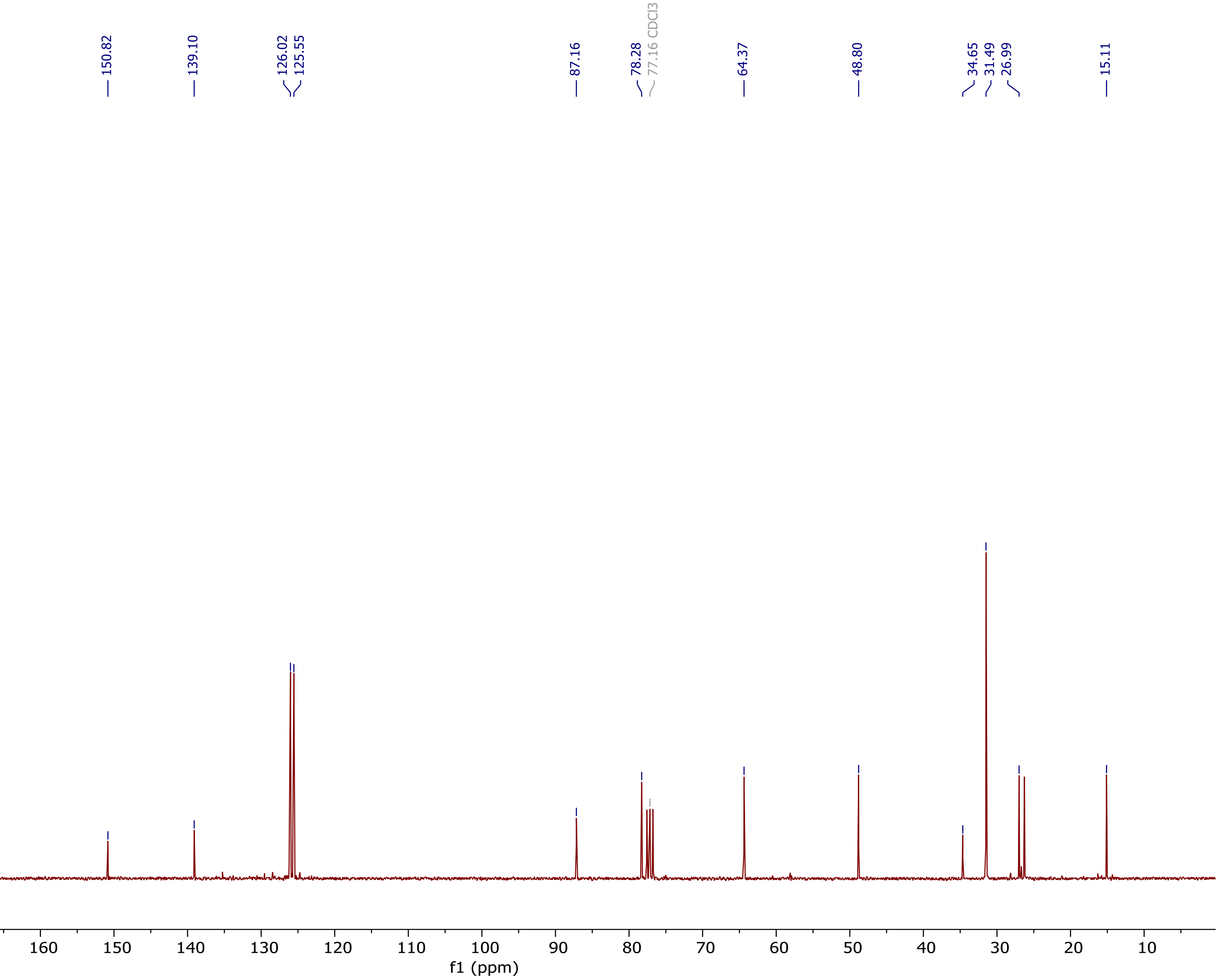

^{1}H NMR (300 MHz, $CDCl_3$) of **16**

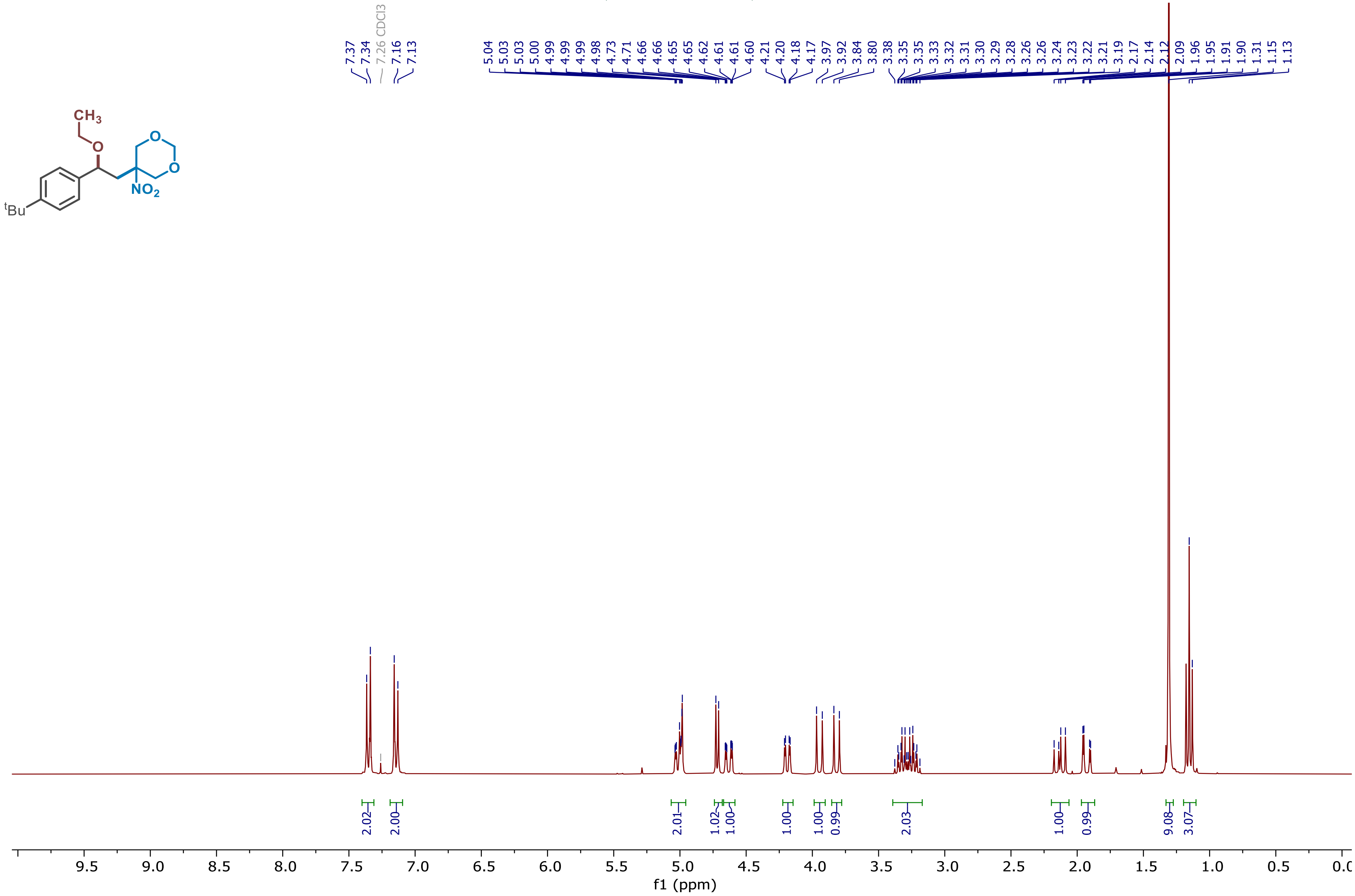

^{13}C NMR (75 MHz, $CDCl_3$) of **16**

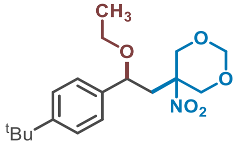

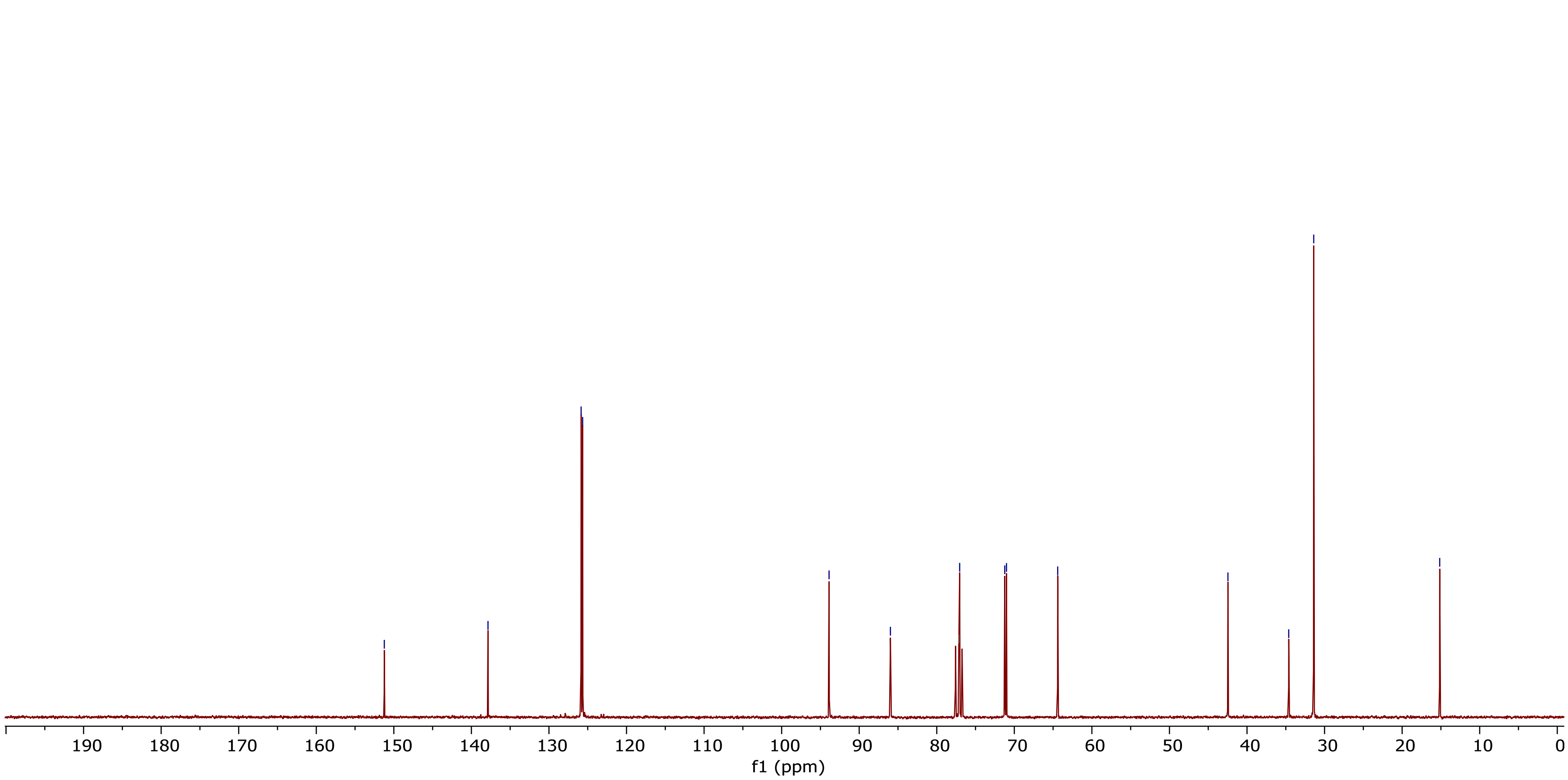

^{1}H NMR (300 MHz, $CDCl_3$) of **17**

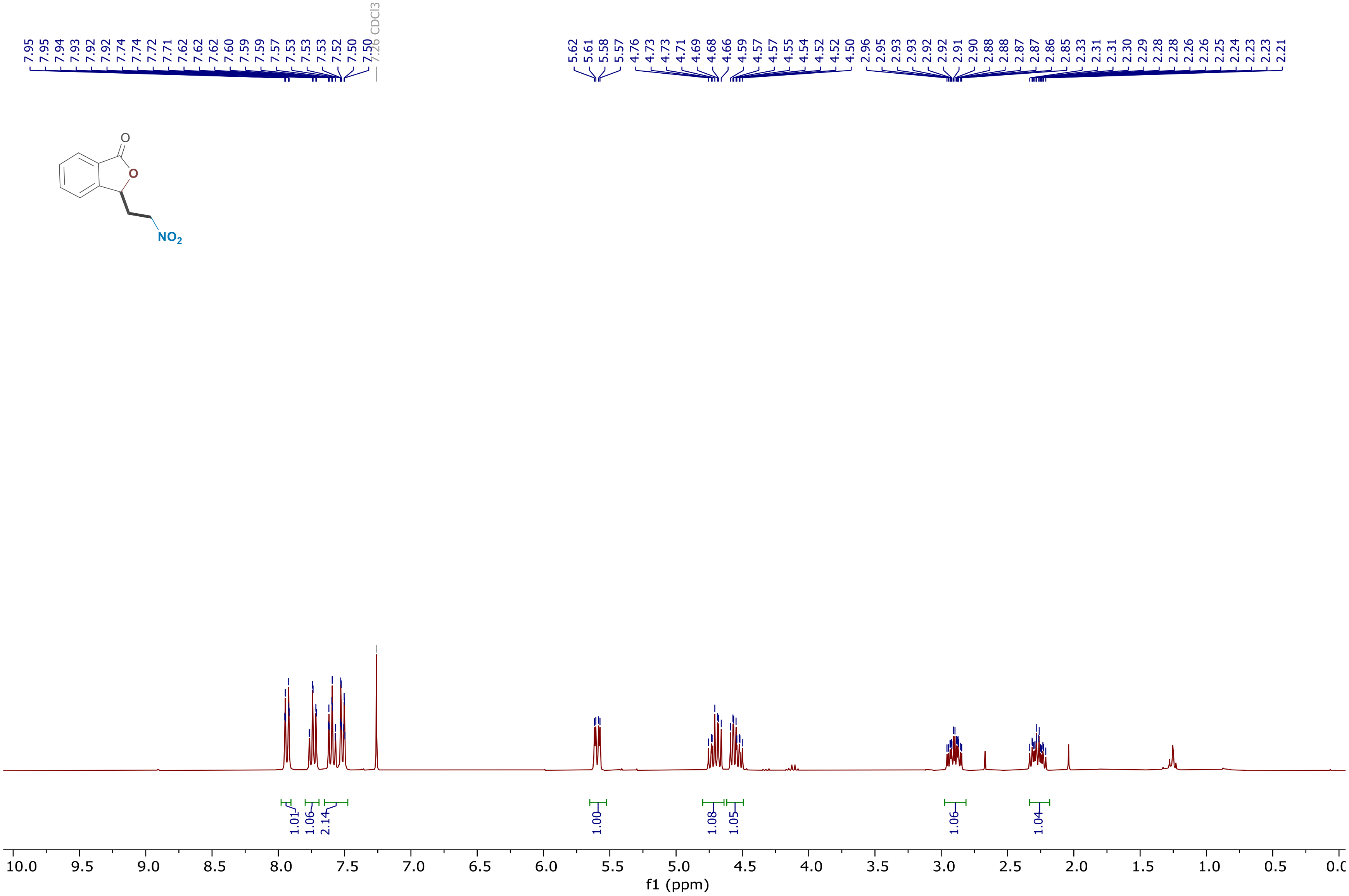

^{13}C NMR (75 MHz, $CDCl_3$) of **17**

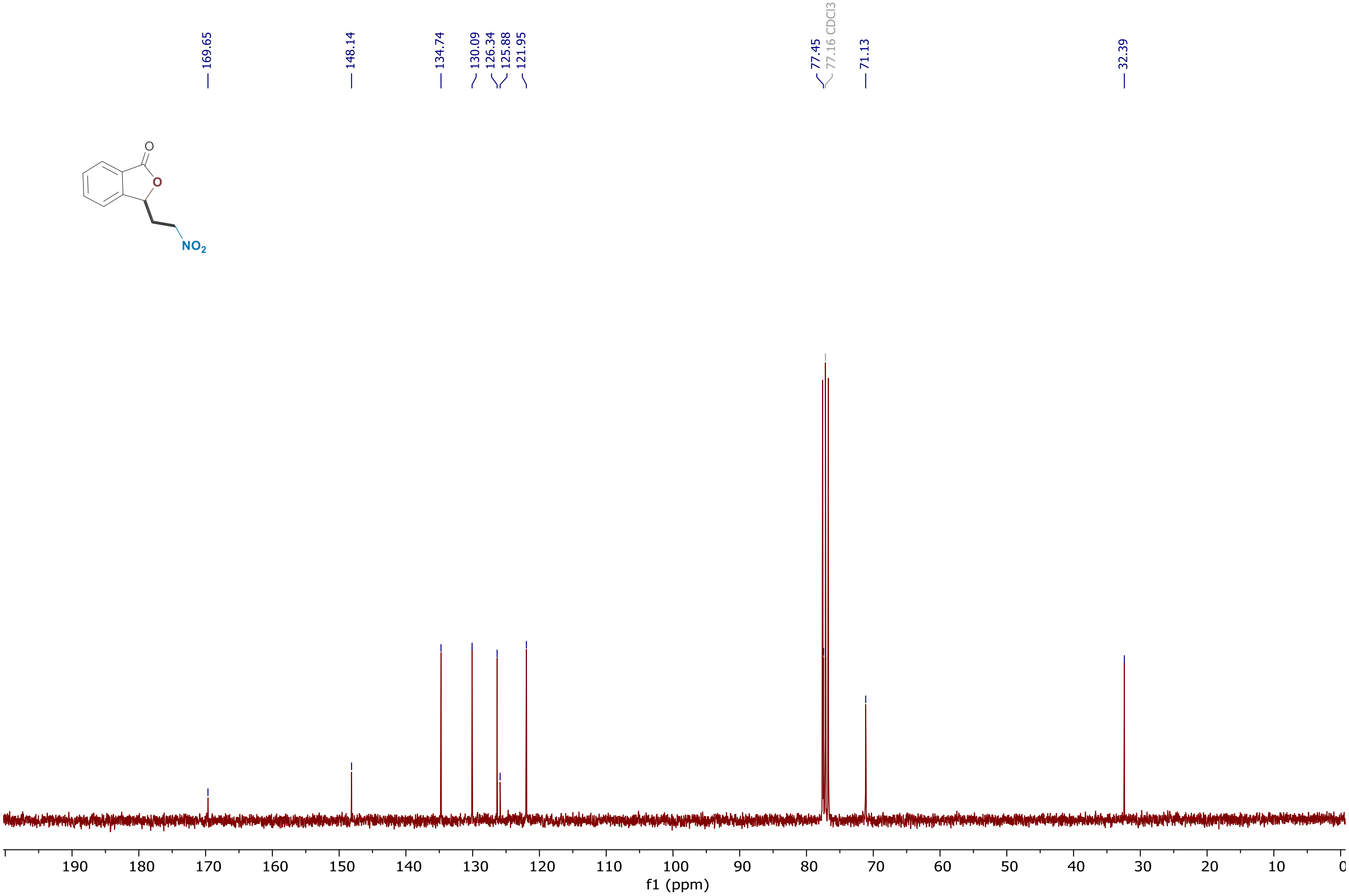

^{1}H NMR (300 MHz, $CDCl_3$) of **18**

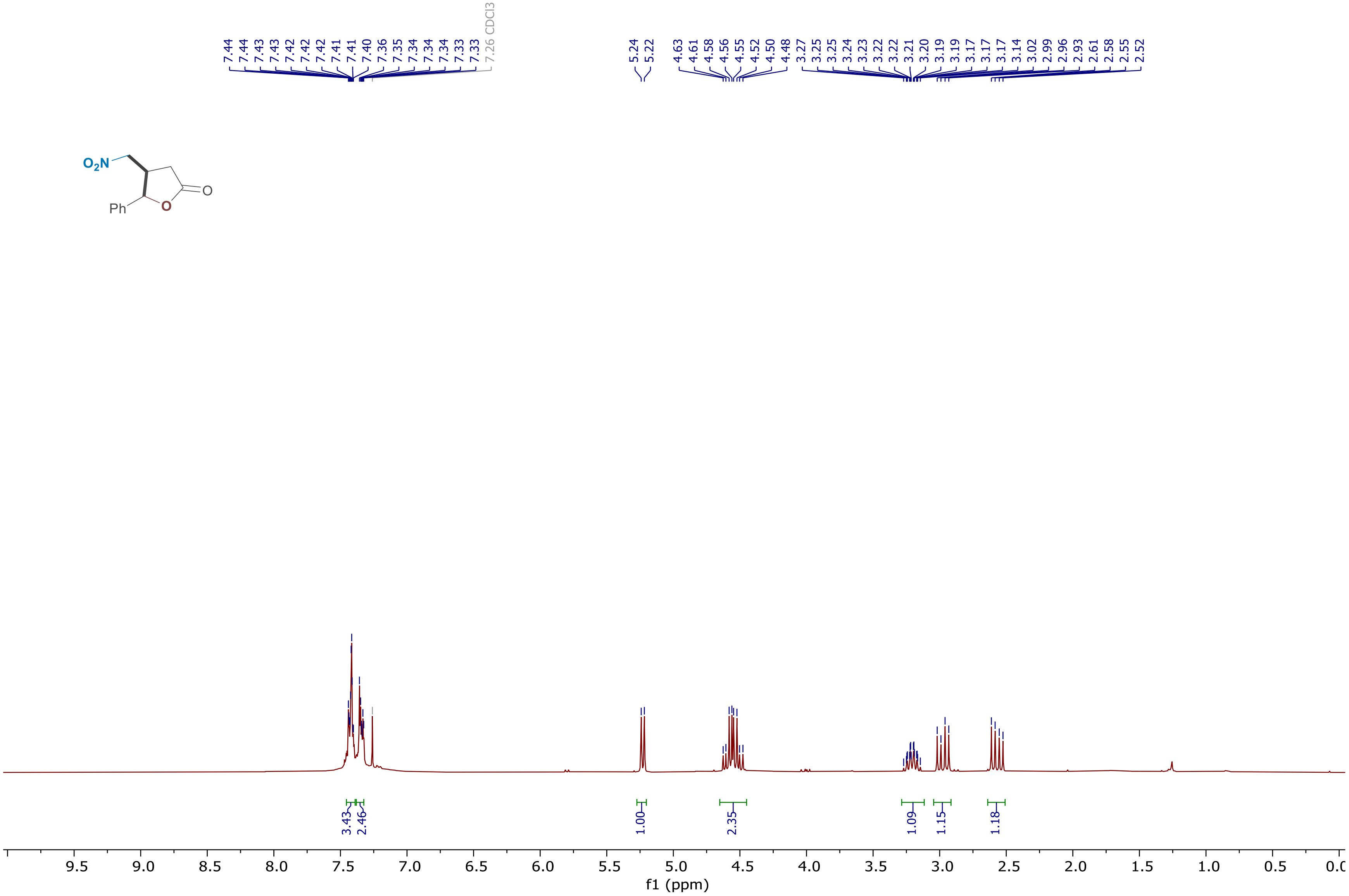

^{13}C NMR (75 MHz, $CDCl_3$) of **18**

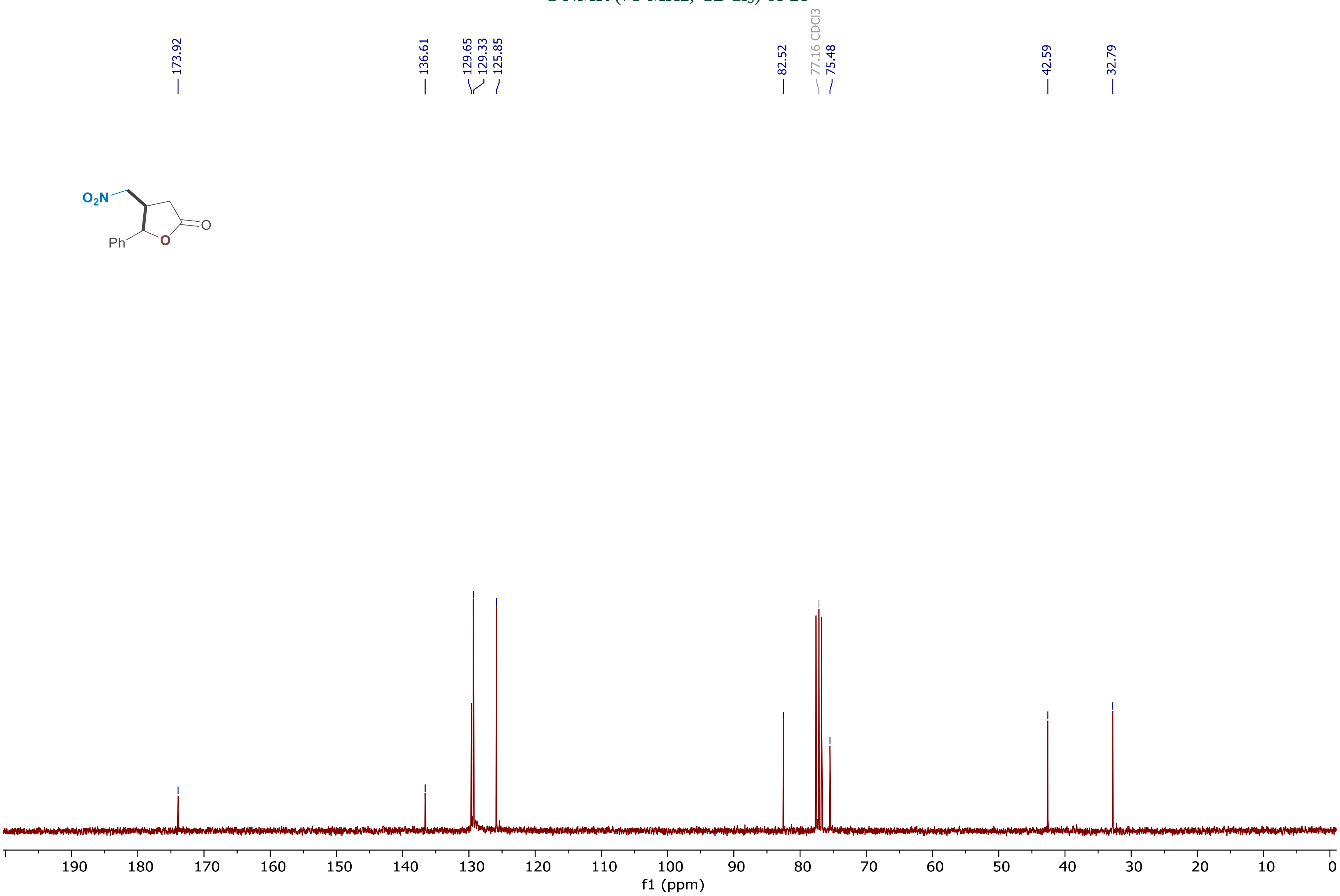

^{1}H NMR (300 MHz, $CDCl_3$) of **19**

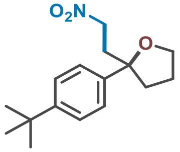

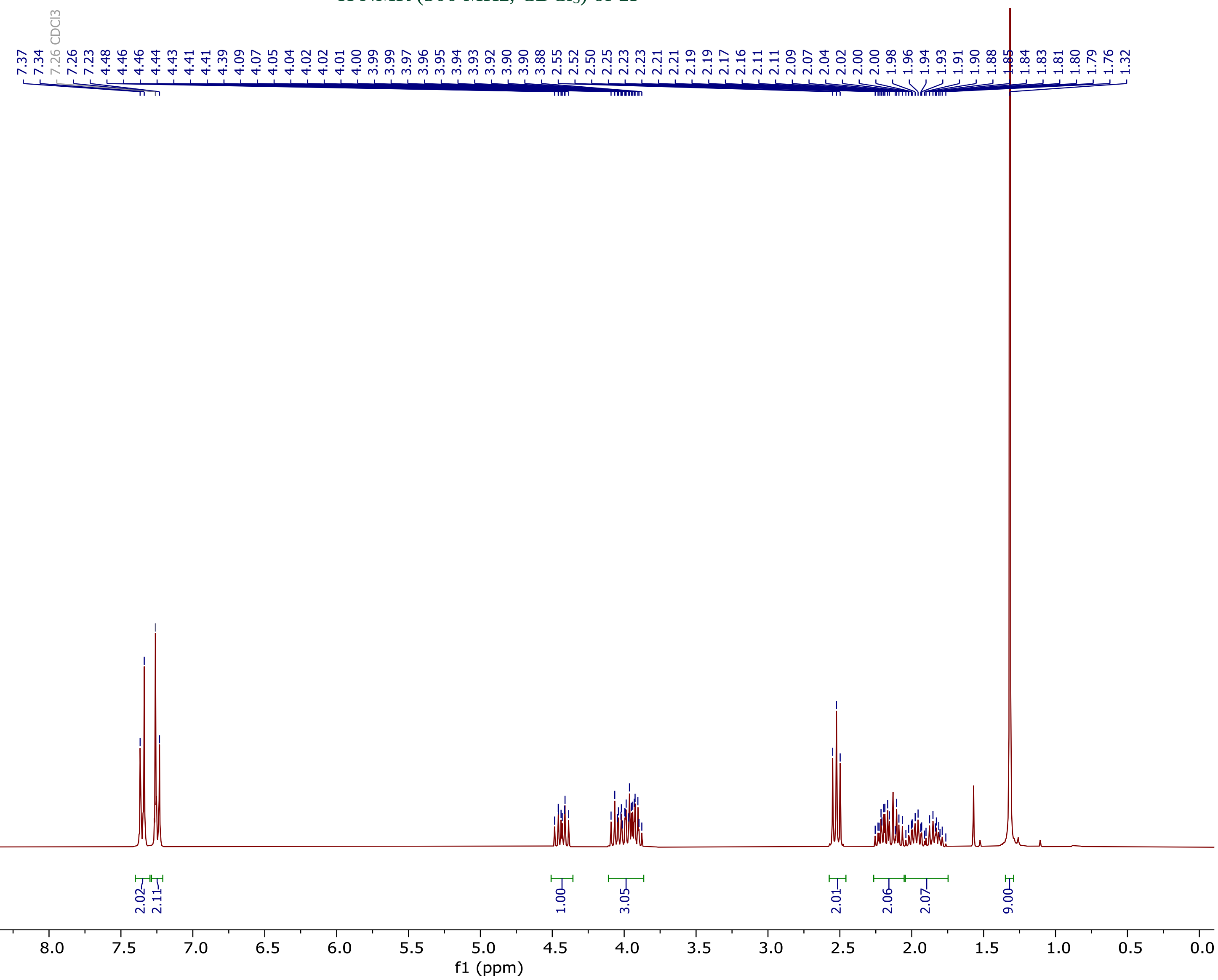

^{13}C NMR (75 MHz, $CDCl_3$) of **19**

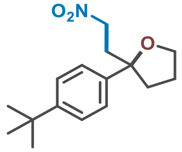

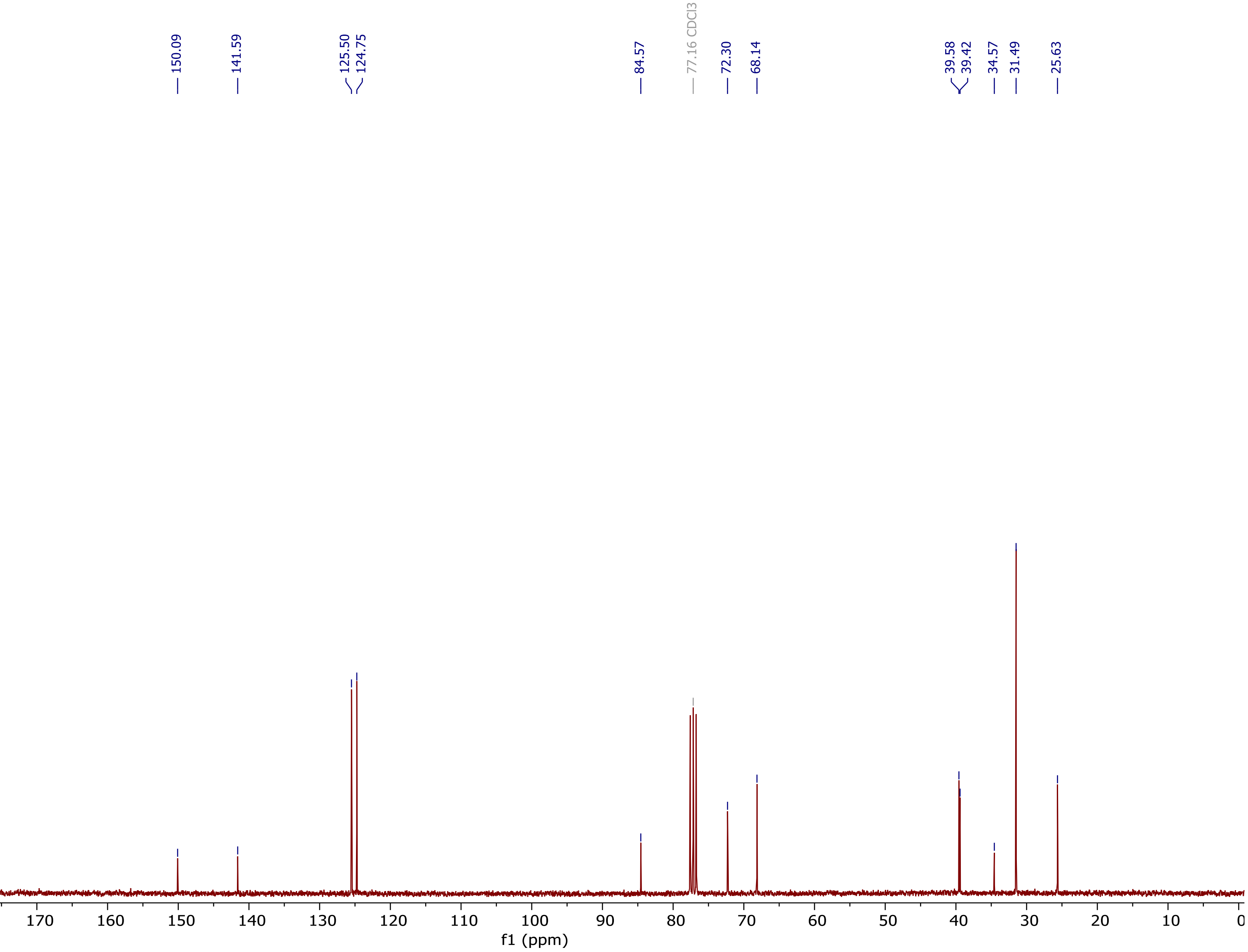

^1H NMR (300 MHz, $CDCl_3$) of **20**

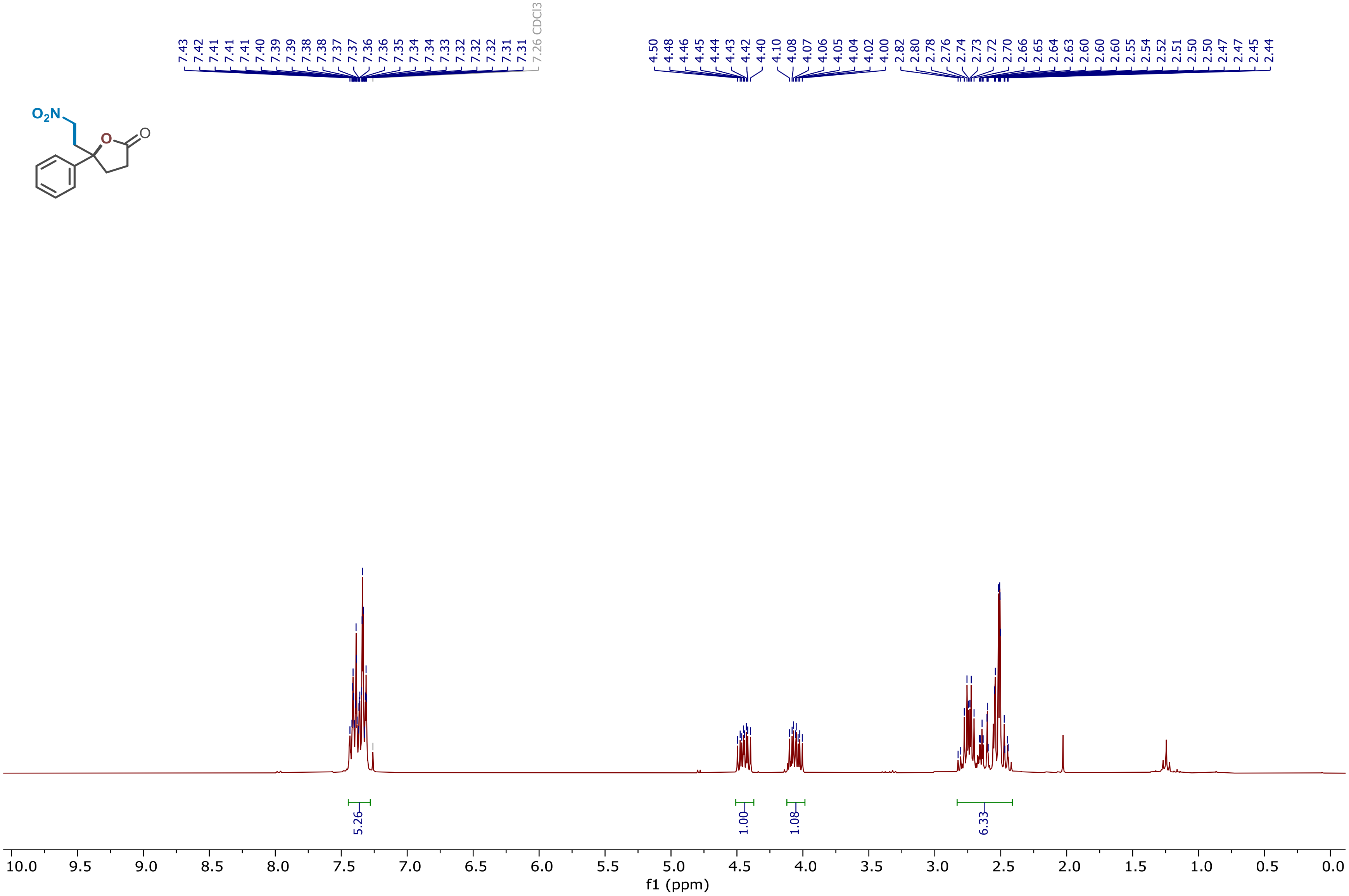

^{13}C NMR (75 MHz, $CDCl_3$) of **20**

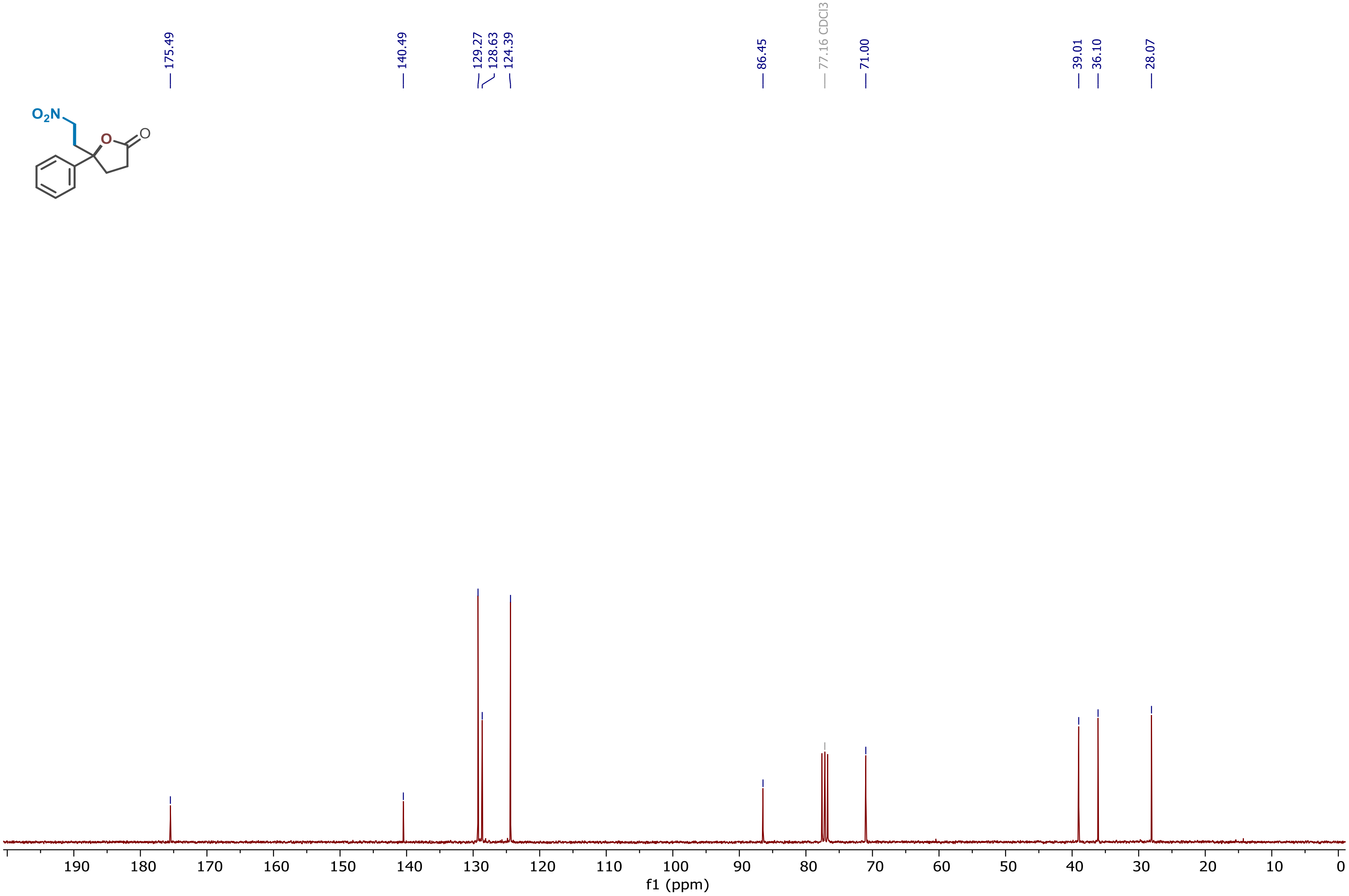

^{1}H NMR (300 MHz, $CDCl_3$) of **21**

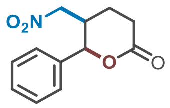

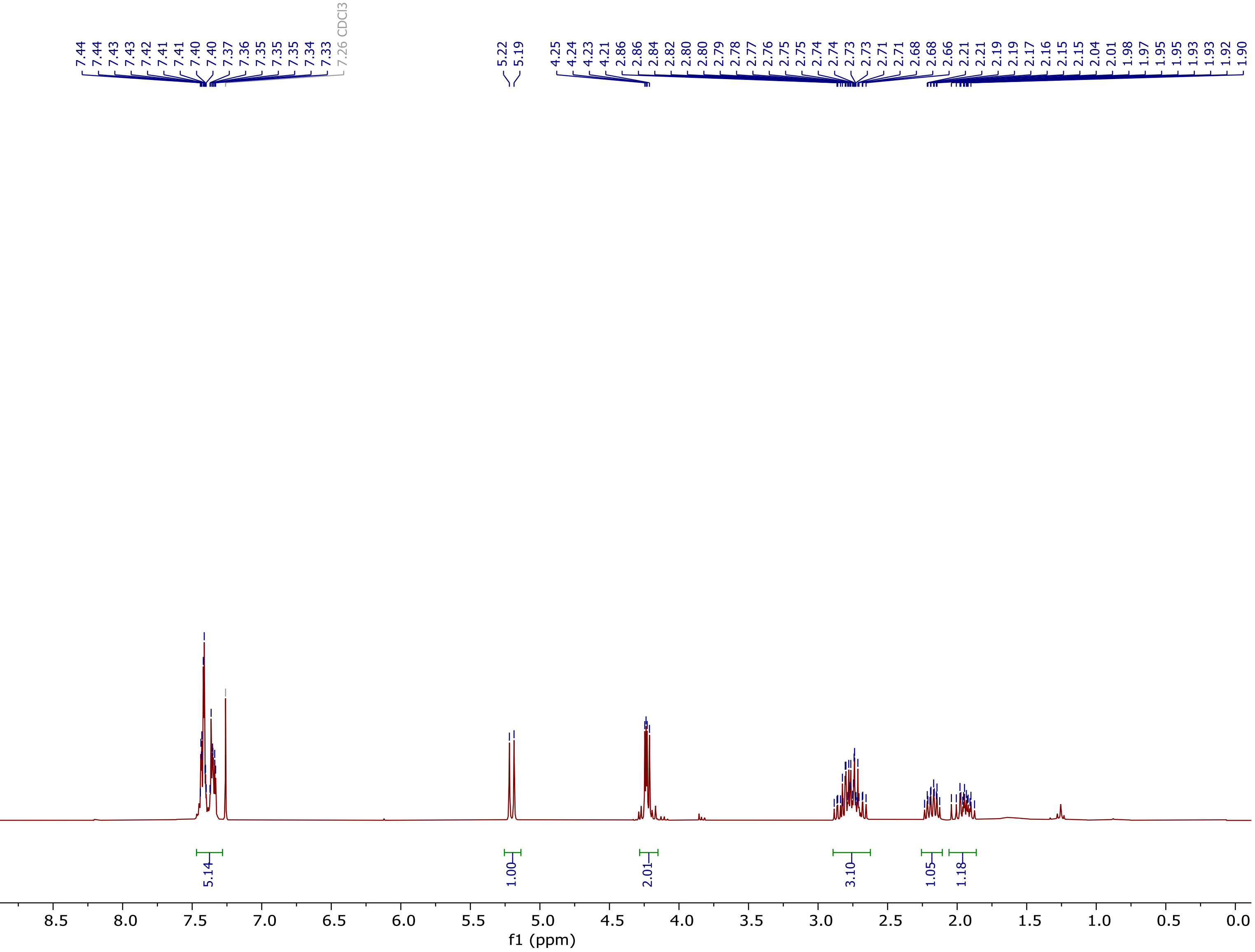

^{13}C NMR (75 MHz, $CDCl_3$) of **21**

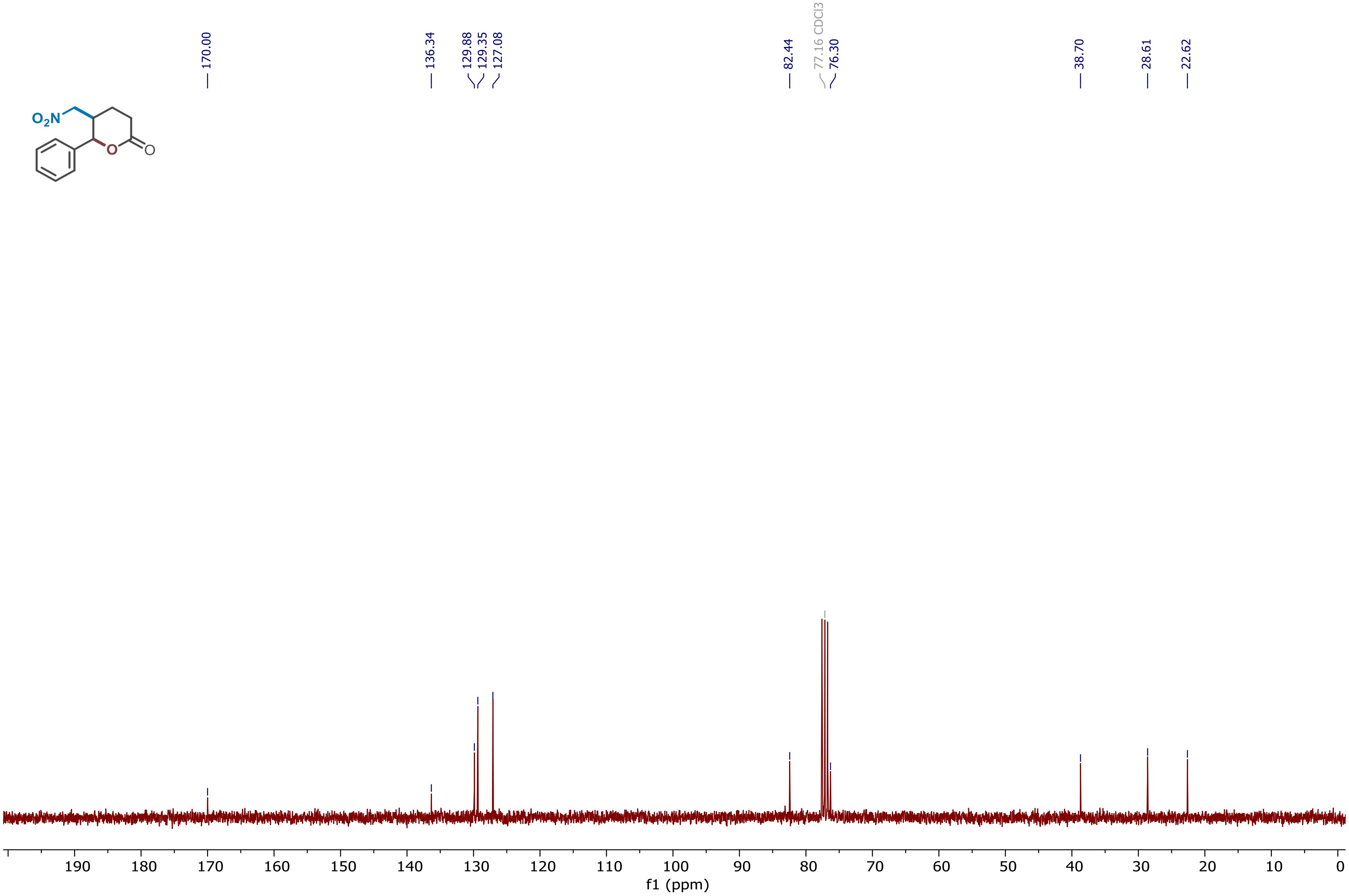

^{1}H NMR (300 MHz, $CDCl_3$) of **22**

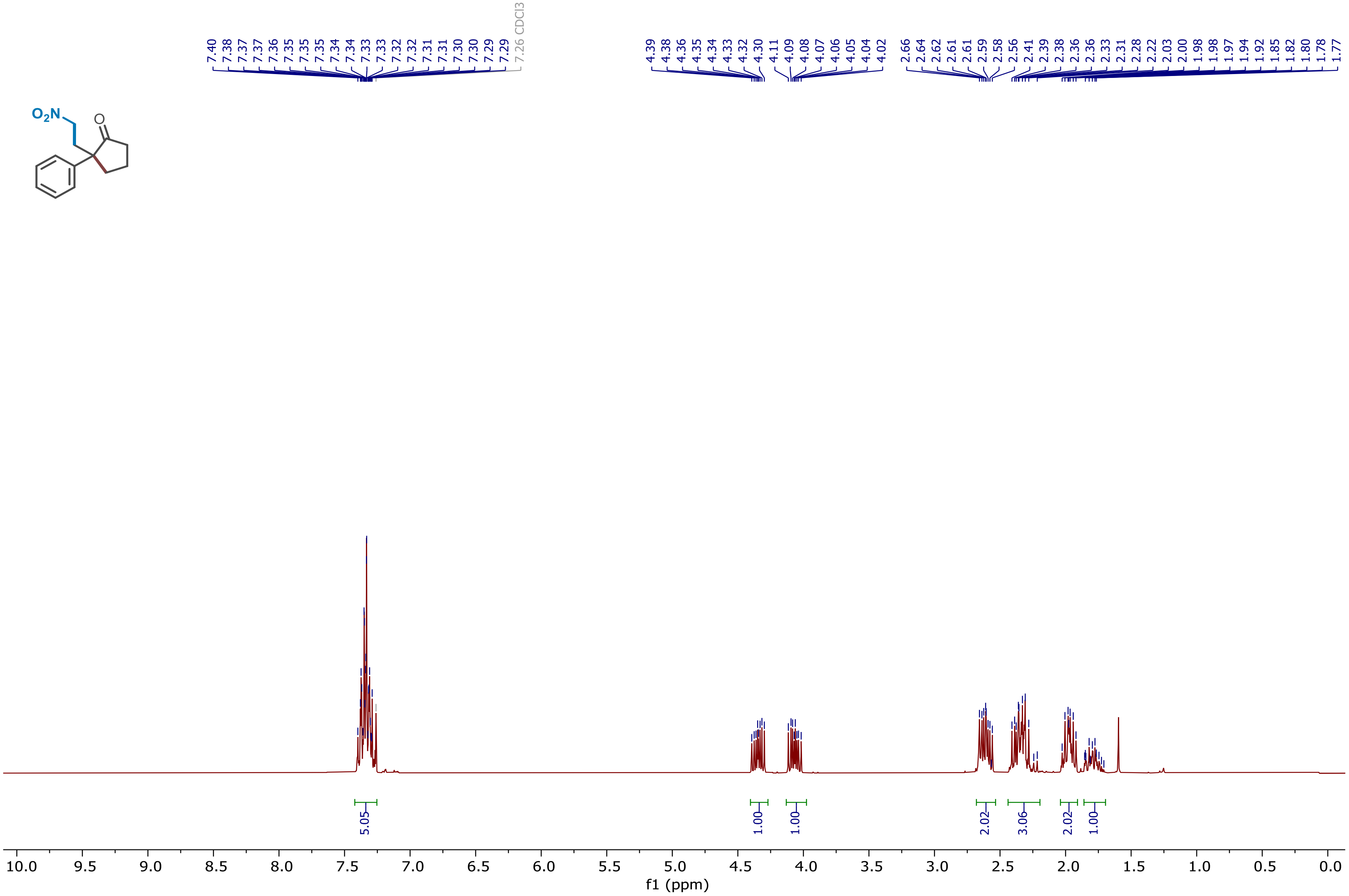

^{13}C NMR (75 MHz, $CDCl_3$) of **22**

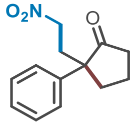

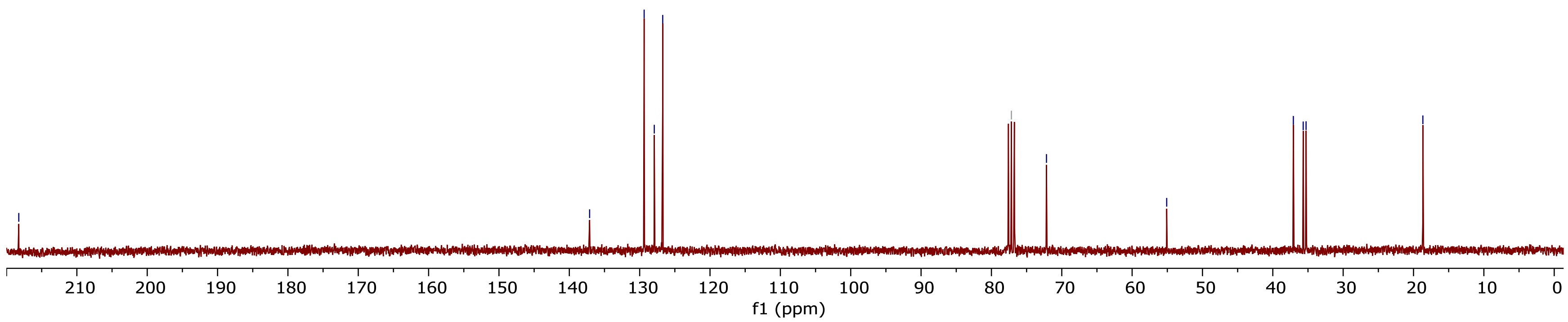

^{1}H NMR (300 MHz, $CDCl_3$) of **23a**

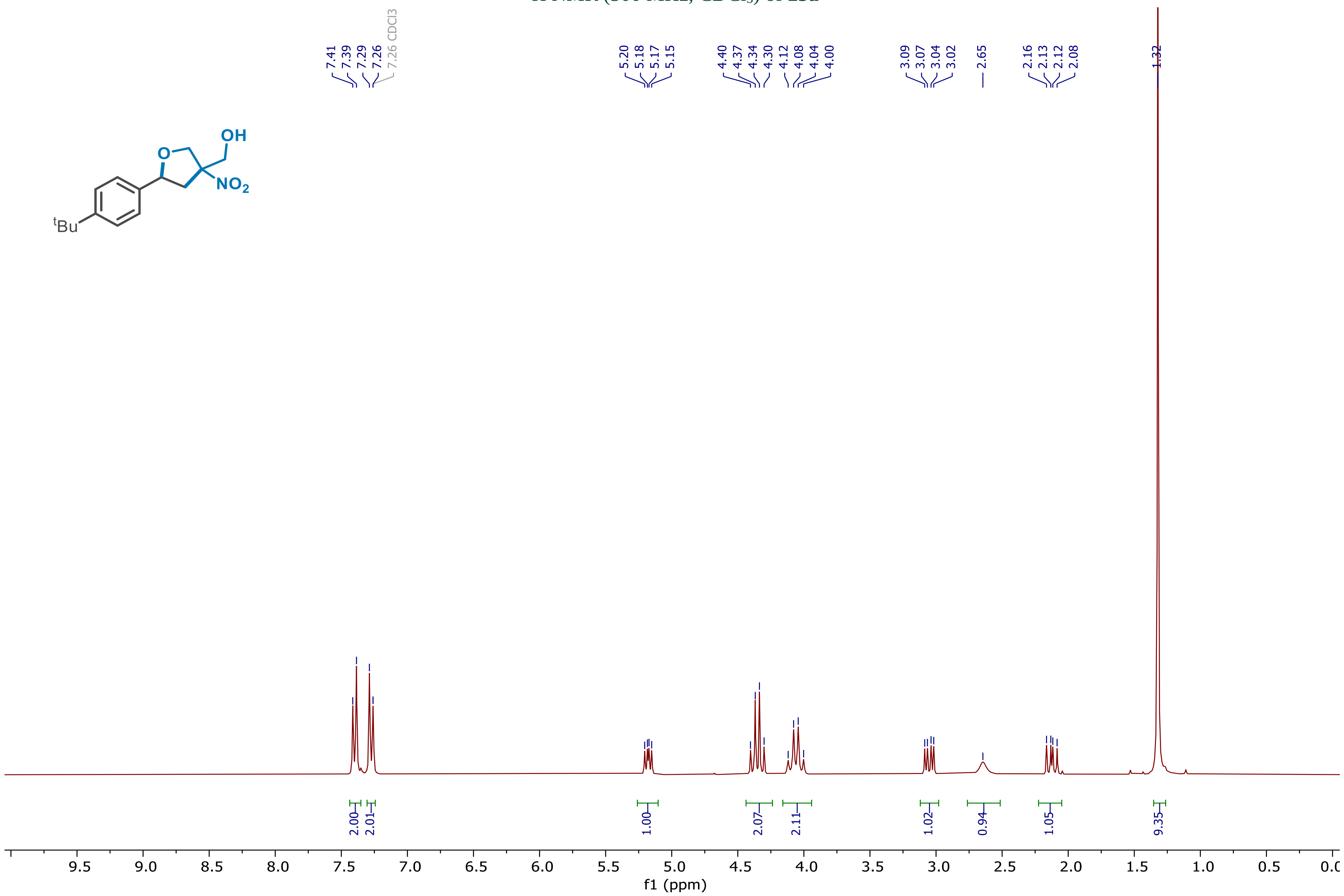

^{13}C NMR (75 MHz, $CDCl_3$) of **23a**

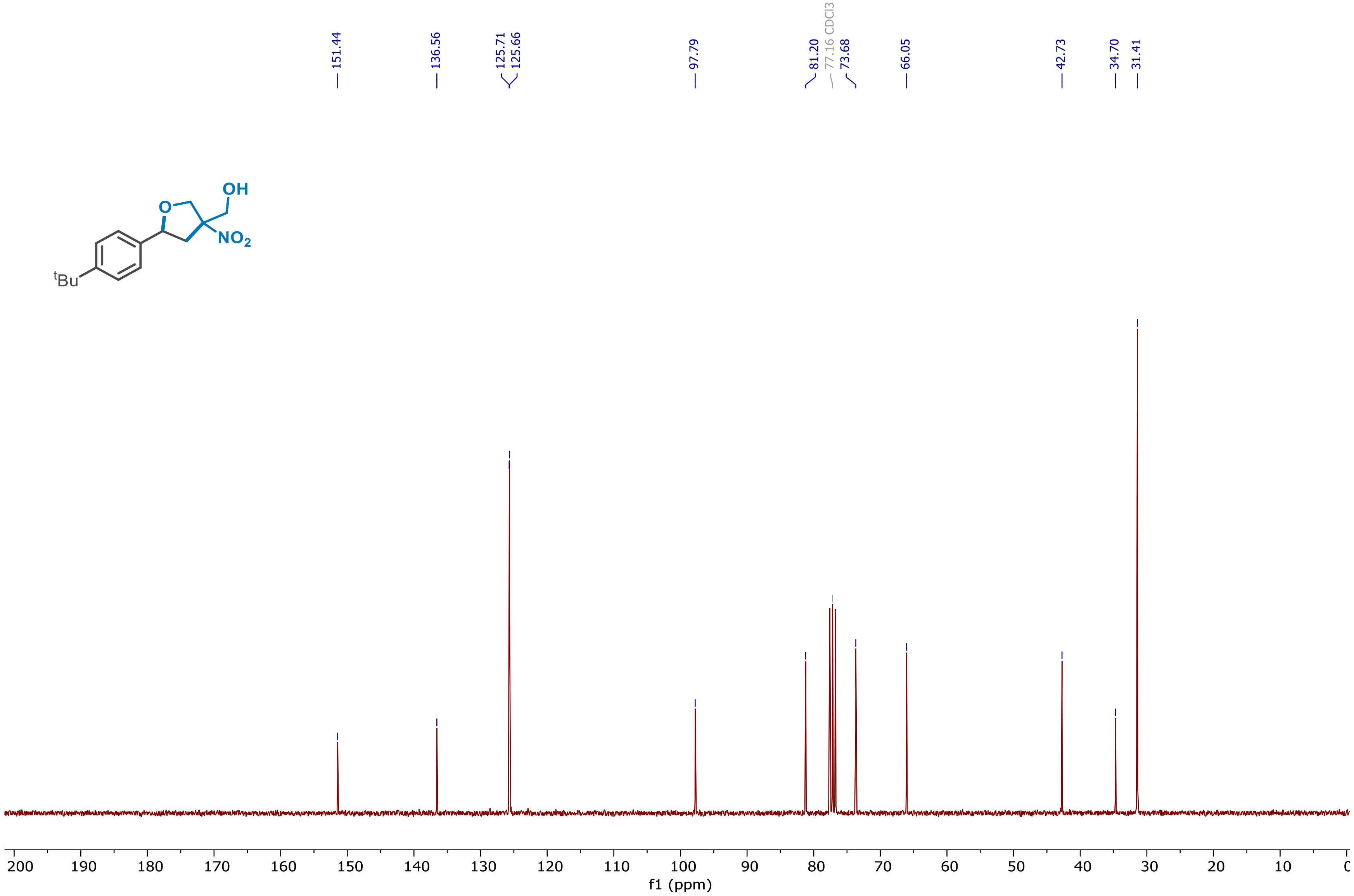

^{1}H NMR (300 MHz, $CDCl_3$) of **23b**

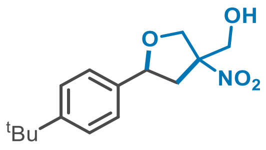

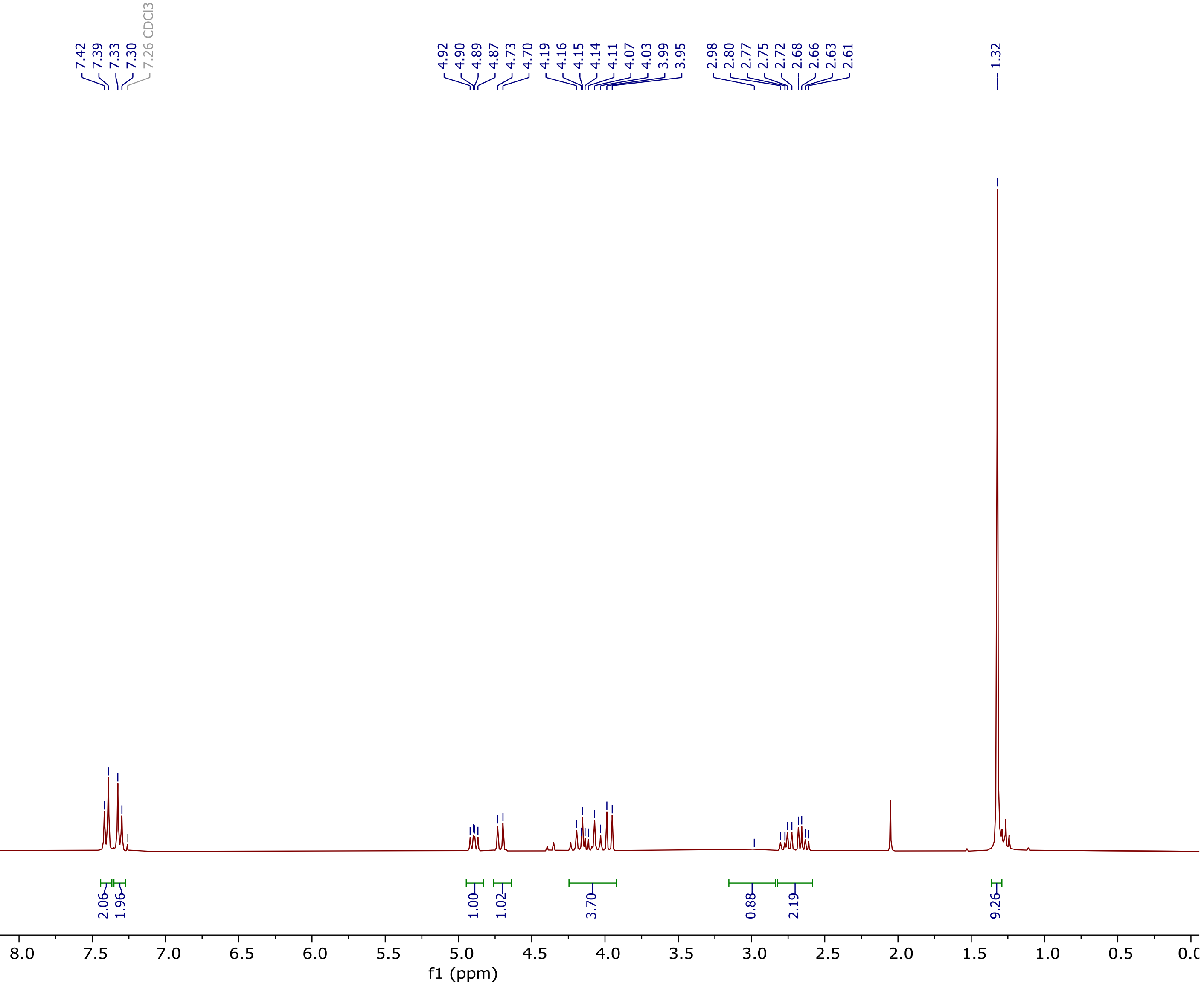

^{13}C NMR (75 MHz, $CDCl_3$) of **23b**

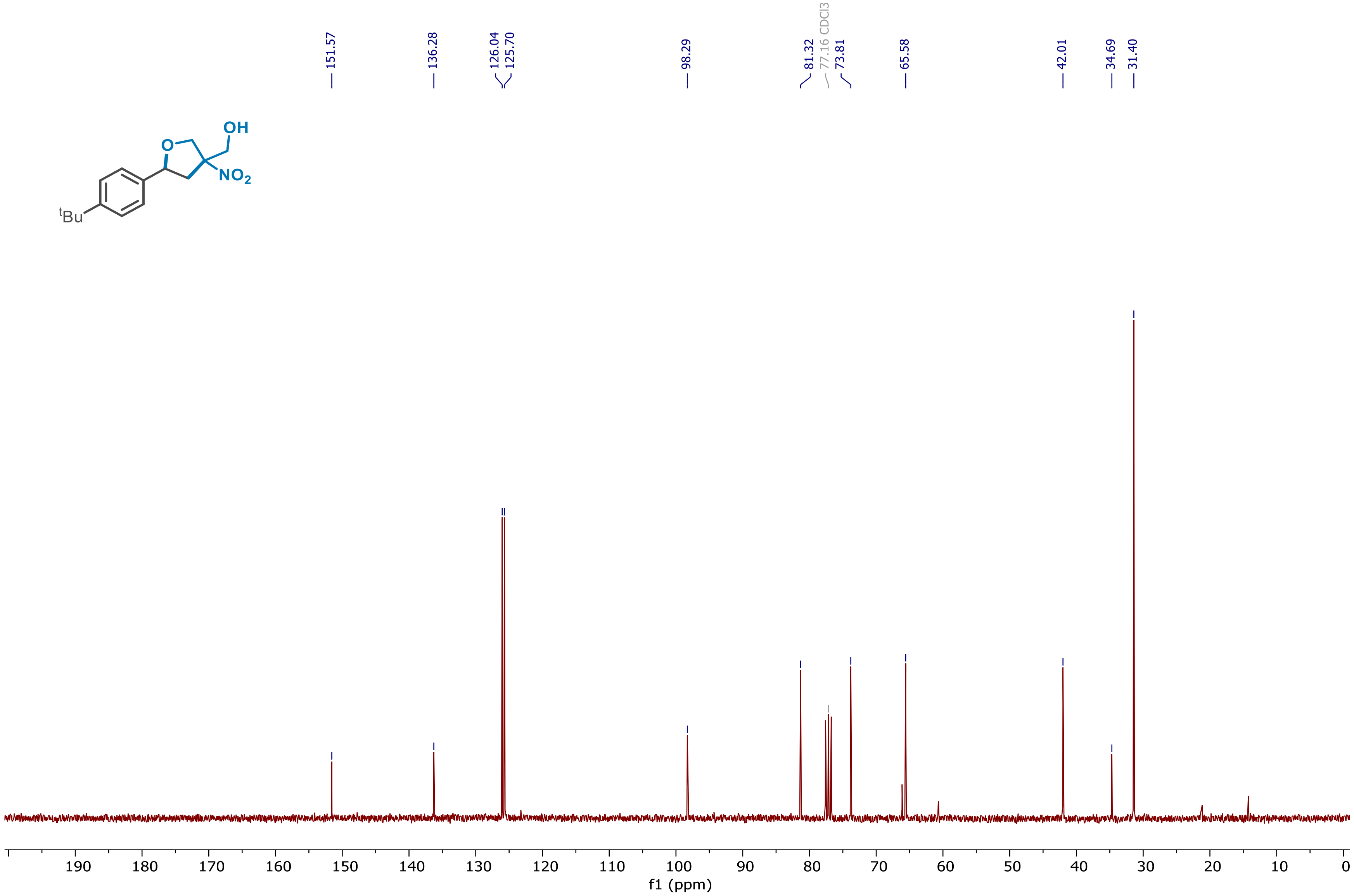

^{1}H NMR (300 MHz, CDCl$_3$) of **24a**

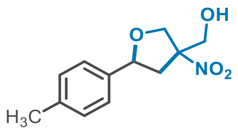

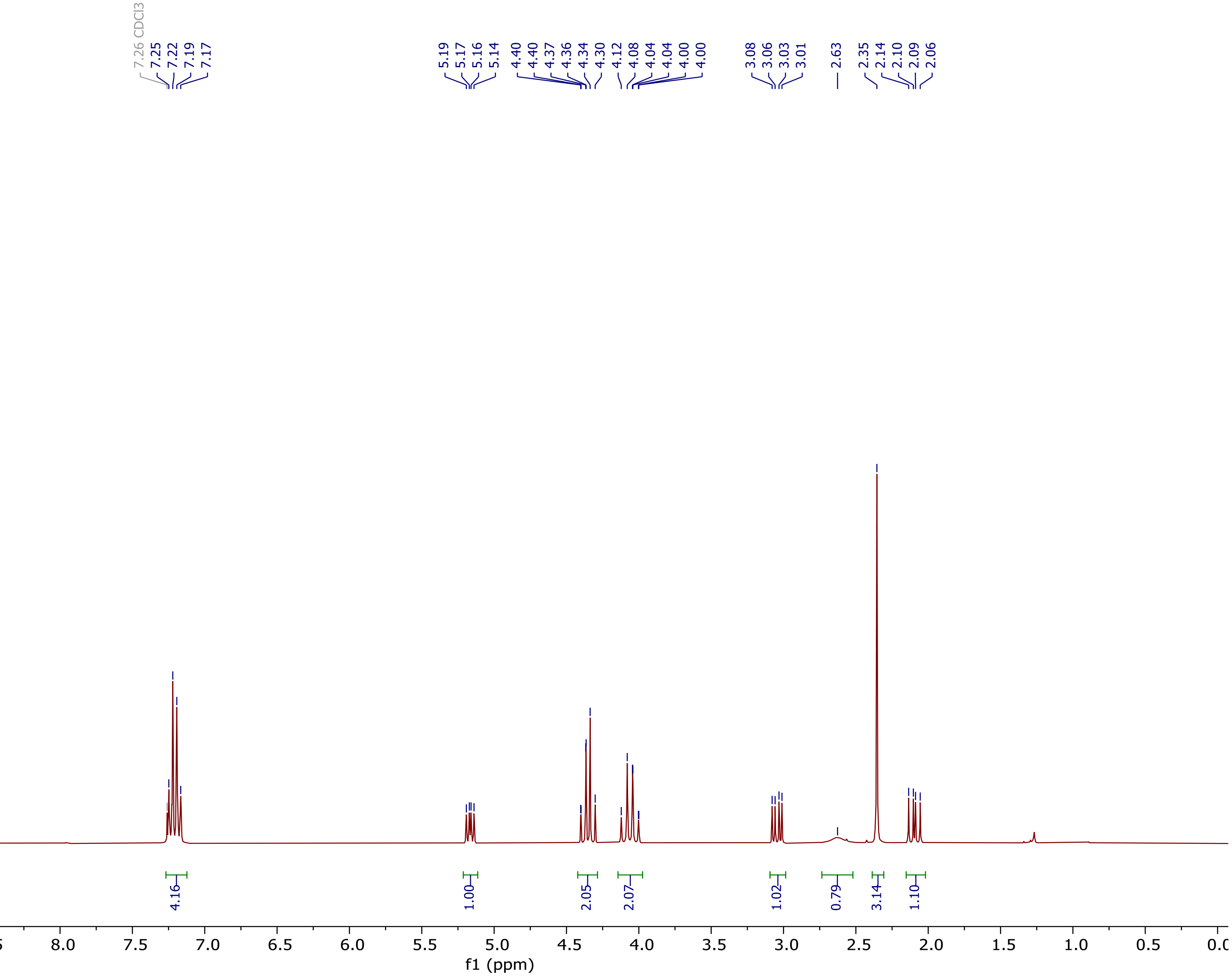

^{13}C NMR (75 MHz, $CDCl_3$) of **24a**

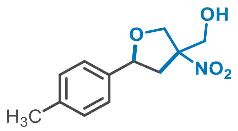

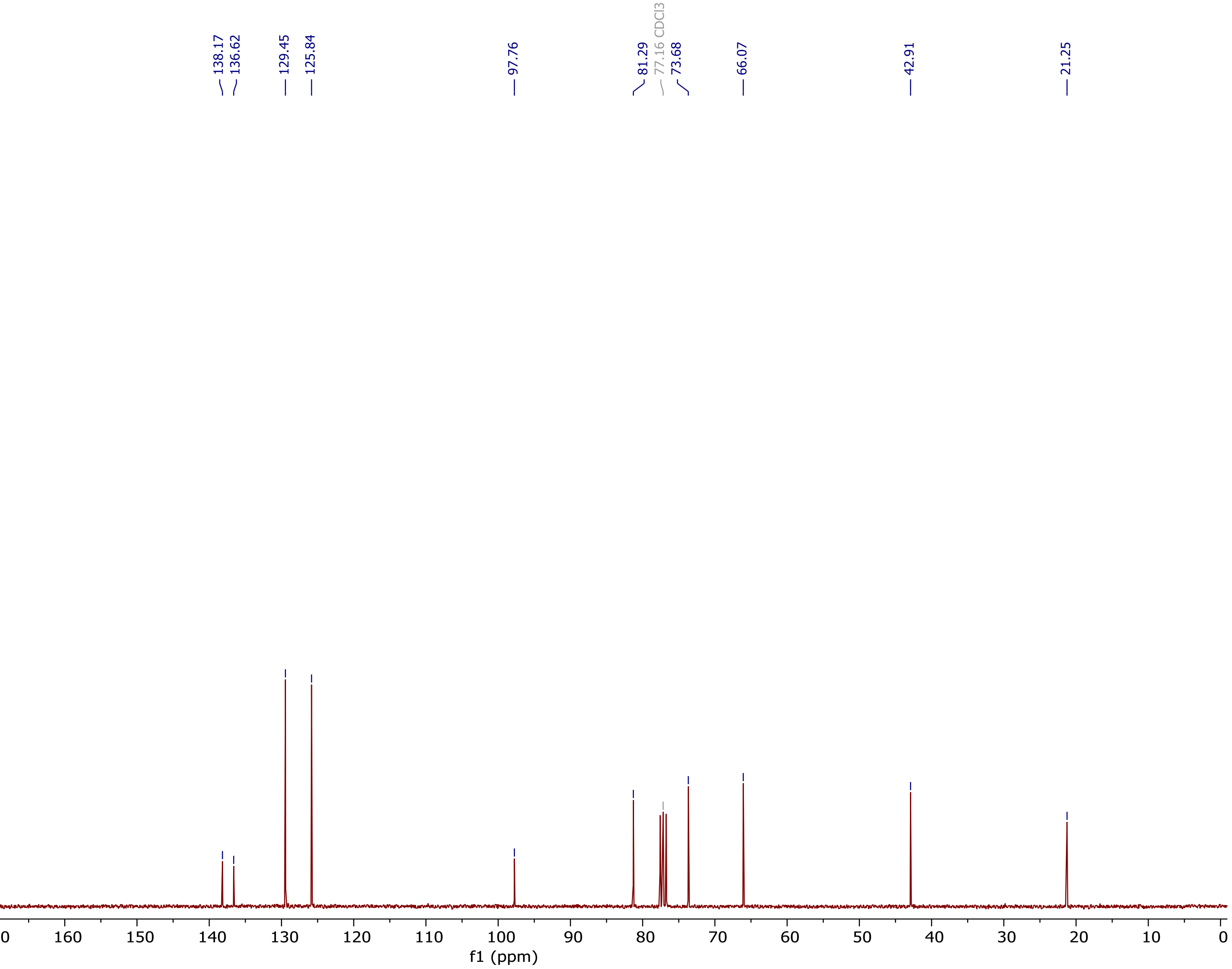

^{1}H NMR (300 MHz, CDCl$_3$) of **24b**

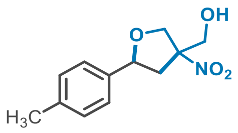

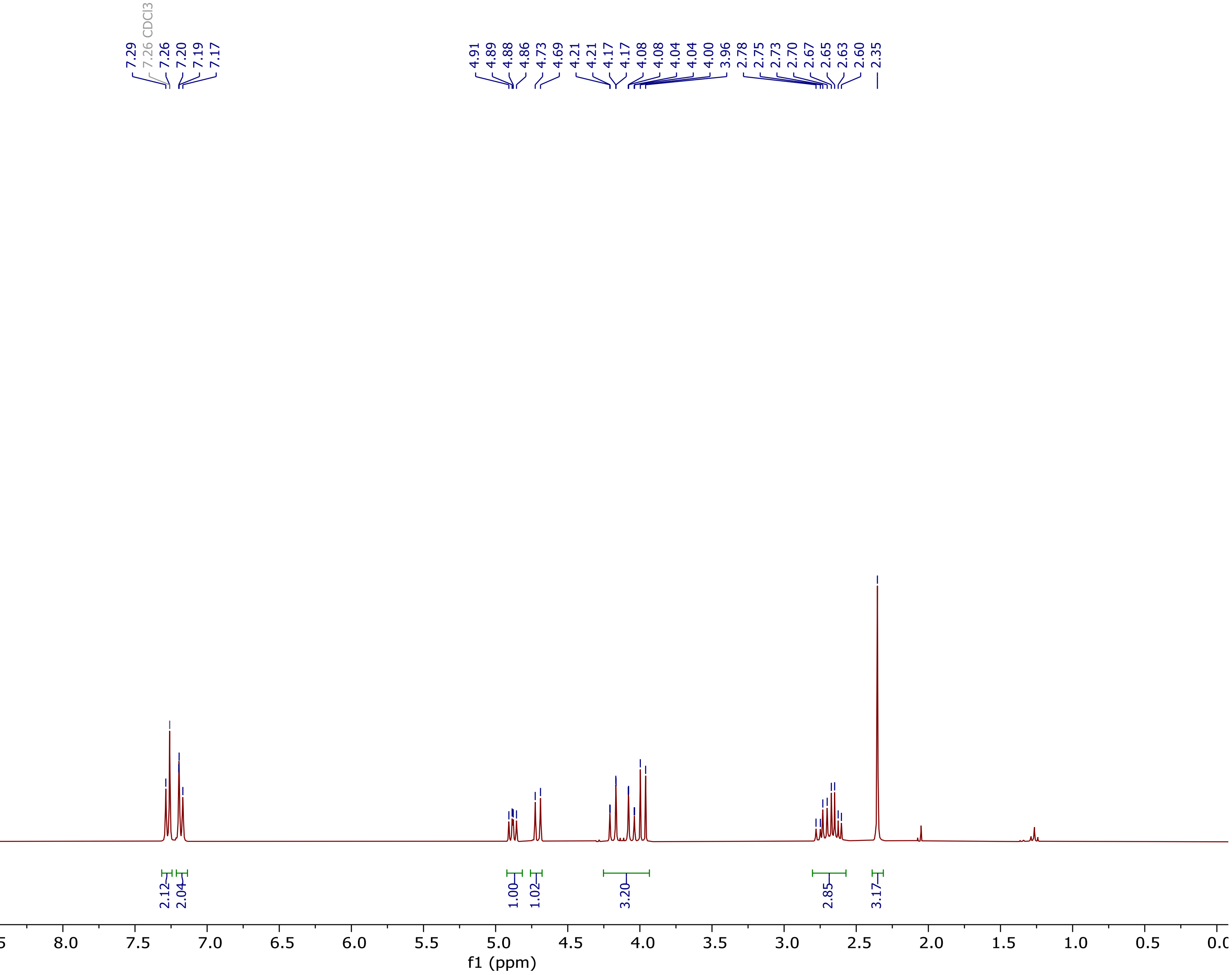

^{13}C NMR (75 MHz, CDCl$_3$) of **24b**

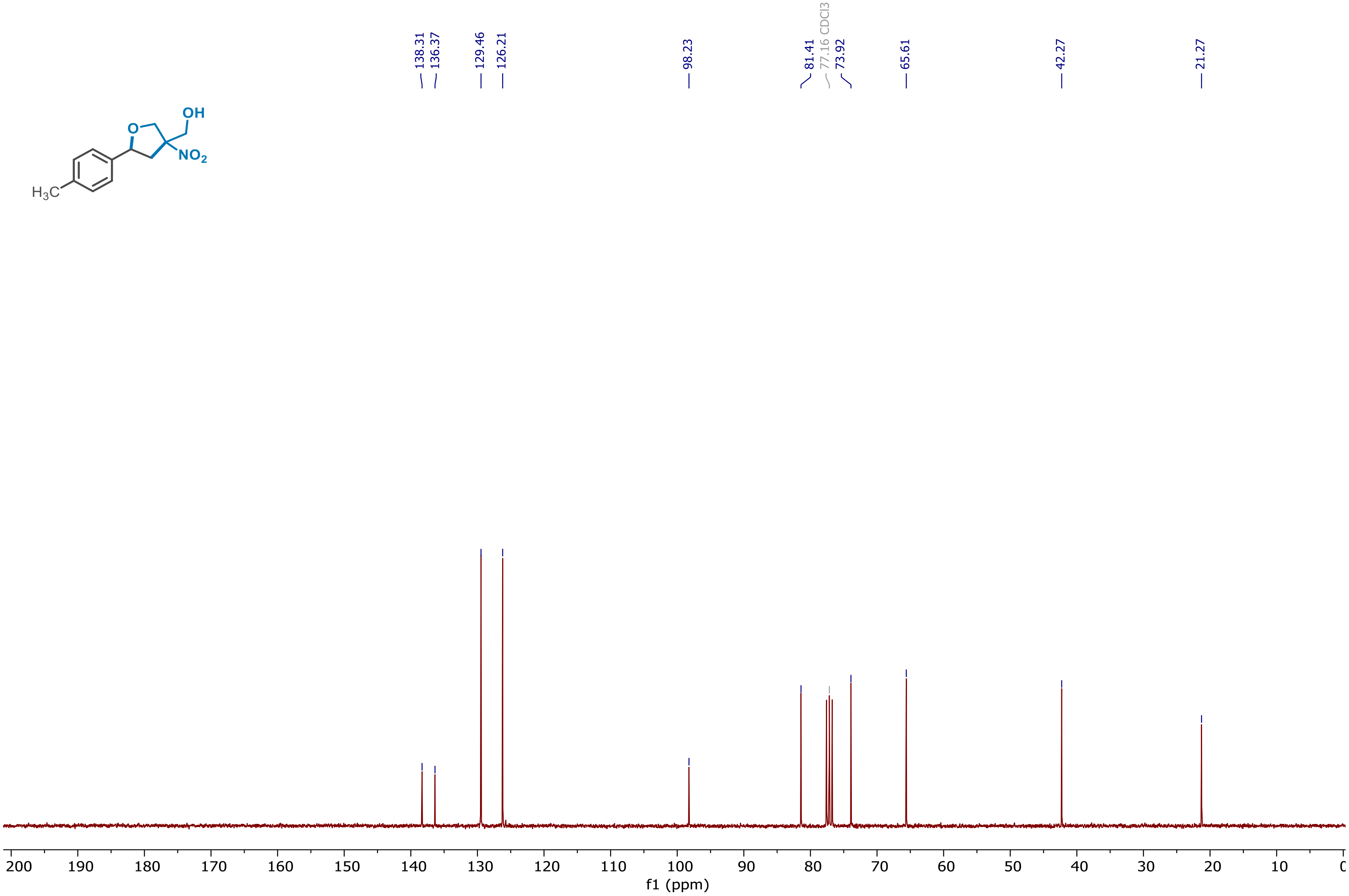

^{1}H NMR (300 MHz, CDCl$_3$) of **25**

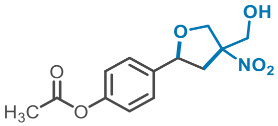

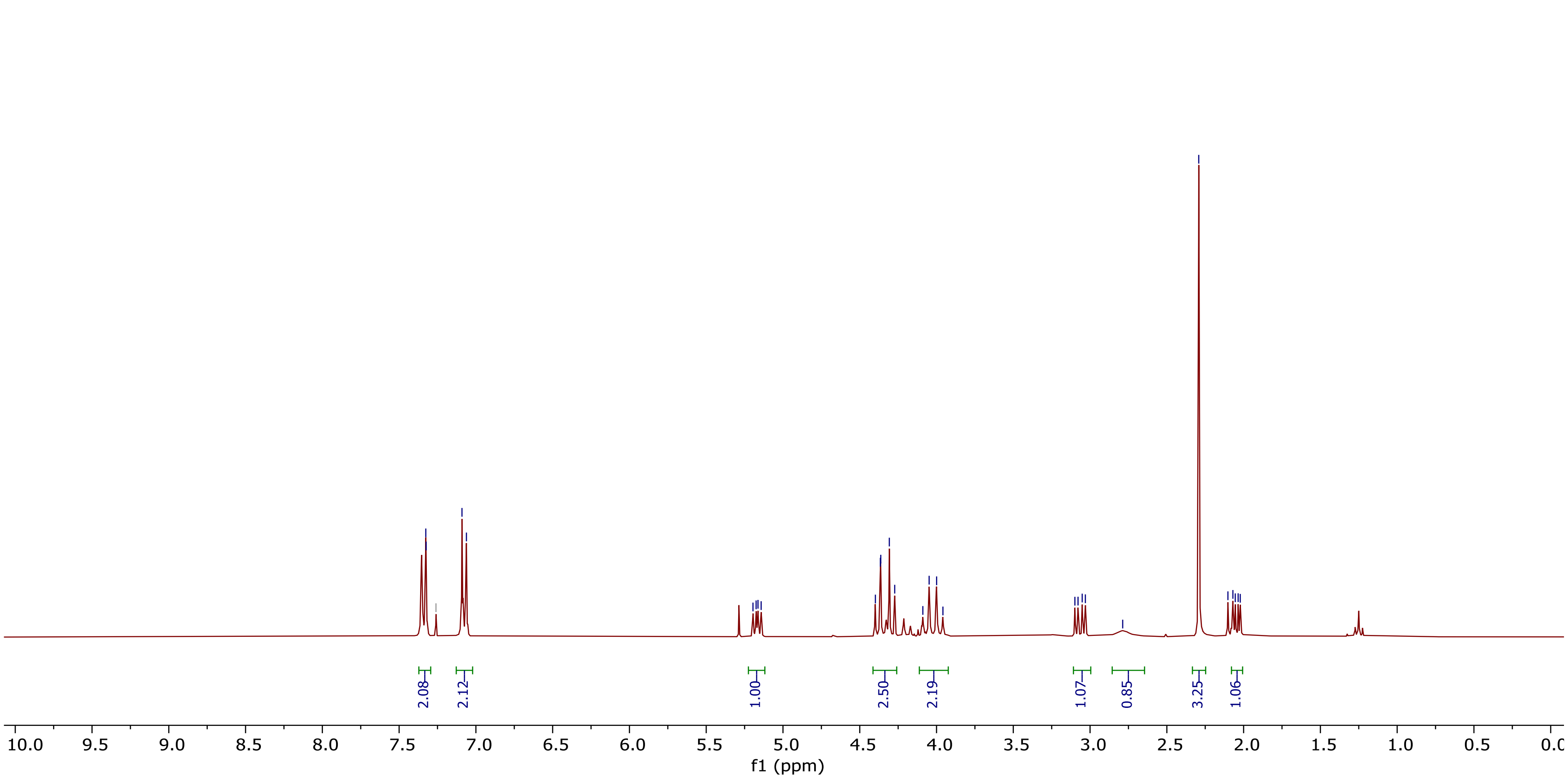

^{13}C NMR (75 MHz, $CDCl_3$) of **25**

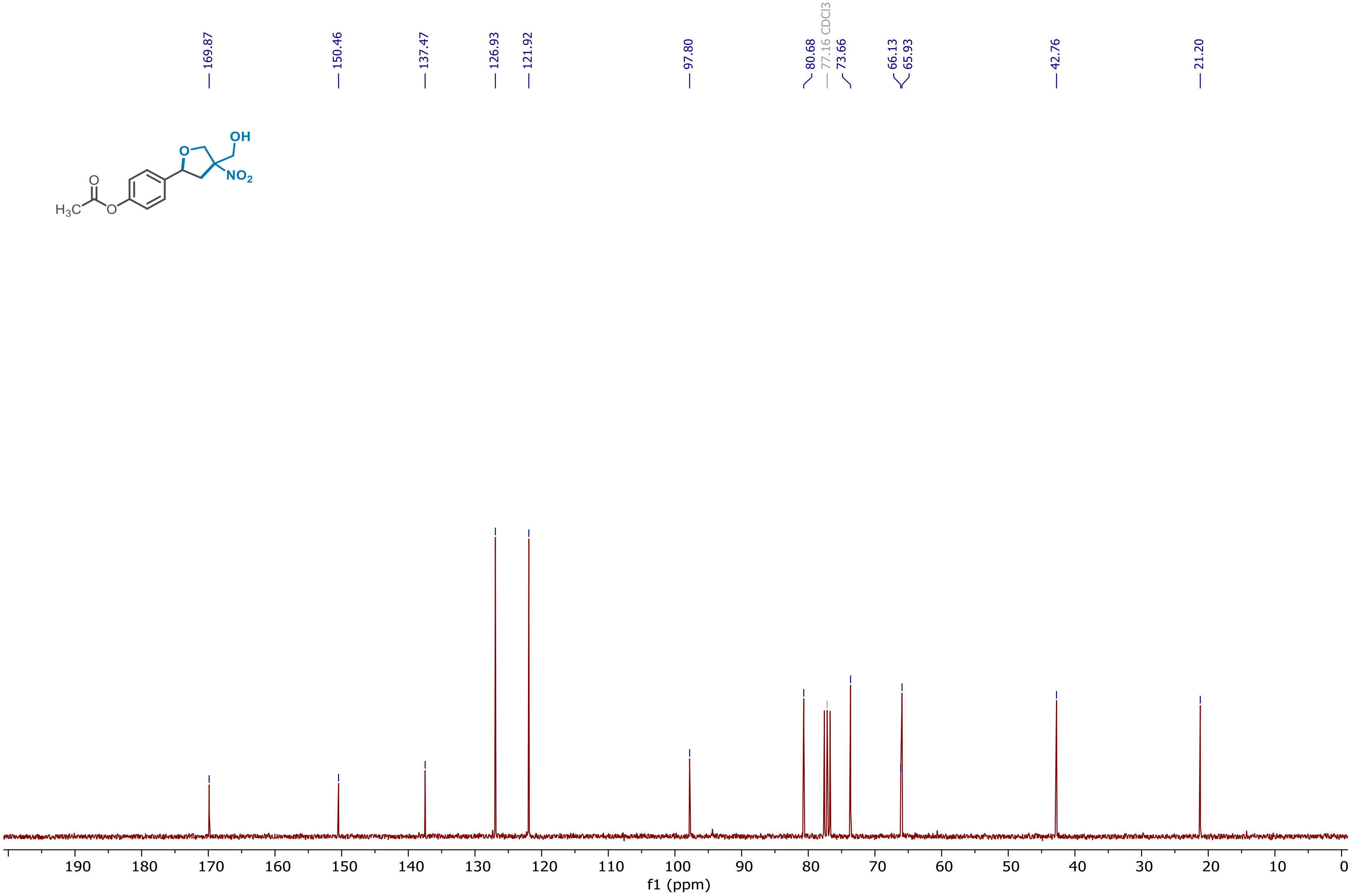

^{1}H NMR (300 MHz, $CDCl_3$) of **26a**

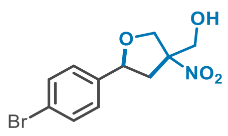

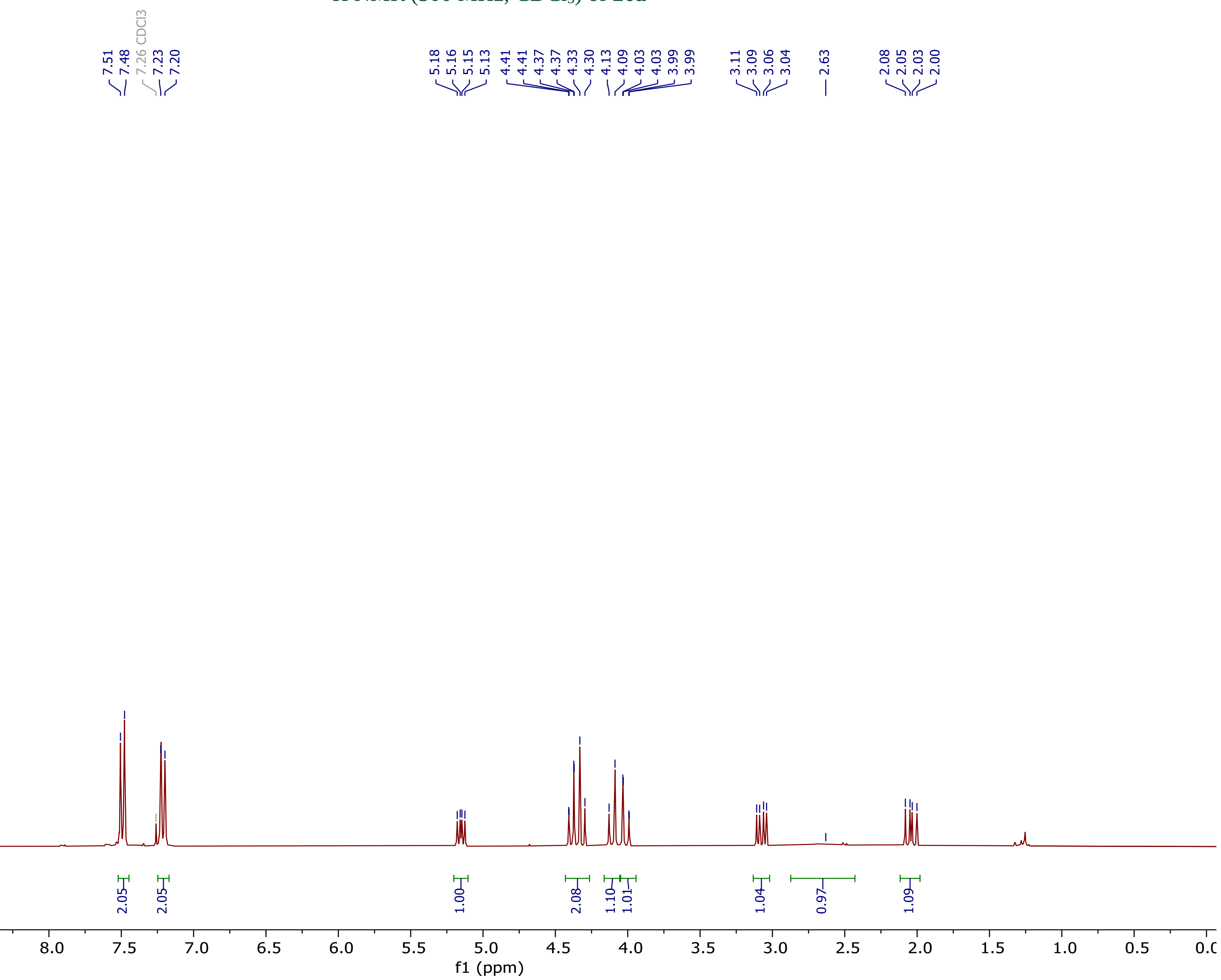

^{13}C NMR (75 MHz, $CDCl_3$) of **26a**

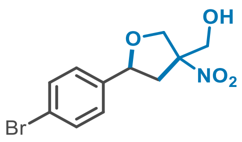

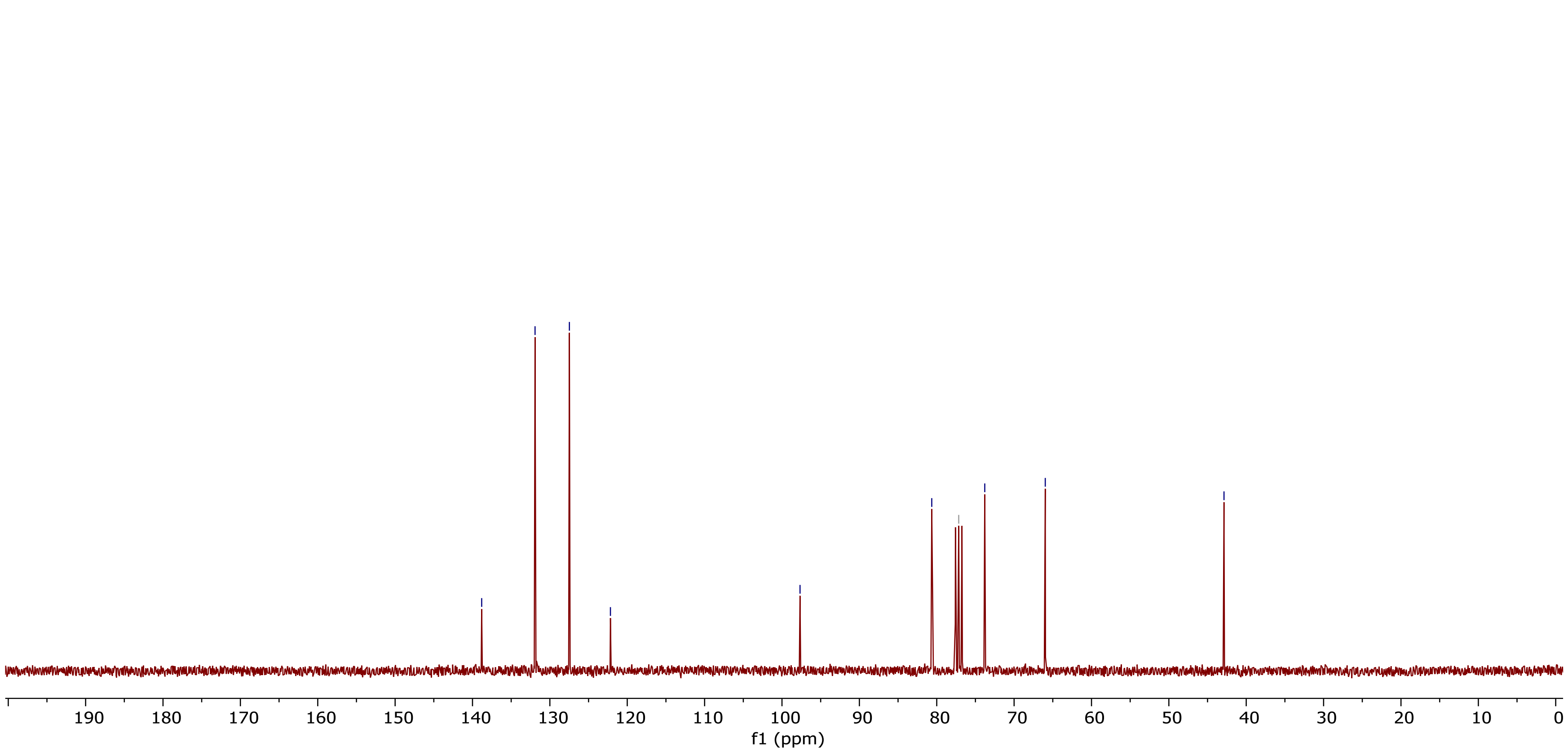

^{1}H NMR (300 MHz, $CDCl_3$) of **26b**

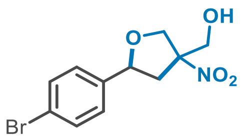

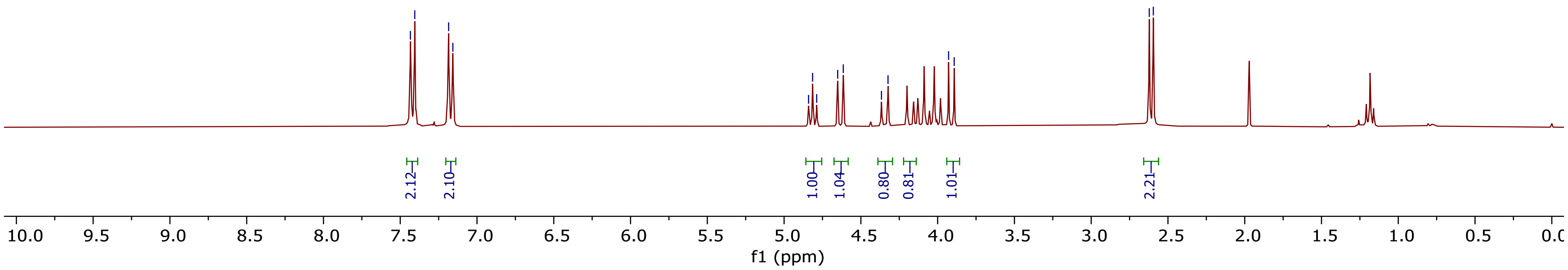

^{13}C NMR (75 MHz, $CDCl_3$) of **26b**

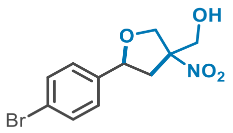

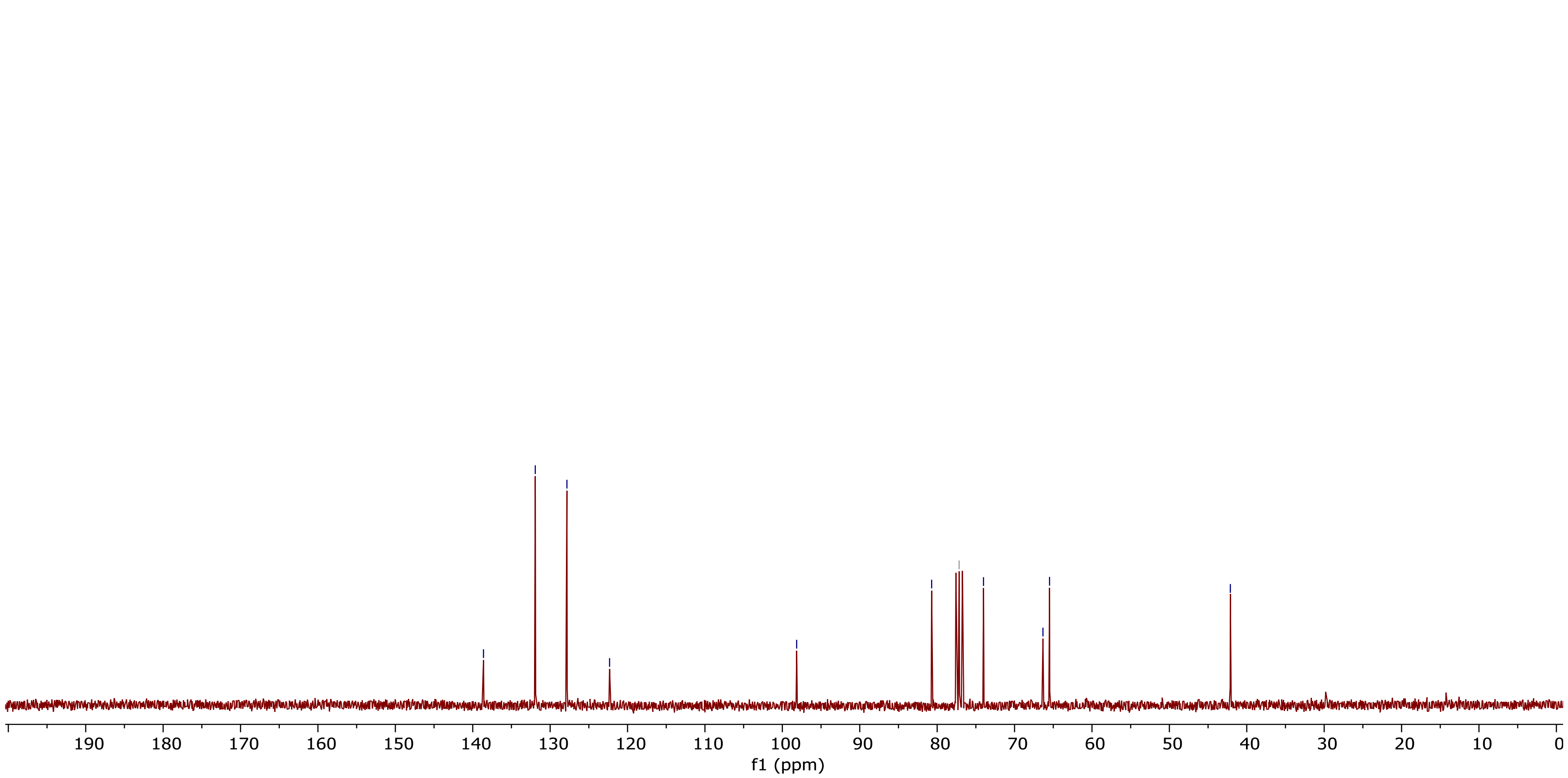

^{1}H NMR (300 MHz, $CDCl_3$) of **27a**

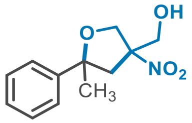

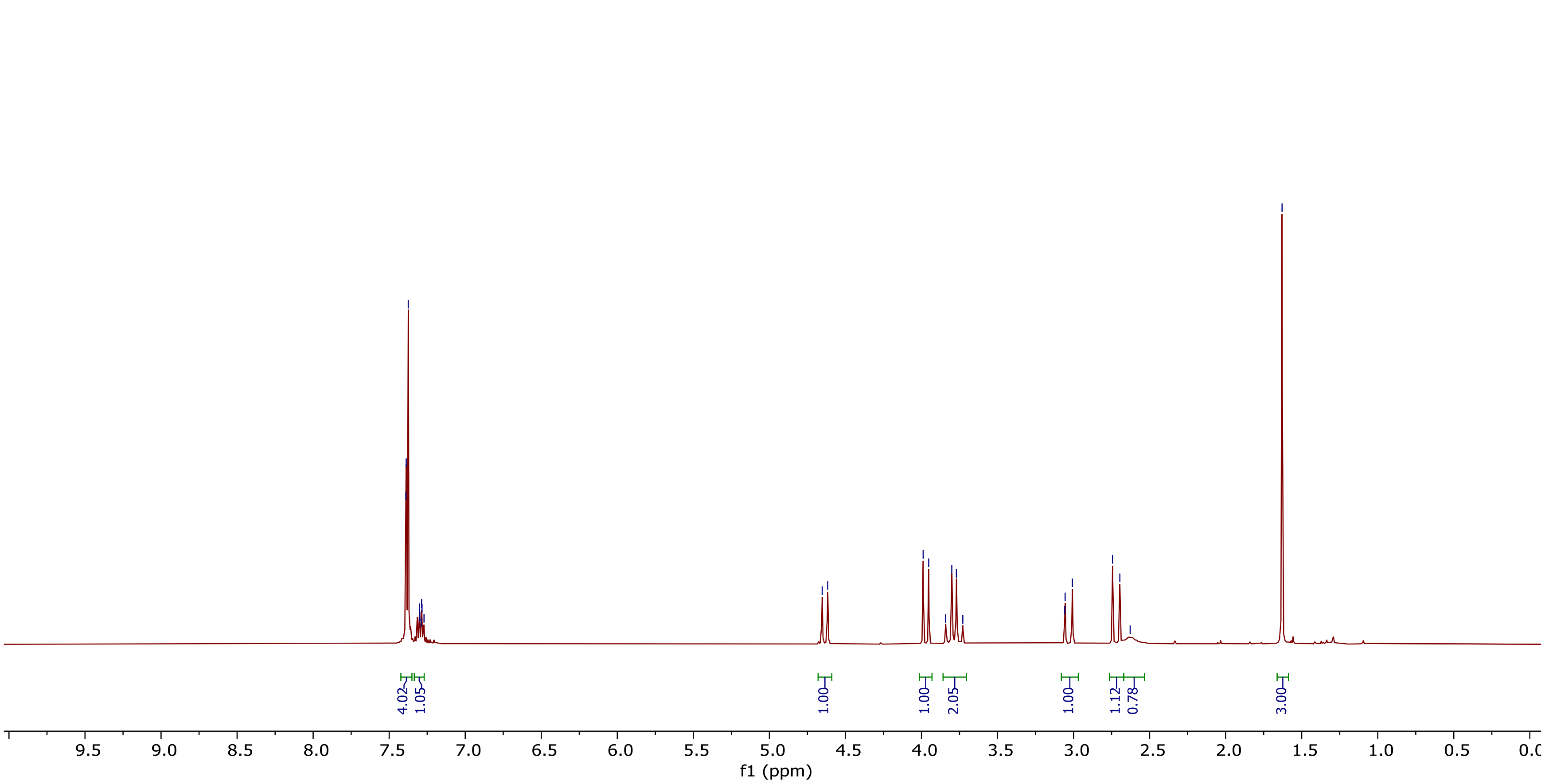

^{13}C NMR (75 MHz, $CDCl_3$) of **27a**

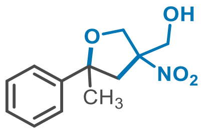

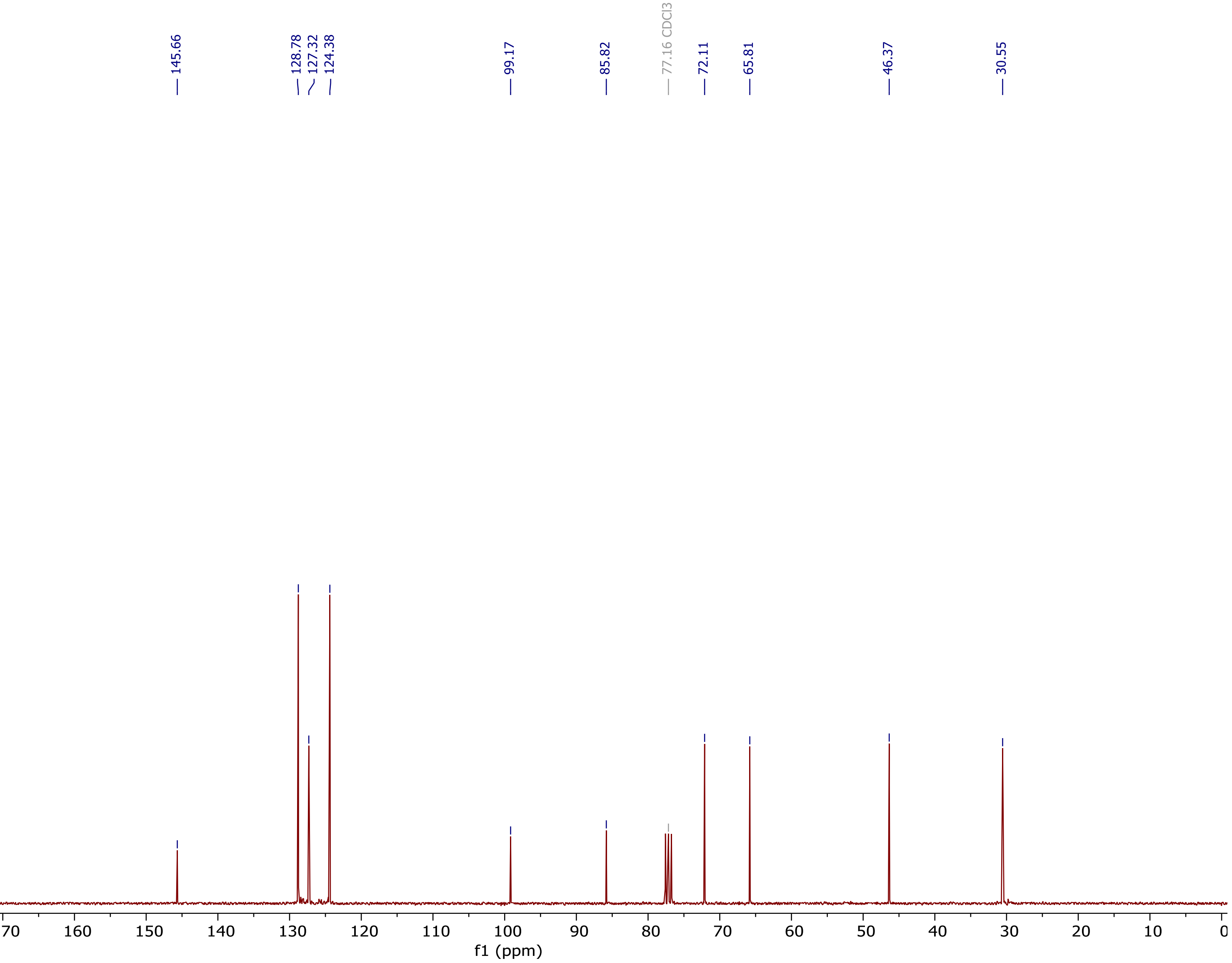

^{1}H NMR (300 MHz, $CDCl_3$) of **27b**

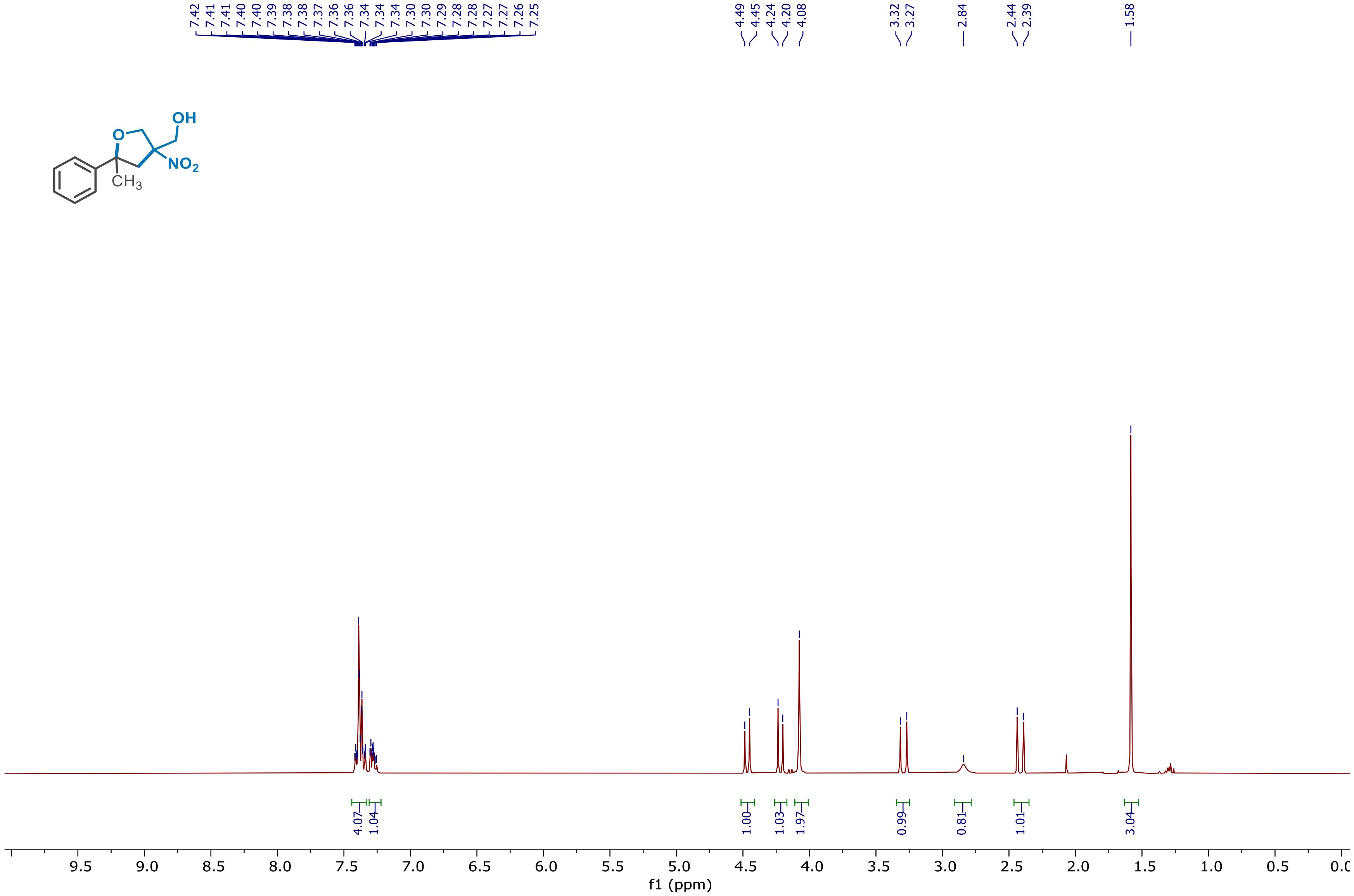

^{13}C NMR (75 MHz, $CDCl_3$) of **27b**

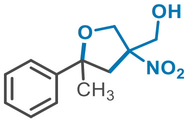

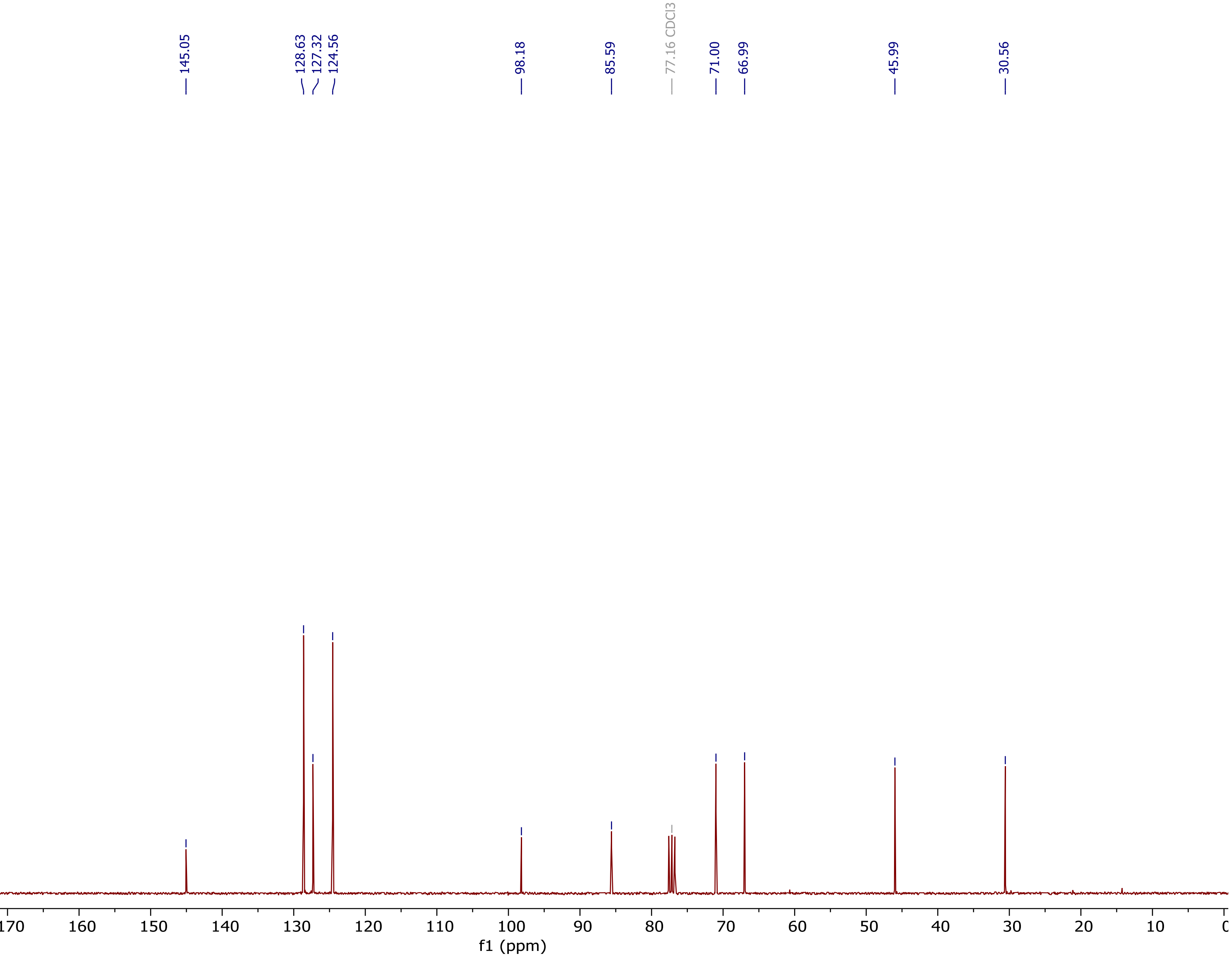

^{1}H NMR (300 MHz, $CDCl_3$) of **28a**

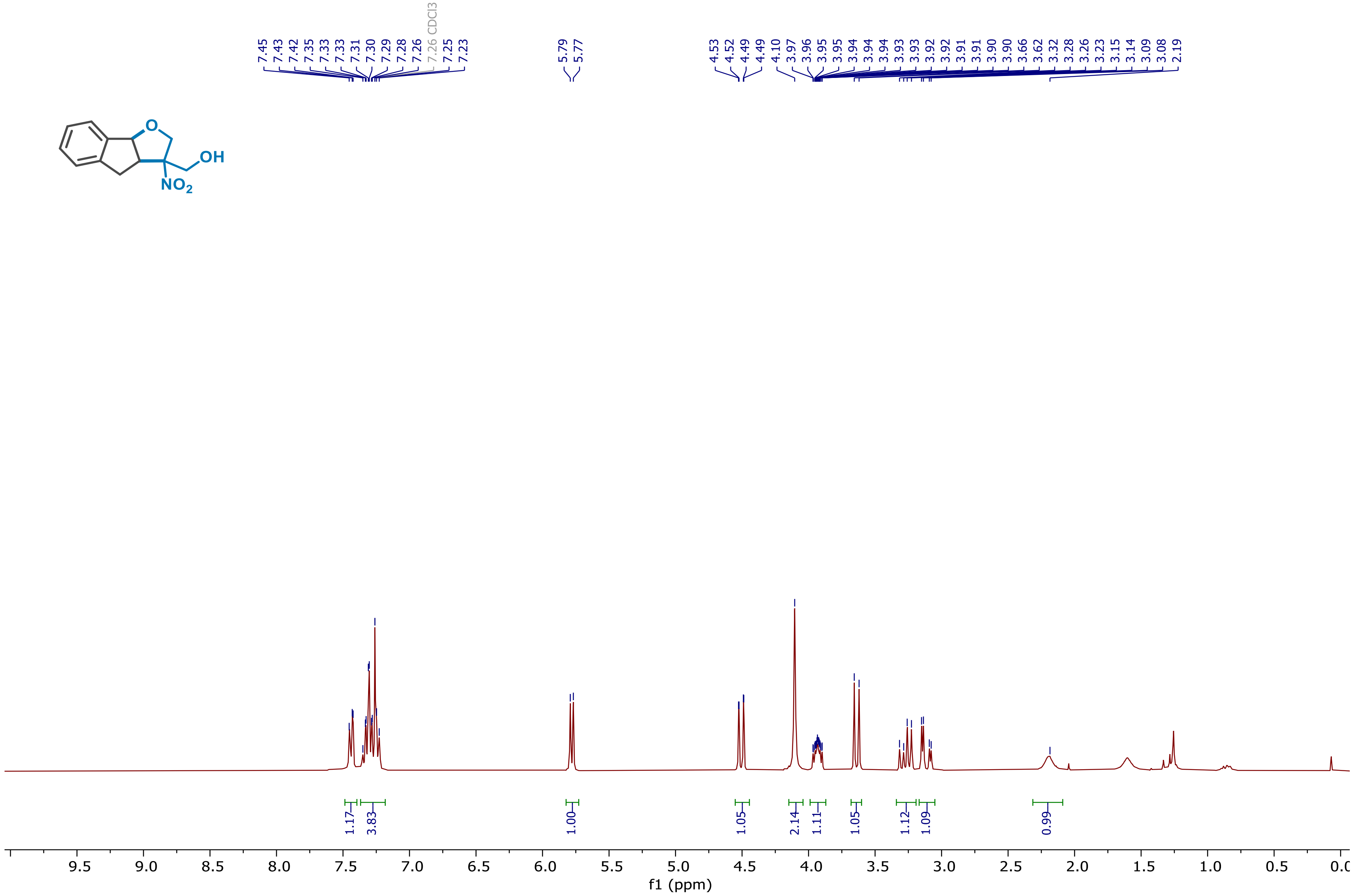

^{13}C NMR (75 MHz, $CDCl_3$) of **28a**

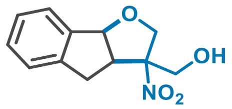

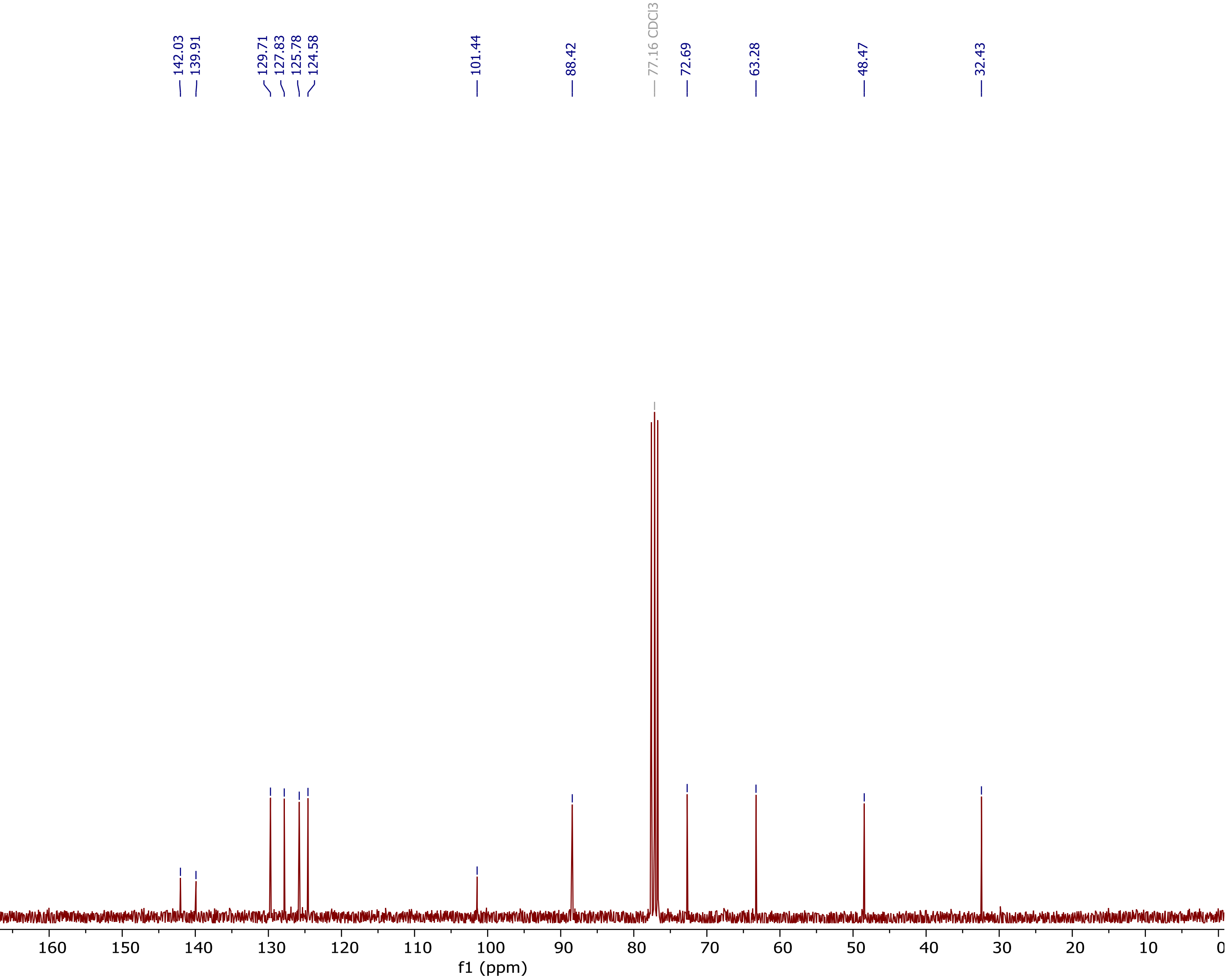

^{1}H NMR (300 MHz, $CDCl_3$) of **28b**

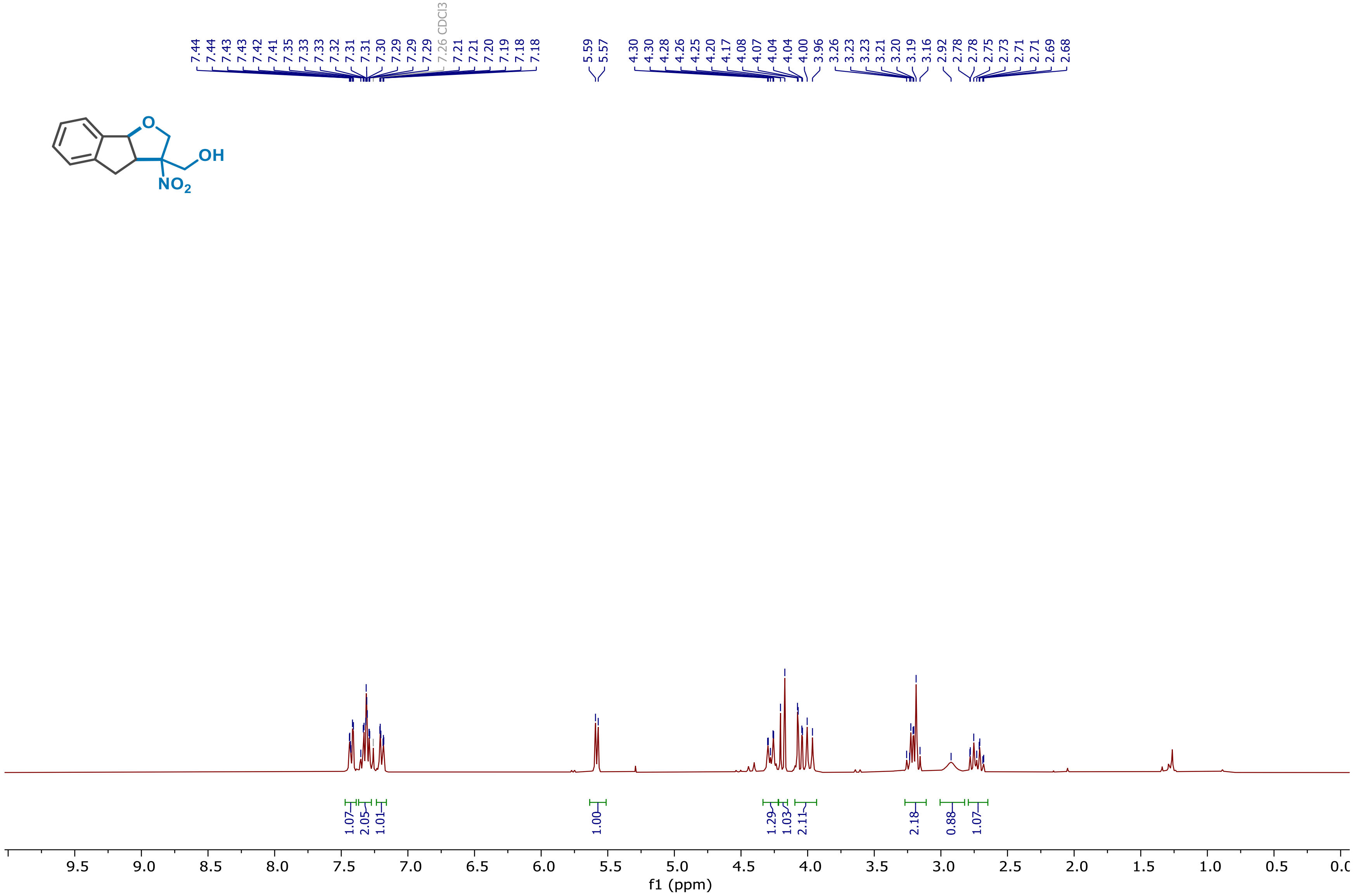

^{13}C NMR (75 MHz, $CDCl_3$) of **28b**

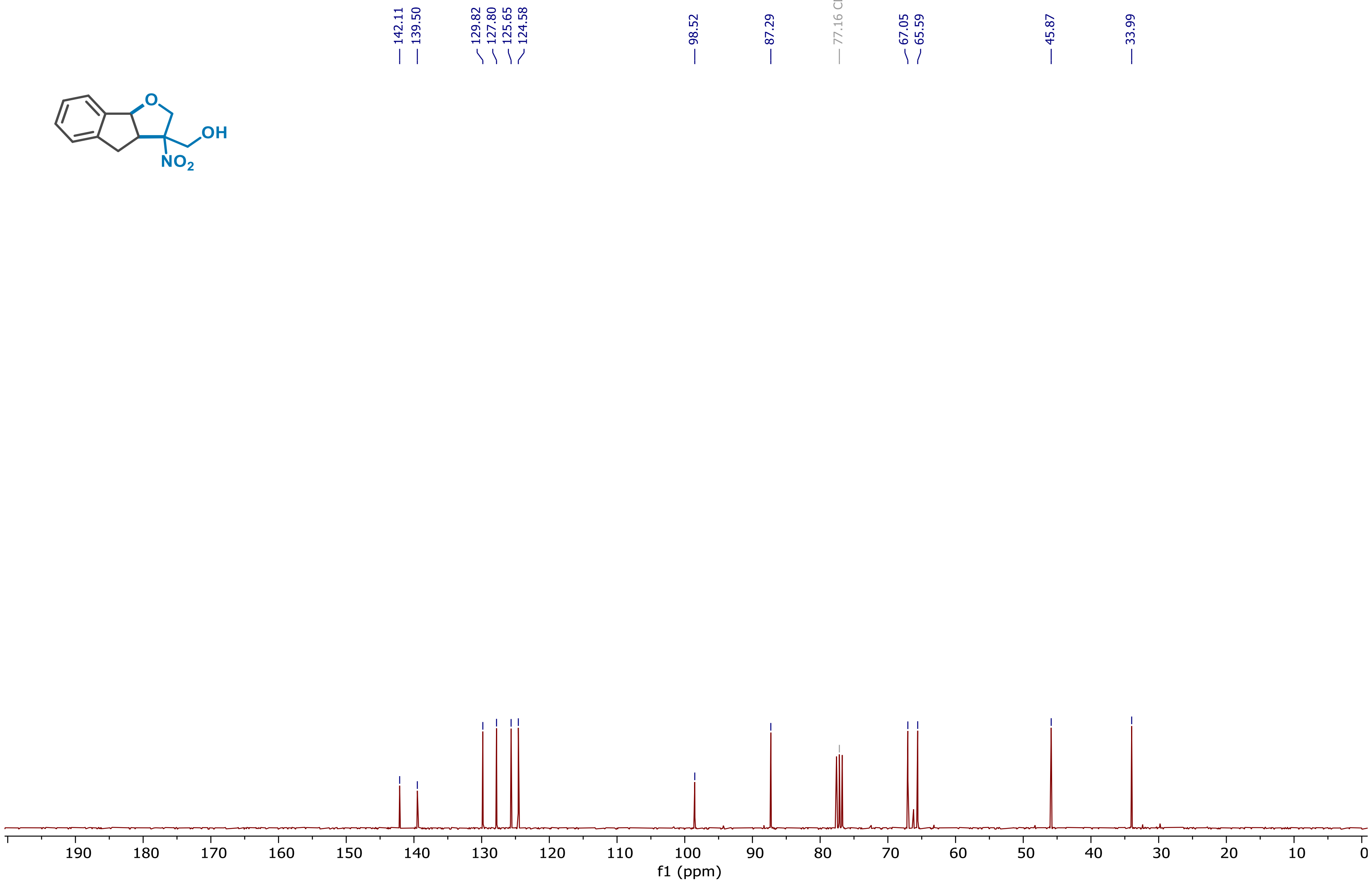

^{1}H NMR (300 MHz, $CDCl_3$) of **29a**

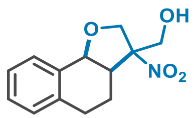

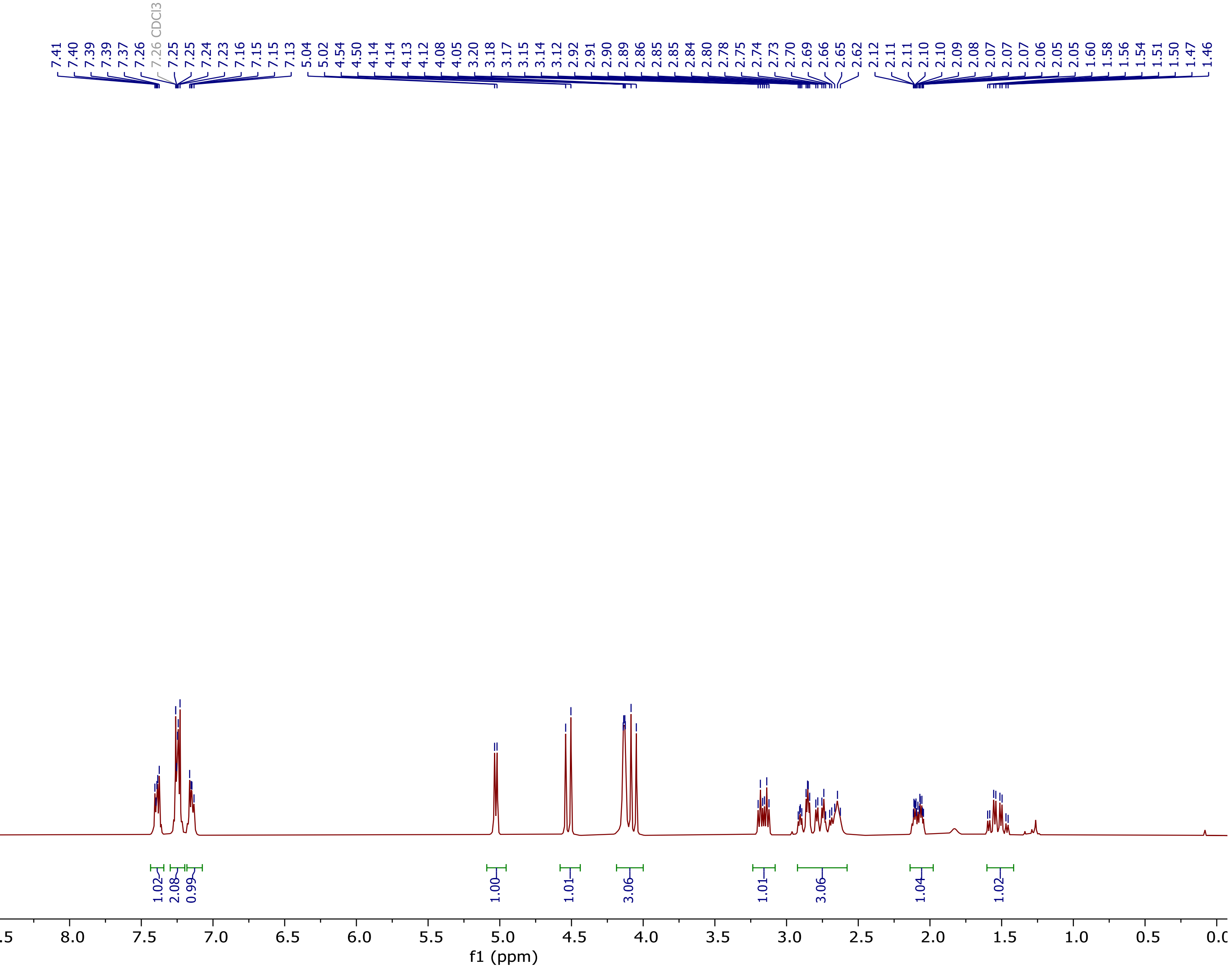

^{13}C NMR (75 MHz, $CDCl_3$) of **29a**

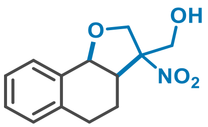

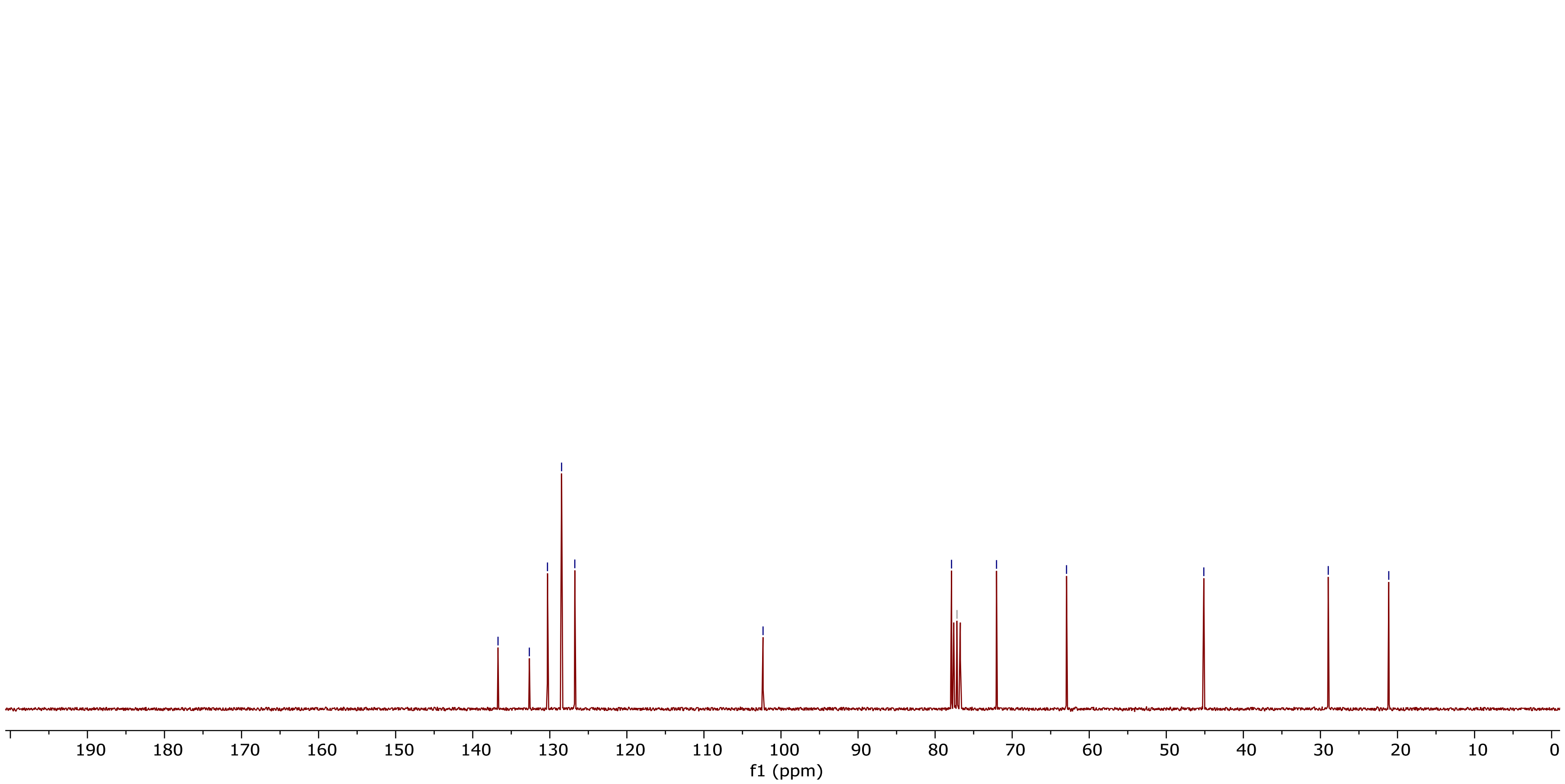

^{1}H NMR (300 MHz, $CDCl_3$) of **29b**

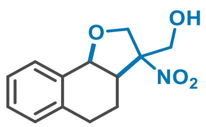

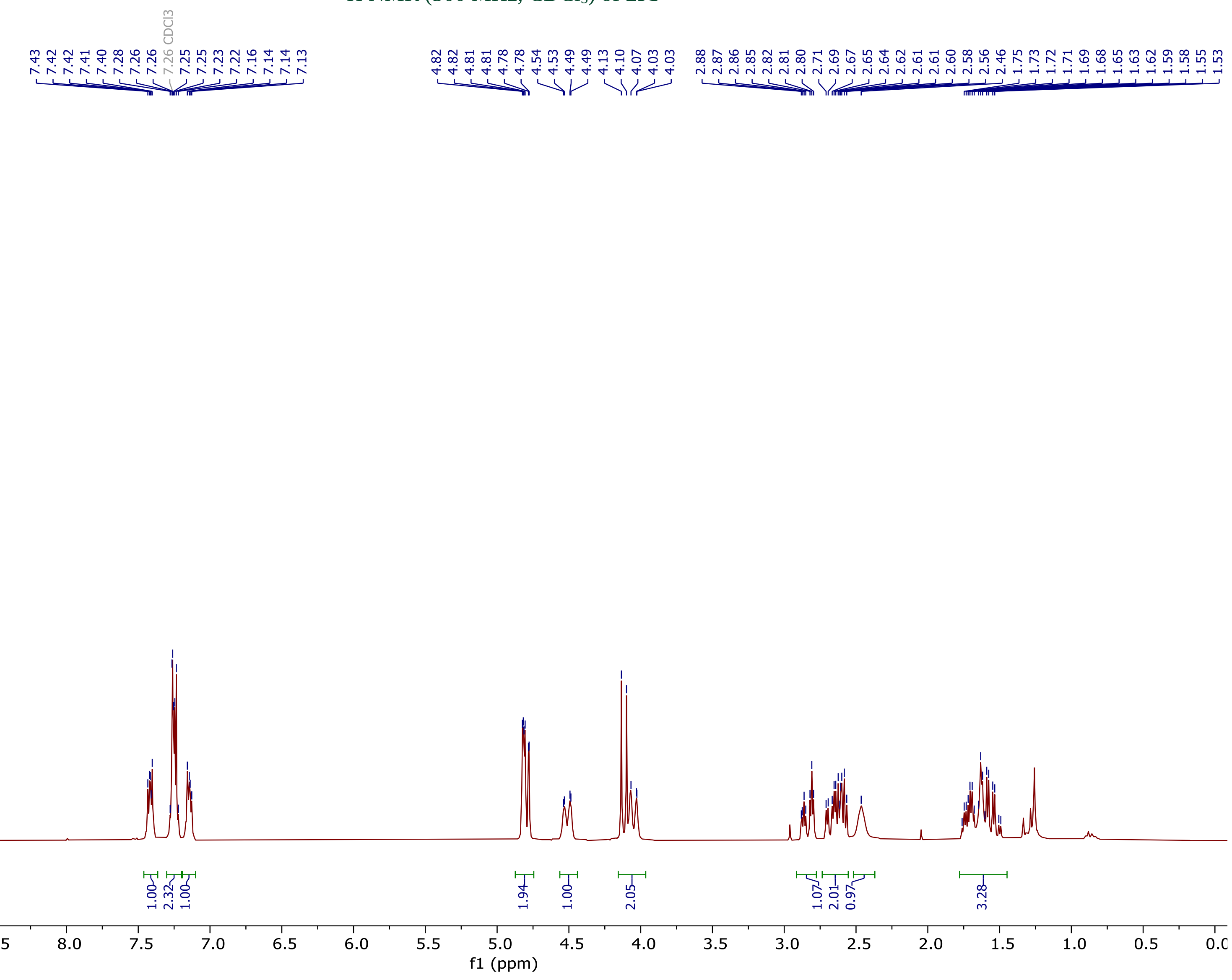

^{13}C NMR (75 MHz, $CDCl_3$) of **29b**

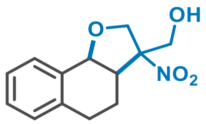

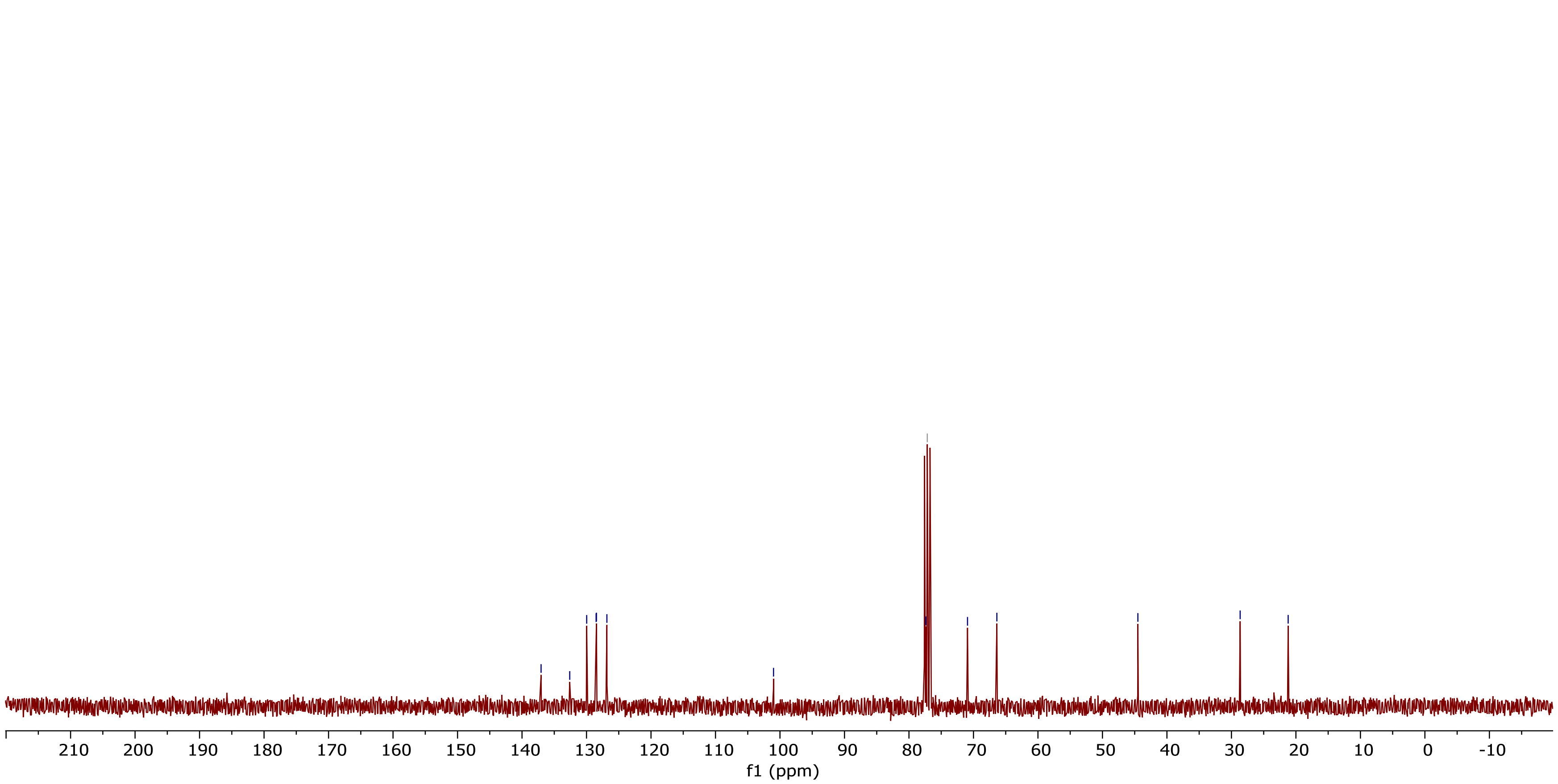

^{1}H NMR (300 MHz, $CDCl_3$) of **30**

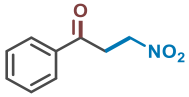

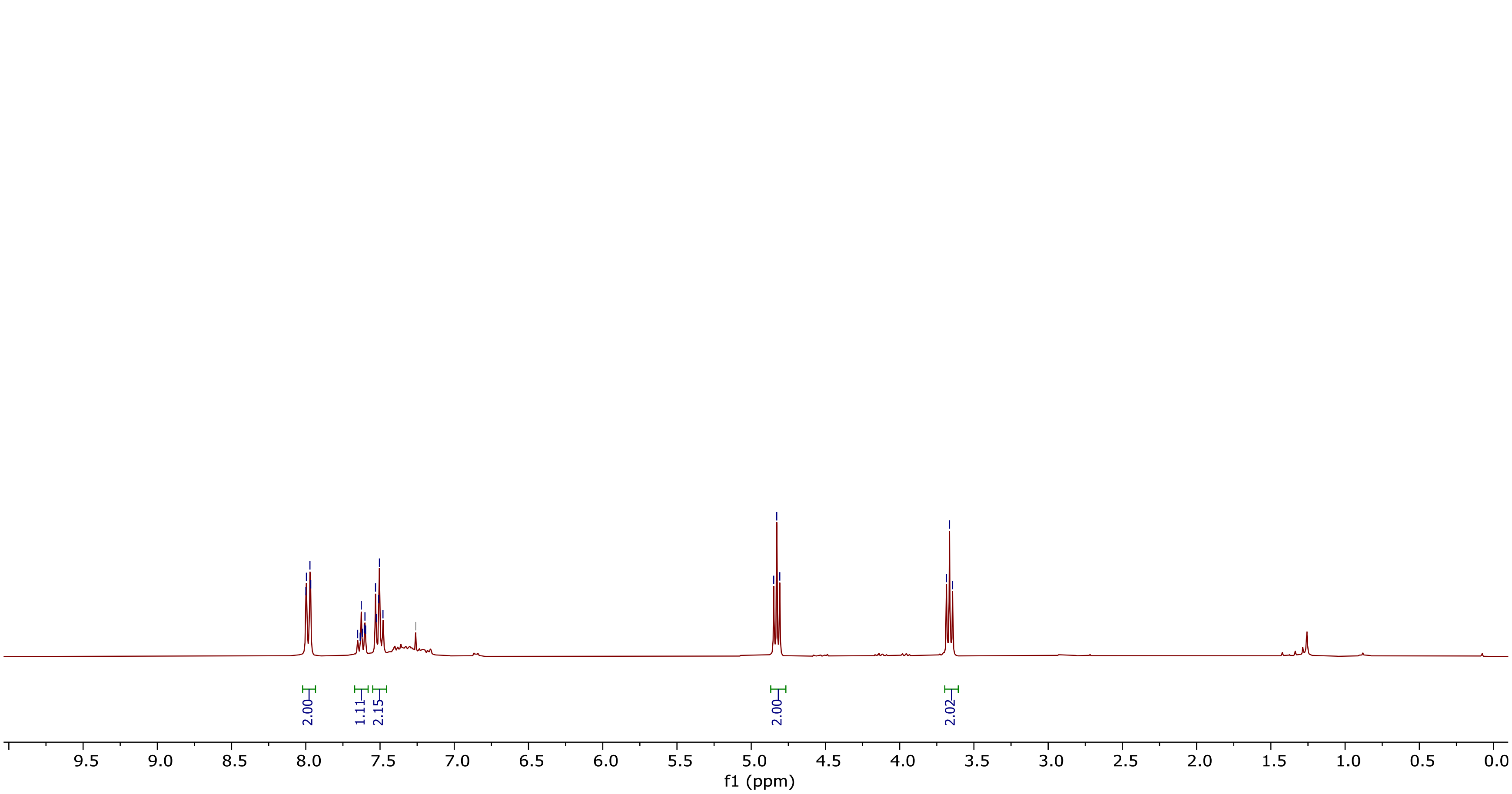

^{13}C NMR (75 MHz, $CDCl_3$) of **30**

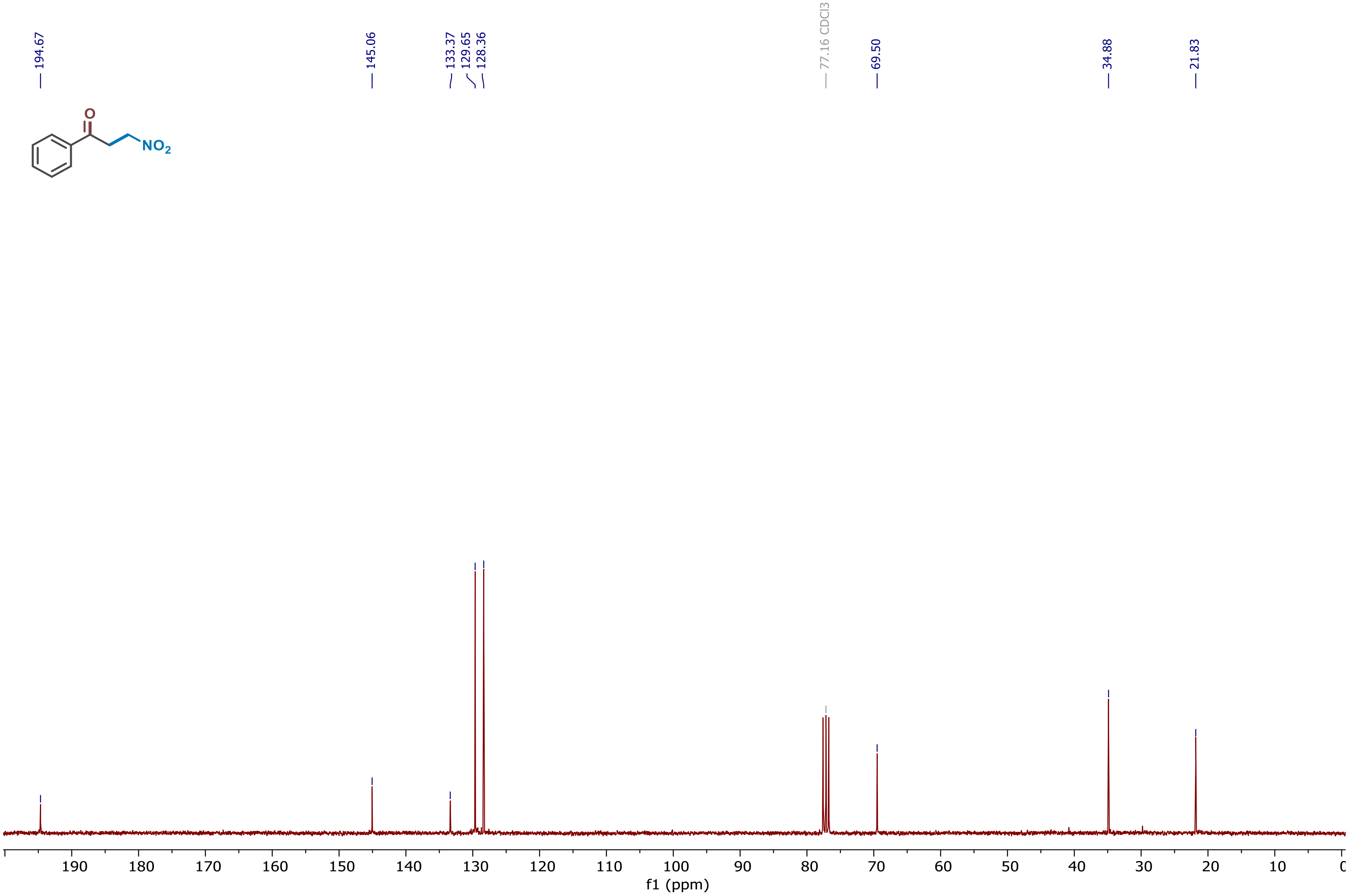

^{1}H NMR (300 MHz, $CDCl_3$) of **31**

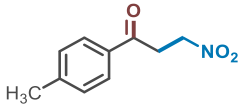

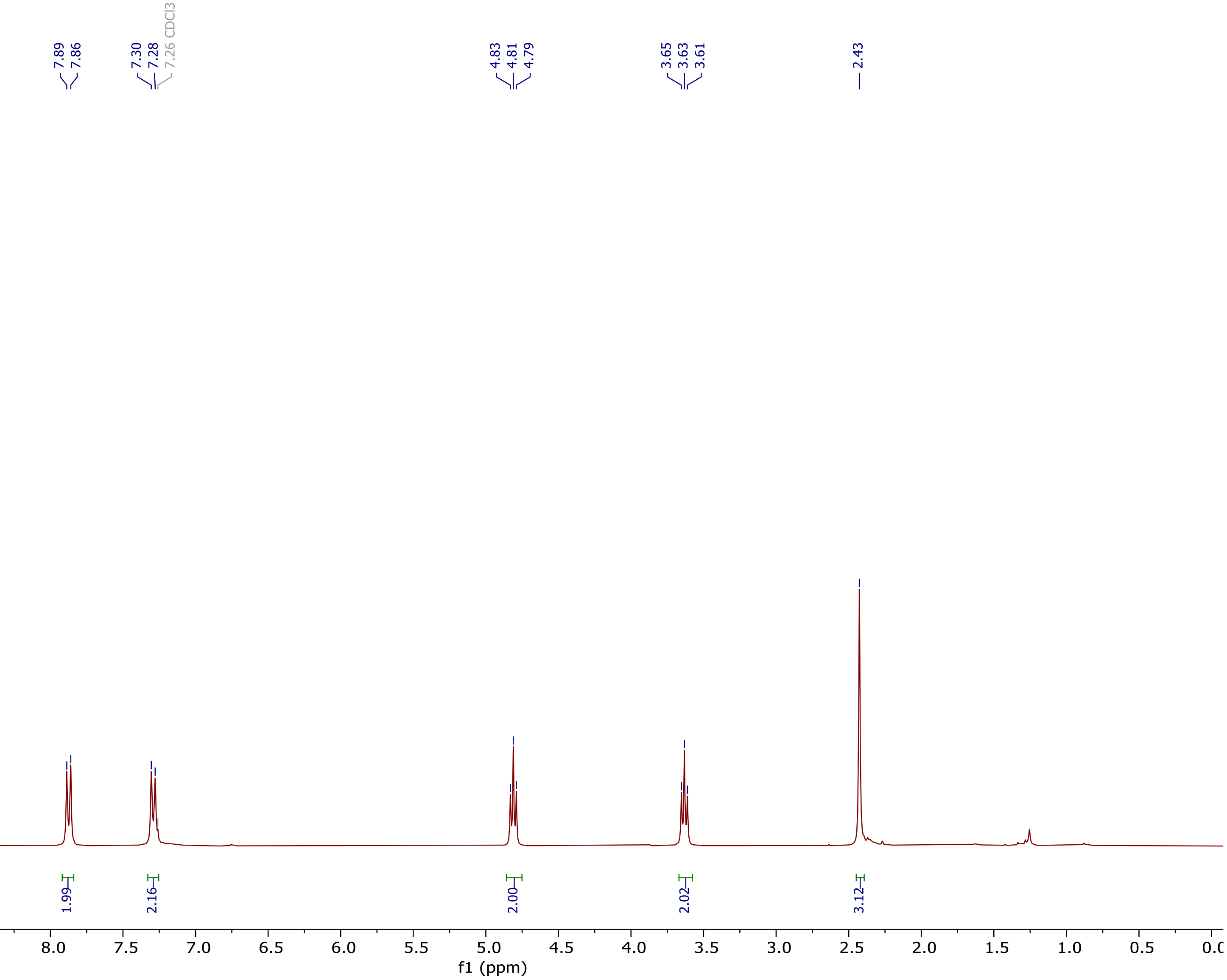

^{13}C NMR (75 MHz, $CDCl_3$) of **31**

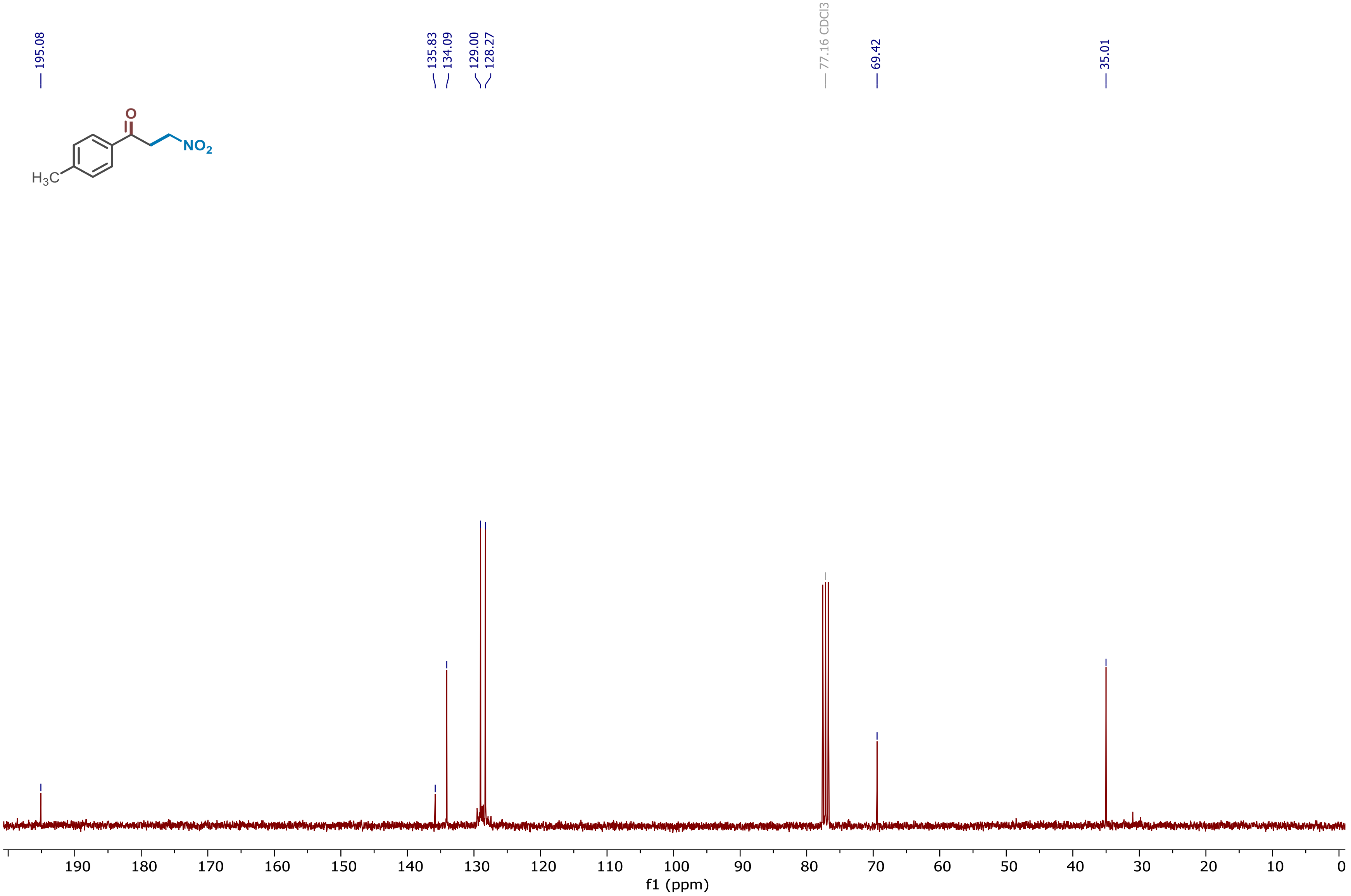

^{1}H NMR (300 MHz, $CDCl_3$) of **32**

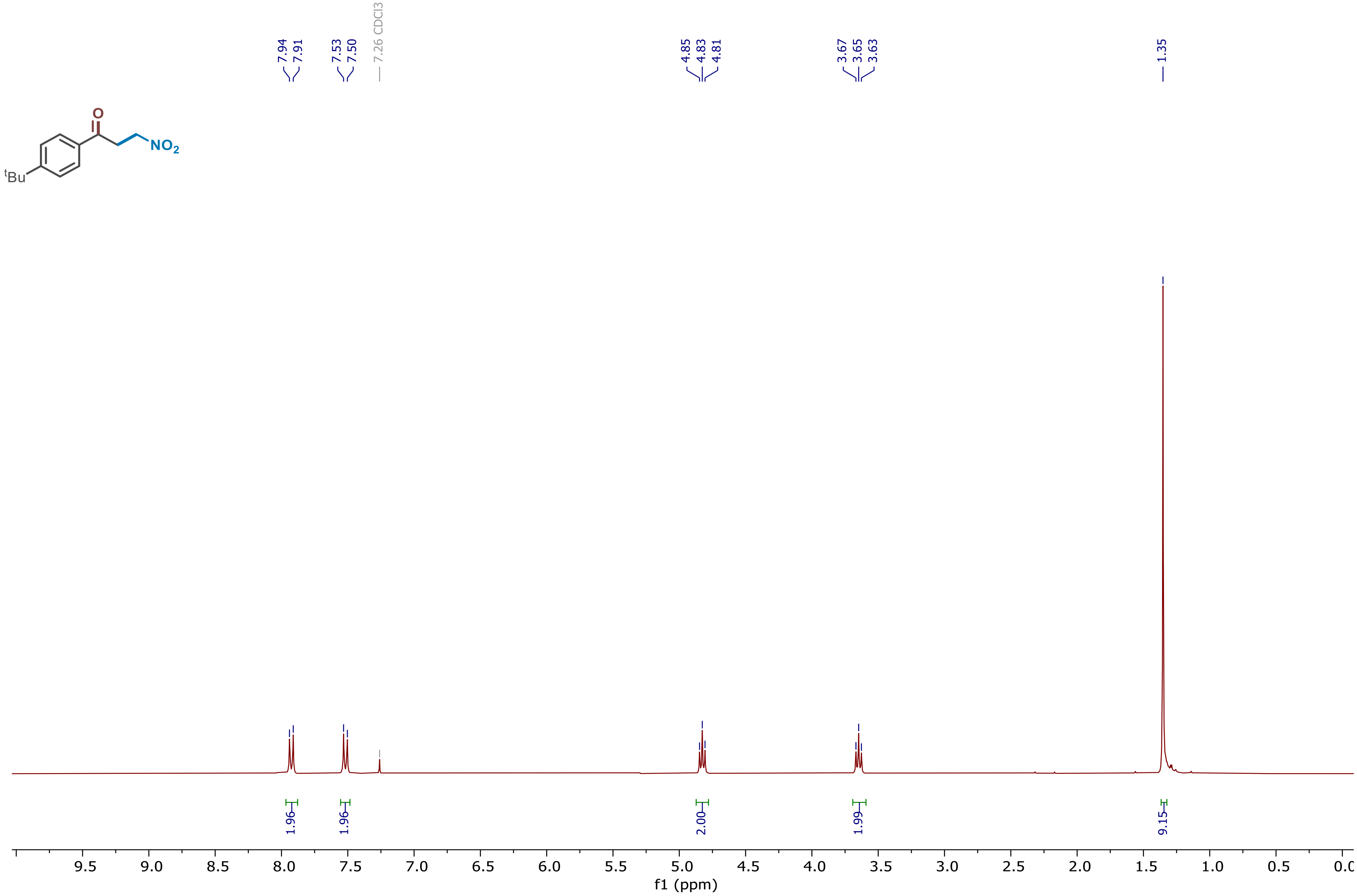

^{1}H NMR (300 MHz, $CDCl_3$) of **34**

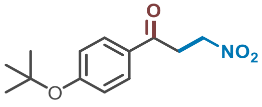

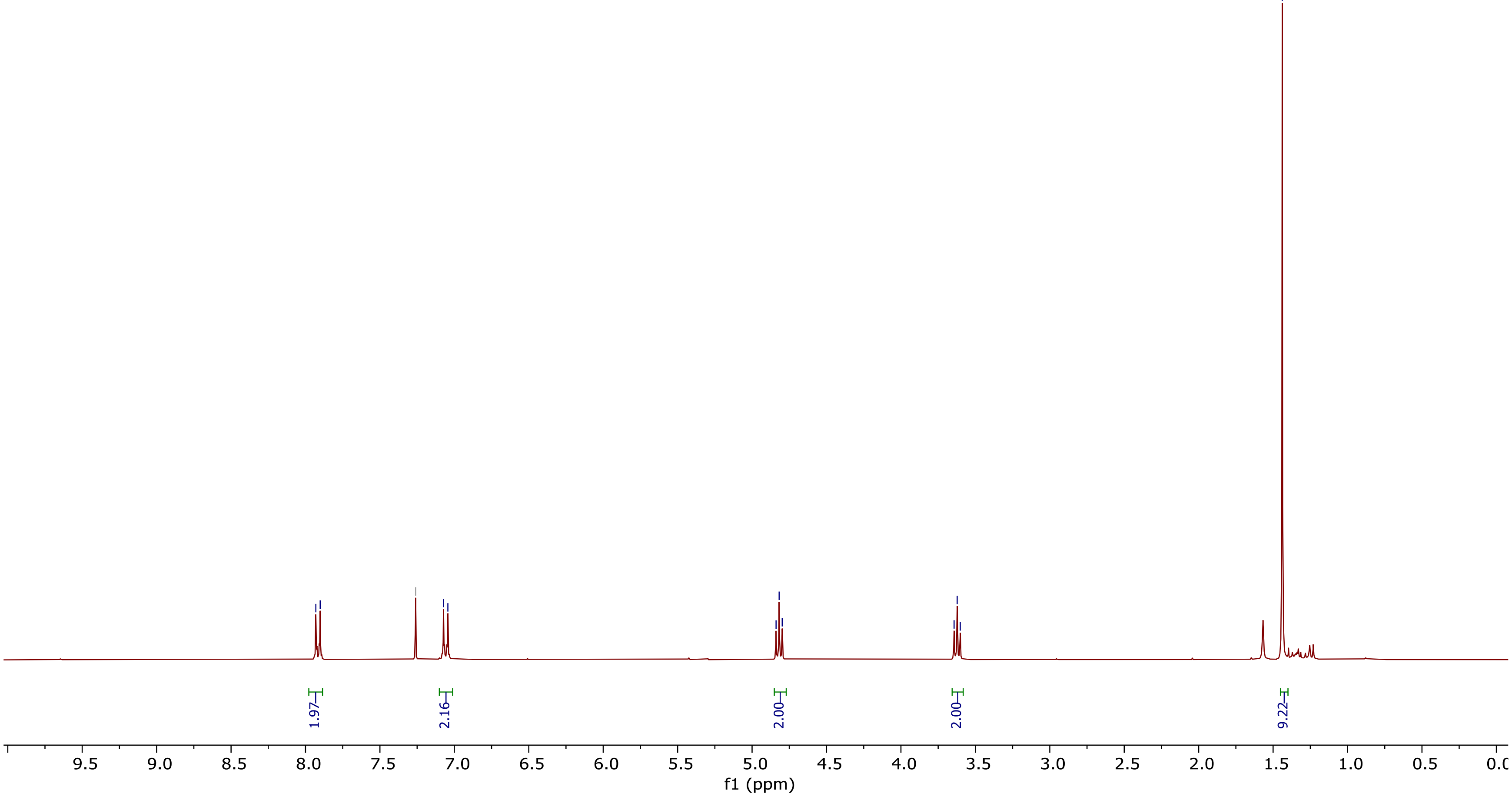

^{13}C NMR (75 MHz, CDCl$_3$) of **34**

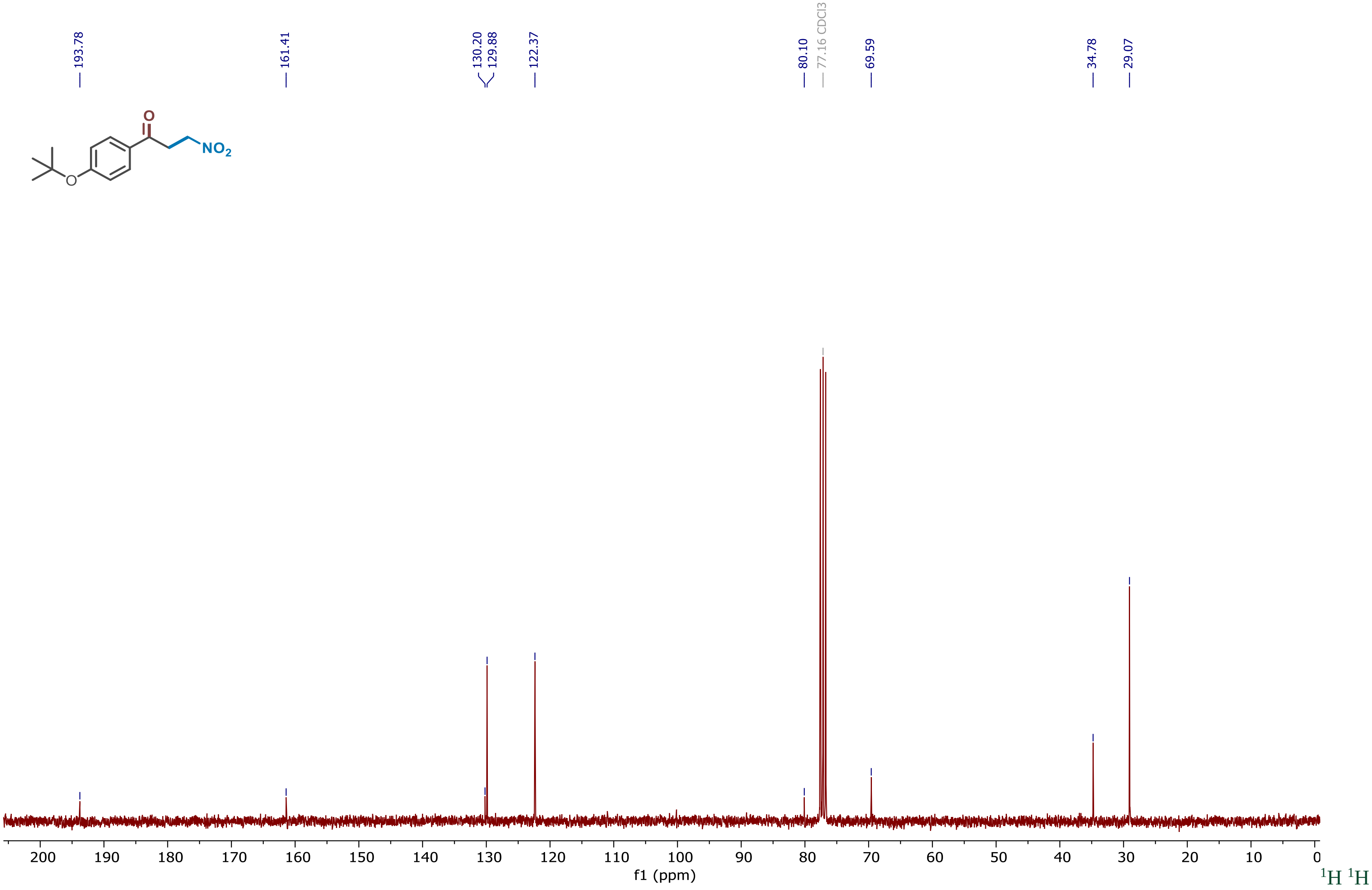

^{1}H NMR (300 MHz, $CDCl_3$) of **35**

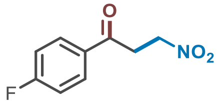

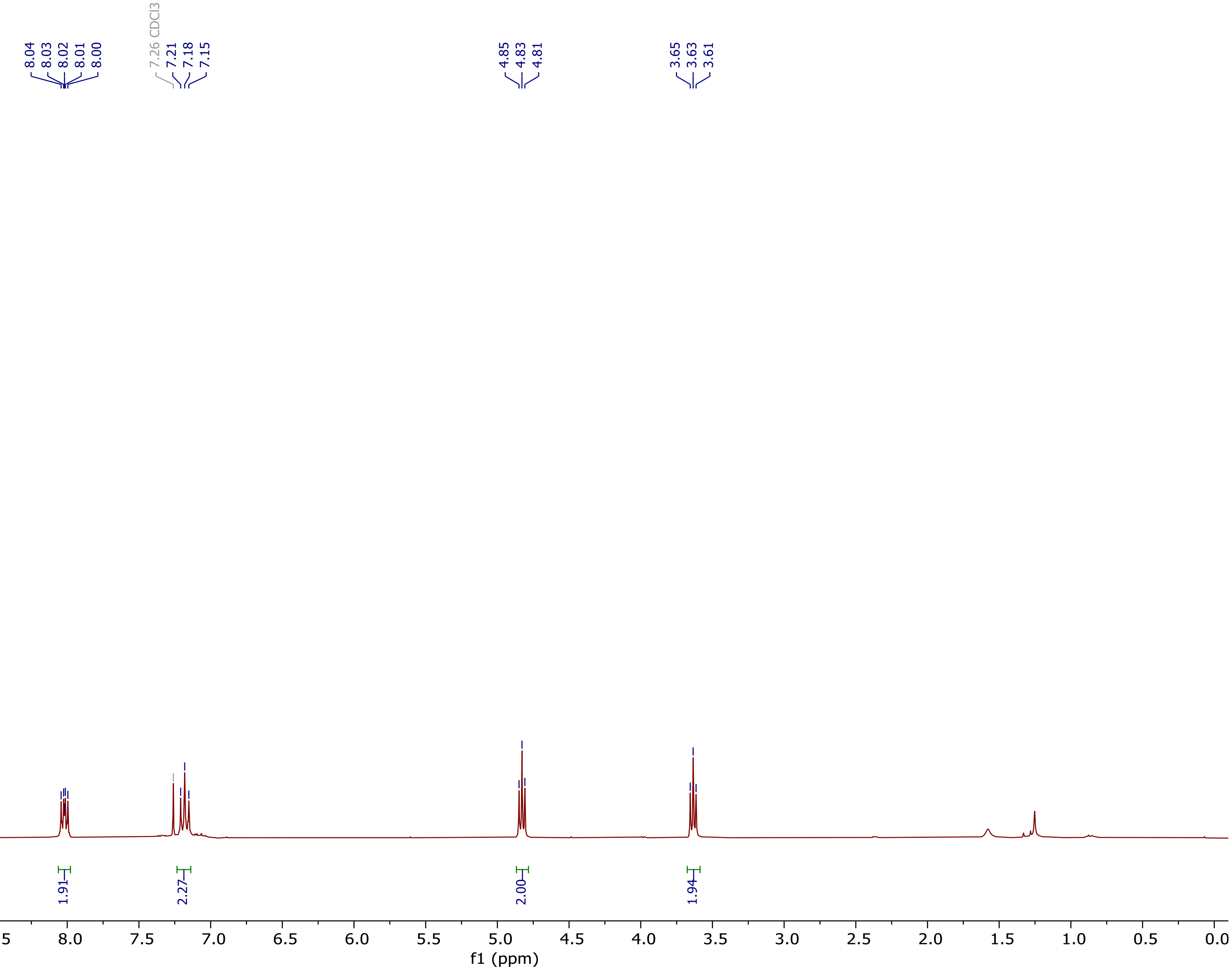

^{1}H-NMR (300 MHz, $CDCl_3$) of **36**

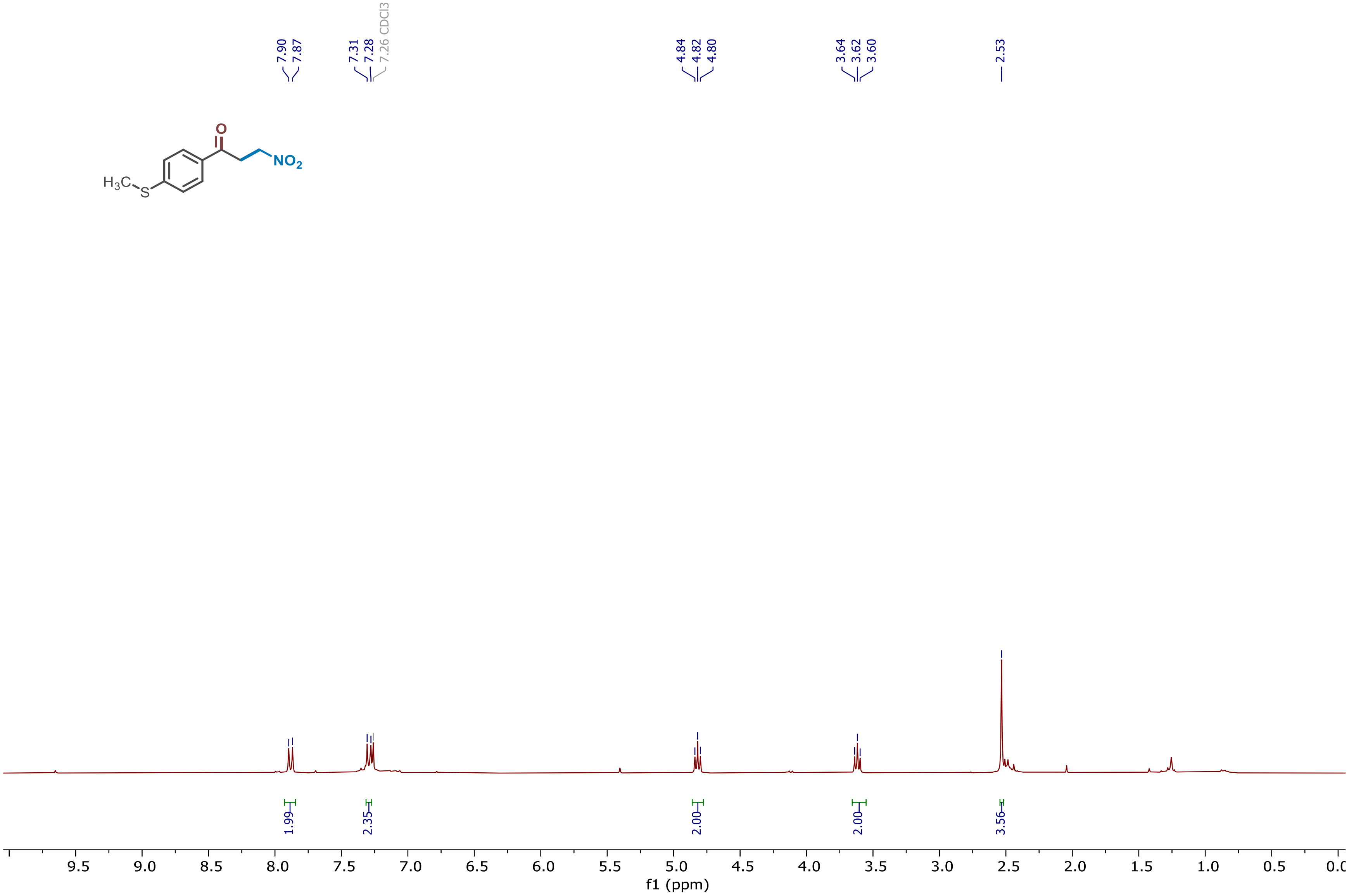

^{13}C NMR (75 MHz, $CDCl_3$) of **36**

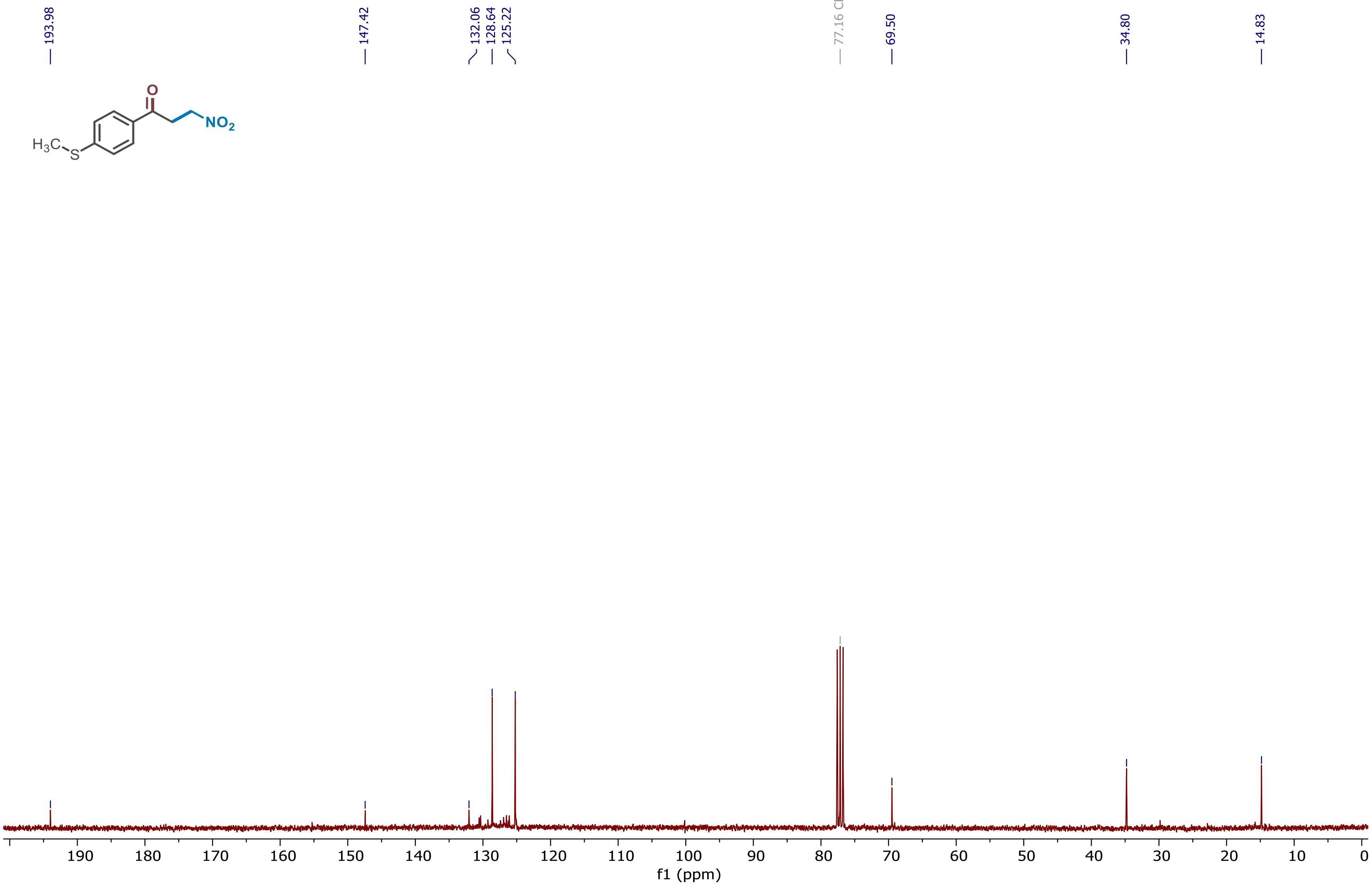

NMR (300 MHz, $CDCl_3$) of **37**

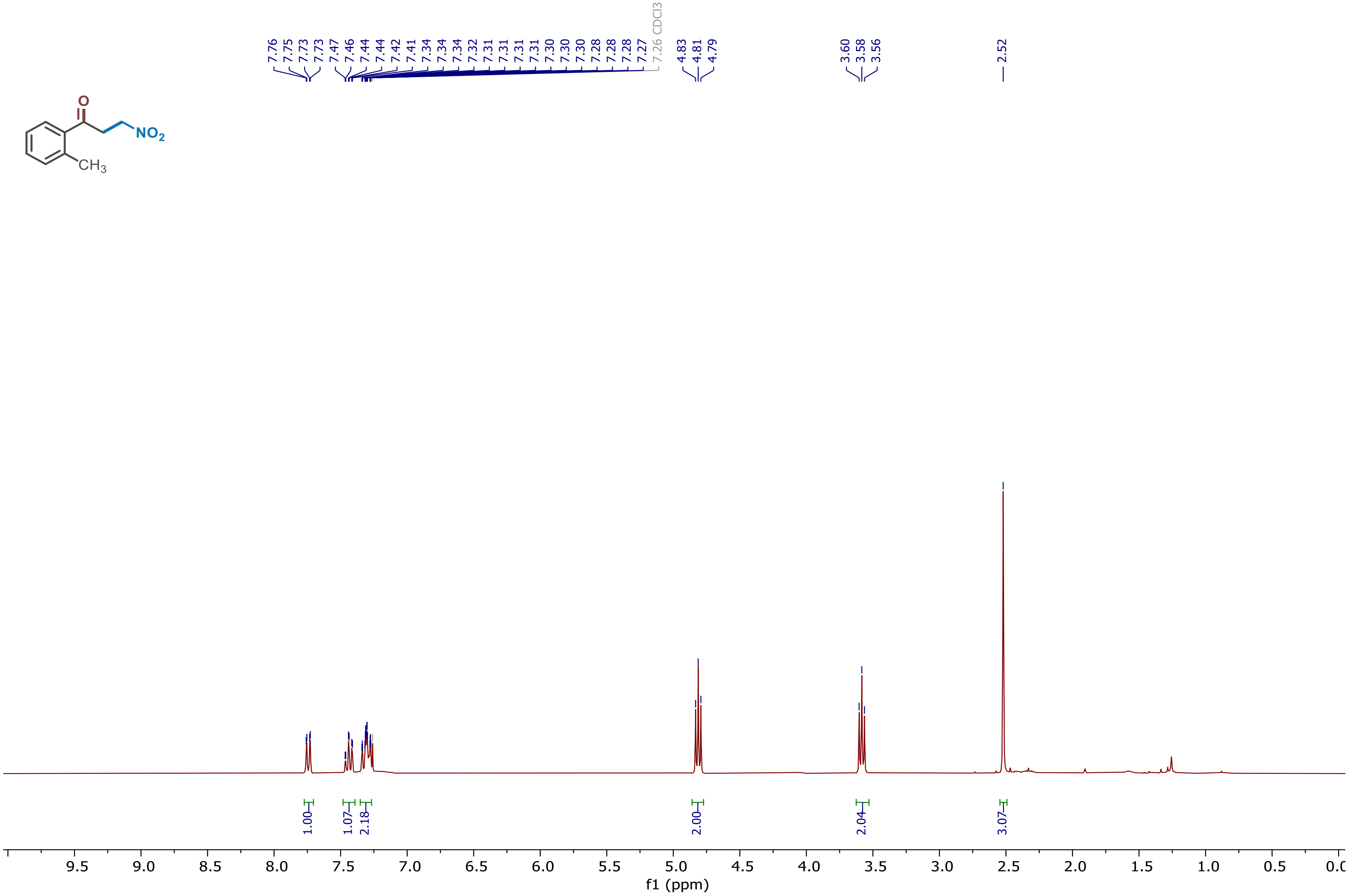

^{13}C NMR (75 MHz, $CDCl_3$) of **37**

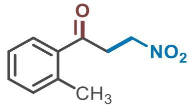

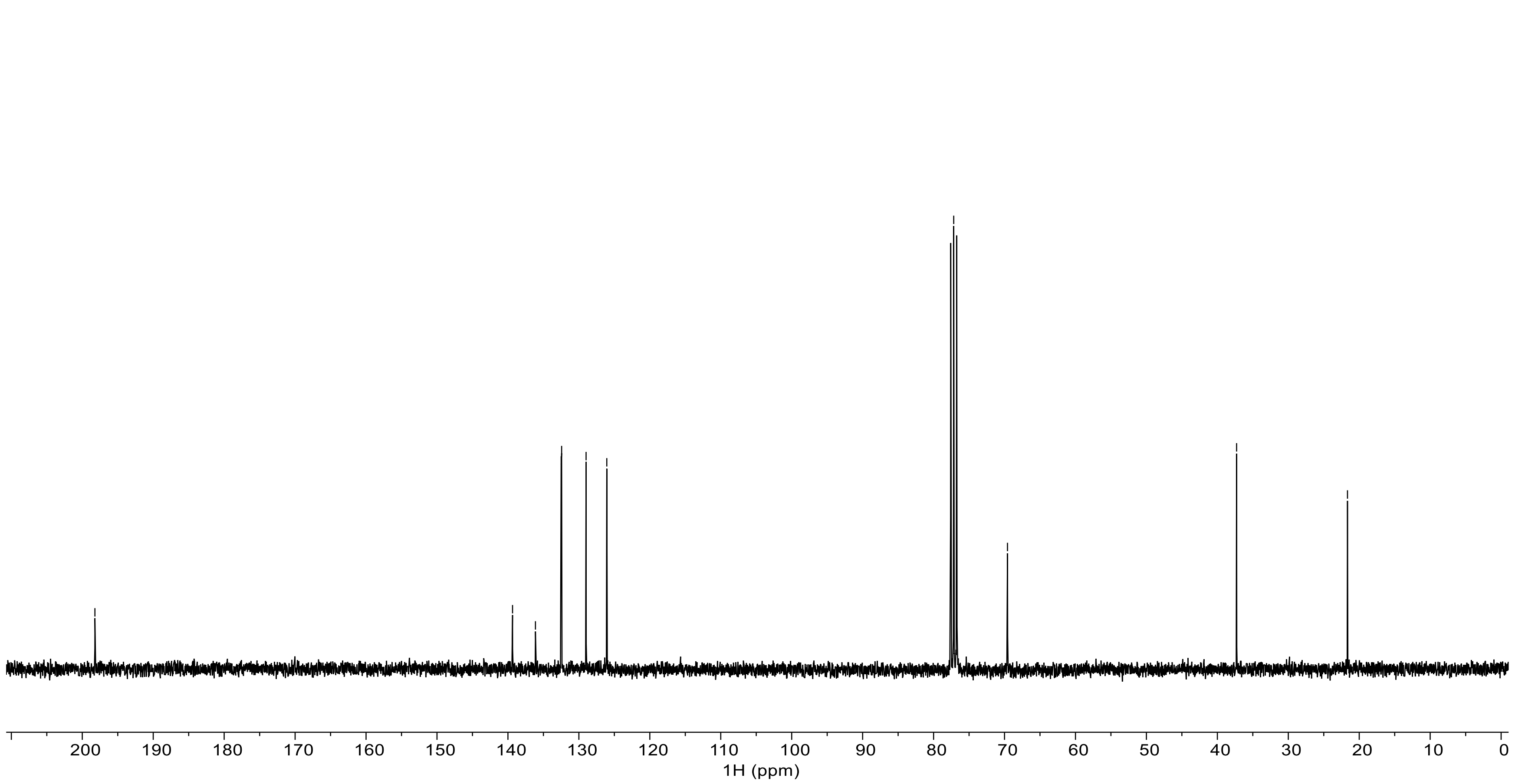

^{1}H NMR (300 MHz, $CDCl_3$) of **38**

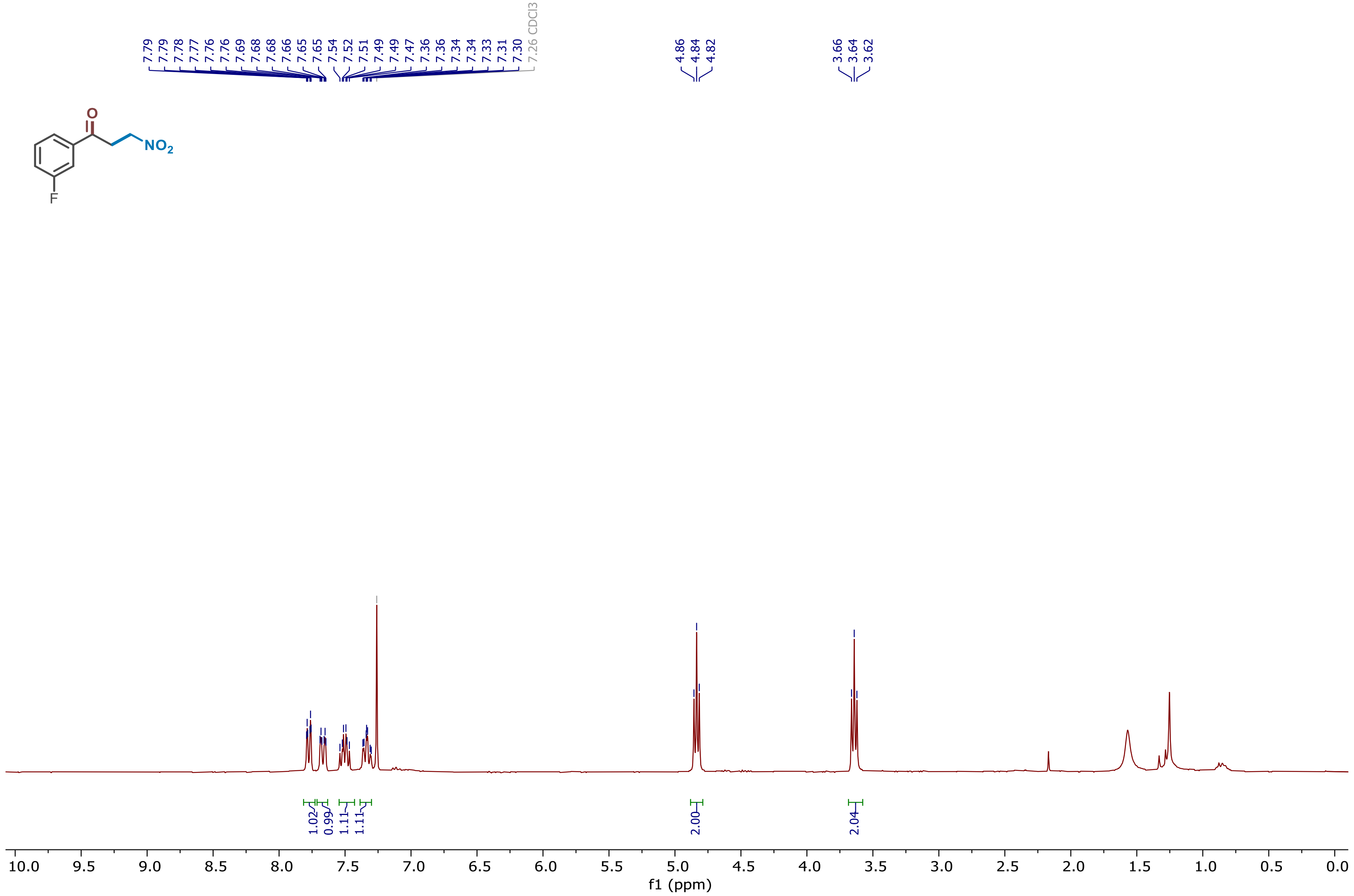

^{19}F NMR (282 MHz, $CDCl_3$) of **38**

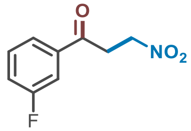

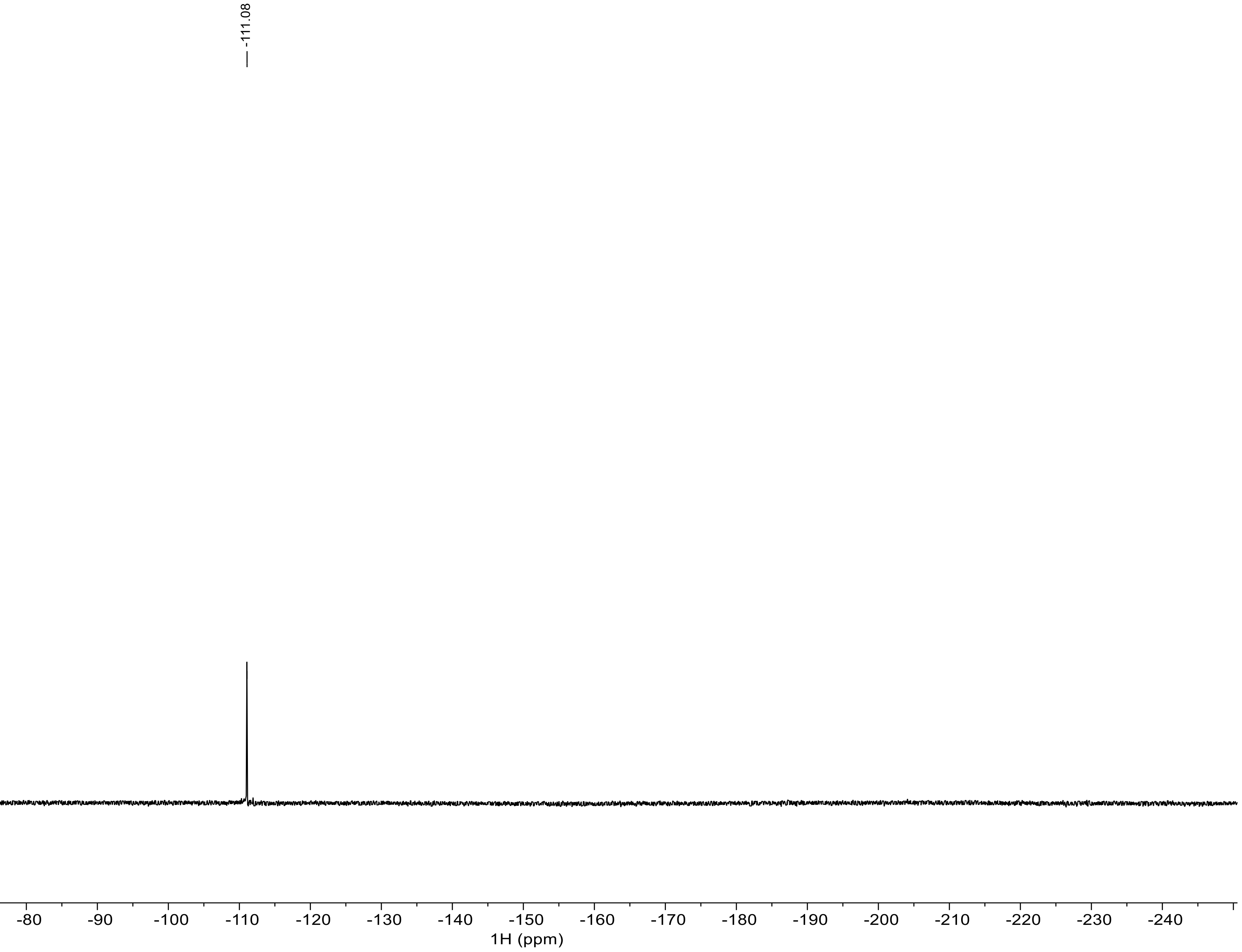

^{13}C NMR (75 MHz, CDCl$_3$) of **38**

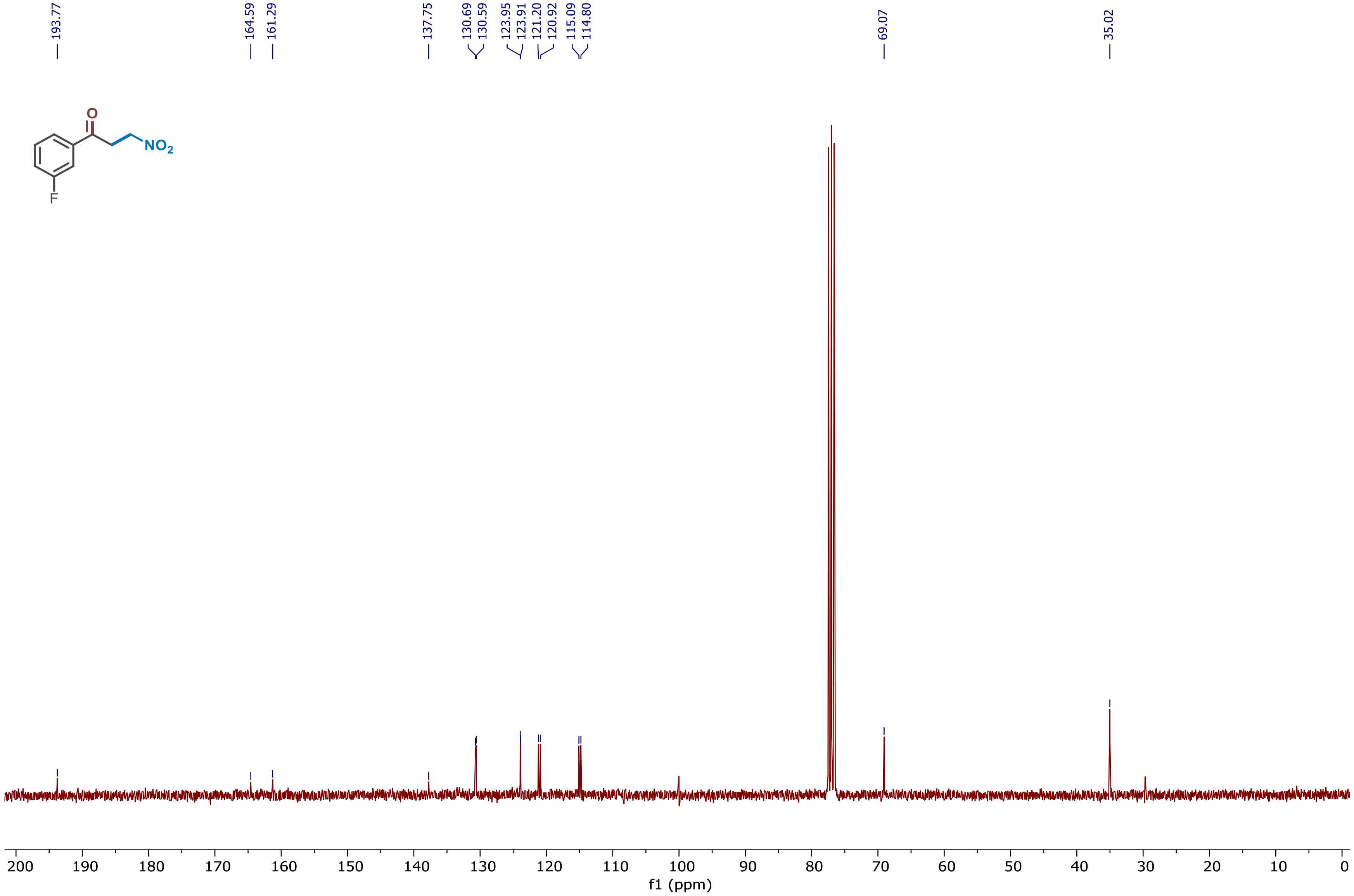

^{1}H NMR (300 MHz, $CDCl_3$) of **39**

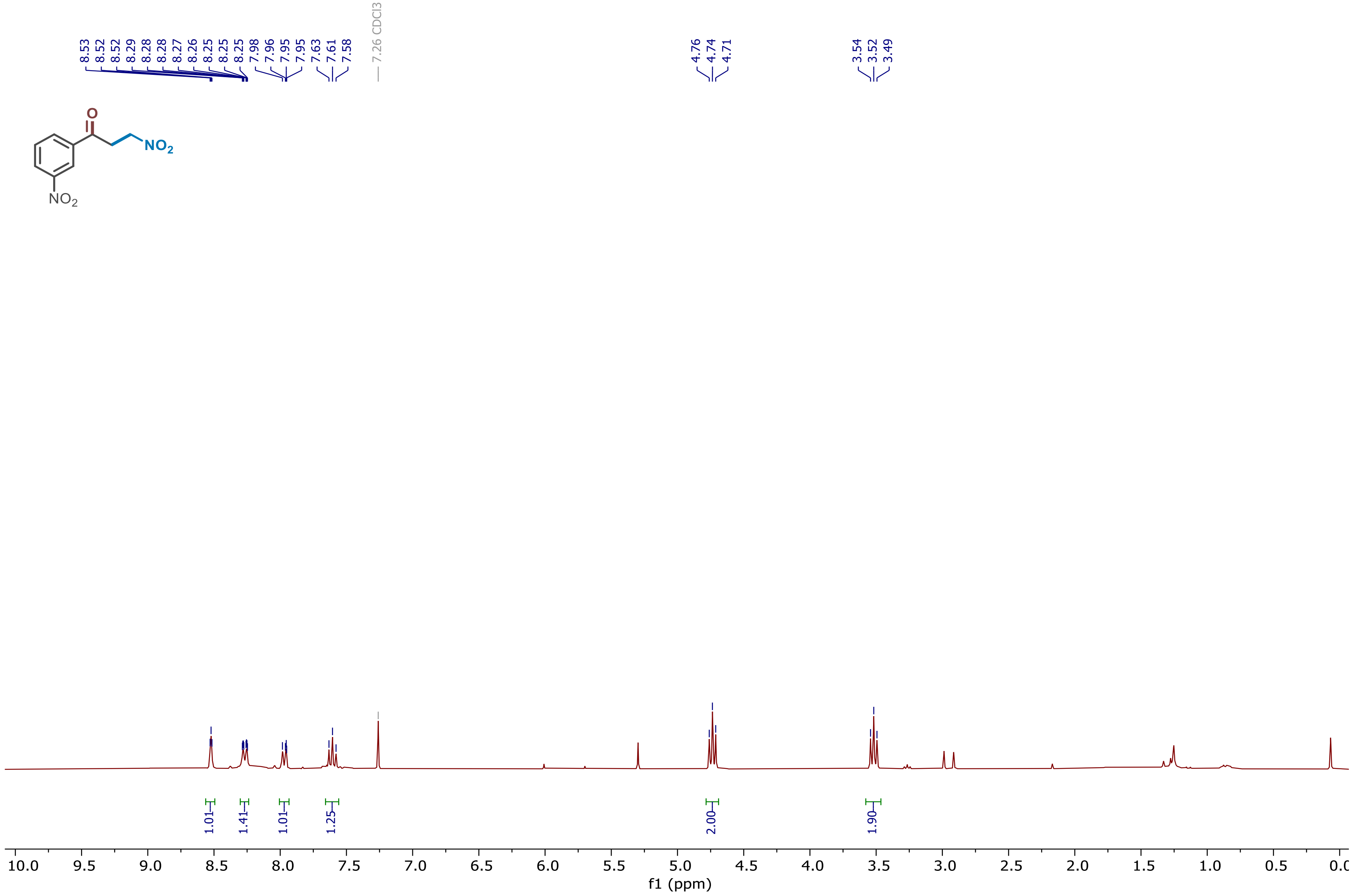

^{13}C NMR (75 MHz, $CDCl_3$) of **39**

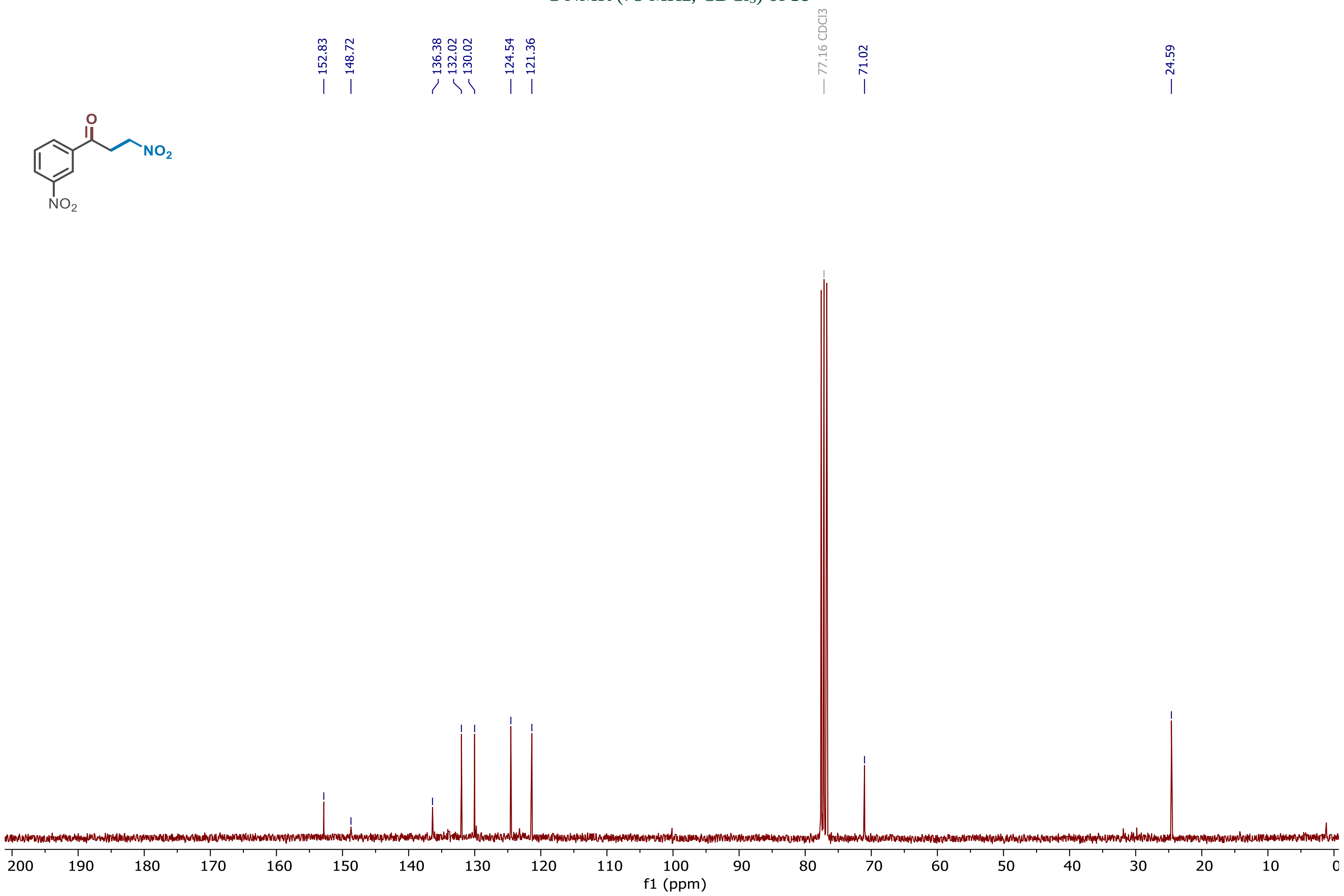

^{1}H NMR (300 MHz, $CDCl_3$) of **40**

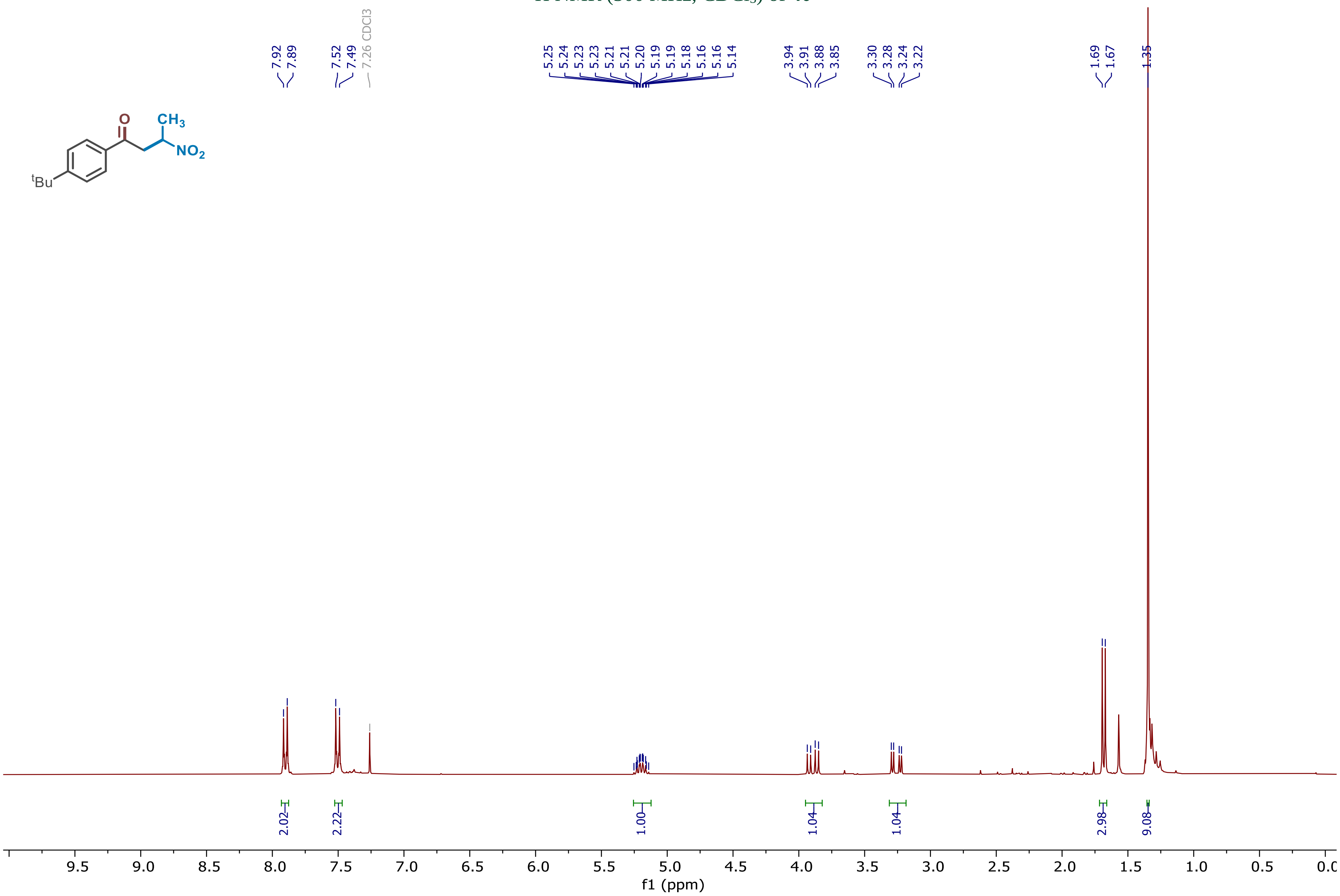

^{13}C NMR (75 MHz, CDCl$_3$) of **40**

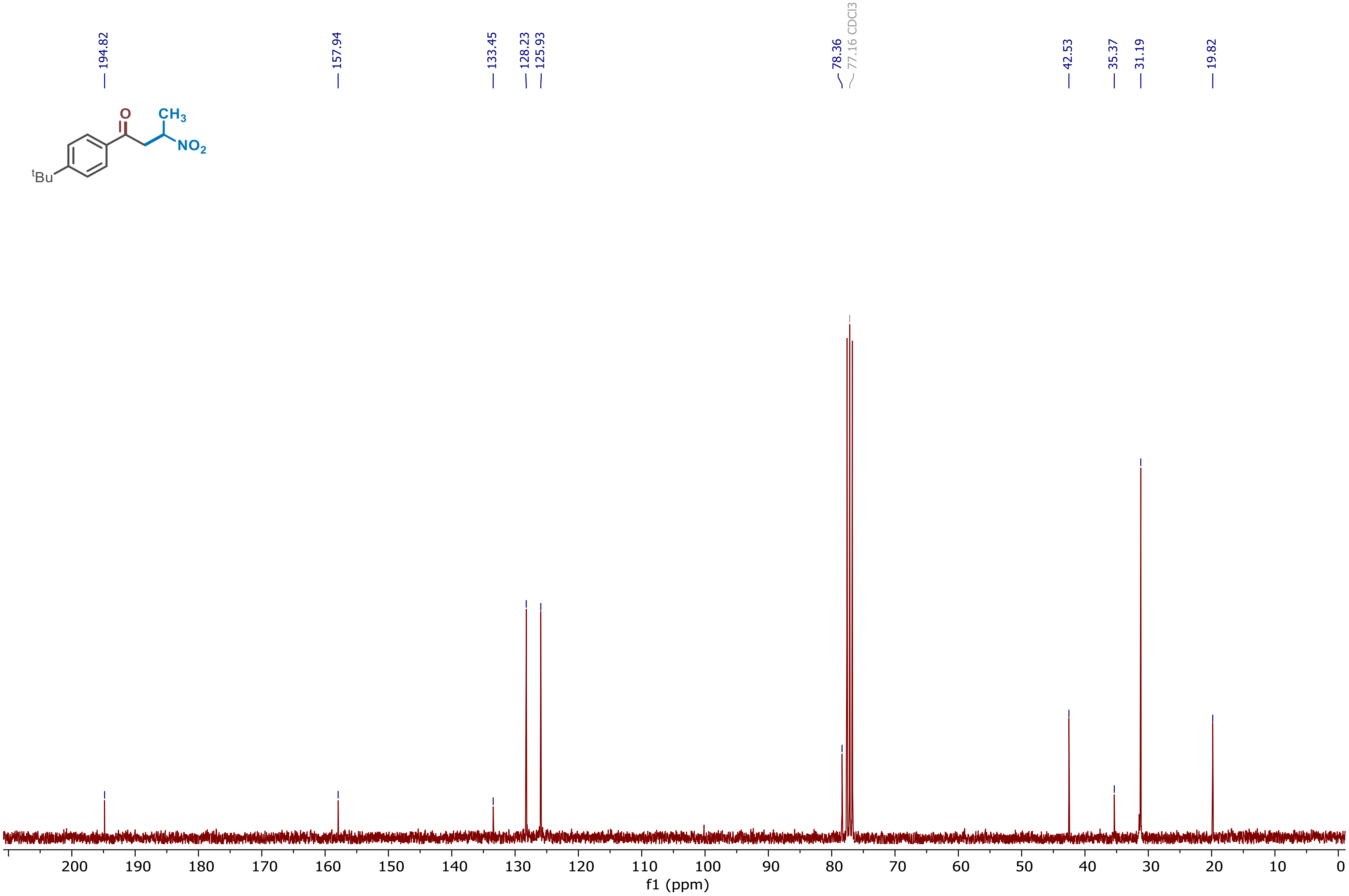

^{1}H NMR (300 MHz, $CDCl_3$) of **41**

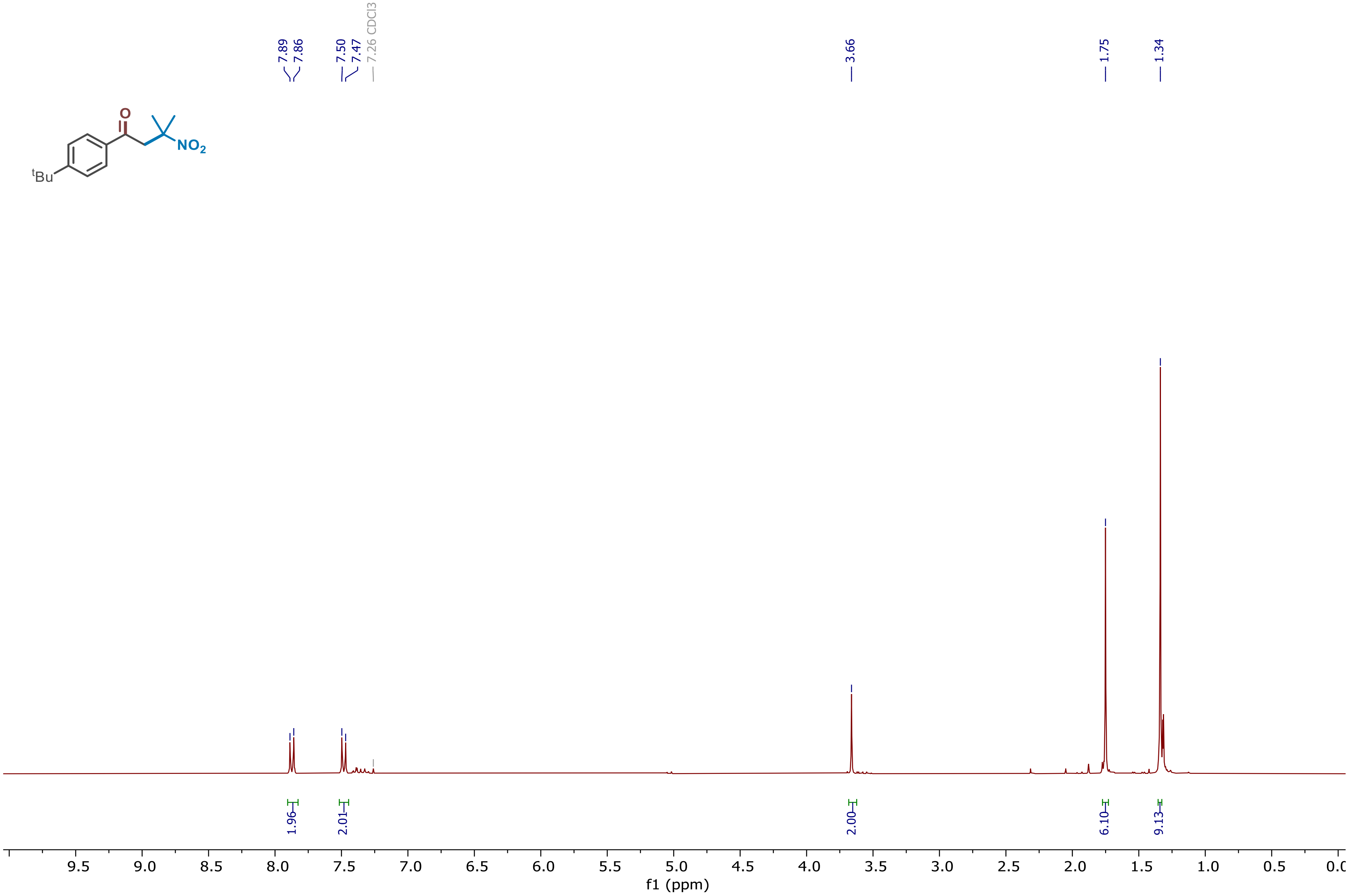

^{13}C NMR (75 MHz, CDCl$_3$) of **41**

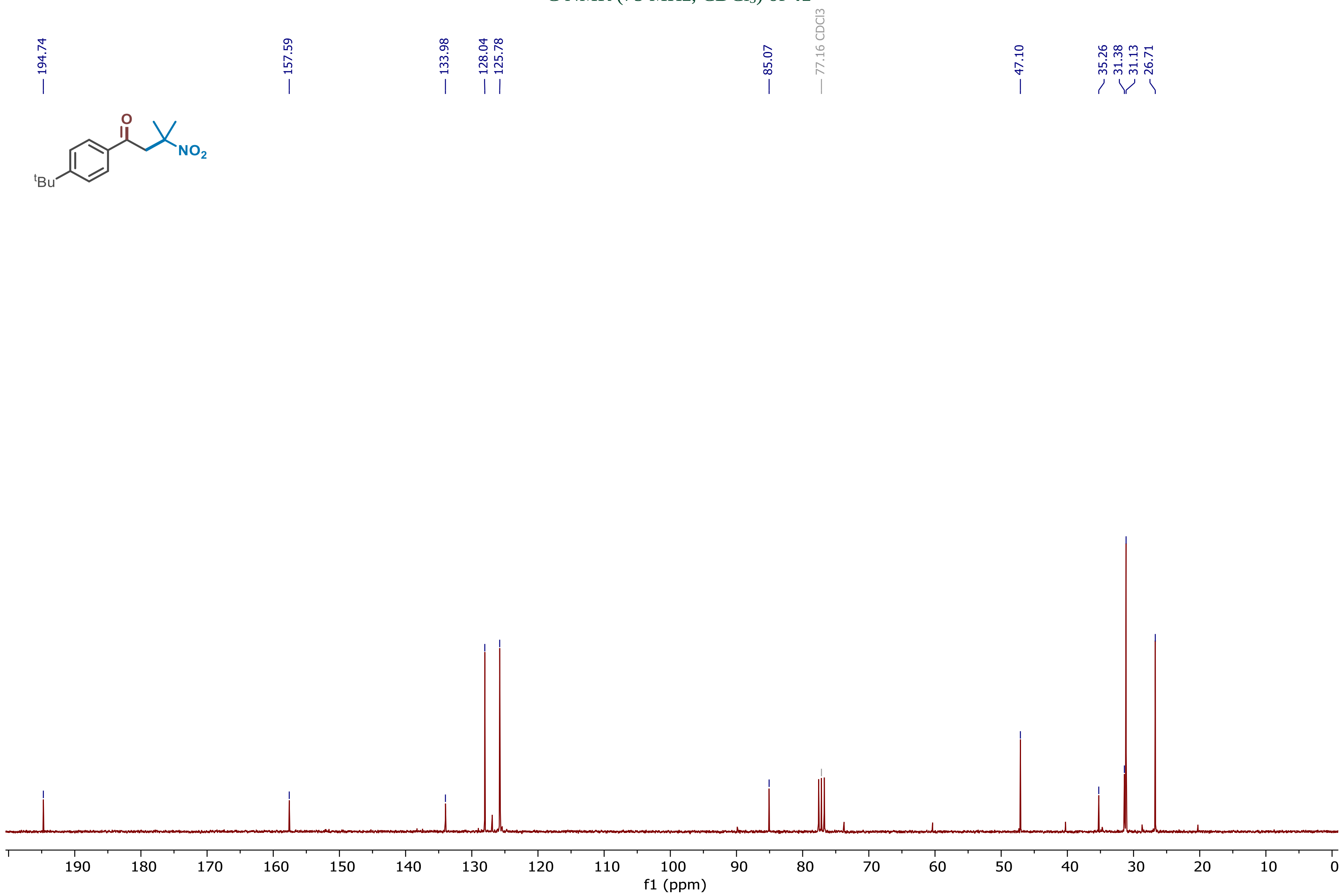

^{1}H NMR (300 MHz, $CDCl_3$) of **42**

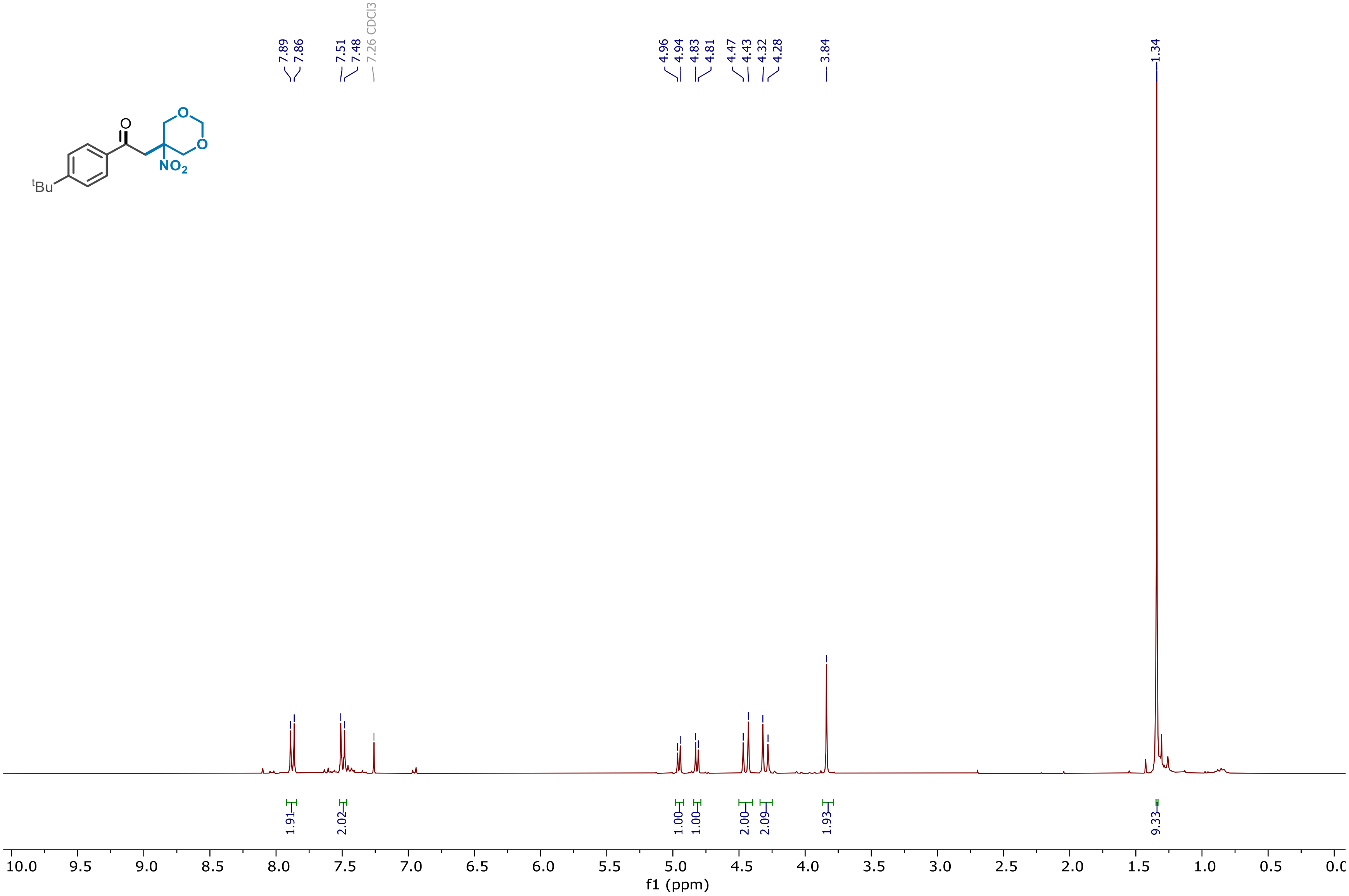

^{13}C NMR (75 MHz, $CDCl_3$) of **42**

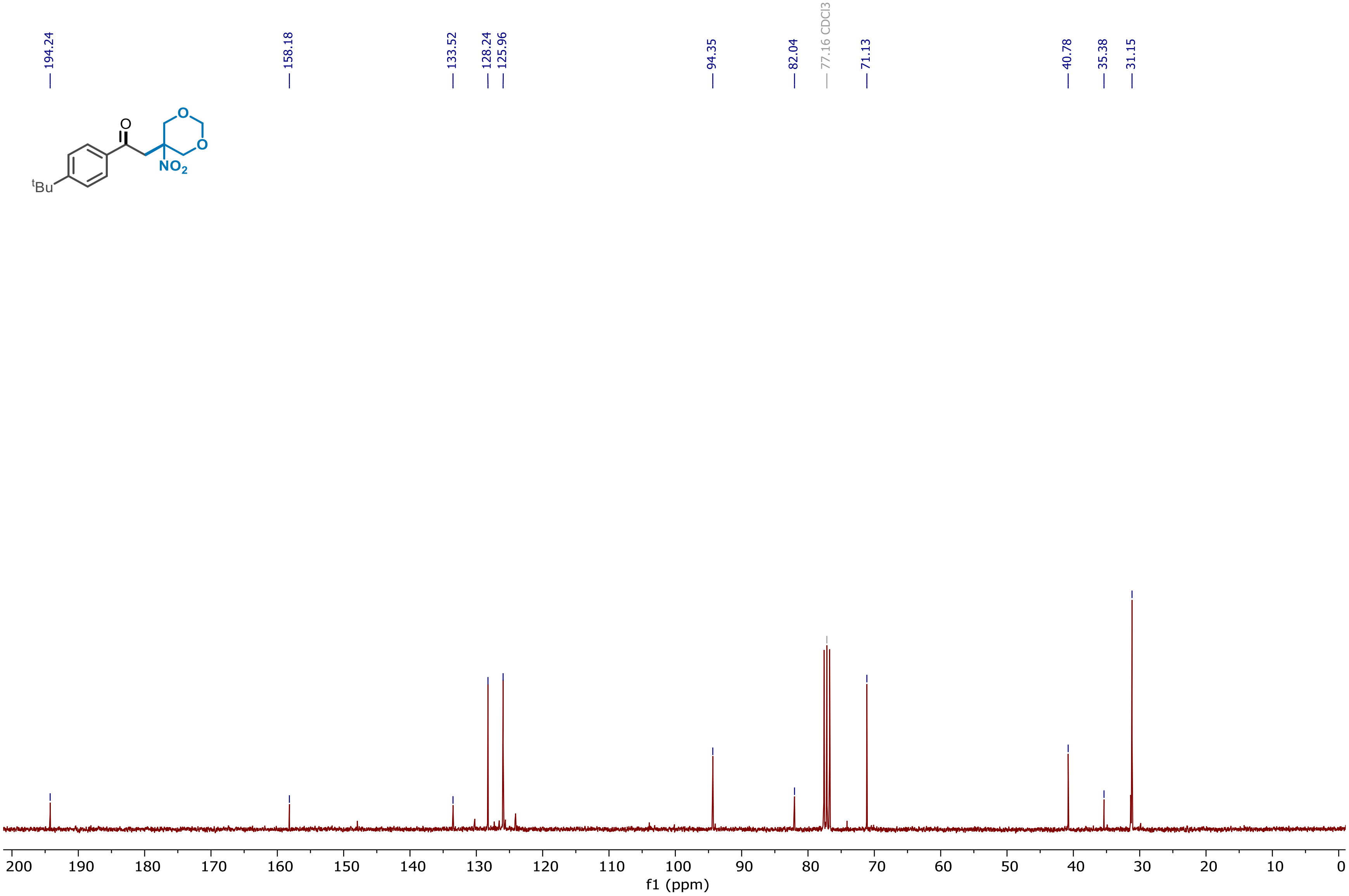

^{1}H NMR (300 MHz, $CDCl_3$) of **43**

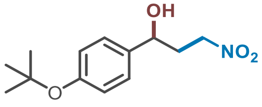

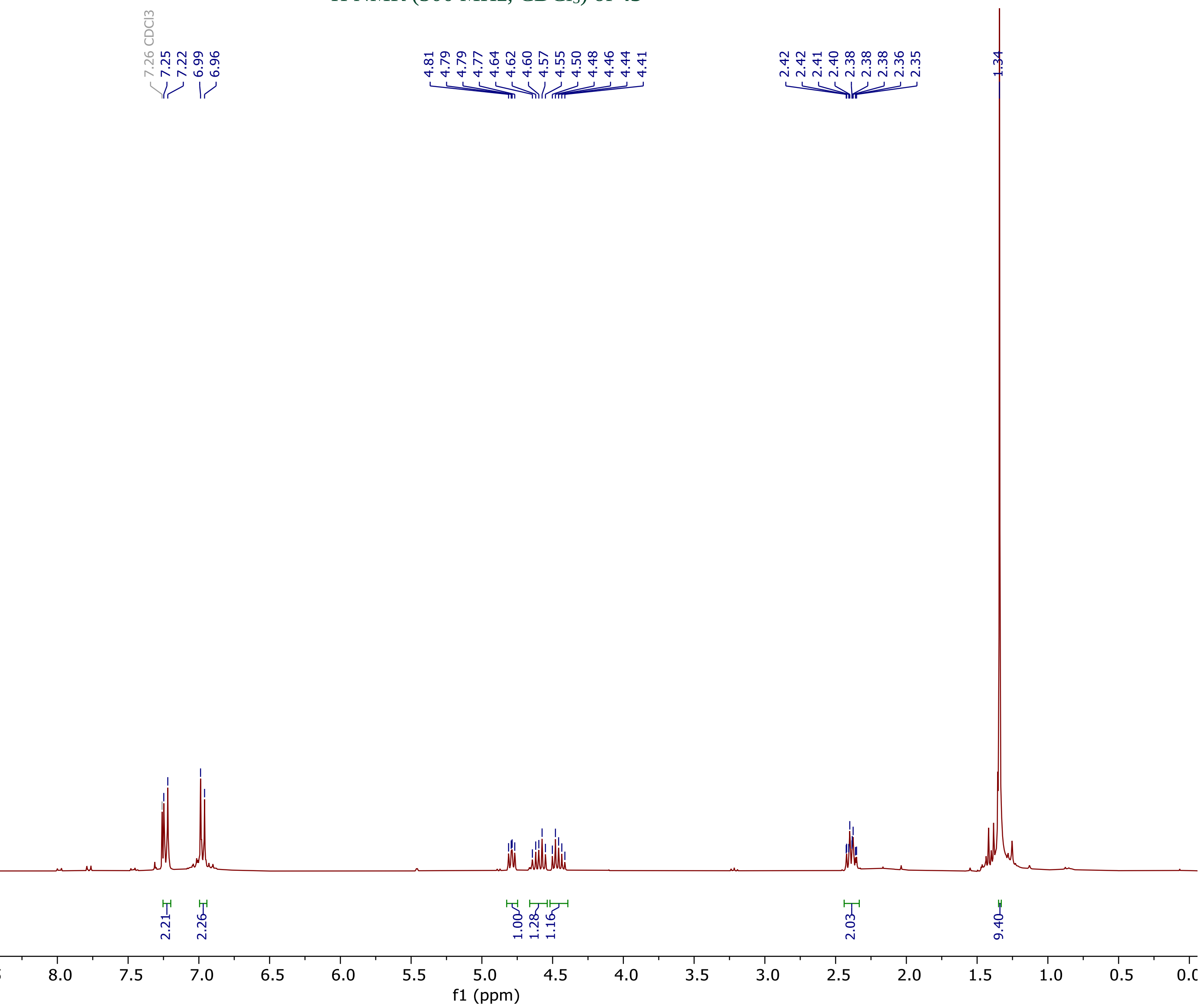

^{13}C NMR (75 MHz, $CDCl_3$) of **43**

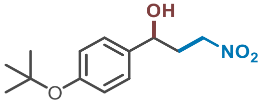

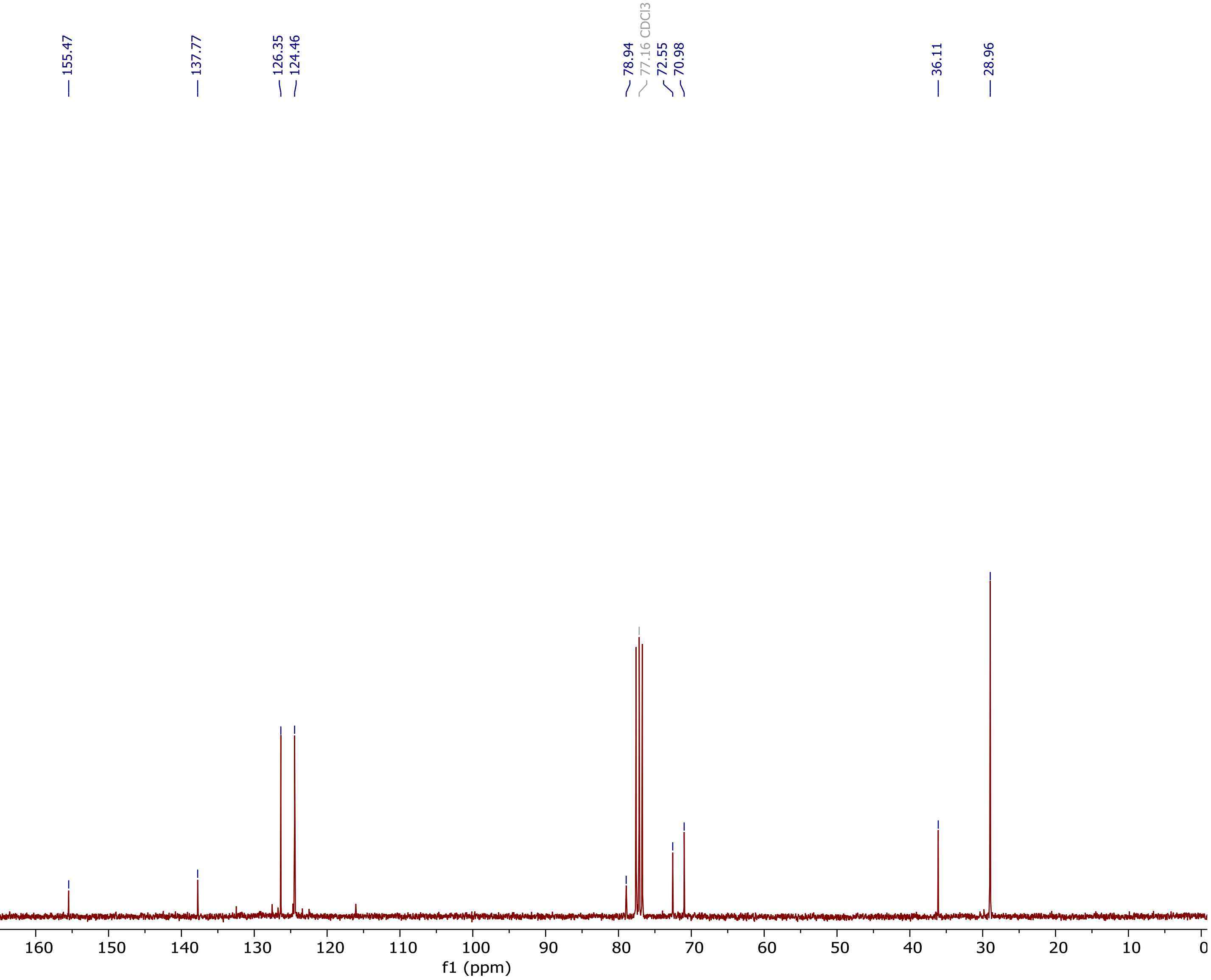

^{1}H NMR (300 MHz, $CDCl_3$) of **45**

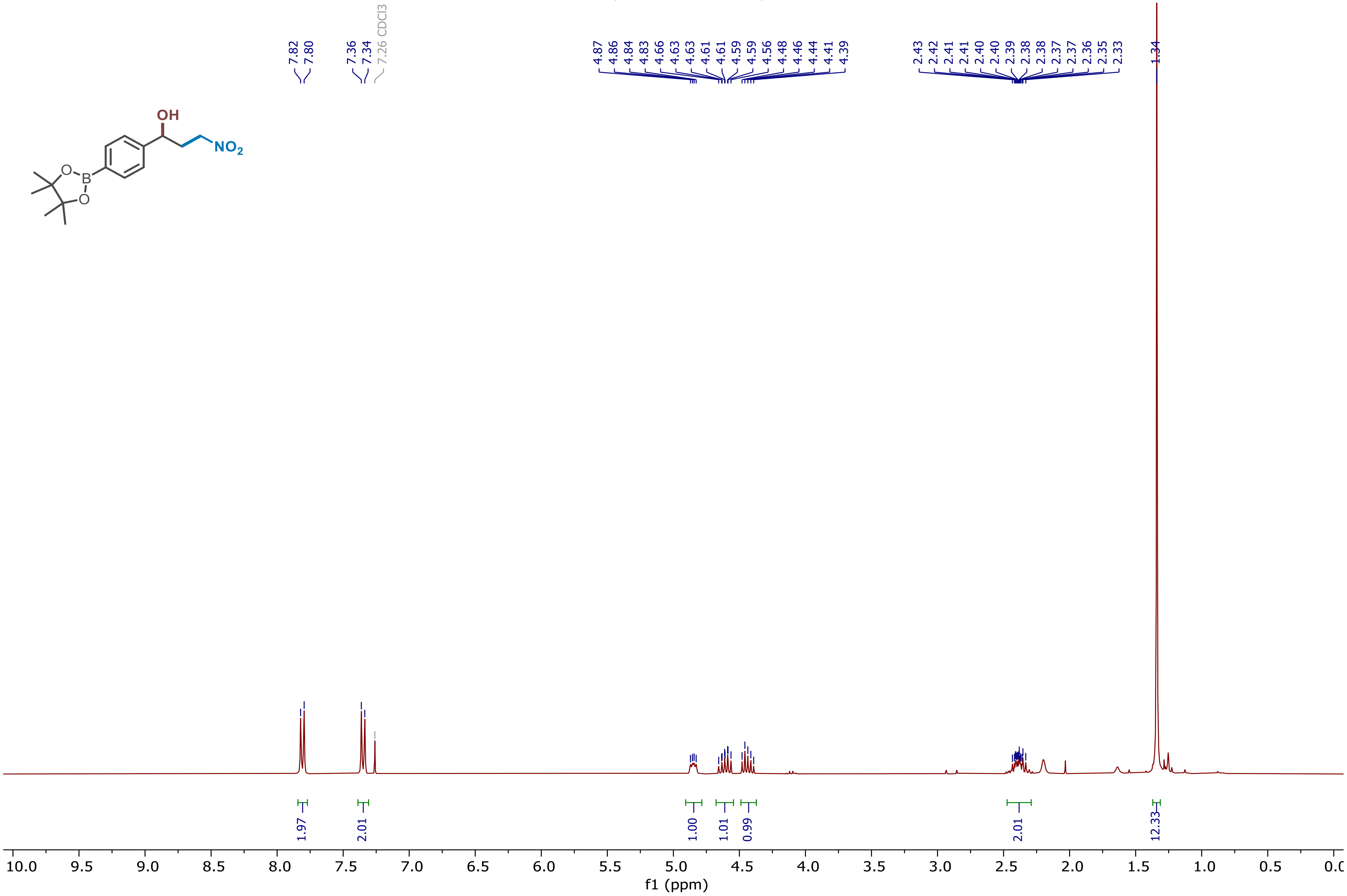

^{13}C NMR (75 MHz, CDCl$_3$) of **45**

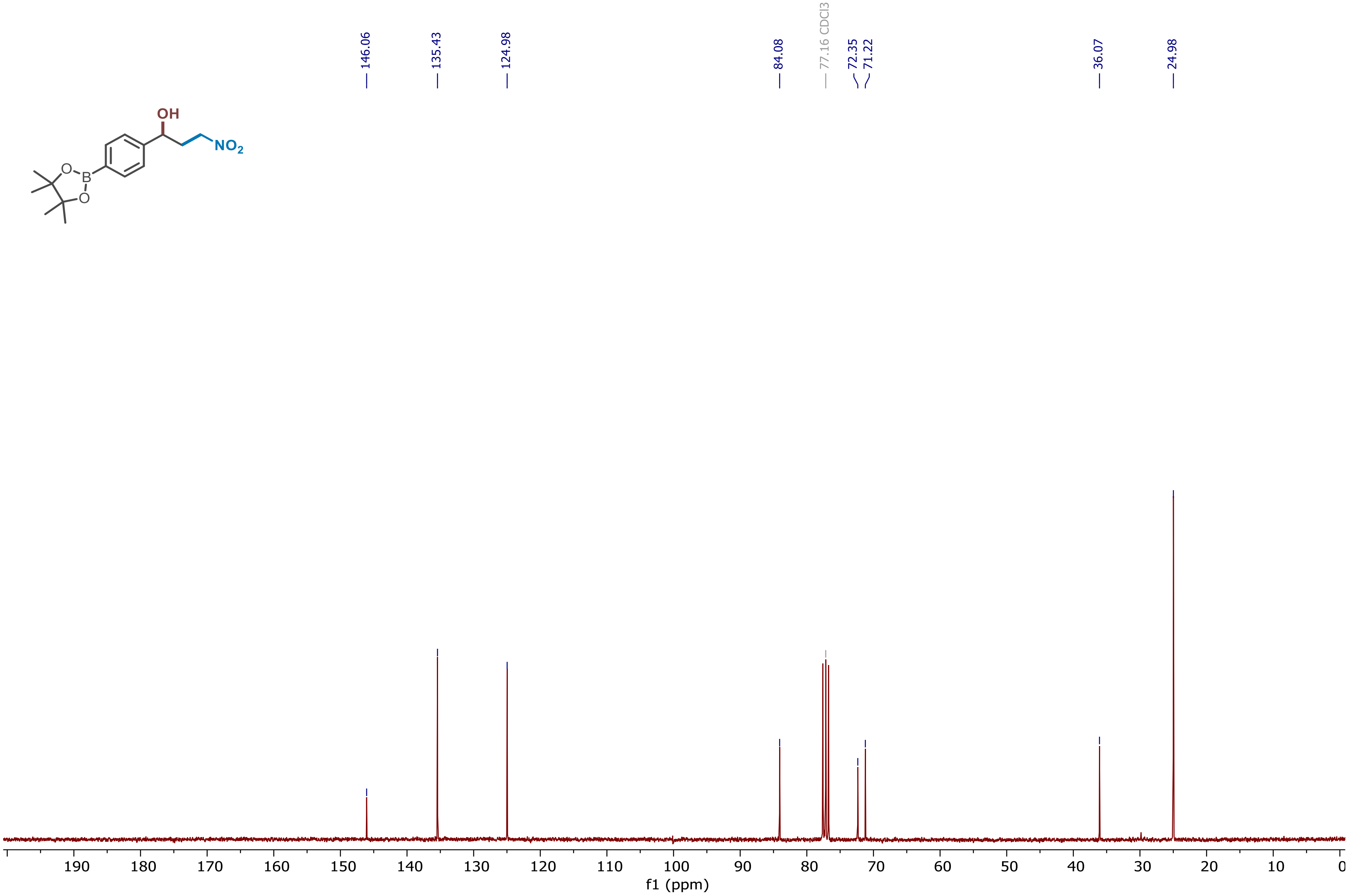

^{1}H NMR (300 MHz, $CDCl_3$) of **46**

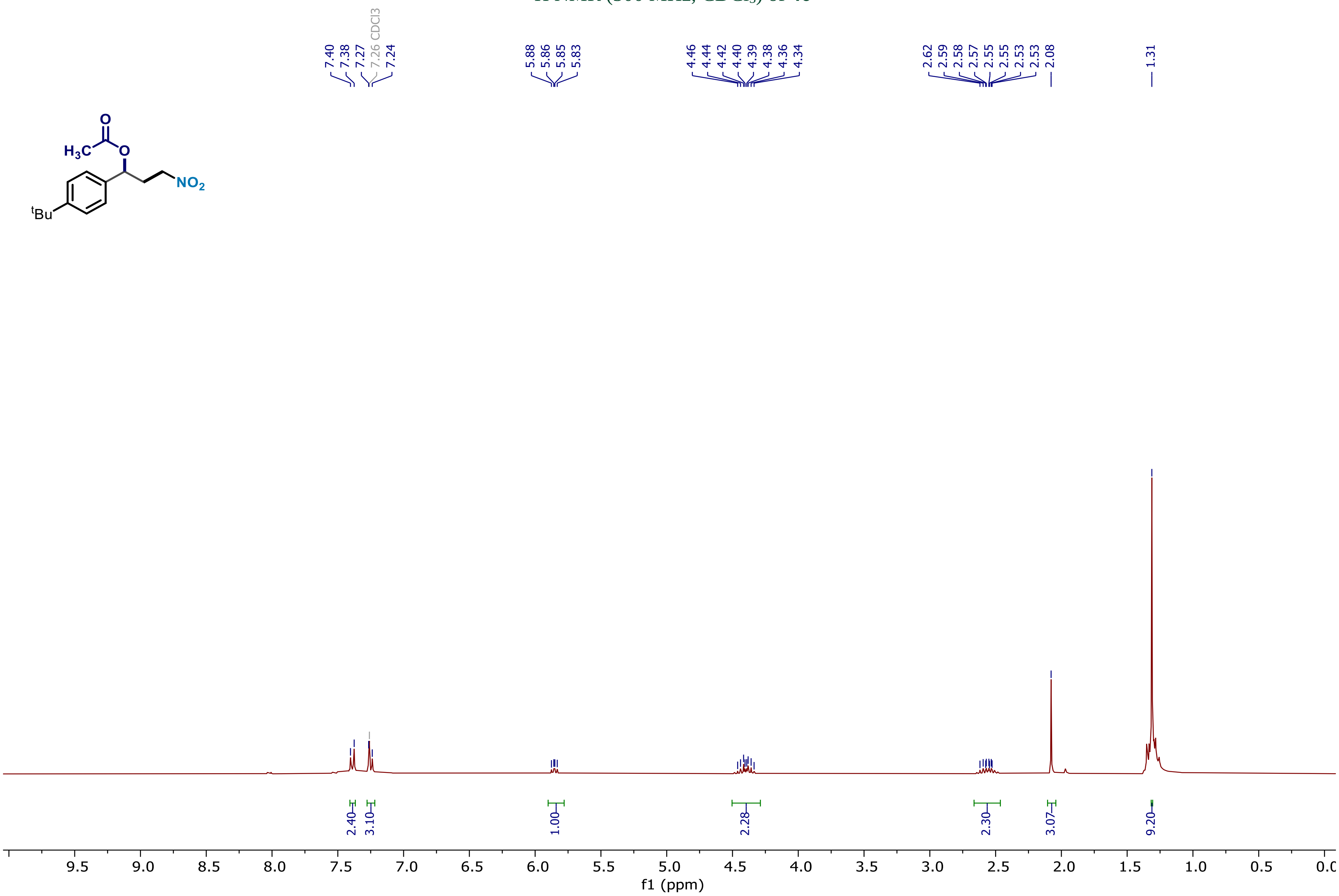

^{13}C NMR (75 MHz, $CDCl_3$) of **46**

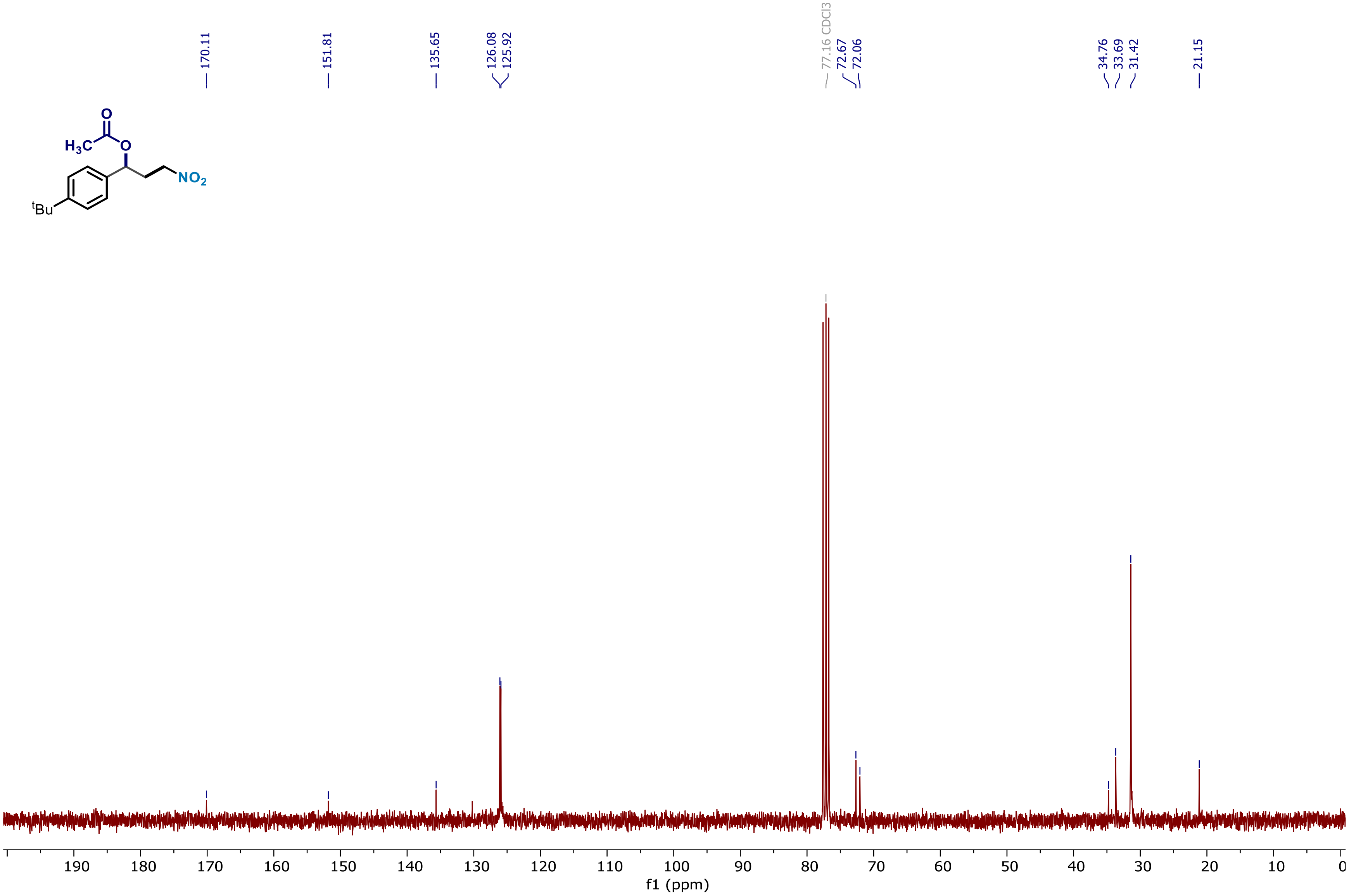

^{1}H NMR (300 MHz, $CDCl_3$) of **47**

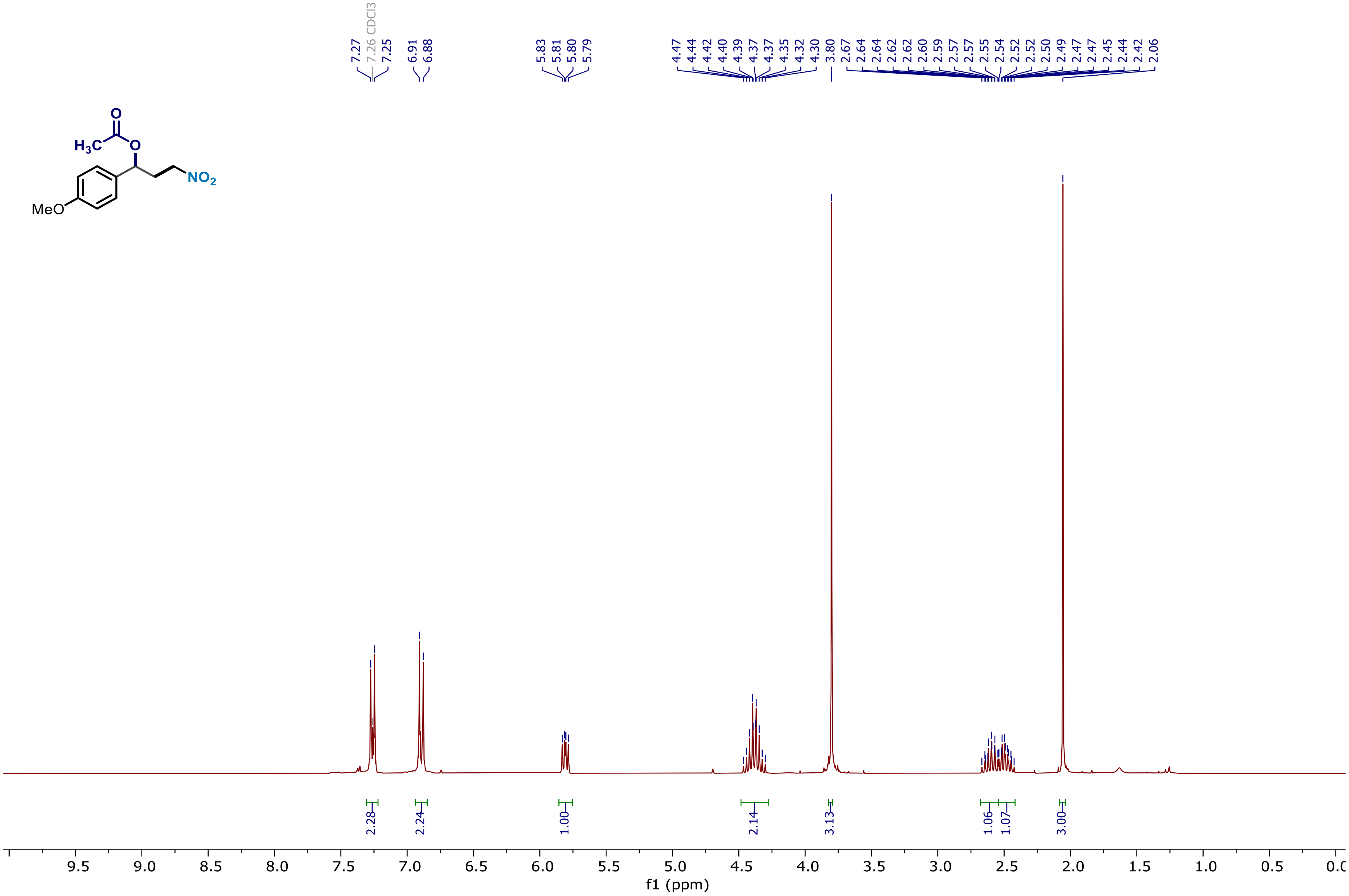

^{13}C NMR (75 MHz, $CDCl_3$) of **47**

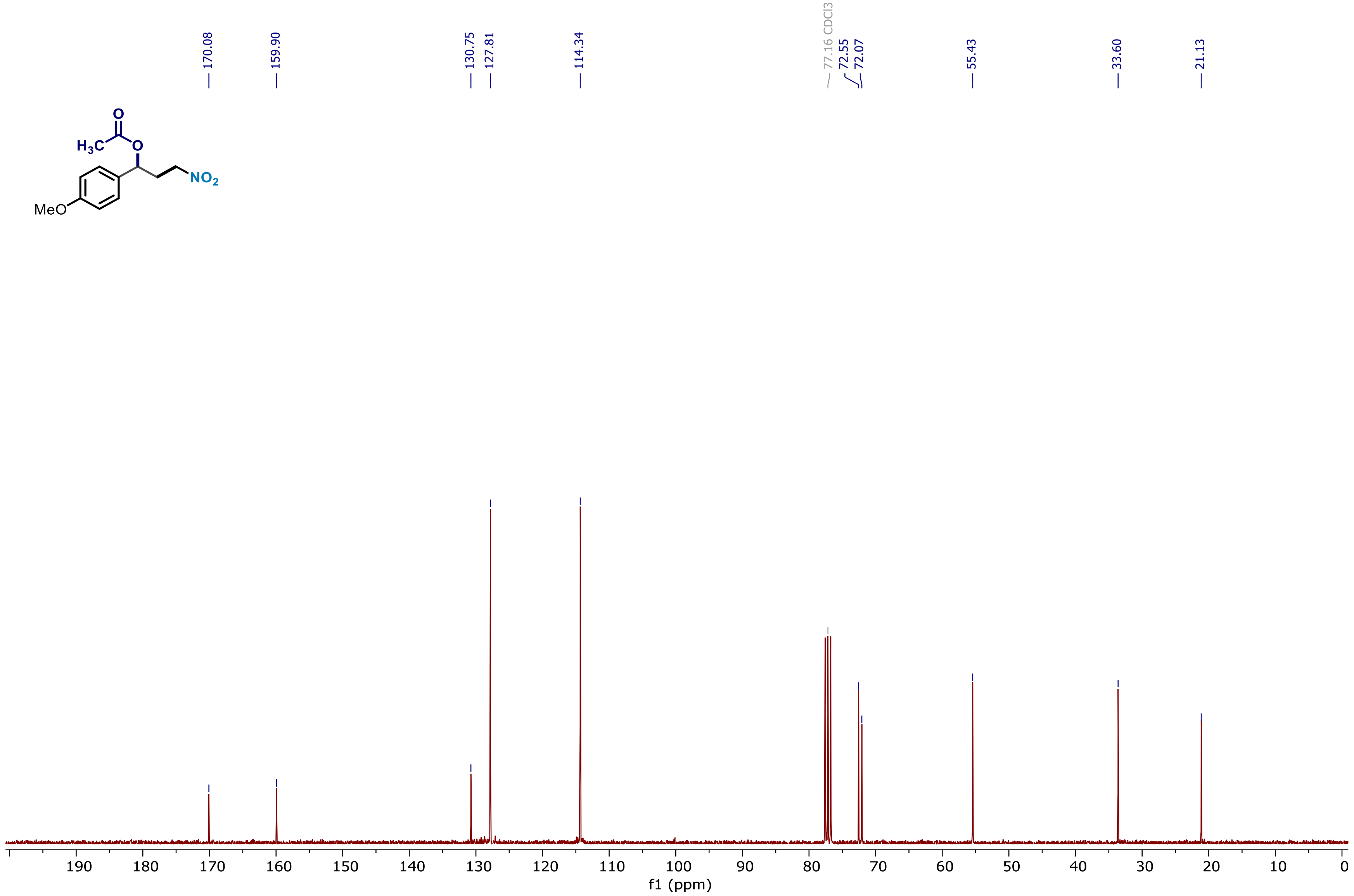

^{1}H NMR (300 MHz, $CDCl_3$) of **48**

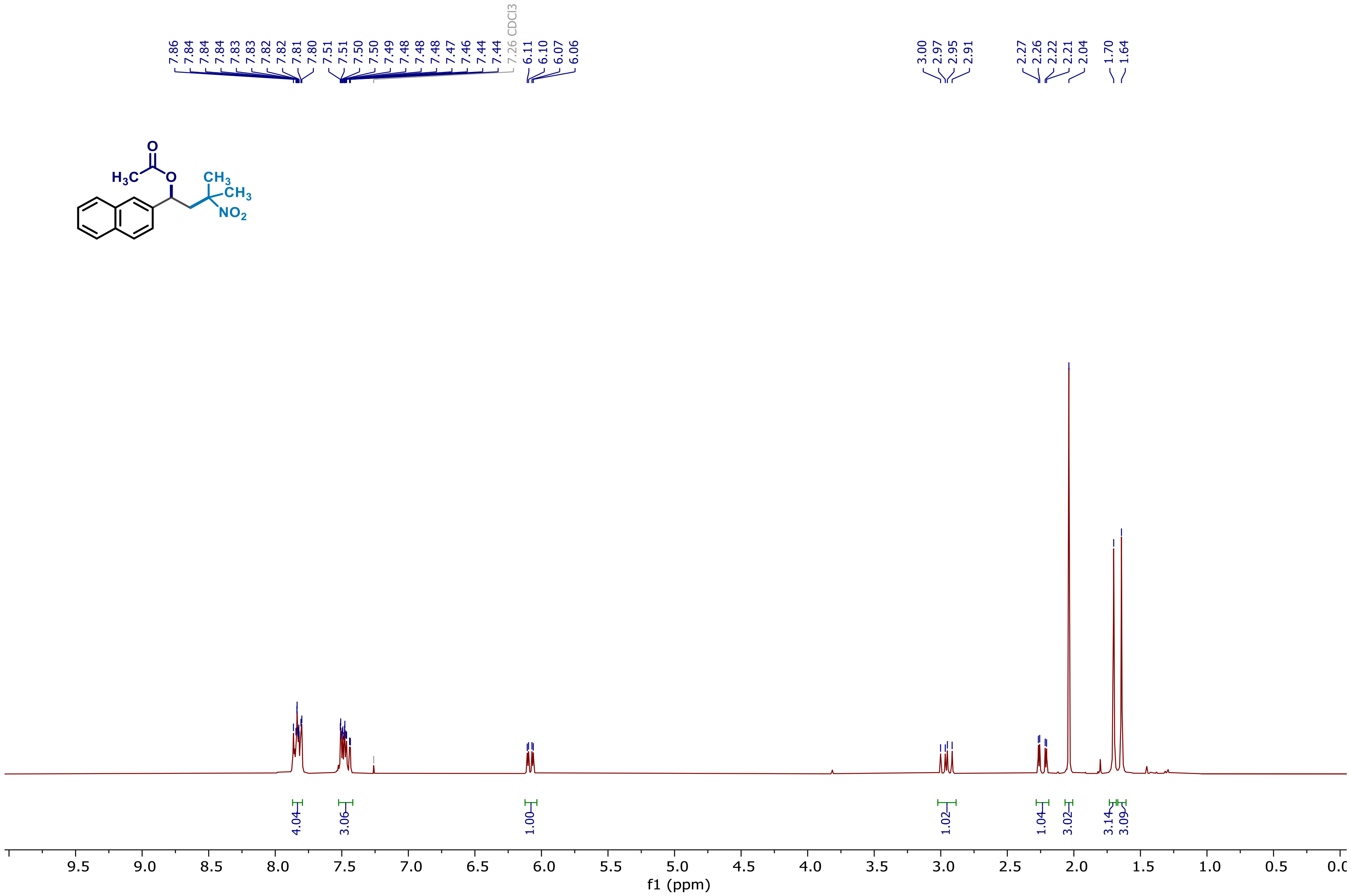

^{13}C NMR (75 MHz, $CDCl_3$) of **48**

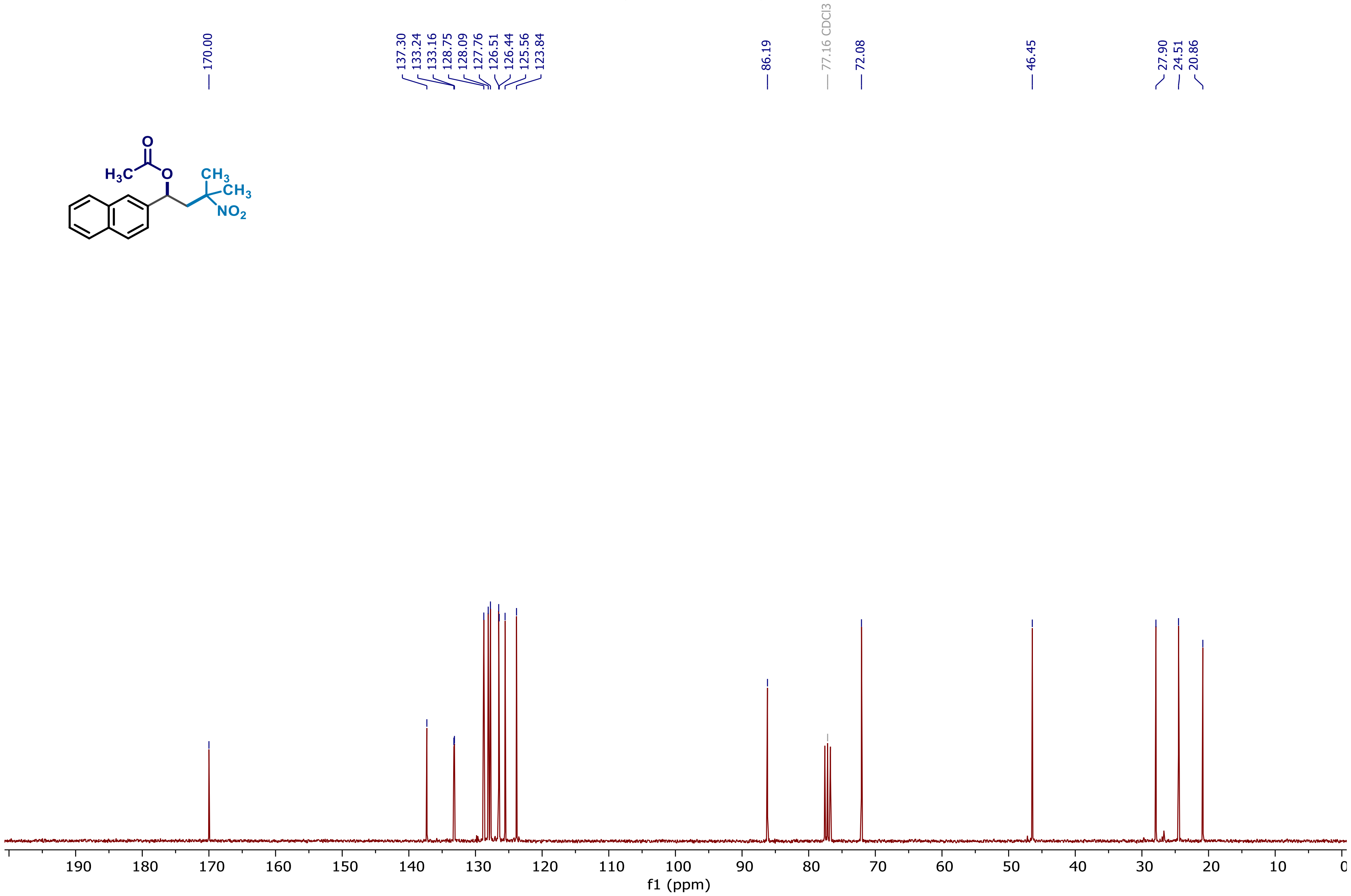

^{1}H NMR (300 MHz, $CDCl_3$) of **49**

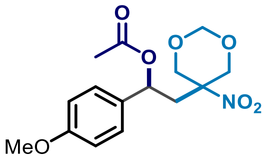

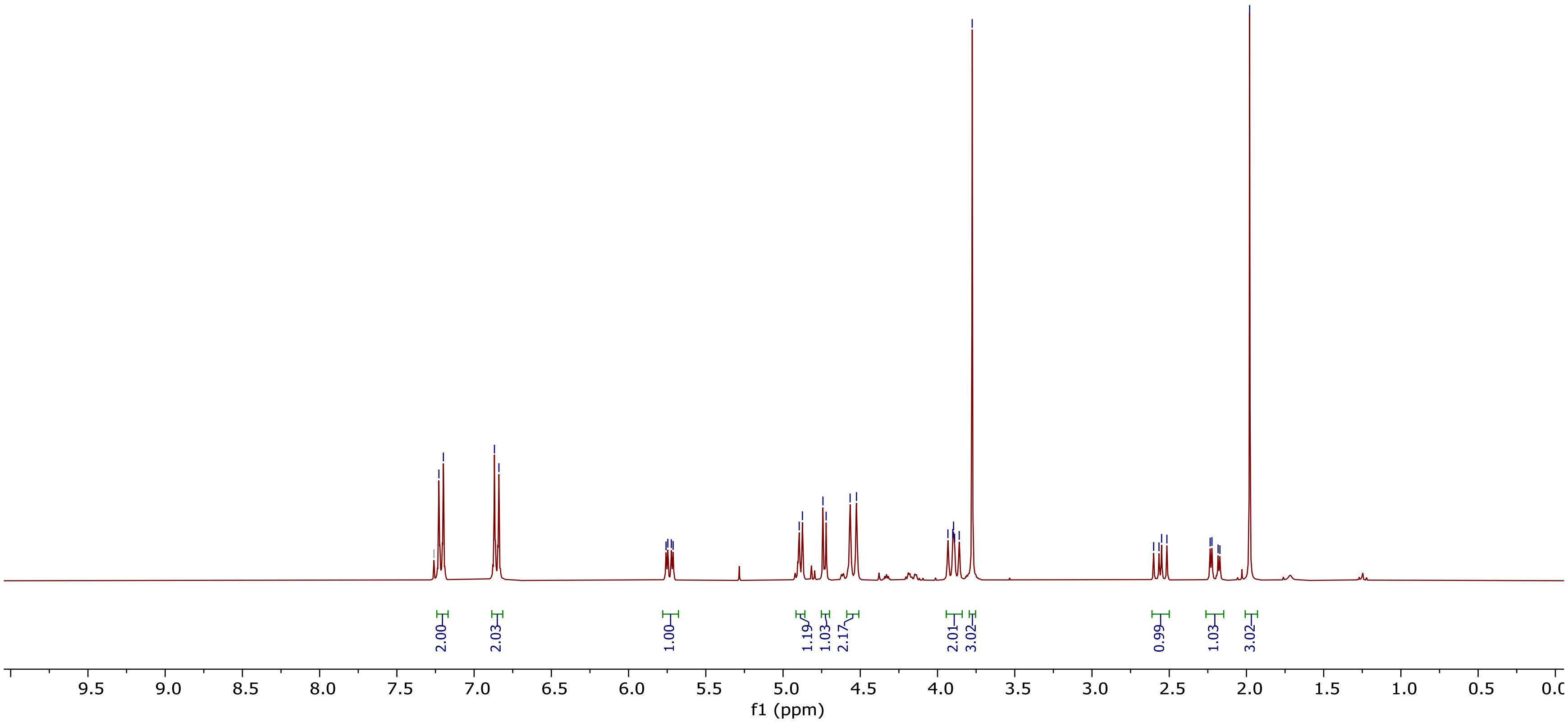

^{13}C NMR (75 MHz, CDCl$_3$) of **49**

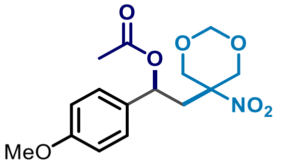

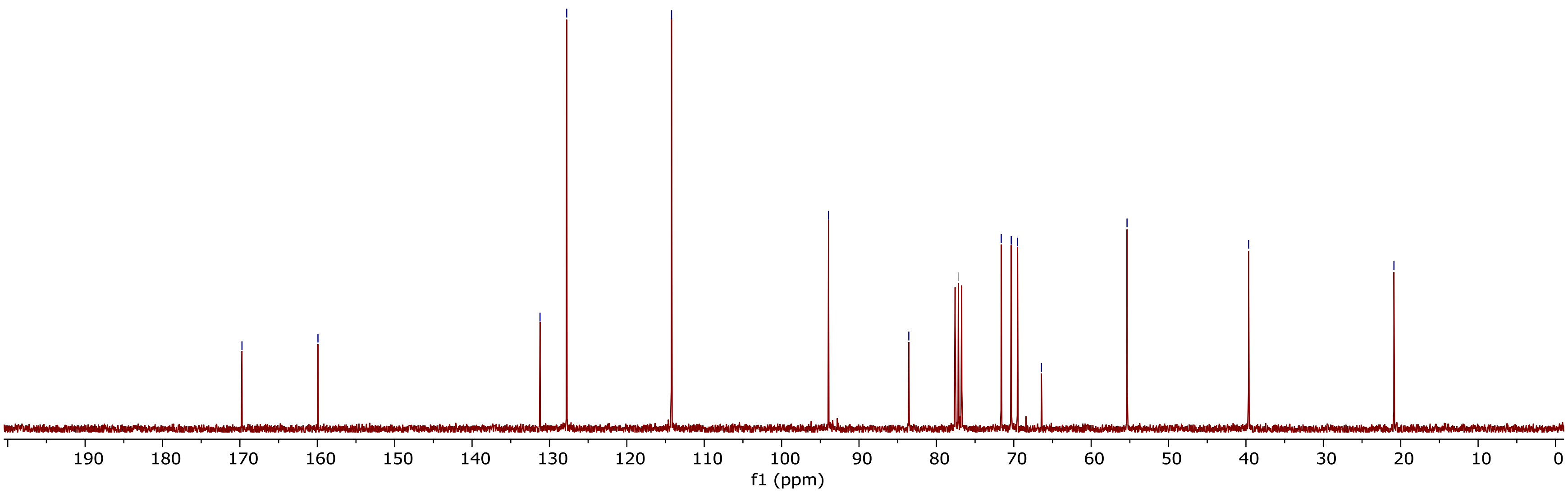

14. References

(1) S. Patra, R. Giri, D. Katayev, *ACS Catal*. **2023**, *13*, 16136–16147.

(2) M. K. W. Choi, P. H. Toy, *Tetrahedron* **2003**, *59*, 7171–7176.

(3) S. Patra, B. N. Nandasana, V. Valsamidou, D. Katayev. *Adv. Sci*. **2024**, *11*, 2402970.

(4) R. Giri, S. Patra, D. Katayev, *ChemCatChem*, **2023**, e202201427.

(5) S. Gabrielli, A. Palmieri, A. Perosa, M. Selva, R. Ballini, *Green Chem*., **2011**,*13*, 2026–2028.

(6) W. Zeng, X. Tan, Y. Yu, G.-Q. Chen, X.Zhang, *Org. Lett*. **2020**, *22*, 858–862.

(7) A. J. Fernandes, R. Giri, K. N. Houk, D. Katayev, *Angew. Chem. Int. Ed.* **2024**, *63*, e202318377.
